# Evolution and conservation of polycomb repressive complex 1 core components and putative associated factors in the green lineage

**DOI:** 10.1186/s12864-019-5905-9

**Published:** 2019-06-28

**Authors:** Yong Huang, Ling Jiang, Bo-Yu Liu, Cheng-Fang Tan, Dong-Hong Chen, Wen-Hui Shen, Ying Ruan

**Affiliations:** 1 Key Laboratory of Crop Epigenetic Regulation and Development in Hunan Province, Hunan Agricultural University, Changsha, 410128 China; 2 International Associated Laboratory of CNRS-FU-HAU on Plant Epigenome Research, Hunan Agricultural University, Changsha, 410128 China; 3 Key Laboratory of Plant Genetics and Molecular Biology of Education Department of Hunan Province, Hunan Agricultural University, Changsha, 410128 China; 4 State Key Laboratory of Subtropical Silviculture, SFGA Engineering Research Center for Dendrobium catenatum (D. officinale), Zhejiang A&F University, Hangzhou, 311300 China; 5 Institut de Biologie Mole’culaire des Plantes du CNRS, Universite’ de Strasbourg, 12 rue du Ge’ne’ralZimmer, 67084 Strasbourg Cedex, France

**Keywords:** Polycomb, PRC1, Phylogenetic analysis, Domain organization, Evolution

## Abstract

**Background:**

Polycomb group (PcG) proteins play important roles in animal and plant development and stress response. Polycomb repressive complex 1 (PRC1) and PRC2 are the key epigenetic regulators of gene expression, and are involved in almost all developmental stages. PRC1 catalyzes H2A monoubiquitination resulting in transcriptional silencing or activation. The PRC1 components in the green lineage were identified and evolution and conservation was analyzed by bioinformatics techniques. RING Finger Protein 1 (RING1), B lymphoma Mo-MLV insertion region 1 homolog (BMI1), Like Heterochromatin Protein 1 (LHP1) and Embryonic Flower 1 (EMF1) are the PRC1 core components and Vernalization 1 (VRN1), VP1/ABI3-Like 1/2/3 (VAL1/2/3), Alfin-like 1–7 (AL1–7), Inhibitor of growth 1/2 (ING1/2), and Early Bolting in Short Days (EBS) / Short Life (SHL) are the associated factors.

**Results:**

Each PRC1 subunit possesses special domain organizations, such as RING and the ring finger and WD40-associated ubiquitin-like (RAWUL) domains for RING1 and BMI1, chromatin organization modifier (CHROMO) and chromo shadow (ChSh) domains for LHP1, one or two B3 DNA binding domain(s) for VRN1, B3 and zf-CW domains for VAL1/2/3, Alfin and Plant HomeoDomain (PHD) domains for AL1–7, ING and PHD domains for ING1/2, Bromoadjacent homology (BAT) and PHD domains for EBS/SHL. Six new motifs are uncovered in EMF1.

The PRC1 core components RING1 and BMI1, and the associated factors VAL1/2/3, AL1–7, ING1/2, and EBS/SHL exist from alga to higher plants, whereas LHP1 only occurs in higher plants. EMF1 and VRN1 are present only in eudicots. PRC1 components undergo duplication in the plant evolution. Most of plants carry the homologous core component LHP1, the associated factor EMF1, and several homologs in RING1, BMI1, VRN1, AL1–7, ING1/2/3, and EBS/SHL. Cabbage, cotton, poplar, orange and maize often exhibit more gene copies than other species. Domain organization analysis shows that duplicated gene functions may be of diverse.

**Conclusions:**

The PRC1 core components RING1 and BMI1, and the associated factors VAL1/2/3, AL1–7, ING1/2, and EBS/SHL originate from algae. The core component LHP1 is from moss and the associated factors EMF1 and VRN1 are from dicotyledon. PRC1 components are of functional redundancy and diversity in evolution.

**Electronic supplementary material:**

The online version of this article (10.1186/s12864-019-5905-9) contains supplementary material, which is available to authorized users.

## Background

Polycomb-group (PcG) proteins play essential roles in epigenetically repressing or activating genes transcription in animals and plants, and are implicated in stable and heritable transcriptional silencing or activating of target genes during organism development [1–5]. PcG proteins are evolutionarily conserved and are involved in various aspects of plant development, such as the timing of flowering and seed development [3, 6–8], and response to abiotic and biotic stresses [9]. In contrast with animal PcG proteins, which are involved in maintaining pluripotency and preventing cell differentiation, plant PcG proteins are required to promote cell differentiation by suppressing embryonic development [10].

Polycomb proteins can form at least three distinct complexes in metazoan, namely polycomb repressive complex 1 (PRC1), PRC2 and polycomb-like PRC2 (Pcl-PRC2) [11]. PRC1 and PRC2 complexes were found earlier than Pcl-PRC2 [12–14]. PRC2 is attributed to the trimethylation of lysine-27 on histone H3 (H3K27me3) [15]. Pcl-PRC2 is required to generate high levels of H3K27me at polycomb target genes [16]. PRC1 catalyzes H2A monoubiquitination (H2Aub) and causes transcriptional silencing after histone H3K27me induced by PRC2 or via an unknown mechanism [17].

PRC1 was previously believed to be the major determinant in silencing genes via histones H2A ubiquitination [18]. PRC1 also facilitates transcription of many genes [19]. PRC1 maintains gene silencing through interfering with recruited mediators to inhibit activated RNA polymerase II preinitiation complex assembly [20]. Histone H3K27me induced by PRC2 provides a binding site for the N-terminal chromodomain of the PRC1 Pc subunit; furthermore, PRC1 catalyzes H2Aub via its RING1a/b subunit, the Really Interesting New Gene (RING) domain of which exhibits E3 ubiquitin ligase activity [18, 20, 21]. BMI1 and RING1a positively regulate H2A ubiquitylation, resulting Hox gene silencing [22]; BAH domain proteins EBS/SHL–EMF1 complex mediates genome-wide transcriptional repression by effecting H3K27me3 [23]. PRC1 directly influences the transcription of active genes. PRC1 subunit mutations alter the phosphorylation of RNA polymerase II and occupancy by the Spt5 pausing-elongation factor at most active genes [19]. RING1a and BMI1 also activate genes via chromatin-associated protein Ubiquitination [24, 25].

**Fig. 1:**
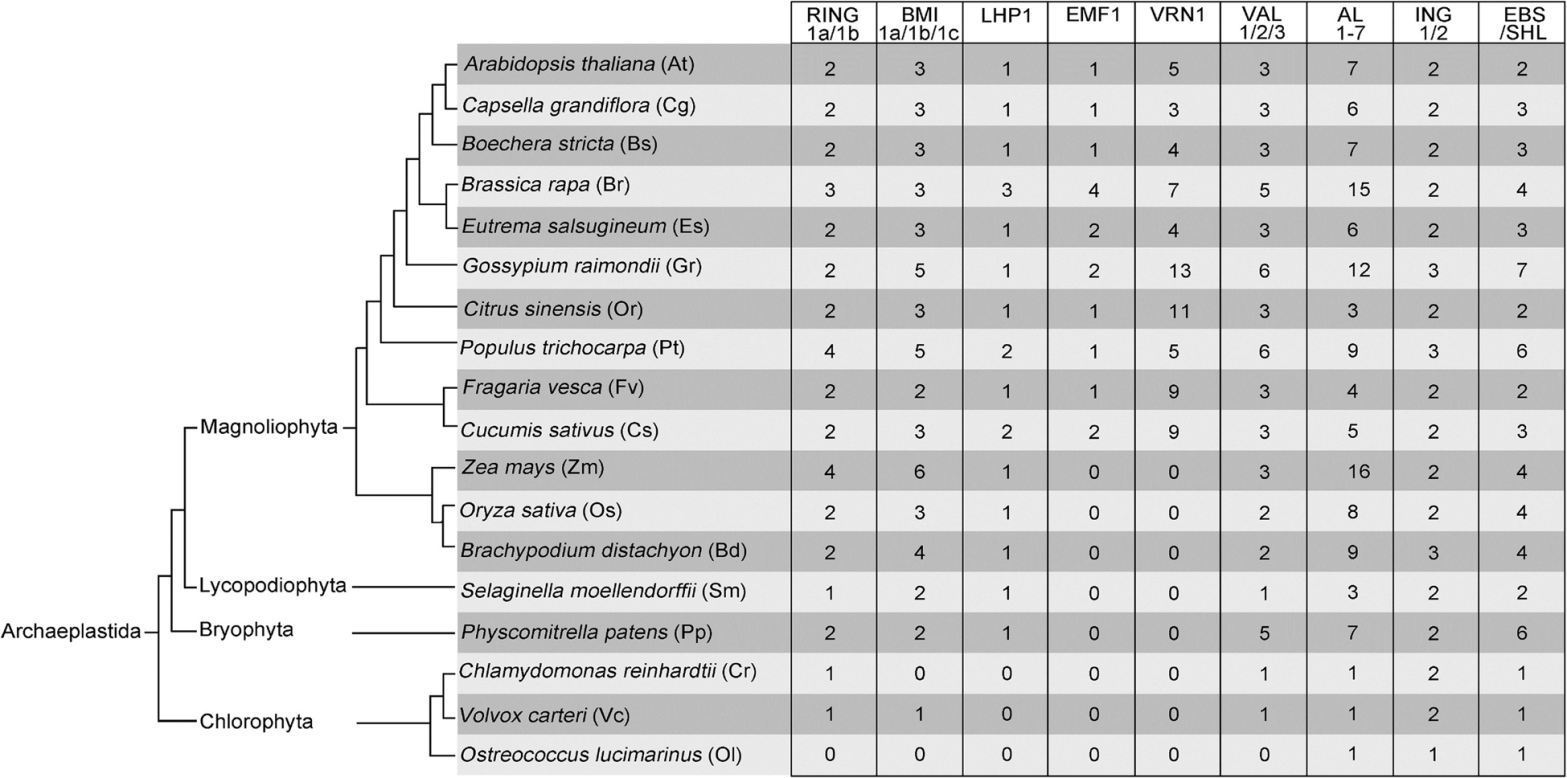
PRC1 proteins in representative plants. Numbers show the copy numbers of homologous proteins in each plant. Data were derived from Phytozome 12.0 (http://www.phytozome.net) and *Brassica* Database (http://brassicadb.org/brad/)

Drosophila core PRC1 is composed of four subunits, Polycomb (Pc), Posterior sex combs (Psc), Polyhomeotic (Ph) and Sex combs extra (Sce), but only Pc, Psc and Sce are found in plants [2, 26]. In plants, PRC1 was thought to be absent until *Arabidopsis* RING finger proteins including RING1a/b and BMI1a/b/c have been characterized. Plant PRC1 participates in multiple processes during vegetative and reproductive development, such as the control of stem cell fate determinacy, the prevention of vegetative-to-embryogenesis conversion, flowering timing, floral organ number and identity, and seed development [27, 28]. *Arabidopsis* PRC1 complex comprises of three core components, ring finger protein 1 (RING1a/1b), B lymphoma Mo-MLV insertion region 1 homolog (BMI1a/1b/1c) and like heterochromatin protein 1(LHP1), and six associated components, Embryonic Flower 1 (EMF1), Vernalization 1 (VRN1), VP1/ABI3-Like 1/2/3 (VAL1/2/3), Alfin-like 1–7 (AL1–7), Inhibitor of growth (ING1/2), and Early Bolting in Short Days /Short Life (EBS/SHL) (Fig. 1, Additional file 1). PRC1 complexes, including AtRING1/AtBMI1-PRC1, EMF1-PRC1 and PRC2-H3K4 demethylase-PRC1, have been proposed [29]. The two prominent roles of PRC1 complex are: as a reader of H3K27me3, LHP1 with specific H3K27me3 binding depending on the chromatin organization modifier (CHROMO) domain; as a writer of H2Aub1, RING1 and BMI1 providing ubiquitin ligase activity relied on RING domains [19, 30]. EBS and SHL are bivalent H3K27me3 and H3K4me3 readers [31]. However AtRING1s and AtBMI1s are required for H2AK119ub, but not for PRC2 induced H3K27me3 [29].

The PRC1 subunit EMF1 cooperates with PRC2 to repress key regulators in *Arabidopsis* development [32]; however EMF1 and VRN1 exist in dicotyledon species [27]. VRN1 and VAL1/2/3 are plant-specific components of PRC1 [29]. VAL1 and PRC1 form a complex via interaction with AtBMI1a/1b [17, 33]. The VAL1-PRC1 complex maintenances differentiated state, such as seed maturation, through catalyzing H2Aub by specifically binding to gene promoters [17, 34]. PRC1 also acts as both a reader of H3K4me3 and a writer of H2Aub1 in establishing a stable repression chromatin state [35]. AL proteins are novel interactors of the PRC1 core components AtBMI1a and AtRING1b [36]. AL proteins target PRC1 to form AL PHD-PRC1 complexes with active chromatin for transitioning from H3K4me3 to H2Aub1/H3K27me3 to establishing gene silencing [35]. Epigenetic regulatory protein ING proteins are characterized by a nuclear localization sequence and a high affinity to H3K4me3; however the role of these proteins as associated factors of PRC1 remains unclear [37]. Lee et al. showed that ING and AL are nuclear proteins involved in chromatin regulation through binding to H3K4me3/2 [38]. ING1 is regulated in response to activity by binding to a site upstream of the Ppp3r1 transcription start site [39]. EBS and SHL, the plant-unique BAH-domain containing proteins, implement Polycomb silencing or activating by forming a complex with EMF1, and function as such as floral phase transition [23, 31].

PRC1 are highly divergent between animals and plants. Core components and associated subunits of PRC1 form functional complex. The PRC1 core components RING-finger proteins (RING1 and BMI1) and LHP1 exist in the ancestor of seed plants, whereas EMF1, VRN1, and EBS/SHL have been recently identified [27, 31, 40, 41]. RING1, BMI1 and LHP1 proteins have originated from mosses [42]. However the evolution of PRC1 associated factors, VRN1, EBS/SHL, VAL, AL and ING, remains unclear. Core components and associated of PRC1 are distributed in different plant species. The rapidly accumulating data of sequenced plant genomes allows to conduct systematic studies on plant gene evolution and functional differentiation. Therefore, this study aims to analyze the evolution and conservation of PRC1 core components and newfound associated factors, especially domain organizations and genes duplication, in the green lineage from lower to higher plants. The results would provide information for understand the evolution and conservation of plant PCR1.

## Results

### PRC1 core component: RING1

**Fig. 2:**
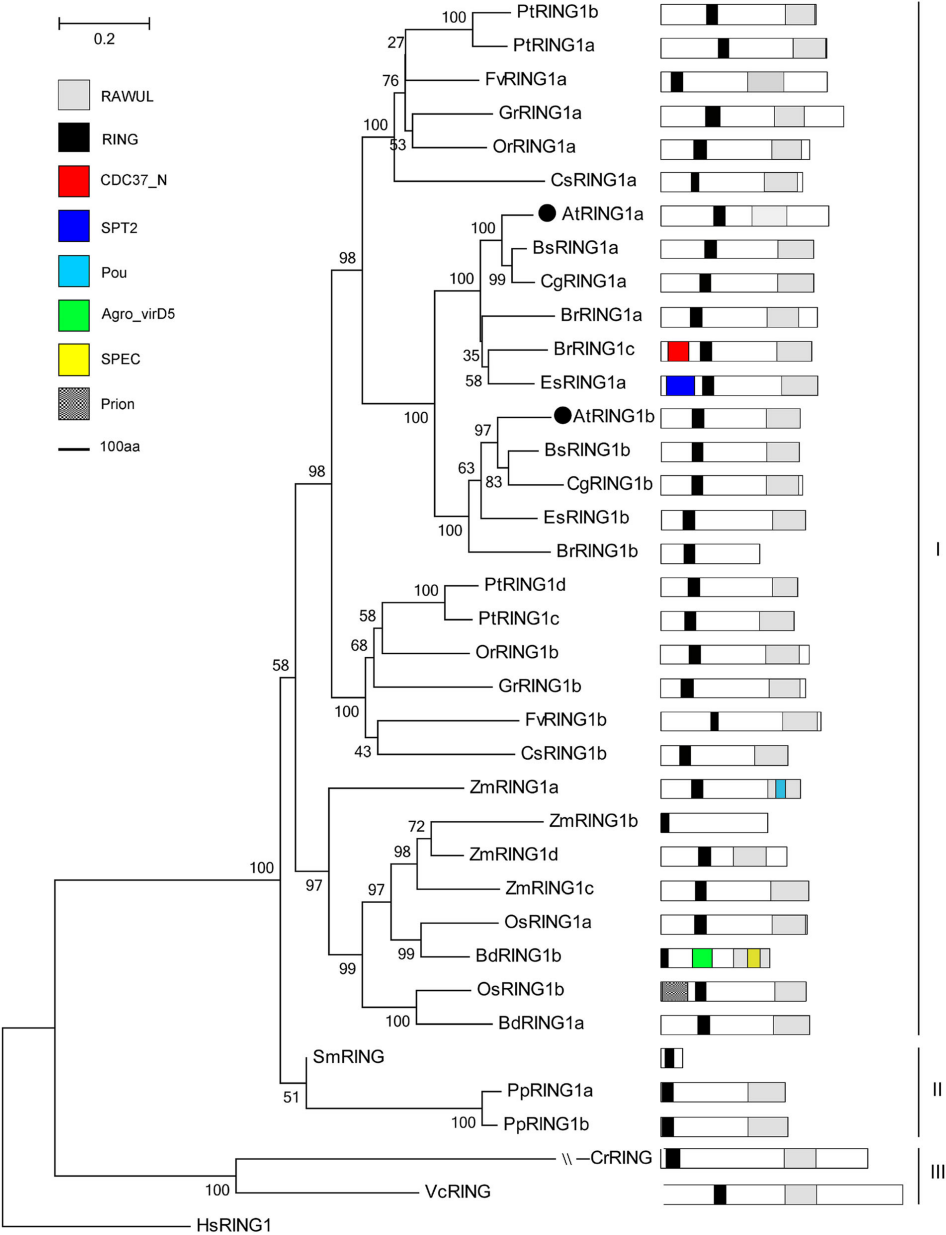
Phylogenetic tree of RING1 proteins in the Green Lineage. Plant RING1 homologs exist from algae to higher plants, and were subgrouped into three clades, Group-I seed plant, Group-II moss-fern, and Group-III algae. RING and RAWUL domains are the essential features of RING1 proteins

**Fig. 3:**
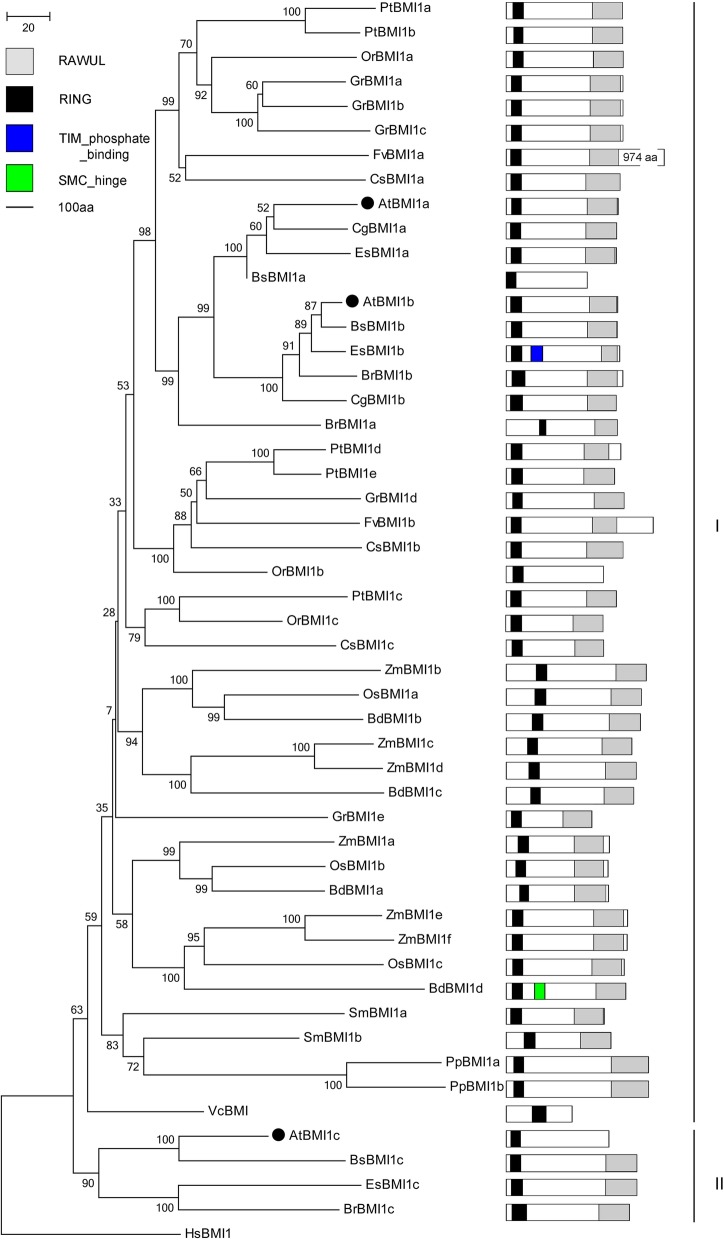
Phylogenetic tree of BMI1 proteins in the Green Lineage. Plant BMI1 homologs exist from algae to higher plants, and were subgrouped into two clades: Group-I BMI1a/1b homologs existing from algae to higher plant; and group-II BMI1c homologs specially in Cruciferae. RING and RAWUL domains are the essential features of BMI1 proteins

PRC1 RING finger proteins are composed of the two clades (Additional file 2), RING1 and BMI1, both of which are characterized by a conserved combination of RING and Ring-finger And WD40 associated Ubiquitin-Like (RAWUL) domains (Figs. 2 and 3). The ubiquitin ligase activity of PRC1 complex writers relies on their RING-domain [27]. In animals, RING1b is a key H2Aub writer, whereas RING1a plays minor roles. BMI1 exhibits no E3 ligase activity but can stabilize and enhance RING1b functions [43, 44]. In *Arabidopsis*, both AtRING1a/b and AtBMI1a/b/c families can catalyze H2Aub. At the vegetative stage, AtRING1a/1b can repress vegetative-to-embryonic transition and ectopic meristem formation mainly via suppressing the mis-expression of embryo master regulators and stem cell regulators, respectively [45, 46]. At the reproductive stage, the *Arabidopsis* double mutant *ring1a;ring1b* exhibits exceedingly high numbers of floral organs, and strong phenotypes displaying dramatic swelling gynoecium and completely sterile [45]. Both AtRING1a and AtRING1b can control the maintenance of floral stem cells and proper carpel development by repressing KNOX-I expression [42]. RING1a/b mutation can cause early vegetative phase transition by regulating the H2Aub status at the *SPL* locus [47].

In a phylogenetic tree, plant RING1 proteins can be divided into three branches: seed plant (Group-I), moss-fern (Group-II), and algae (Group-III, Fig. 2). The phylogenetic relationship of RING1 homologs is consistent with plant evolution. RING1 has undergone one and two duplications in eudicot and monocot ancestors, respectively [40]. Most RING1 proteins exhibit two copies in each species, but PtRING1 and ZmRING1 occur in four copies, and BrRING1 present in three copies. However, the duplication event may occur after the separation of monocotyledons and dicotyledons. RING1 proteins in eudicots display similar domain organizations (Fig. 2). RING1 proteins in monocots are represented only by Poaceae, displaying few variable domain organizations. The typical RING domain, POU, which is a bipartite DNA binding domain, and Ras Exchanger Motif are also found in ZmRING1a, *Agrobacterium* VirD5 protein, and Spectrin repeats domains display in BdRING1b. Prion domain occurs in OsRING1b. Intriguingly, the POU domain was first identified in plants. Group-II exists in fern and *Physcomitrella patens*, whereas Group-III is present in two algae. However, both of these groups are well conserved in domain organizations.

### PRC1 core component: BMI1

The three BMI1-like proteins, AtBMI1a, AtBMI1b, and AtBMI1c exist in *Arabidopsis* [48]. BMI1 deficiency (*atbmi1a;atbmi1b* double mutant) causes an embryonic-like structure at the vegetative stage and a high number of floral organs in the reproductive stage, a feature similarly found in the *ring1a;ring1b* double mutant [45, 46, 49]. Similar to RING1 proteins, BMI1a/1b functions as PRC1 writers for H2Aub, coordinating with PRC2-mediated H3K27me3 to maintain cell identity [50]. AtBMI1a/1b functions as an E3 ubiquitin ligases, and is involved in drought-response [51]. MIR156A and MIR156C are also target genes of AtBMI1, regulating vegetable to productive development transition [28]. Notably, AtBMI1c acts as an imprinted gene that expresses maternally allele in the endosperm but biallelically in the stamen [49]. BMI1 proteins can be identified in all plants, and in alga *Volvox carteri*, but not in the algae *Ostreococcus lucimarinus* or *Chlamydomonas reinhardtii*; furthermore, BMI1 are divided into two groups, namely BMI1a/1b and BMI1c homologs (Fig. 3). All BMI1s contain highly conserved RING and RAWUL domains except for BsBMI1a, PtBMI1d, and OrBMI1b, OrBMI1b lacks RAWUL domain. The sequence length of BMI1s usually occurs between 350 and 550 aa, but FvBMI1c comprises 974 aa residues with an overlong C-terminus. Dicotyledon contains three copies of BMI1 except for poplar and cotton five copies, and orange two copies. All BMI1a/1b proteins show similar domain organizations, but ThBMI1b harbors another TIM-phosphate-binding motif adjacent to the RING domain; furthermore, BdBMI1d owns a Structural Maintenance of Chromosomes (SMC) proteins Flexible hinge motif, which is responsible for DNA dimerization and as an essential determinant of dynamic SMC–DNA interactions [52]. Highly conserved AtBMI1c and its homologs are only found in Crucifera (Fig. 3).

The RAWUL domain was first identified in the PRC1 RING finger proteins, RING1 and BMI1 families, and is conserved in plant and worm [48]. The RAWUL domain may be involved in epigenetic regulation by binding to PRC1 or other factors. In mammals, RAWUL has been shown to bind to Ph homologs though this phenomenon has not been confirmed to date. Thus, the RAWUL domain may bind to other proteins involved in histone ubiquitination. Sanchez-Pulido et al. suggested that some other proteins demonstrate PRC1 histone ubiquitination functions [48]. *Arabidopsis* HTA10 displays the conserved PKKT consensus sequence [53]. Maize ubiquitinated H2A may be involved in H2A ubiquitination [54]. Grain RAWUL protein Gnp4/LAX2 regulates grain length via the auxin signaling pathway by interfering with OsIAA3-OsARF25 [55]. The RAWUL domain can form a protein-protein interaction module with the PAL domain of AL6 N-terminus, which is an associated factor of PRC1 [36, 48, 56].

The RAWUL domain is not highly conserved between animals and plants. However, a sequence alignment analysis of RING1a/1b homologs shows that the domains are considerably conserved from lower to higher plants, and BMI1a/1b/1c lacks β5. The RING proteins (BrRING1b, ZmRING1b and SmRING) and the BMI1 proteins (AtBMI1c, BsBMI1a, OrBMI1b and VcBMI) do not contain RAWUL domains (Figs. 2 and 3, Additional file 3). RING and RAWUL domain are possibly be special domains for RING1 and BMI1 families.

### PRC1 Core component: LHP1

In *Arabidopsis*, LHP1, an activator and a repressor of transcription, is first identified as a *Drodophila* Heterochromatin-associated Protein 1 (HP1) homolog that binds to H3K27m3 markers established by PRC2 and catalyzes monoubiquitination at lysine 119 of histone H2A [57]. LHP1 may be an analogous role to the fly Pc in a PRC1-like complex [36]. LHP1 contains two typical domains, Chromatin Organization Modifier (CHROMO) domain which is essential for H3K27me3 binding specificity [10], and Chromo Shadow (ChSh) domain [58]. In contrast with its animal counterpart, LHP1 is predominantly located within the euchromatin [59]. The localization and retention of Fern LHP1 are controlled by distinct domains, and its retention at the nucleolus and chromocenters is conferred by the ChSh domain [60]. *P. patens* PpLHP1 interacts with PpCMT through their chromo domains [61]. As a PRC1 reader in plants [27], LHP1 controls multiple developmental pathways related to organ development, cell size, and vegetative to reproductive phase transitions [57, 62].

**Fig. 4:**
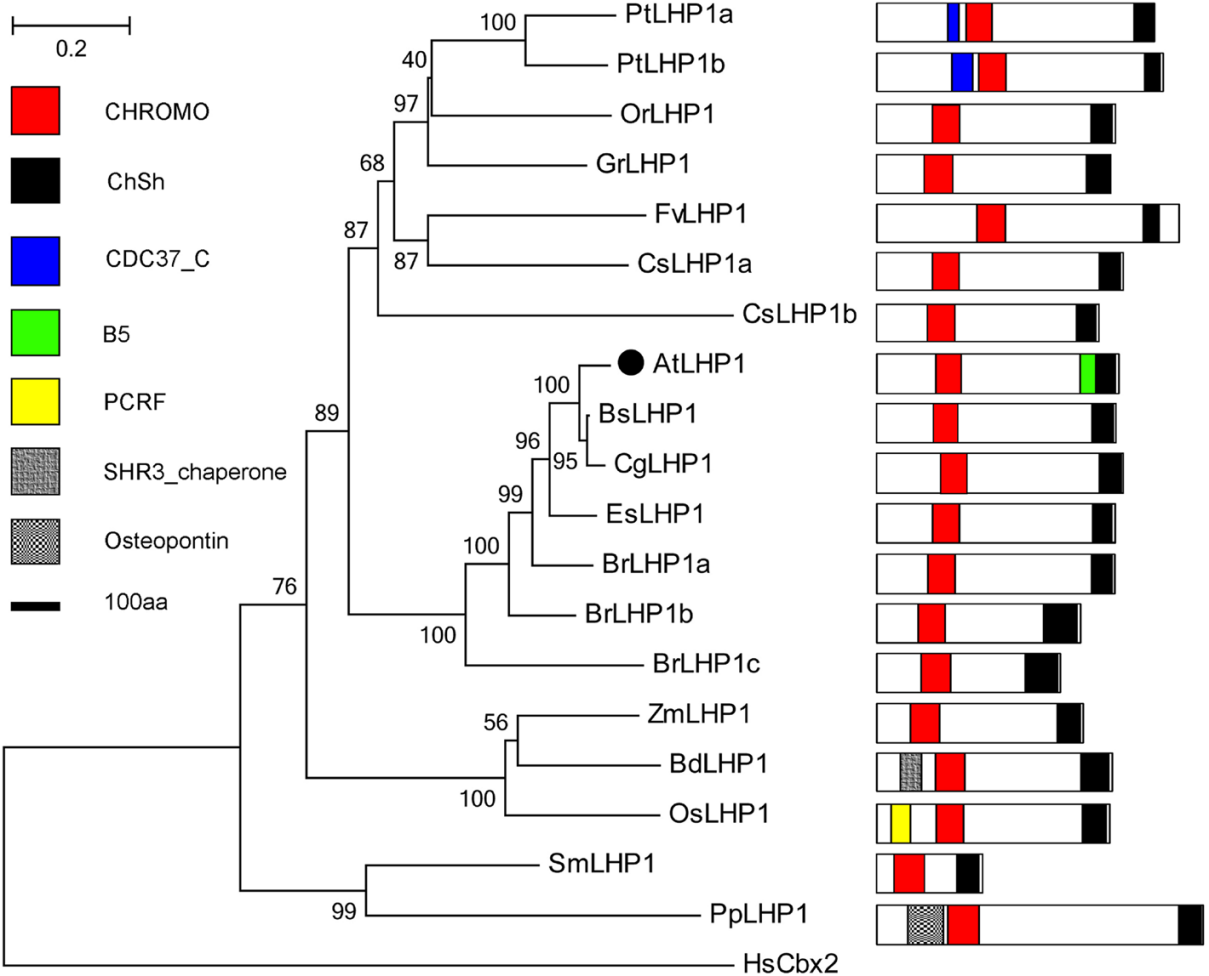
Phylogenetic tree of LHP1 proteins in the Green Lineage. Plant LHP1 homologs exist in higher plant, and not in alga. CHROMO and CHSH domains are the essential features of LHP1 proteins

LHP1 homologs also undergo plant evolution. Aside from distinguished CHROMO and ChSh domains, some LHPs contain other distinct motifs (Fig. 4). For example, poplar LHP1s have an additional CDC37 domain in their N-terminus, and AtLHP1 comprise an additional B5 domain found in phenylalanine-tRNA synthetase β subunits. OsLHP1 consists of another Peptide Chain Release Factors domain linked to the protein family; furthermore, this domain plays an important role in newly synthesized polypeptide chains released from peptidyl-tRNA [63]. BdLHP1 contains another ER membrane protein SH3, which is associated with membrane localized chaperones. PpLHP1 comprises an additional ostepontin domain.

### PRC1 associated factor: EMF1

EMF1 and VRN1 are specifically found in dicotyledon species [27, 40]. Both EMF1 and VRN1 are non-sequence specific DNA-binding proteins that regulate gene expression during flower organ development. Aubert et al. regarded EMF1 as a novel protein involved in the controlling of shoot architecture and flowering in *Arabidopsis*; furthermore, EMF1 loss-of-function mutants cause accelerated embryonic to reproductive development transition [64]. EMF1 and EMF2 participate in the PcG-mediated silencing of flower homeotic genes and are crucial for vegetative development [32, 65]. EMF1, ATX1, and ULT1 can work together to maintain chromatin integrity and prevent precocious seed gene expression after germination [66]. EMF1 is associated with an H3K27me3 reader that is required for H3K27me3 [23, 67]. EMF1, LHP1, and histone H3 lysine-4 demethylase can form an EMF1c complex to play important roles in the regulation of MIR172 and *Flowering Locus T* (*FT*) [28, 68].

**Fig. 5:**
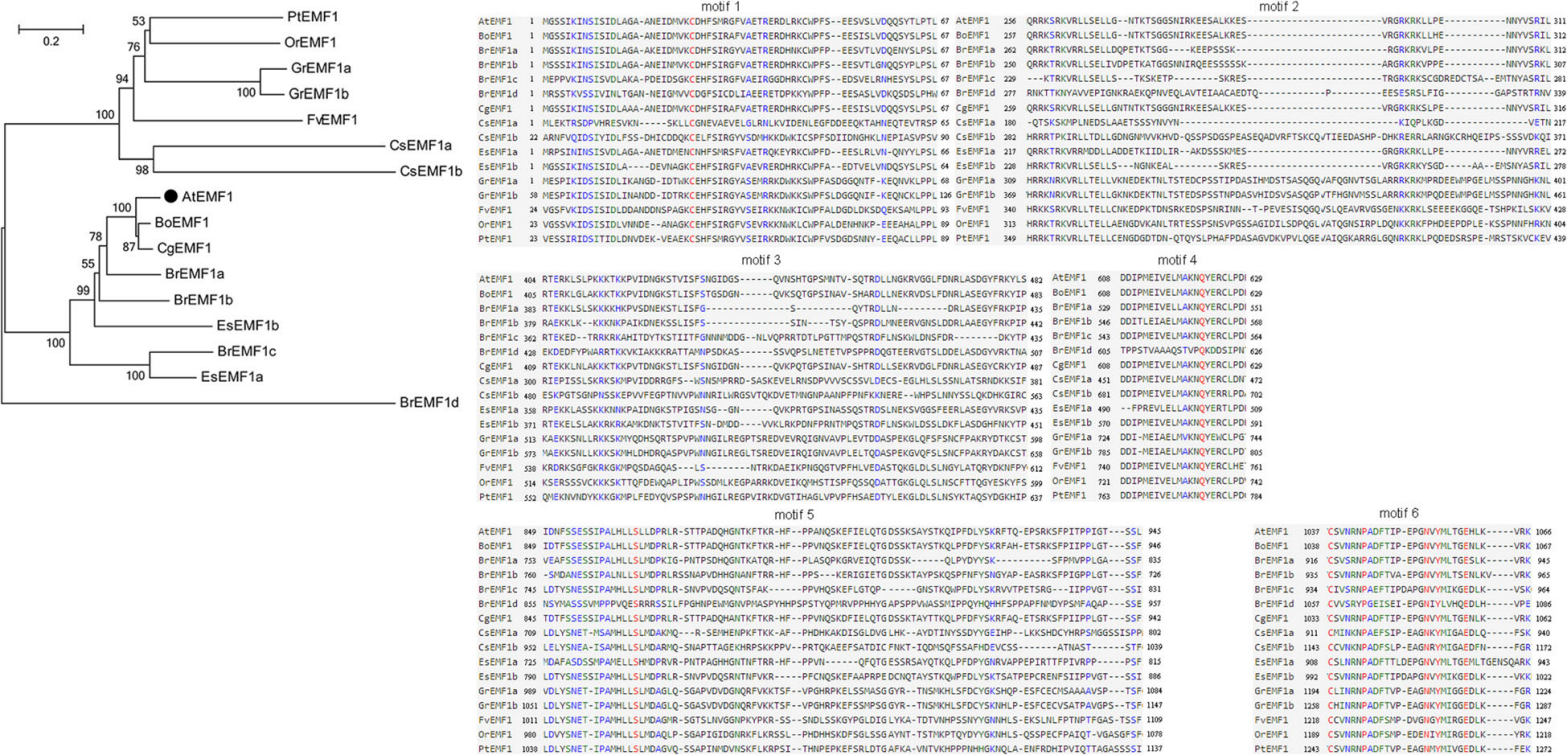
Phylogenetic tree of EMF1 proteins in the Green Lineage. Plant EMF1 homologs only exist in dicotyledons. Six motifs are detected in plant EMF1 proteins

Each species harbors a single EMF1 homologous gene except for cucumber, cotton and *Eutrema* with two, and cabbage with four. Phylogenetic analysis shows that EMF1 are well conserved in dicotyledon, but may lack representative or intact domains in the Pfam and SMART database. Protein sequence alignment suggests that six conserved motifs, especially motif 4, 5, and 6 (Fig. 5, Additional file 4), and functions of which are unknown.

### PRC1 associated factor: VRN1

VRN1 and VAL1/2/3 are plant-specific components of PRC1, and are subclades of the plant specific B3 domain transcription factor family (Additionalfile 5). Similar to EMF1, *Arabidopsis VRN* genes can mediate vernalization and play a major role in vegetative to reproductive phase transition in response to prolonged cold treatment. VRN1 localizes in the nucleus and is sequence-nonspecific in DNA binding, targeting at *FLC,* and *FT2* [69–71]. Loss-of-function mutants show similar phenotypes to other PRC1 mutants [72].

**Fig. 6:**
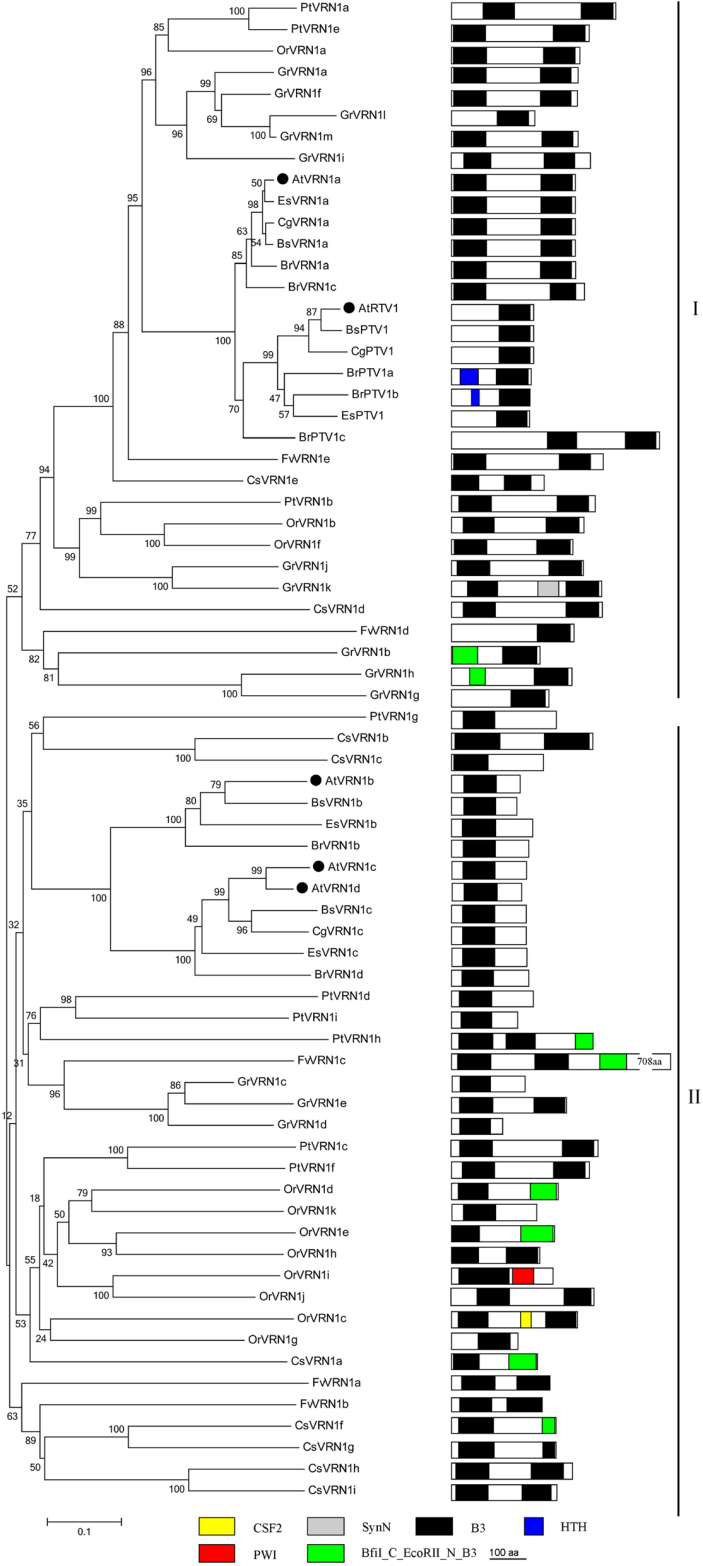
Phylogenetic tree of VRN1 proteins in the Green Lineage. Plant VRN1 homologs only exist in dicotyledons and were subgrouped into the two clades: Group-I AtVRN1a/RTV1 and group-II AtVRN1b/1c/1d. B3 domain is the essential features of plant VRN1 proteins

VRN1 and its homologs are subgrouped into the two clades, AtVRN1a/RTV1 and AtVRN1b/1c/1d. The B3 domain is possibly a special domain of the VRN1 family (71, Fig. 6). AtVRN1, which is named AtVRN1a in this study, is characterized with two B3 domains, is found only in higher plant species and specifically binds to DNA [40]. In the current study, five VRN1 homologs are identified in *Arabidopsis*, and multiple homologs are also found in other dicotyledons by BlastP. The domain organization showed that AtVRN1a and its homologs consist of two B3 domains (Fig. 6). AtRTV1, AtVRN1b/1c/1d and their homologs mainly in group-II show a loss in the second B3 domain, which may be important for its functions [71]. This domain is replaced by the BfiI_C_EcoRII_N_B3 super family, which contains an N-terminal DNA binding domain of type IIE restricted endonuclease EcoRII-like proteins, a C-terminal DNA binding domain of type IIS restricted endonuclease BfiI-like proteins, and plant-specific B3 proteins [73].

### PRC1 associated factor: VAL1/2/3

VAL proteins, which are identified as a transcriptional repressor, are required for global repression of embryonic genes expression [74]. The seedlings of *va l1 val2* double mutant can form embryo-like proliferations in roots and apical meristem, but not in leaves. *Val2/val3* mutants exhibit similar dominant effects in the *val1* homozygous mutant plants [74]. In *val1* mutants, 39% of transcripts in the FUSCA3 regulator are depressed, whereas the core LAFL network transcription factors are not. All putative targeted transcripts of VAL1 act through epigenetic and/or transcriptional repression [75]. Additionally, VAL1 and VAL2 are involved in vernalization via PcG. VAL proteins work together with BMI1 to mediate the monoubiquitylation of H2AK119, and initiate the repression of seed maturation genes [17]. VAL proteins mediate repression through recruiting a histone deacetylase complex to LEC1/AFL genes [76]. VAL1 represses *FLC* transcription by promoting histone deacetylation [77]. VAL1 downregulates AGL15 by H3K27me3 deposition at the upstream sequences of *AGL15* [78].

**Fig. 7:**
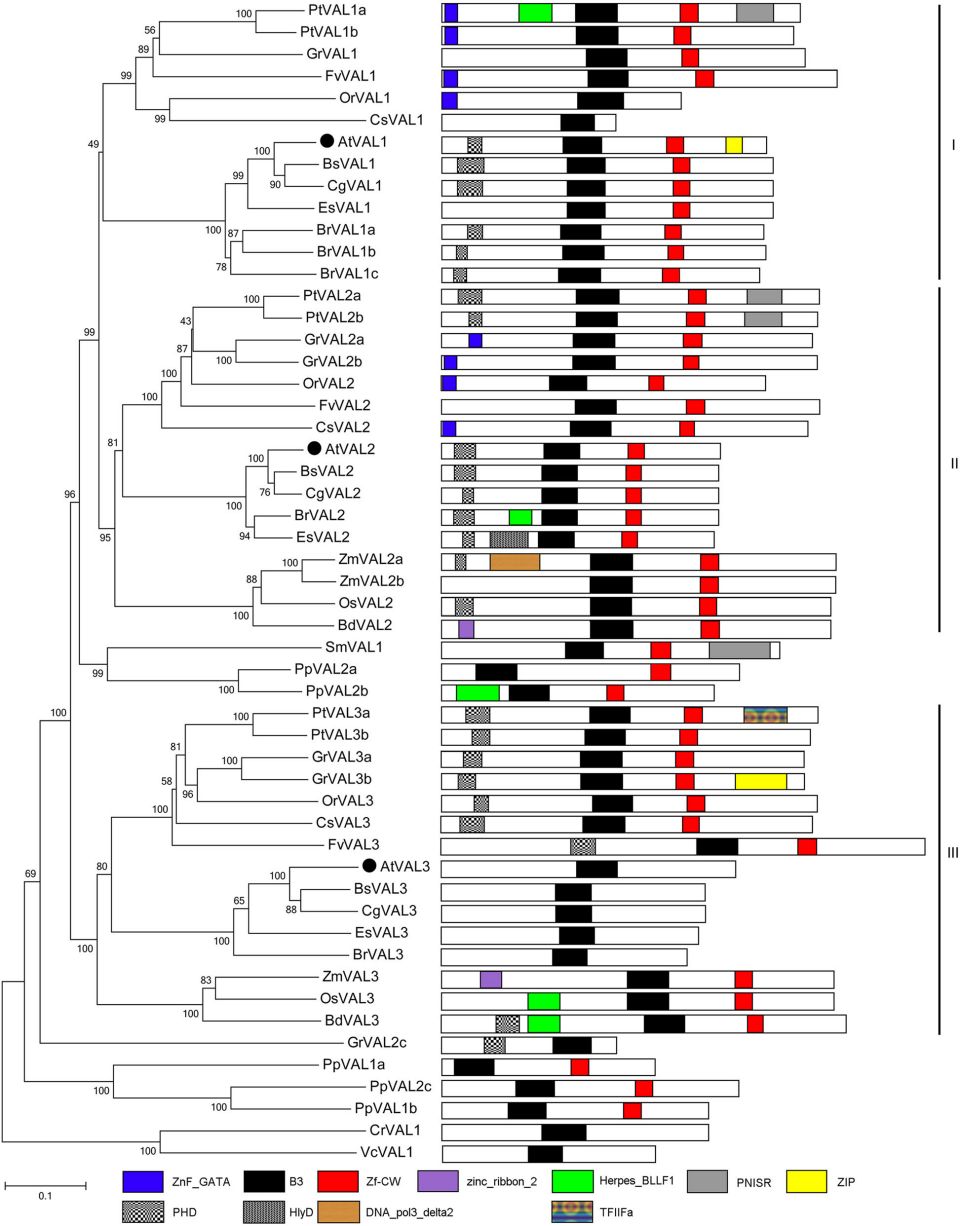
Phylogenetic tree of VAL1/2/3 proteins in the Green Lineage. Plant VAL1/2/3 homologs exist from algae to higher plant, and were subgrouped into the three clades: Group-I VAL1 homologs existing in dicotyledons and group-II VAL2 and Group-III VAL2 homologs in angiosperm. B3 and zf-CW domains are the essential features of plant VAL1 proteins

Except for B3 and zf-CW domains, which are possible special domains for the VAL1/2/3 family, most VAL1/2/3 homologs carry additional Zinc finger motifs, such as PHD and ZnF-GATA, at the 3′-terminal (Fig. 7). The VAL1-B3 domain is necessary to interact with the canonical Sph/RY element within *AGL15* and *FLC* [78–80]. The zf-CW domain is a member of the histone modification reader modules for epigenetic regulation [81]. In the current study, the VRN1 family is just found in dicotyledons (Fig. 6) and its homologs contain one or two B3 domains. By contrast, VAL1/2/3 proteins are found from alga to angiosperm, and only one B3 domain. Furthermore, VAL1/2/3 proteins can be clustered into three groups (Fig. 7). Group-I-containing VAL1 homologs are only found in dicotyledons; Group-II-containing VAL2 homologs and Group-III-containing VAL3 homologs are found in both dicotyledons and monocotyledons. As indicated by our results Alga and fern show only one VAL homologous protein, whereas moss demonstrates five members, and *O. lucimarinus* exhibits none.

### PRC1 associated factor: AL1–7

AL proteins, carrying a conserved PHD domain, were identified as a transcription factor [82]. Arabidopsis Alfin proteins are regarded as H3K4me2/3 readers [36, 38] and function as novel partners of AtRING1 and AtBMI1 [36]. The AL protein is involved in many developmental processes, such as enhancement of MsPRP2 expression in alfalfa roots and contributes to salt tolerance [83]. In *Arabidopsis*, both AL1 and AL5 can bind to the promoter regions of target genes and suppress multiple negative factors to confer abiotic stress tolerance [84, 85]. *Arabidopsis* AL6 is implicated in regulating the expression of root hair elongation-related transcripts on phosphate starvation; furthermore, this process is due to its PHD domain that can bind to H3K4me3, which is an epigenetic regulation strategy for low phosphate availability [86, 87]. However, AtAL7 plays a negative role in salt tolerance [88]. In the current study, the AL and ING families share PHD domain, and the phylogenetic tree shows that they belong to different branches, suggesting their close relation (Additional file 6). The ALs family is the largest PRC1 associated factor family. *Arabidopsis* comprise seven ALs that can be divided into three groups, AtAL1/2, AtAL3/4/5 and AtAL6/7 [84, 88]. In the current study, the AL proteins of seed plants can be divided into three groups, namely, Group-I (AL1/2), Group-II (AL3/4/5), and Group-III (AL6/7). The AL proteins of spore plant are located on the bottom of the phylogenetic tree (Fig. 7). Maize and cotton comprise more AL protein members than others species.

Aside from FvAL5, which comprise 687aa with three Alfin domains and one PHD domain, most plants are extremely conserved in domain organizations, that is one Alfin domain and PHD, and exhibit a sequence length of approximately 230-300aa. (Fig. 7, Additional file 1). One Alfin and PHD domians, the special domain for the AL family, are distributed on the N- or C- terminus of the proteins. The PAL motif, located in the Alfin domain, of AL2 and AL7 proteins can bind to RING1 and BMI1 [35]. PHD-finger proteins are universally found in eukaryotes and act as key players in regulating transcription and chromatin structure [38, 89]. PHD finger is required for H3K4me3/2 binding in the AL and ING families [38].

### PRC1 associated factor: ING1/2

AL proteins exist only in plants whereas ING proteins are widely distributed in yeast, animals and plants. ING was first identified in mammals, and all five ING proteins can bind to H3K4me3/2 through PHD fingers and act as components of histone modifications [38, 90]. However, these proteins have rarely studied in plants. Similar to AL proteins, conserved AtING proteins can recognize H3K4me3/2 mediated by PHD fingers, whereas the biological functions of AtING are unknown [38].

**Fig. 8:**
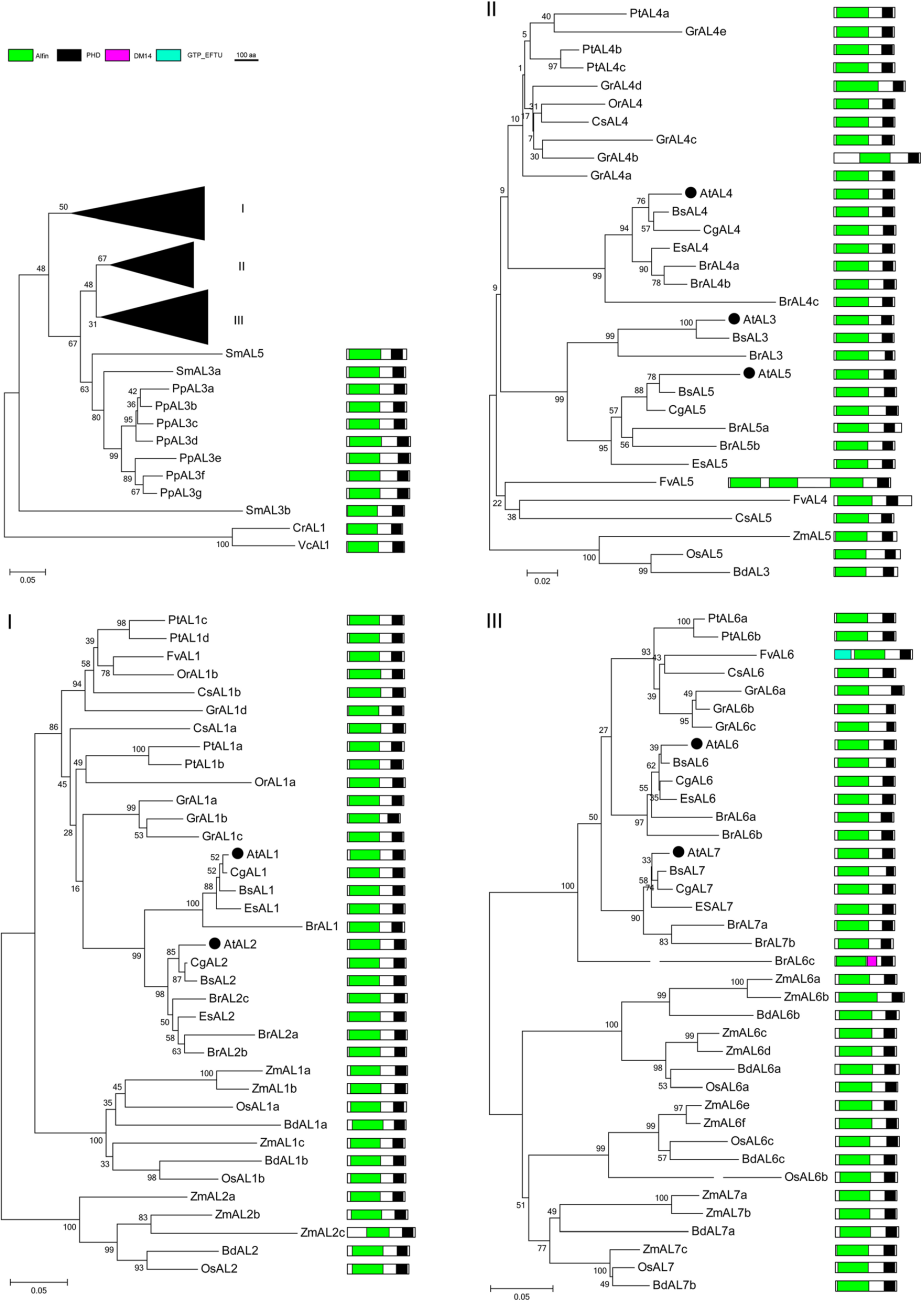
Phylogenetic tree of ING1/2 proteins in the Green Lineage. Plant ING1/2 homologs exist from algae to higher plant, and were subgrouped into the two clades: Group-I ING1 homologs existing from algae to higher plants and group-II ING2 homologs in higher plants. ING and PHD domains are the essential features of plant ING1 proteins

Most plants contain two ING genes (Fig. 8). ING proteins carry an N-terminal ING domain that binds to unmodified H3 tails, and a C-terminal PHD domain that is necessary for binding H3K4me2/3 [38]. In contrast with previous works, we identified VcING1/2, OlING1 and CrING1/2 homologs in green algae. The PHD fingers in VcING2 and CrING2 are replaced by the Tudor domains, which are also implicated in protein-protein interactions. The Tudor domain may bind to the symmetrically dimethylated arginines of arginine-glycine rich sequences and histone H4 dimethylated at Lys20 [91].

### PRC1 associated factor: EBS/SHL

EBS and SHL are BAH-domain-containing proteins [23], which are only found in plant kingdom and widely distributed from lower to higher plants (Fig. 1, [92]). *Arabidopsis* EBS proteins are negative transcriptional regulators, and *ebs* mutations result in early flowering phenotypes [93]. EBS and SHL bind to different floral integrators, EBS regulating *FT* and SHL repressing *SOC1* [94, 95]. EBS and SHL act redundantly in regulation of seed dormancy [96]. EBS/SHL are H3K27me3 readers that can also bind H3K4me3 [31, 97].

**Fig. 9:**
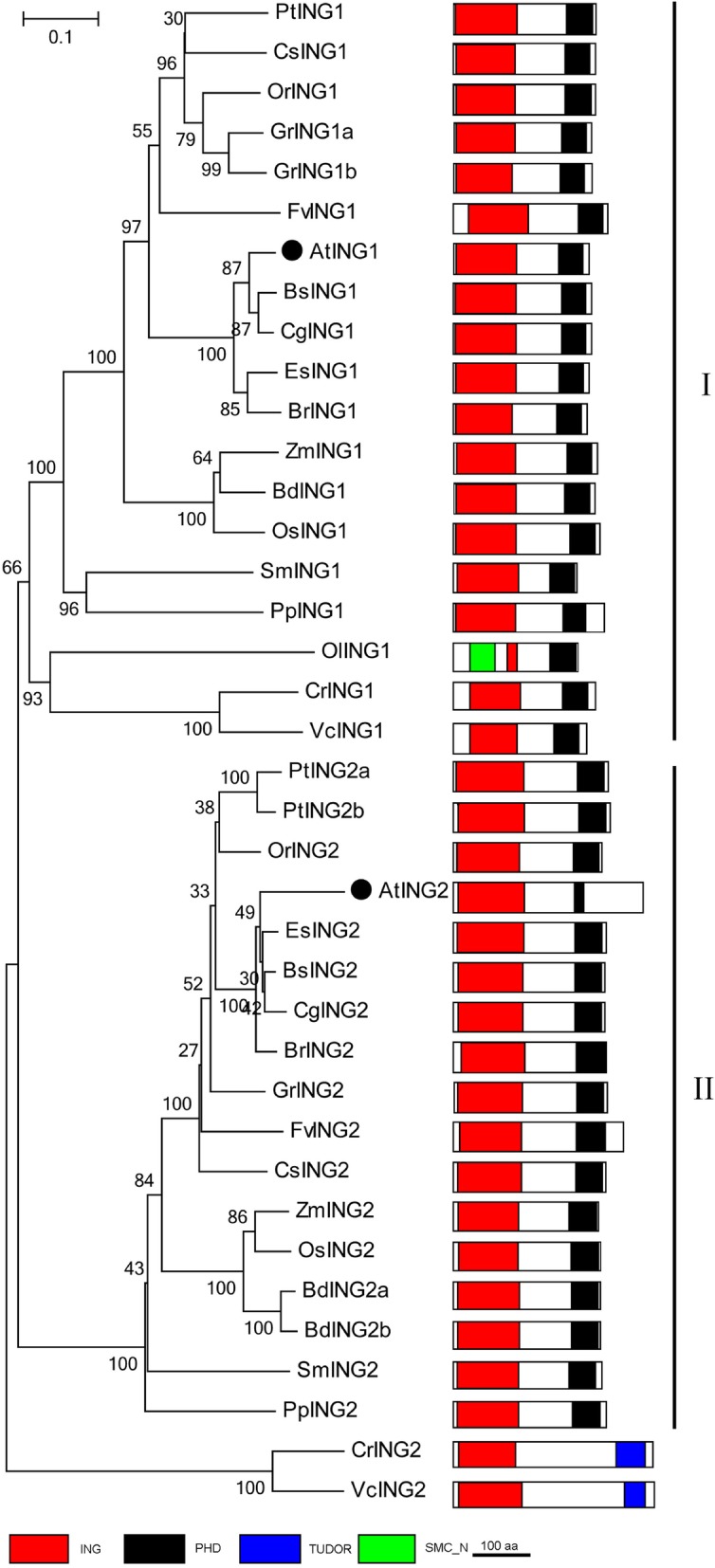
Phylogenetic tree of EBS/SHL proteins in the Green Lineage. Plant EBS/SHL homologs exist from algae to higher plant, and were subgrouped into the two clades: Group-I EBS homologs existing in higher plants and group-II SHL homologs in angiosperm. BAH and PHD domains are the essential features of plant EBS/SHL proteins

EBS/SHL proteins, sub-grouped to two clades (group-I EBS homologs and group-II SHL homologs). Group-I exists in higher plants, but group-II only in angiosperm. Three EBS homologs, PpEBSe/d/f from moss and EBS/SHL homologs from algae are at the bottom of the phylogenetic tree. Most species comprise a single SHL copy, but multiple EBS copies, as reported in poplar, cotton and moss (Figs. 1 and 9). EBS/SHL proteins are highly conserved in length, which ranges from 199 to 336 aa (most are around 220 aa), and in domain organization, an N-terminal BAH domain and a C-terminal PHD domain (Fig. 9). PHD finger is related to H3K4me2/me3, and BAH domain reads H3K27me2/me3 mark. In general, H3K4me3 correlates with transcriptional activation, whereas H3K27me3 correlates with gene silencing in plants and animals. EBS possesses a BAH domain and a PHD domain that reads and affects H3K27me2/me3 and H3K4me/me3 marks, respectively [31, 94]. Furthermore, BAH domain, not the PHD finger, mediates the interaction of SHL or EBS with EMF1 [23]. BAH-H3K27me3 and PHD-H3K4me3 interactions are important for SHL-mediated floral repression [97]. EBS/SHL balances active and repressive chromatin states.

## Discussion

The presence of PRC2 complex in lower plants including algae has been reported [41, 98]. PRC1 is also evolutionarily ancient, and its subunits are found in mammals, plants, insects, and other species, but are lost in several primitive organisms [41]. Sanchez-Pulido et al. proposed that RING1 has originated before the last eukaryotic common ancestor, and BMI1 has duplicated and diverged from RING1 proteins [48]. In plants, RING1 and BMI1 are present in chlorophyte, moss, monilophyte, lycophyte, gymnosperm and angiosperm genomes. However, LHP1 is found only in land plants, but not in chlorophyte. EMF1 and VRN1 have originated from the ancestor of seed plants [40]. Chen et al. inferred that RING1 and BMI1 have originated in mosses [42].

In our dataset, the core components RING1 and BMI1 homologs are detected in algae, except in the most compact eukaryotic *O. lucimarinus*. LHP1 is only found in land plants, but not in algae. These results are in agreement with Berke and Snel [40] (Fig. 1). Consistent with the results of Molitor and Shen [27], but contrary to those of Berke and Snel [40], the associated components VRN1 were exclusively found in eudicots, but not in all seed plants (Fig. 1); Maize VRN1, a MADS-box transcription factor, is homologous to *Arabidopsis* APETALA 1 [99, 100] and not to AtVRN1. Ubiquitous AL proteins are regarded as PRC1 associated factors [36], and ING1/2 exhibits similar functions, thus both these proteins are regarded as PRC1 associated components in this paper (Fig. 1). AL1–7, ING1/2, and EBS/SHL are found in all plant species, from algae to higher plants (Fig. 1, Additional file 1). AL proteins can interact with AtRING1 and AtBMI1 and form AL-PHD-PRC1 complexes to repress the propagation ofH3K4me3. Berke and Snel suggested that the absence of PRC1 subunits is not detrimental for plants, notwithstanding their important roles in other biological processes, such as in LHP1 [40].

Parihar et al. suggested that LHP1 exists in mosses and is conserved in land plants [101]. The sporadic occurrence of subunits in chlorophytes and mosses strongly suggests that the proteins, LHP1, RING1 and BMI1, may demonstrate primitive functional roles in moss [40]. LHP1, as a reader of H3K27me3, may play a role as the fly Pc in a PRC1-like complex [36]. We anticipated that the RING1/BMI1-PRC1 complex is sufficient for alga H2Aub1, but LHP1 is not necessary for alga because of its absence in alga (Fig. 1). In land plants, LHP1 is involved in multiple basic developmental processes including organogenesis and floral transition, which has not been reported in alga. AL6/7 are H3K4me3 readers and PHD domain-containing AL proteins can recruit AtBMI1b and AtRING1ato mediate the transition from H3K4me3 to H3K27me3 [38, 102]. Furthermore, EBS/SHL functions as the H3K4me3 or H3K27me3 reader [23, 31]. It was suggested that ALs or EBS/SHL may make up for the absence of LHP1 in algae. ING induces targeted histone acetylation where the PRC1 complex is not involved [103, 104]. Both AL and ING exist in algae because both gene families may bind to target genes, except PRC1. Furthermore, AL and ING are involved in special functions, experimental evidence for which is however needed.

**Fig. 10:**
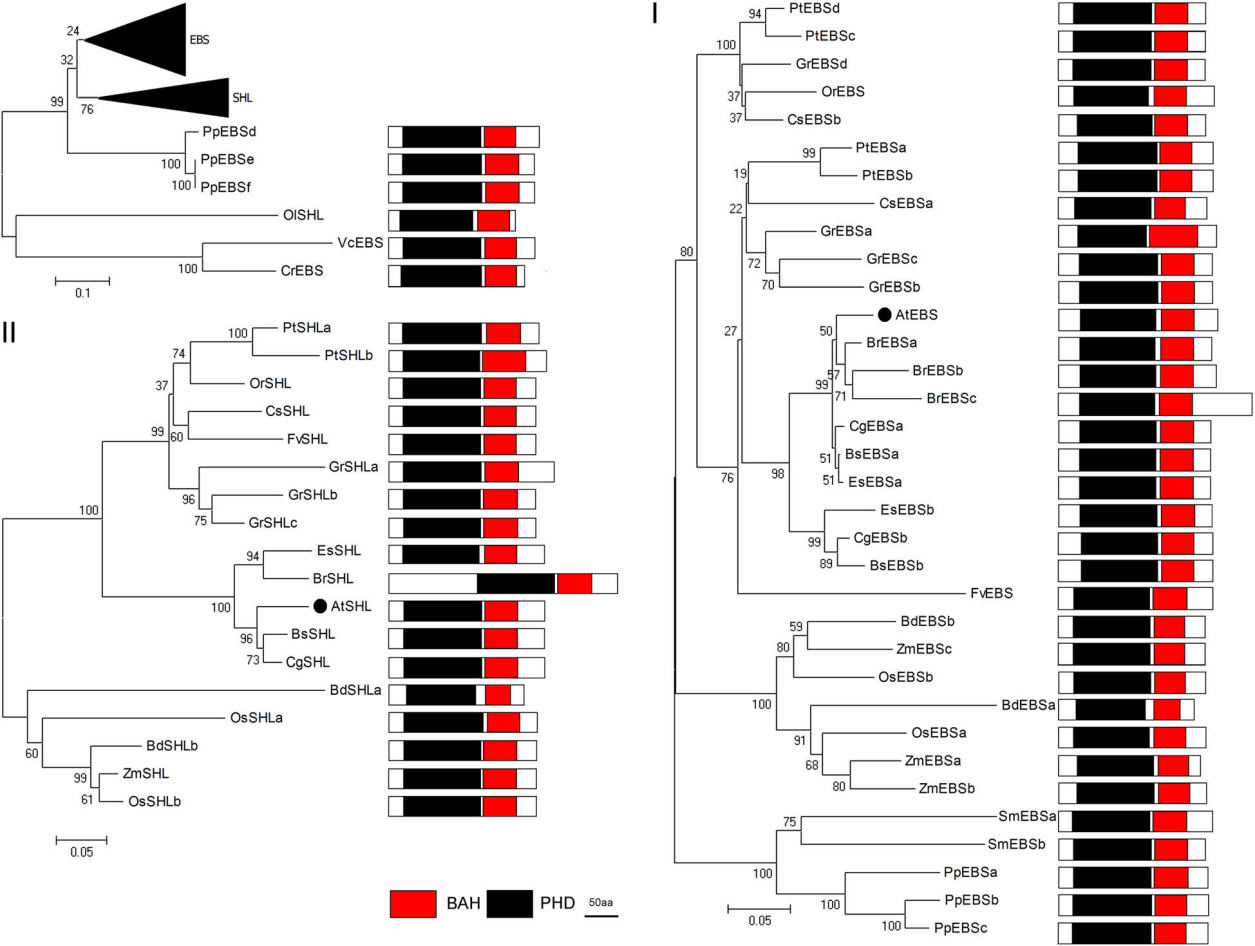
Phylogenetic tree of AL1–7 proteins in the Green Lineage. Plant AL1–7 homologs exist from algae to higher plant, and were subgrouped into the three clades: Group-I AL1/2, Group-II AL3/4/5, and Group-III AL6/7. AL homologs of fern, moss and algae were divided from angiosperm. Alfin and PHD domains are the essential features of plant AL proteins

Interestingly, VAL/AL/ING and RING1/BMI1 co-exist from lower to higher plants (Figs. 1, 7, 8, 10). RING1 and BMI1 are conserved in plants [27]. The number of RING and BMI1 homologous genes is relatively stable, but ALs and EBS/SHL exist greatly in higher plants, especially in polyploids (Fig. 1). VRN, VAL, AL, and ING proteins carrying B3 or PHD domain mainly function in binding histones or genes and are involved in epigenetic regulation related to PRC1 core components. EBS/SHL is a reader of H3K4me3 or H3K27me3. However, the associated factors may or may not be involved in the evolution of core PRC1.

PRC1 proteins are highly conserved in plant domain organization (Figs. 2, 3, 4, 5, 6, 7 and 10). Each subunit exhibits the following special domain organizations: RING1 and BMI1 share RING and RAWUL domains, LHP1 with CHROMO and ChSh domains, VRN1 with one or two B3 domain(s), VAL1/2/3 with B3 and zf-CW domains, AL1–7 with PHD and Alfin domain, and ING1/2 with PHD and ING domain, EBS/SHL with PHD and BAH domain (Additional files 7 and 8). Moreover, six new motifs are discovered in EMF1, and motifs 4, 5 and 6 are well conserved (Fig. 5, Additional file 4).

Gene duplication is one of the primary driving forces in the evolution of genomes and genetic systems, and is a major mechanism for the establishment of new gene functions and the generation of evolutionary novelty. Nonfunctionalization, neofunctionalization and subfunctionalization are the three alternative outcomes of duplicated genes [105]. RING1 has undergone two duplications in the monocot ancestor and then has subsequently undergone several species-specific duplications or losses. However, BMI1 has undergone three duplications in monocots and two duplications in eudicots [40]. In the current study, PRC1 components have multiple homologs in *Arabidopsis*, except for LHP1 and EMF1, which are conservations in numbers. Other PRC1 members often exhibit several homologs in monocot and dicot plants, except algae and Cruciferae. *Brassica rapa*, *Gossypium rainondii*, *Populus trichocarpa*, and *Zea mays* always harbor multiple members of BMI1, VRN1, VAL1/2/3, AL1–7, and EBS/SHL. BMI1a/1b is a result of a Brassicaceae-specific duplication, and BMI1c exists in *Cruciferae,* a member of the second eudicot orthologous group (Fig. 3) [40]. Polycomb proteins encoding genes are duplicated in plant evolution process. The most recent polyploidy event was specific to the Brassicaceae family [106].

*B. rapa* (2n = 38) has undergone whole genome triplication after divergence from *Arabidopsis thaliana* (2n = 10), and many PRC1 genes are duplicated [107, 108]. For example, AtRING1a and AtRING1b exhibit conserved sequences and domain organizations. *atring1a* or *atring1b* mutants show normal phenotypes, but *atring1;atring1b* forms ectopic meristem, and AtRING1a and AtRING1b are of functional redundancy [45]. In the current study, *B. rapa* in Brassicaceae comprises three RING1 homologs, except for BrRING1b lacking the RAWUL domain (Fig. 2); three BMI1 homologs with well conserved domain organizations (Fig. 3); three LHP homologs with CHROMO and ChSh domains but lacking the B3 domain (Fig. 4). The associated factors EMF1, VAL1/2/3, AL1–7, and EBS/SHL family are composed of several members (Figs. 6 and 7). This phenomenon also occurs in cotton, poplar and maize. Several conserved members indicate possible functional redundancy, whereas the observed variation in domain organization suggests the genes may be functionally divergent.

## Conclusions

RING1, BMI1, VAL1/2/3, AL1–7, ING1/2, and EBS/SHL are found from algae to higher plants, However, LHP1 only exists in higher plants, and EMF1 and VRN1 are only found in eudicots. Most plants carry one homologous core component LHP1 and a single associated factor EMF1, but multiple members of RING1, BMI1, VRN1, VAL1/2/3, AL1–7, ING1/2 and EBS/SHL. Cabbage, cotton, poplar, orange, and maize often possess more gene copies than other species. The domain organizations are well conserved, except for individual members of proteins with multi-copies. Six motifs are also uncovered in EMF1. The results will facilitate functional studies on these important epigenetic regulatory genes in plants.

## Methods

### PRC1 subunit identification

The sequences of PRC1 proteins from *A. thaliana* were retrieved from the NCBI. The sequences then were used as queries to search other organisms (http://www.phytozome.net, version 12.0) by the BLASTp tools (http://blast.ncbi.nlm.nih.gov). The sequences were then used as query to Blastp in TAIR (http://www.arabidopsis.org/Blast/index.jsp), and sequences hitting to relevant PcG proteins of *Arabidopsis* were analyzed for their domain organizations (Additional file 1). The sequences were named based on their relationship to *Arabidopsis* homologous genes if they could be confirmed, or based on the location on the chromosome if the relationship could not be clearly determined.

### Protein domain organization and phylogenetic analysis

The protein sequences were collected to analyze domain organization by using NCBI-CD searches (http://ncbi.nlm.nih.gov/Structure/cdd/wrpsb.cgi) in the Pfam and SMART database. The low-complexity filter was turned off, and the Expect Value was set at 10 to detect short domains or regions of less conserved in this analysis. Sequences with special domain organizations were used to construct phylogenetic trees.

Multiple sequence alignments were performed using the ClustalW 2.0 program [109]. The resulting file was subjected to phylogenic analysis using the MEGA7.0 program [110]. The trees construction setting was dependent on full length protein sequences. Phylogenetic tree analysis was set as follows: neighbor joining as tree inference; sites as pairwise deletion included; substitution model consisting of P-distance (for BMI1, VRN1, VAL, AL and ING) or passion (for RING1,LHP1, EMF1, and EBS/SHL); and Bootstrap test of 1000 replicates for internal branch reliability.

## Additional files

Additional file 1: Detailed information of PRC1 components in the green lineage. (XLSX 274 kb)

Additional file 2: Phylogenetic tree of BMI1 and RING1 domain containing proteins in the green lineage. (JPG 2829 kb)

Additional file 3: Sequence alignment of BMI1 and RING1 RAWUL domain in the green lineage. (JPG 2023 kb)

Additional file 4: Sequence alignment of EMF1 proteins in the green lineage. (JPG 5338 kb)

Additional file 5: Phylogenetic tree of VAL1 proteins in the green lineage. (JPG 2607 kb)

Additional file 6: Phylogenetic tree of AL1 and ING1 proteins in the green lineage. (JPG 3065 kb)

Additional file 7: Mark domains of PRC1 components. (XLSX 9 kb)

Additional file 8: Proteins sequence of PRC1 components. (DOCX 151 kb)

## Data Availability

The datasets used and/or analysed during the current study available from the corresponding author on reasonable request.

## Abbreviations

AL1–7: Alfin-like 1–7
B3 domain: B3 DNA binding domain
BMI1: B lymphoma Mo-MLV insertion region 1 homolog
CHROMO: Chromatin Organization Modifier
ChSh: Chromo Shadow
EMF1: Embryonic Flower 1
H2Aub: Monoubiquitination
ING1/2: Inhibitor of growth
LHP1: Like heterochromatin Ppotein 1
Pc: Polycomb
PcG: Polycomb group
Ph: Polyhomeotic
PHD: Plant HomeoDomain
PRC1: Polycomb Repressive Complex 1
PRC1: Polycomb Repressive Complex 2
Psc: Posterior sex combs
RAWUL: Ring-finger And WD40 associated Ubiquitin-Like
RING1: Ring finger protein 1
Sce: Sex combs extra
VAL1/2/3: VP1/ABI3-Like 1/2/3
VRN1: Vernalization 1
