# Supplementary figures and images for: Evolution and conservation of polycomb repressive complex 1 core components and putative associated factors in the green lineage
Source: BMC Genomics. 2019 Jun 28;20:533. doi: 10.1186/s12864-019-5905-9 (PMC6599366; doi:10.1186/s12864-019-5905-9)

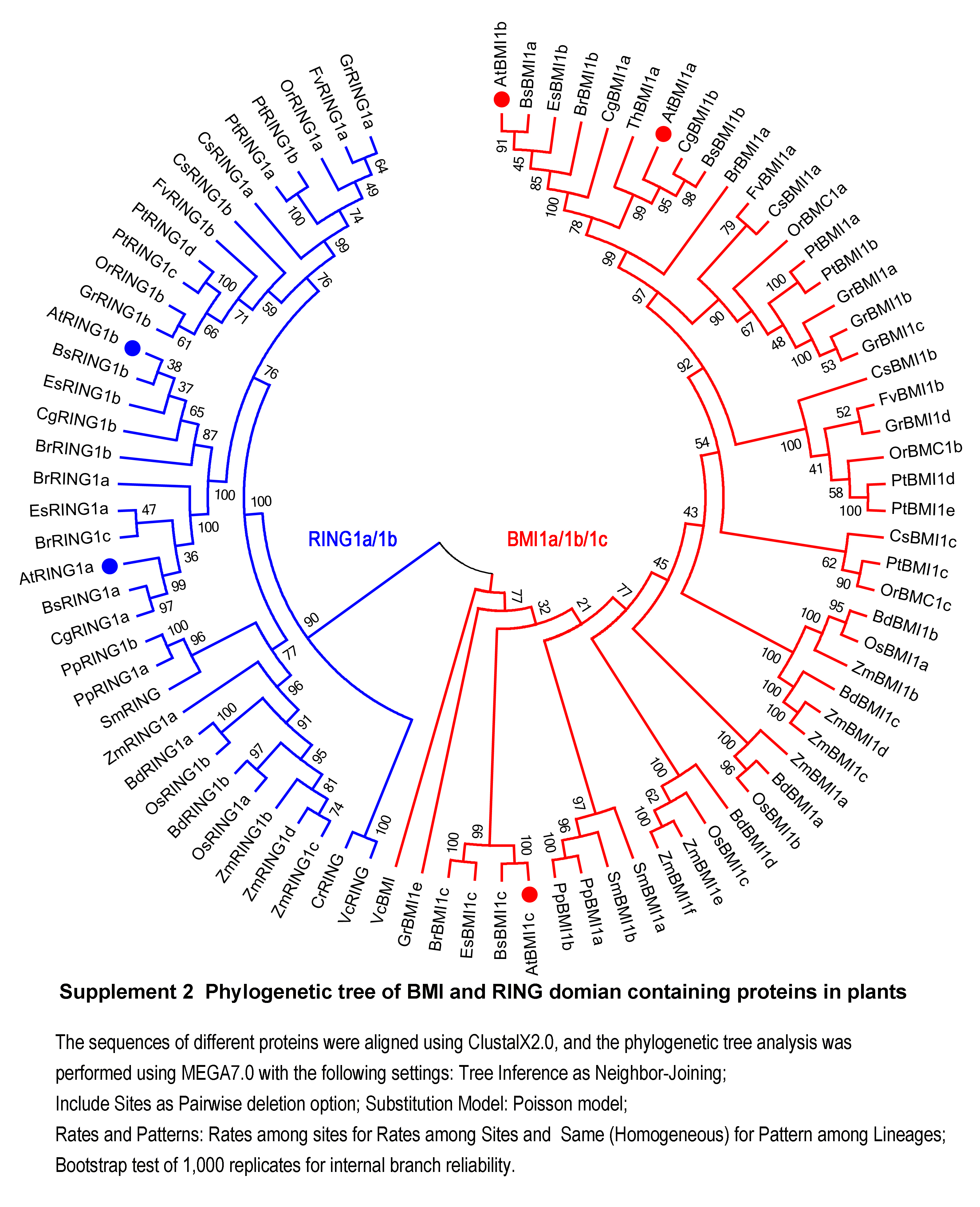

Supplement: Supplementary file 2 — Phylogenetic tree of BMI1 and RING1 domain containing proteins in the green lineage. (JPG 2829 kb) [file 12864_2019_5905_MOESM2_ESM.jpg]

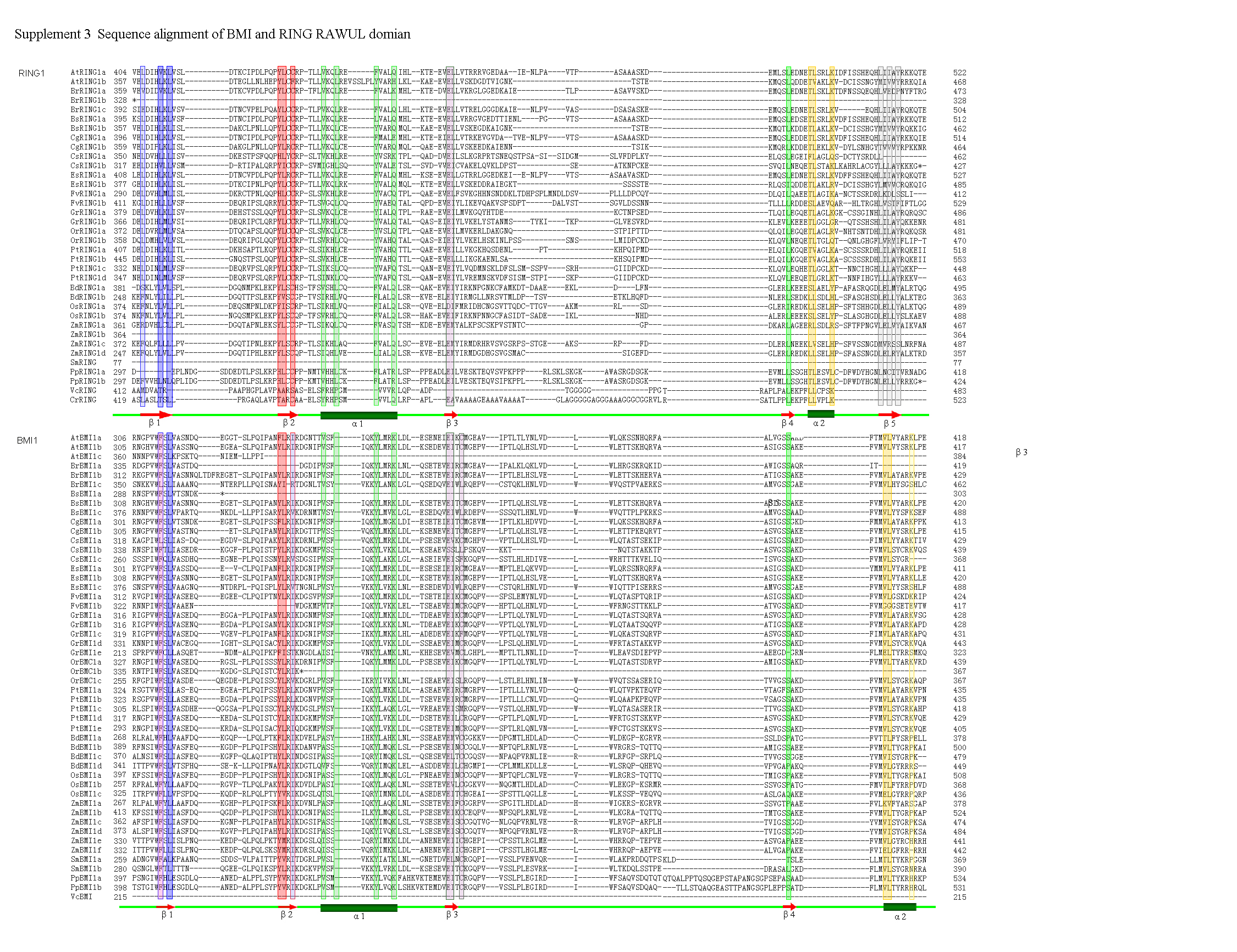

Supplement: Supplementary file 3 — Sequence alignment of BMI1 and RING1 RAWUL domain in the green lineage. (JPG 2023 kb) [file 12864_2019_5905_MOESM3_ESM.jpg]

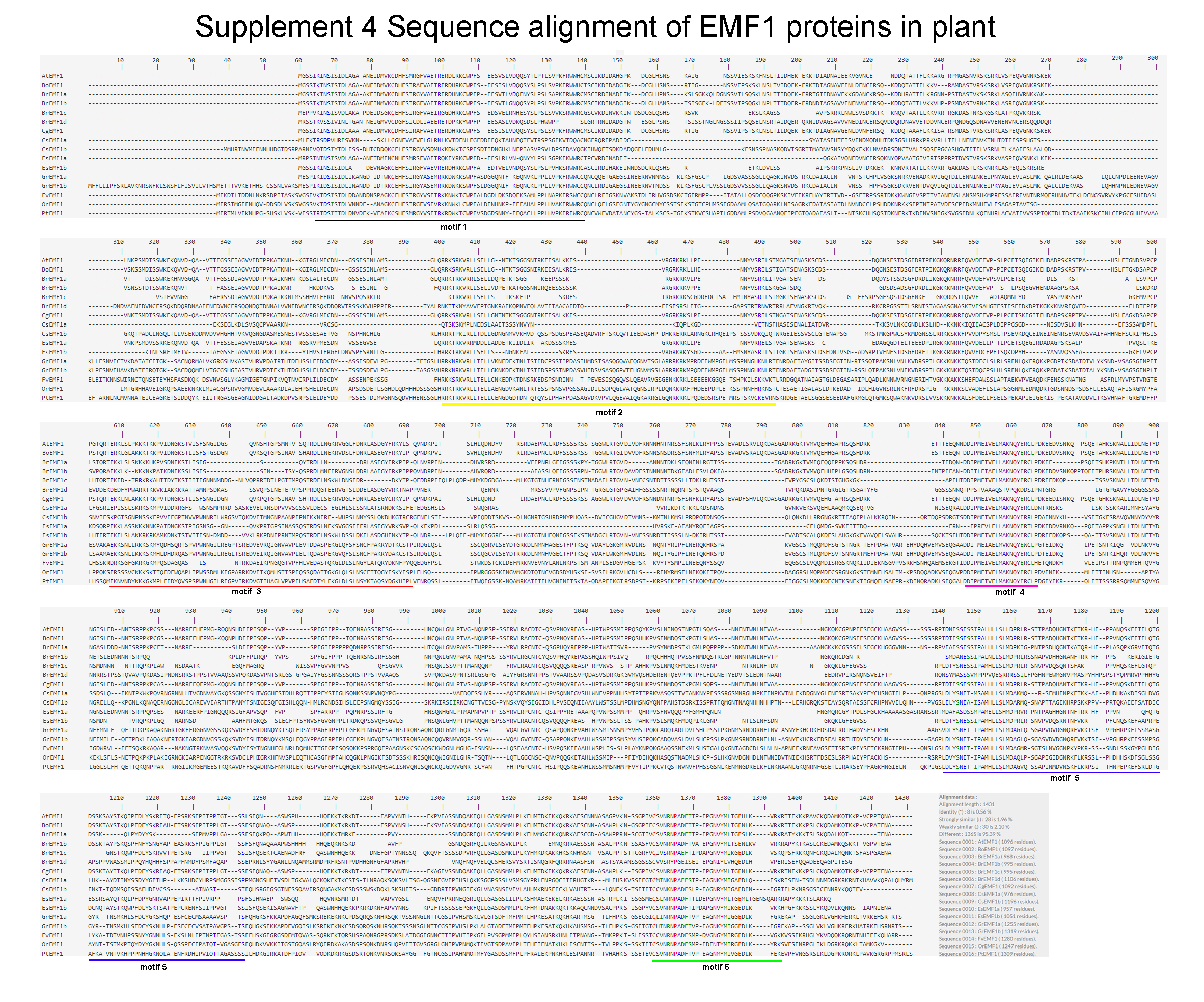

Supplement: Supplementary file 4 — Sequence alignment of EMF1 proteins in the green lineage. (JPG 5338 kb) [file 12864_2019_5905_MOESM4_ESM.jpg]

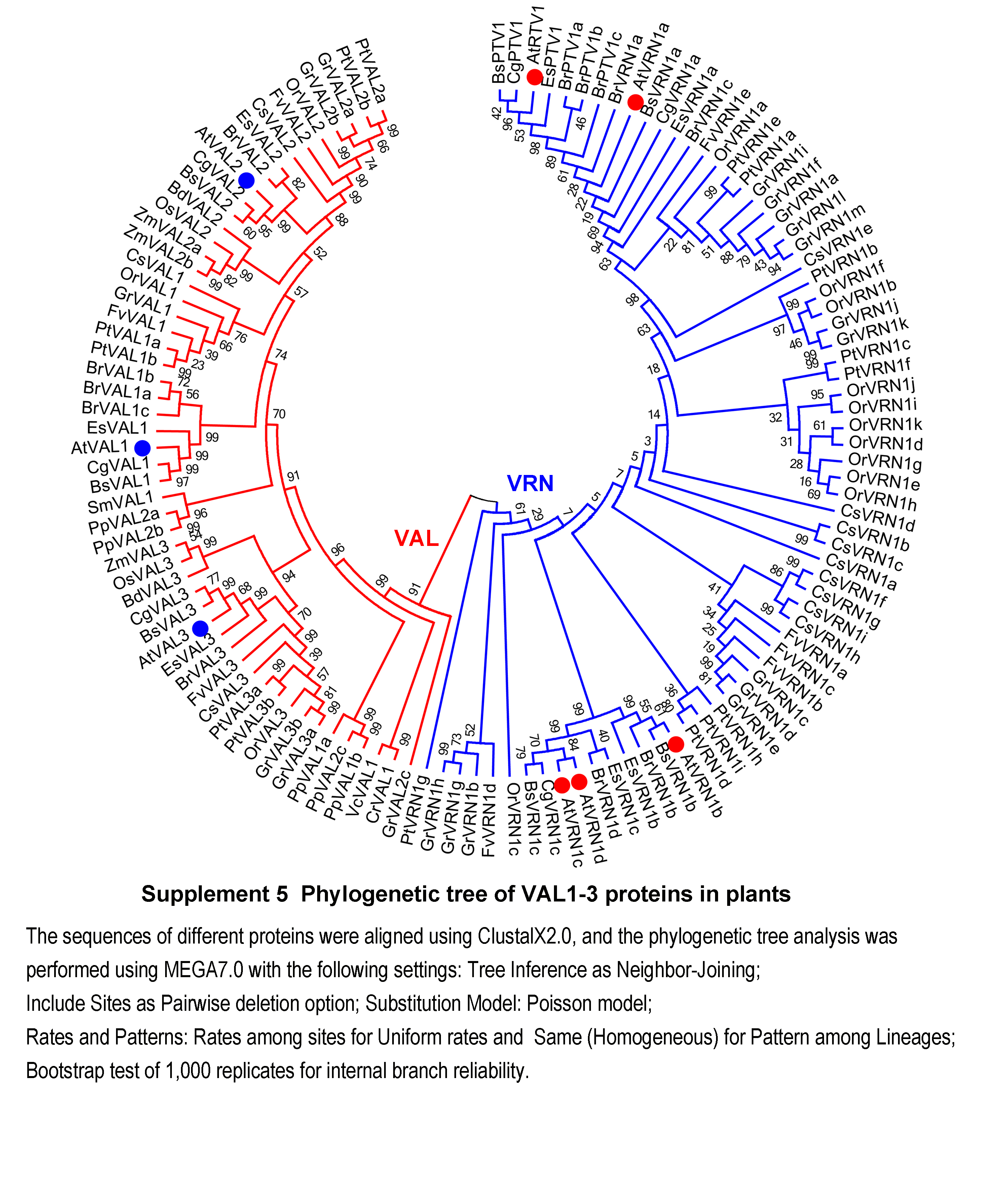

Supplement: Supplementary file 5 — Phylogenetic tree of VAL1 proteins in the green lineage. (JPG 2607 kb) [file 12864_2019_5905_MOESM5_ESM.jpg]

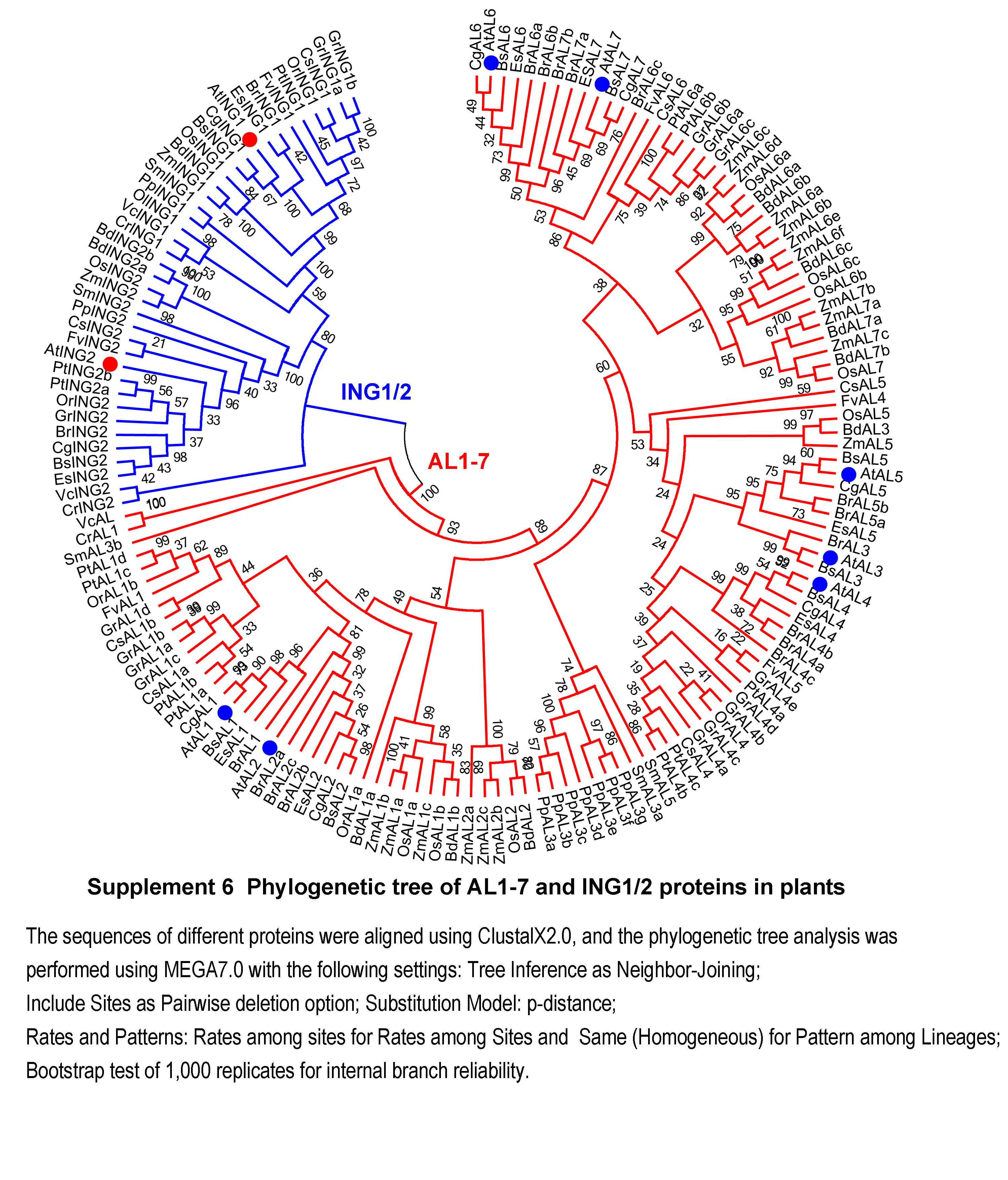

Supplement: Supplementary file 6 — Phylogenetic tree of AL1 and ING1 proteins in the green lineage. (JPG 3065 kb) [file 12864_2019_5905_MOESM6_ESM.jpg]
