# Supplementary material for: Evolution and conservation of polycomb repressive complex 1 core components and putative associated factors in the green lineage
Source: BMC Genomics. 2019 Jun 28;20:533. doi: 10.1186/s12864-019-5905-9 (PMC6599366; doi:10.1186/s12864-019-5905-9)
Supplement: Supplementary file 8 — Proteins sequence of PRC1 components. (DOCX 151 kb) [file 12864_2019_5905_MOESM8_ESM.docx]

**Proteins sequence of PRC1 core components and associated factors**

**Core component: RING1a/1b**

>AtRING1a AT5G44280 *Arabidopsis thaliana*

msvknnsfssaeipdvadqprdrfnpeatqdlqekdetkeekegdeevkhdeaeedqevvkpndaeedddgddaeedeeeeveaeedeeaeeeeeeeeeeeeeeedskerspssisgdqsefmeidlgeirkdvqcpiclgiikktrtvm eclhrfcrec idksmrlgnn ecpacrkhca srrslrddpk fdaliaalft nidsyeeeel afhedemarnkqiqasiaqisqrqsealvkrrslgkeaavlmrspriasgsrrrrnsrnmeqqnaseahedddnddnnnrgrdkdsssdergtevrqkkrrkrstsrstqhpsssganknngncadndtevyrdtkgispglvwnpeilawgrggtrsntrhgnntsggssksvrnarvnklveylrssvdgssveldihvklvsldtkcipdlpqpylccrptllvkqlrefvalqihlkteevellvtrrrvgedaaienlpavtpasaaaskdemlslednetlsrlkidfissheqhliiayrkkq te

>AtRING1b AT1G03770 *Arabidopsis thaliana*

MPSLKSFSAAEEEDDQLGRNSEAERFNPEAVEKEEDPDKMDEKDESGDEEDDVKRDQVEAEDEEALGEEEVSDSKERSQSSSAGELSESEYMVVDLADICKDVQCSICLGIIRKTRTVMECLHRFCRECIDKSMRLGNNECPTCRKHCASRRSLRDDPNFDALIAALFKNIDKFEEEELNFRQDDEARNKQIQASIAQVSQRQSKALVKRKSVGKGTAILSRSRRSGGGSRRRRNCRNIEQDTSEANDDDDQNKRGKDSSSDEPCERQRKKRSATQPSSSNANNNDNCAGNGTEQTHQRDSRVISPVLVWNSELIAWGRGGTRSNTRQGNNNQGAISKRNARLKRLVEYLGSLEGNSVELDIHLKLVSLDTEGLLNLHEPYLCFRPTLLVKQLREVSSLPLYVARHLKLKAEEVELLVSKDGDTVIGNKTSTEKMQSLQDDETVAKLKVDCISSNGYMIV VYRRKQIA

>BsRING1a Bostr.3148s0029.1 *Boechera stricta*

MPVKKSFSSAEIPDEADQRSDRFNPEAEQDLQEKGENKDEEEVKHDEAEEEEEEVRPNEAEEEEEEGKEDDDDDAEKEVEEEQKEEEDEQEEEEEEEEEEEEEEEEEEEEEEEDEEEESKERSPSSISGDQSQSEFMEIDLGEIRKDVQCPICLGIIKKTRTVMECLHRFCRECIDKSMRLGNNECPACRKHCASRRSLRDDPKFDSLIAALFTNIDSYEEEELAFHEDEKARNKQIQASIAQISQRQCEALVKRRSFGKEAAVLMRSPRIASGSRRRRNCRNMEQQYASEAHDDDNNDGNNNNGGGKDSSSDERGTEVRQKKRRKRSTSRSTQHPSSSGANNNNGNCADNDTEVYRDSKGISPGLVWNPEILAWGRGGTRSNTRHGNNTSGGSIKSLDIHLKLVSFDTNCIPDLPQPYLCCRPTLLVKQLRELVALQMHLKTEEVELLVRRGVGEDTTIENLPGVTSASAAASKDEMQSLEDSETLSRLKVDFISSHEQHLIIAYRKKQTE

>BsRING1b Bostr.26675s0385.1 *Boechera stricta*

MPALKNFSAADEEYDQLGRNSEAERLNPEADEKEEDPDEMDDKDESGDVEDDVKRDEVEAEDVEALQEDDSKERSQCSSGGEQSESEFMDIELADIRRDVQCSICLGIIKKTRTVMECLHRFCRECIDKSMRLGNNECPTCRKHCASRRSLRDDPNFDAFIATLFKNIDKFEEEELAFRQDDEARNKQIQASIAQVSQRQSAVLAKRSFGKDTAVLPRSRRSGSGSRRRRNCRNMEQDTSEAHDDDDQNNRGKDSYSDAPCAEILQRKRKKRSTTHPSSSAANNSDNCAGNGTEQAHHRESRGISPGLVWNPEILAWGRGGTRSNTRQGNNNQGASSKRNARLNRLVEYLGSLEGNSVELDIHLKLISLDAKCLPNLLQPYLCCRPTLLVKQLCEYVARQMQLKAEEVELLVSKEGDKAIGNKTSTEKMQTLKDDETLAKLKVDCISSHGYMIVVYRQKKIG

>BrRING1a Bra033732 *Brassica rapa*

MPVKKQGDGGGDQQQQQLDRFDPEAEENNQPEDLQHSTLEKKDDEQEQEEVKRDEAEKEEEEEEGDEEPEEDSKEKSPSADKSEFMEIDLGEIRKDVQCPICLGIIKKTRTVMECLHRFCRECIDKSMRLGNNECPACRKHCASRRSLRDDPKFDALIAALFTNIDTYEEEEFAFHEDDKARNKQIQASIAEISQRQSEALVKRKSFGKEAAVLMRSQRSGSGSRRRRNSRNTEQNADEAHEDDDNNEDHNNNNGGGGRDSSSDERGPEVRVRKRRKRSANNNNGNCGDKDTEVYRDSSKGISPGLVWNPEMLAWGRSATRSNPRHENNTTGGSSKSVRNARVNKLVEYLKRSSVDGKSVEVDIDVKLVSLDTKCVPDLPQPYLCCRPTFLVKQLREFVALKMHLKTEDVELLVKRGLGGEDKAIETLPASAVVSKDEMQSLEDNETLSKLKTDFNSSQEQHLVEDPNYFTRGATSACSSKSYKEKCVSSSSTQNKHYGPSPNVMTLVDLEDEMKEDLMANDLE

>BrRING1b Bra015262 *Brassica rapa*

MPALNNFSAAEKKDDQLGRTTQAEEKEEDPDHMDVMEEEGSKERSSSSTSEEQSESEFIEGIDLEDIRKYVQCPICLGIIRKTRTFMECLHRFCQECIDKSMRFGNHECPACRKHVPSRRSLRPDPKFDAFIAAIFGNVDSNEEQDLAFDEDELARNKQFQATIAQVSQRQSEALVKSSGKDAGVLPRSQPSGSGSRRRRRNSRNMVHDTSQAAHDDDGDNNRVNASSSAKILPRKRNRRSATRSTAHPSSSSCPSNNDNNCANNVTEEAHHRDSRGIAHGFAWGKGGRRSNARQANNNQGASSSKSVRNARLNRLVDYLSTLESNSV

>BrRING1c Bra027538 *Brassica rapa*

MSVEKSLKAAEIPDGEGGDKDQRGDRFGPEPEEKQEEDLQERKDEEGGDDEEEVKRDEAEEEEVVEEDEEDADEEEEEEKDEEEEEEEEDSKEKSPSSASGVNSEYGSFSTLCIQIFVEIDLGEIRKDVQCPICLGIIKKTRTVMECLHRFCRECIDKSMRLGNKECPACRKRCASRRSLRDDPTFDALIAALFSNIDTYEEEEFAFHEDDKARNKQIQASIAEVSQRQSEALVKRKSFGKEASVSTRPQRRRRRNCRNMEQNTVEEANEDDNNDDNNGKDSSSEERGAEVRLRKRRKRSTSRSTLNPSSSGANNNNGNCAENDADMNLRDNNSKGISPGLVWNPEILAWGRGGTRSHTRHGNNATGGSSKSVRNARVNRLVECLRSSVDGNSIEDIHLKLVSVDTNCVPELPQAYLCCRPTLPVKQLREFVALQLHLKTEEVELLVTRELGGGDKAIENLPVVASDSASASKEEMQSLEDNETLSRLKVEQHLIIAYRQKQTE

>CgRING1a Cagra.9908s0004.1 *Capsella grandiflora*

MPVENNSFSSAEIPDESDQRSDRFNPEVEQDLQEKGEGKEEEEVKPEEEVKPEEEEDDDDEEDAEKEVEEEEEEEEEEEEEEEEEEEEEEEDEEDEEEESKERRSPSSMSADQPEFMEIGLGEIRKDVQCPICLGIIKKTRTVMECLHRFCRECIDKSMRLGNNECPACRKHCASRRSLRDDPKFDSLIAALFTNIDSYEEEELAFHEDEKARNKQIQASIAQISQRQSEALVKRRSFGKEAAVLMRSPRIASGSRRRRNCRNMEQASEAHDDDNNDDTTNNGGGKDSSSDERGTEVRQKKRRKRSTSRSSQHPSSSGVNNNNGNCADNDTEVYRDSKGISSGLVWNPEILAWGRGGTRSNTRHGNNTSGGSIKIARNARVNKLVEHLRSSIDGSTVELDIHLKLVSLDTNCIPDLPQPYLCCRPTLLVKQLREFMALEMHLKTEEIELLVTRKEVGVDATVENLPVVTSASAAASKDEMQSLEDDETLSRLKVDFISSHEQHLIIAYRKKQIE

>CgRING1b Cagra.0897s0008.1 *Capsella grandiflora*

MPALKNLSAADEEYDQLGRNSEADRFNPEAVETEGDPEKMDDNKEESGDEADDVKRDQVEAEDEEALEEDSKERSQPSSGPGGEQSDSEYIDVDLADIRREVQCPICLGIIKKTRTVMECLHRFCQECIDKSMRLGNNECPTCRKHCASRRSLREDKLFDAFIATLFPNIDKFEEEELAFHQDDKARNKQIQASIARVSKQQSSAFSKKKSSGKDAAVLSRTQRSGSSSRRRRSCRSMEQDTSEPQDDDDQNNRVKDSSSDEPCALQRKRRKRSTTHPSSSAANNNDKCAGNGTEQAHHKDIRGISPGLVSNAEILAWGRGGTRSNTRQGNNNQGANSKRNARLNRLVEYLGSLEEGNKVELDIPLKLISLDAKGLPNLLQPYLRCRPTLLVKQLCEYVARQMQLQAEEVELLVSKEEDKAIENNTSIKKMQRLKDDETLEKLKVDYLSNHGYTVVVYRPKKNRIEEVGASDPNNVHE

>OrRING1a orange1.1g011622m *Citrus sinensis*

MPAQKRSLQESHHANNGEQLQGEEQQRQDSFQQSHAKQSRHERQQLELQQNEQQQLEEDQKDDEEVEGEGDEEDEENEDDSEGPSSDTSGEKPEFVFVELPEIRKDVQCPICLGIIKKTRTVMECLHRFCRECIDKSMRLGNNECPACRTHCASRRSLRDDPNYDALISALYPDIDKYEEEELAFHEEERTRNKQIQASIAKIFQRQSEALVKRRTPGKDTASPFMTRPQRNPRTAHLRRRRNSRGTEHQGSEDNEDENDDNGGKDSSSNDERSAEVRPRRRKRRSGIRSSQPSSSAANSDGGCIENDLEVGRESRGISPGLVWNPEMLAWGRGGTRSNTRHGSASGCNSKSSRSTRLNKLVEYLRSLQENNDELDVRLMLVSADTQCAPSLQQPYLCCQPSLSVKQLCEYVSLQTPLQAEEVEILMVKERLDAKGNQSTPIPTTDQLQILEGQETLAGLRVNHTSNTDHLILAYRQKQSR

>OrRING1b orange1.1g010664m *Citrus sinensis*

MPAQKRSYENATTNNNNNNHIIITPSEVVEAVLDDDGDDTLQRDHSNNLEEQQPPQDSTPAAAADVDGNESDRSRSSGDGEKDEFVIVKLSDIRKEVQCPICLGIIRKTRTVMECLHRFCRECIDKAMRLGNNECPACRTHCASRRSLRDDLNYDALIAALYPDIDKYEEEELAFQDEEAARNKQIQASIAQTFQRQTEALGRKRTPKSTSALRRSHGRYRDTPLRGRRNYRMTELQGSDENDDANGDAGKDSSSADERSTEVRPKRRKRWYGARFSQSSSAAAGTDGGGDENDSEVHRESMGASVGLIGPSERLAWGKGGIRSHTRHGSVSGSNGKNARNNRLSKLVDYLQSLEEKDDQLDMHLVLVSLDEQRIPGLQQPYLCCRPTLSVRHLCQYVAHQTALQASEIEIYLVKELHSKINLPSSSNSLMIDPCKDKLQVLNEQETLTGLQTQNLGHGFLVRYIFLIPTSIGRFMHTLQLTTKHGKQHSFLIHSCSLIHTLNI

>CsRING1a Cucsa.384920.1 *Cucumis sativus*

MPAHKRSWSDTVHGEDPRDQQFSHQDAKQSRTERETKTDIEEEEEEEEQREEQLKEGQRVDEGEEEKQRQQLEEATGDRVKKLATLLTLTGIIKKTRTVMECLHRFCRECIDKSMRLGNNECPACRTHCASRRSLRDDPNYDALIAALYPDIDKYEEEELTFHEEERNRNKQIQESIAQIFQRQSEALSKKRILGKDTAGVILTRSRRNHRNVHLRRQNGRGDEVSGYEDNDDDDDNNEGKDSSSADERFTEVRQRRKKRHPTVRSSQPSSSIANIDSGDGCAESDLDMSRENRTVSPGLVLNTEMLGWGRGGVRSNSRHGSAGGSGNKSSRSSRLMKLAKYLRGLEENNNELDVHLLLISVDKESTPSFQQPHLYCRPSLTVKHLREYVSRKTPLQADDVEILSLKGRPRTSNEQSTPSASISIDGMSLVFDPLKYELQSLEGEIFLAGLQSDCTYSRDLL

>CsRING1b Cucsa.046340.1 *Cucumis sativus*

MPARKRAYDSLDVDLLPPRLHNTFNNERSQDEQSQESDRSSSSSQGDEDEFIAVKLSDIRKEVQCPICLGIIKKTRTVMECLHRFCRECIDKSMRLGNKECPACRTHCASRRSLRDDPNYDTLIAVLYPDIEKYEEEELAFQEEEKARNKQIQTSIAQTLQRQTESLGRKRSKPSRRLSSRGPKSFQNHIESLCFDENEDENDYDVSKNSSSADERMDTRPKRPRRCGPVRFSQSSSATGADGADGGGIEHEYEVNKEKVGASLGLVGSSEKLSWGKGGIRSHTRYGGTNGGAGKISRNNRIAKLSDYVRNSENVSEEELDIHVLLVSMDRTIPALQRPYICCRPSVMIGHLSQYVALETSLSVDVVEICVAKELQVKLDPSTSEATKNPCKESVQILNEQETLSTAKLKAHRLACGYLLLAYKKKG

>EsRING1a Thhalv10003181m *Eutrema salsugineum*

MPVKTSFSAAEIPDEEVEDKDQPRDRFNPEAEENEEEDLEEKVEKKDEEEEGDDAEVKRDEADEEEEAEEADEDEEEDEADGEEEEDDVEDEDAEEEQEEEEEEEEDSKEKSSSPNSGEQSEFMDIDLGEIRKDVQCPICLGIIKKTRTVMECLHRFCRECIDKSMRLGNNECPACRKHCASRRSLRDDPKFDALIAALFTNIDSYEEEELAFHEDDKARNKQIQASIAEISQRQSEALVKRRSFGKEAAVLMRSQRSASGSRRRRNCRNMEQNALETHEDDNNDDNNNNGGGGGKDSSSDERGAEVRQRKRRKRSTIRSTQHPSSSGANNNNNNGNCADNDTEVYRDSKGISPGLEWNPEILAWGRGGTRSNTRHGNNTPGGSVKSLRNARVNKLVEYLRSNVDGNSLELDIHLKLVSLDTNCVPDLPQPYLRCRPTLLVKQLREFVALQMHLKSEEVELLGTRRLGGEDKEIENLPVVTSASAAVASKDEMQSLEDNETLSRLKVDFFSSHEQHLIIAYRQKQTE

>EsRING1b Thhalv10007494m *Eutrema salsugineum*

MPALKSFSAAEEEDDQLCPNPEADRYKSEAEEEEEEDPDQMDEKDEDDNEGGDEEVEDDVTRDEEEEEEVEAEDEEALDEEDSKERSQSSTSEELSESEYMDIELGDIRRDVQCPICLGIIKKTRTVMECLHRFCRECIDKSMRLGNNECPACRKHCASRRSLRDDPKFDAFIAALFKNIDRFEEEELACHEDELARRKQIQVSIAQVSQRQSEALVKRRSFGKDAGVLPRPQRIGSGSRRRRNYRNMEQNTPEADDDDDDNNNRGKDSSSDEPCAEILLRKRKRRSTTRSTAHPSSSCQNNNDICANKGTEEAHHRDSRGISPGLVWNPEILAWGRGGTRSNTRQGNNNQGASSKSVRNARLNRLVEYLGSLESNSGELDIHLKLISLDTKCIPNLPQPYLCCRPTLLVKQLRKYVALQMQLKTEEVELLVSKEDDRAIEGKTSSSSTERLQSIQDDETLAKLRVDCISSHGYLMVVCRQKQIG

>FvRING1a mrna17739 *Fragaria vesca*

MSEYLEDCDGSPSQEKPEFVLVQLPDIRKDVHCPICLGIIKKTRTVMECLHRFCRECIDKSMRMGNNECPACRTHCASRRSLRDDPNYDALIATLYPDIEKYEEEELAFHEEERTRNEQIQASIAQIFQRQSEALIKRRSLGKDTQDGFNMRPQRNSRSAFQRRRNSRATELRGFEDNEDEIDKSPPAEERYTEVRPRRLRRRTGFRSSQPSSSVTNSNAGCTENDLEVSRDSRGISPGLVWSSEMLAWGRGGARSHTRHGVASGCSNKSSRSARLSKLVDYLRSLKENNDELDVHLMLISLDKRCTPNLQQPHLCCRPSLSVKHLREYVACQTPLQAEEVELFSVKGHHNSNDDKLTDHPSPLMNDLDSVPLLLDPCQYDLQILQAEETLAGIKANCTSSRDRLKDLSSLISHFVHQGWLEGVLLALTAPKFHHLLFADDSFLFEQLANVLGVKRVDKHEGYIGLPTSKIEAWLGDGSLSIAYSLPLIPHAMWLGKSRLPTHTPPDGWFSKINSEDEDASVVDNVVKEVRHMFTSRPQFCQRTSMFLHKQLSPS

>FvRING1b mrna29729 *Fragaria vesca*

MPAQKRGFEEAVEDGDDPAAKLHENGHEEPPEEEESDRSRSPEPSEDEKDDEKDELDFAMRAWFVRGSKHVAFCGRYRDASSFSEFLLEFAFVAEASGGFEKSRWSGEKTHPSGTKMEFTVGSTQLESFYSNMHAELGVLAKCNGLWLGWYNMDVQYGIIRKTRTVMECLHRFCRECIDKSMRLGNNECPACRTHCASRRSLRDDPKYDALIAAIYPDIDKYEEEELAFHEEEKARNKQIQASIAQTFRRQSEALGRKRTTAKATAAAFMRKSQGNYKNLRRRRNNKNAVEIQGSDDNEEANGNDGGKDSSSDERTEPRPKRPKRWTGARYYQRSPATANADGDYENDYEVNREVMGSSVGFVGSPERLAWGKGGLRSHTRYGSSSGANGKNARSRISKLVDYLRNLEANDKGLDIHLLLVSFDEQRIPSLQRRYLCCRPTLSVGQLCQYVAEQTALQPDEVEIYLIKEVQAKVSPSDPTDALVSTSGVLDSSNNTLLLLRDDESLAEVQARHLTRGHLVSTFIFTLGGLDFRFS

>GrRING1a Gorai.008G154600.1 *Gossypium raimondii*

MPAQKRSLPENLDDEESSLHHQSHTKQSRNEDGQHKPEDETTQLEQEQPEPDQDQQHLKQDPDDQNHQAQLQGDDEDEDDDEDGDDEDDSDGSQSSTSQEKPEFVLVELPEIRKDVQCPICLGIIKKTRTVMECLHRFCRECIDKSMRLGNNECPACRTHCASRRSLRDDPNYDALIAALYPDIDKYEEEELAFHEEERTRNKQIQASIAQIFQRQSEALVKRRSLGKESSTFSARSQRHHRSAHPRRRRNSRGVEHQGSEDNEDENDDNGGKDSSSTDERCTEVRQRRRKRRPGIRLSLPSSSVVNSDGGYVENDTEVSRDSRGISPGLVWNPDMLAWGRGGARSHTRHGNSSSGSSKSSRARLNRLVEYLRSLEENDDELDVHLKLISVDEHSTSSLQQPYLCCRPSLSVKQLCEYIALQTPLRAEEVEILMVKGQYHTDEKCTNPSEDTLQILEGQETLAGLKGKCSSGINHLILAYRQRQSC

>GrRING1b Gorai.010G155500.1 *Gossypium raimondii*

MPAQKPSSEAVFTPEGSDLLLLDQSINNHEQHPVETEDAADESDRCPCPCNENEQDYVIVKLSDVRKEVQCPICLGIIRKTRTVMECLHRFCRECIDKSMRMGNNECPACRTHCASRRSLRDDPNYDSLIAALYPDIEKHEEEELTLHEEEKARNKQIQASITQTLHRQLEVLGRKRTVKANASATIRRSNCRYQRRKYRASEPQESDNNEDANENGSTGSSLADEDLTEVKPKRLKRWEGRCSQPSSAASADGVGDENDSEVNRESLGVSAALSPSERLHL

GASGIRSHTAHGSLSGDENDSEVNRESLGLFAALNGLSERLHWGAGGMRSNTRHGSLSGGNGKKARNSRLPKLVDCLQNLEEKDDELDIHLMLVSIDEQRIPCLQRPYLCCRPTLLVRHLCQYVALQTALQASEIEIYLVKELYSTANMSTYKITKPGLVESVRDKLEVLKEEETLGGLGRQTSSHSHLILAYQKKENRNGQCQV

>PtRING1a Potri.007G138700 *Populus trichocarpa*

MPAHASASSPDNINPDNEQQQGEEQEQEQEQEHHHQQQRQDLSELSPPLQNGHPTDQEQQHHQNGQHQELEEEEEEVDKKEEAEVQEEEEEDYEVKEEEGEEEGGGGGGGGEGKEESDGTQSSSSSSSENEEKPEFVFVELLDIRKDVQCPICLGIIKKTRTVMECLHRFCRECIDKSMRMGNNECPACRTHCASRRSLRDDPNYDALIAALYPDIDKYEEEELAFHEEERTRNKQIQASIAQIIQRQSEALVKRRTLGKESTFMTRSQRTHRPISRRRRNSRGSDFQGYEDIEDENDDNIGKDSSSTDDRSTEVRQRRRKRQRTSQPSSSAVNSEGGCAENDLDSNRENRGLSPGLVWNTEMLAWGRGGTRSHTRHGNPSGCNNKNSRNTRLSKLVEYLRSLDEKNDELDIHLKLITLDKHSAPTLKQPYLCCRPSLSIKHLCEYVAHQTTLQAEEVEILLVKGKHQSDENLPTKHPQIPMDELQILKGQETVAGLKASCSSSRDHLILAYRQKEIIKS

>PtRING1b Potri.017G013000 *Populus trichocarpa*

MWGIKITKQISPIFLFLFLSLLHIHTTRSRPQRKRYIYFRIQQNHIFTERHTQKKHPSMPADNTPPSSPHNVNPHNEEQQHQQQQQRDLSELSPPRQNGHPTDQEPHHLQNGQHQEEGEEGEEEVDIKEQEVQKEEEGVEGGGGGDGEEGKEESDGTQSFSSSSSSSSCEDEEKPEFVFVELPDIRKDVQCPICLGIIKKTRTVMECLHRFCRECIDKSMRMGNNECPACRTHCASRRSLRDDPNYDALIAALYPDIDKYEEEELAFHEEEMTRNKQIQASIAQIIQRQSEALVKRRTMGKESTFMTRTRRTITRRRRNSRSSDFQGSEDIEDENDDNIGKDSSSADERSTEVRHRRPKRRWPSQPSSSAVNSEGGGAENDLDSNRENRGLSPGLAWNTEMLAWGRGGTRSHTRYGNPSGCNNKNSRNTRLSKLVEYLRSLDEKNDELDIHLKLISLGNQSTPSLQQPYLCCRPSLSVKHLCEYVAHQTTLQAEEVELLLIKGKAENLSAKHSQIPMDELQILKGQETVAGLKASCSSSRDHLILAYRQKEIIKS

>PtRING1c Potri.011G056300 *Populus trichocarpa*

MPAQKRSYETLQKEDDALLRRQQDHTNNNHEQKQEPRSDIDDDGGVESDRSQPSSNINEDVKEEYVVVKLSEIRKEVQCPICLGIIRKTRTVMECLHRFCRECIDKSMRLGNNECPACRTHCASRRSLRDDPNYDALIAALYPDIDKYEEEELAFQEDEKARNKEIQATIAQTFDRQAEALSRKRSTAKATAAVFARRTPSRFRDAHSRGRRNYRIAELQGSDDNEDANGGGGKDSSSTDEHSAEVKPKRYRRCSAAFNADGSGGENDSEVNKESVGASAGLISSSERLAWGKNGMRSHTRYGSANGSNVKNARNSRISKLADYLRNLDENDNELDINLMLVSFDEQRVPSLQRPYLCCRPTLSIKSLCQYVAFQTSLQANEVEIYLVQDMNSKLDFSLSMSSPVSRHGIIDPCKDKLQVLEQHETLGGLKTNNCIHGHLLLAYQKKP

>PtRING1d Potri.004G047000 *Populus trichocarpa*

MPAQKRSHETLQKEDDDALIQQQQDHTNNNNNNNNHEQKQQPHDDIDDDGGGGGGDEQSDRSQSSSSINEDVKEEYVVVKLSEIRKEVQCPICLGIIRKTRTVMECLHRFCRECIDKSMRLGNNECPACRTHCASRRSLRDDPNYDALISALYPDIDKYEEEELAFQEEEKARNKQIQATIAQTFHRQAEALSRKKTTAKVTAAAFARRTPSRFRDAHSRGRRNYRMAGFQGSDDNEDANGDGGKDSSSADEHSAEVKPKRCKKWAGARSAAVNADGGGDENDSEVNKESVGGSSGLVGSSERLAWGKNGMRSHTRYGSANGSNVKNARSSRISKLADYLRNLEGNDNELDINLMLVSFDEQRVPSLQRPYLCCRPTLSINKLCQYVAFQTSLQANEVEIYLVREMNSKVDFSISMSTPISKPGIIDPCKDKLQVLEEQETLGRLKTNNFIHGYLLLAYRKKV

>BdRING1a Bd2g21040 *Brachypodium distachyon*

MPAQKRPLPPSAGGDQDGNHVEEEAHGAAVSGTEAGGGGGGGGARSPKVVAANGAGPAELAKERRDGDSDAAEEALQQVEGGVGGGVDDEERDDDDSESSESEGDMDEFIVVKLMDIRKEVQCPICLGIIRKTRTVMECLHRFCRDCIDKSMRLGNNECPACRTHCKSRRSLRDDPNFDALILALYPDIDKYEEEELAFGEEEKTRNKKIQESIAETFRRQTEALVKKRSTAKAIASSRKTRGNLRAKRRGRTSSPDIVTSDIDDEDKEENGNDGSKESSSVDDRSPDVKVKRARRWPVPRPSPSKTIGSIDGTYEGNDDLGGVRDILTTSPLRGEMPAWGKNGTRSQTSRHGNSGGSSGRMVKSGRVAKMVDHLRNADESDSKLYLVLSPLDGQNMPKLEKPYLSCHSTFSVSHLCQFVALQLSRQAKEVEIYIRKNPGNKCFAMKDTDAAEEKLDLFNGLERLKEEESLAELYPAFASRQGDLELMYALRTQG

>BdRING1b Bd2g52362 *Brachypodium distachyon*

MECLHRFCRDCIDKSMRLGNNECPACRTHCASRRSLRDDPNYDALIATLYPDIDKYEEEELAFSEEEKTRNKKIQATIEETIRKQSEAIGKKRSTAKATANAFARKYRRNIQPRGRGKTIALDTSLAVSDDVDIEEGNANGASKESSSADNHSPDLMQKRGSKRPASLSSPARTIGTTDHGIEENGELVSGKESFTSSPLRGEMLAWGKNGTRSQTRYGNIGGLNGRLGKGGRGSKLVEHLRTTNEMDKEFNLYLILLPLDGQTTPSLEKPYVSCGPTVSIRHLVQFLALQLSRKVEELEIYIRMGLLNRSVTMLDPTSVETKLHQFDNLERLSEDKLLSDLHLSFASGHSDLELLYALKTEG

>OsRING1a Os01g58400 *Oryza sativa*

MPAQKRRLSSSPSSRPRDHVETNGMTGASKAAAGSGGGGGGGSGGGVPLPPRGGSAAAAAAKRAADPQPQREGDSDAEFGGGVDGDSESSQSDGDMDEFIIVKLAEIRKEVQCPICLGIIRKTRTVMECLHRFCRDCIDKSMRLGNNECPACRTHCASRRSLRDDPNYDALIAALYPDIDKYEEEELAFSEEERSRNKKIQATIEETIRRQSEAVGKKRSTAKATATVFARKYRRNMRTRGRGKTIAPDIAPTGSDNEDREEGNAIDTTKESSSADDRSSDLMPKRGRKRPASRASPARTIGSSDHVFEENDELIGGKESFTTSPLRGEMLAWGKNGTRSQTRHGSVGGSNGRMAKGGRVAKLVDHLRTTDDMDKEFNLYLVLLPLDEQSMPNLDKPYISCRPTLSIRHLVQFIALQLSRQVEELDIFMRIDHCNGSVTTQDCTTGVAKMRLSDGLERIREDKLLSELHPSFTSHHGDLELLYALKTQG

>OsRING1b Os05g41795 *Oryza sativa*

MPAQKRPAPEAASAPAGGDGHVEGGGAGGGGGADEDAHRGGERSPKVMNGGGPEKEKERRDADSDAEEEEEEAGGGGGGGGADEDRDSPSSESDGDMDEFILVKLMDIRKEVQCPICLGIIRKTRTVMECLHRFCRDCIDKSMRLGNNECPACRTHCASRRSLRDDPNYDALILALYPDIDKYEEEELAFSEEERTRNKKIQASIAETFRRQSEALVKKRSVAKATGSTITRRTRGNMRAKRRGRTSSPDIVATDNEDEDRDENGNEGSKESSSVDDRSPDVRQKRVRRWPVPRSSPAKSIGGIDSSFEDIDDLGSGRDIMSTSPLRGEMLAWGKNGTRSQTRHGNSGGSSGRMAKGGRVTKLVEYLRNTDEFDNKFNLYLVLLPLNGQSMPKLEKPYLSCQPTFSVRHLCQFVALQLSRHAKEVEIFIRKNPNNGCFASIDTSADEIKLNHDALERLEEEKSLSELYPSLASGHGDLELLYSLKAEV

>ZmRING1a GRMZM2G123791 *Zea mays*

MPTQKRPPPPPPPSPSPSAAPGAGSPPPSSPPLPTAEPAEKKPKLEVNGSAGAEANGNANDFGAKEGTEVEASESESEDADAAKQEFVPVKLSDVRKEVQCPICLGIIRKTRTVMECLHRFCRECIDKSMRLGNNECPACRTHCASRRSLRDDPNYDALIAALYPDIDKYEEEELAFNEEENDRNKQIQASIAEAFRKQSEVIGRKSTAKATAAAFVRRSRRNIRPNGQNTYFRGRGKASSDDVALACSEDEEDGNGESCSKEASSAEESSPEKKQKRLPKWPTPRSSPARACNDEVASDEKDDVGISRENFSTSPLRAWGKNGTRSQTRHSSFSGSNGRMVKGGRMIKLVECLRNSDDNEGERDVHLCLLPLDGQTAPNLEKSYLCCGPTLSIKQLCQFVASQTSHKDEEVEMYALKPSCSKPVSTNTCGPDKARLAGEERLSDLRSSFTFPNGVLELVYAIKVAN

>ZmRING1b GRMZM2G083515 *Zea mays*

MQAFLSSLSKCEEGHNNQTEDWMKQIWDVAYDIEDCIDDFAHSLRACNLCNGGIRFVRDKHPNRSIRDMPLQIESRRELLQRSGRSPDGISSVILVEKDRSFIKSKVVIRIMEYLNLFEIHGIIRKTRTVMECLHRFCRDCIDKSMRLGNNECPACRTHCASRRSLRDDPNYDALIAALYPDIDKYEEEEFAFSEQERIQNKKIQETIEETFRRQSDAIGKKRSMAKATATAFARKYRRTRGRVRTIPPDITPTGSSEEERGEGNSKETIREQSSTDDHSPDLRQKRCRKRSGPQGSPAGTIGSIDYSFEENDELVGGKEILATSPLQGEMLAWGGKWCTEPKSTWQCWFKWQDWKEWAHCKIG

>ZmRING1c GRMZM2G169645 *Zea mays*

MPAQKLLISSPTPDDSDGRVAVRHAAATRQRVSELGSRDGASREMDGKSVRQERSSHREPRDGVSQIATTSRWVAAADETEPTPTSSRSYHPMRAGTMDEFMLVKLAEIRKEVQCPICLGIIRKTRTVMECLHRFCRDCIDKSMRLGNNECPACRTHCASRRSLRDDPNYDALIAALYPDIDKYEEEEFAFSEQERIRNKKIQETIEETFRRQSDAIGKKRSMAKATATAFARKYRRTRGRVRTIPPDIAPTGSSEEESGEGNSKETIREQSSADDHSPDLRQKRCRKRSGPQGSPAGTIGSIDYSFEENDELVGGKEILATSPLQGEMLAWGKNGARSQNRHGSVGSNGRTGRSGRIAKLVDYLHTADEMDKEFQLFLLLLPVDGQTIPNLEKPYLSCRPTLSIKHLAQFVALQLSCEVEELEMYIRMDRHRVSVGSRPSSTGEAKSRPFDDLERLNEEKLVSELHPSFVSSNGDMVRSSLNRFNAIILACLVLFR

>ZmRING1d GRMZM2G383833 *Zea mays*

MECLHRFCRDCIDKSMRLGNNECPSCRTHCASRRSLRDDPNYDALIAALYPDIDKYEEEELAFNEQEMTRNQKIQETIEETFRRQSQAIGKKRSIAKETGTAFARKYRRNMRTRGRGRTTTPDIAPTSSNEEDREGENVNEVTKVPSSADDHSPDLRQKRCRKTSTSQASPARNIGSSDHSFEENDELVAAKEILATSPLRGEMLAWGKNGTRSQNRHGSAGSNGRIGRSGRIAKLVDHLCNADEMDKEFQLYLVLLPVDGQTIPHLEKPYLSCQPTLSIQHLVELIALQLSRKVEELEMYIRMDGDHGSVGSMACSIGEFDGLERLREDKLLSELHPSFASSNGDLELRYALKTRD

>SmRING 8697 *Selaginella moellendorffii*

ITVYLSEIRKEMQCPICLGIIRKTRTVMECLHRFCRECIDKSMRLGNNECPACRTHCASRRSLRDDPNFDSLIAALY

>PpRING1a Pp1s194_136V6 *Physcomitrella patens*

MQCPICLGIIRKTRTVMECLHRFCRECIDKSMRLGNNECPACRTHCASRRSLRDDPNFDALVAAIYPDLDEYEEEELAFFEDEALMNRQIQANIADTFKRQSEAIARRRTASKATAAAIVRKAHGNFRSVQSRQRGRGRGRRGGRRSRYSEGSARYEDDDDFYDEDKADTATSGDETQSEPEPNPKRRRAKVQTYSSGEENSENESLSAATRDQHEAPASFSGDESPADSLARESDGDASPTNGEGTLKSWAKGTRSSRYGSVSGVPVANGSRSIKSLPRTQDLADALSAAARTEKEDEPLNDGSDDEDTLPSLKRPHLCCPPNMTVHHLCKFLATRLSPPPEADLEILVESKTEQVSVPKPPPRLSKLSKGKAWASRGDSGKEVMLLSSGHTLESVLCDFWDYHGNLNCITVRNADGI

>PpRING1b Pp1s412_47V6 *Physcomitrella patens*

MQCPICLGIIRKTRTVMECLHRFCRECIDKSMRLGNNECPACRTHCASRRSLRDDPNFDALVAAIYPDLDEYEEEELAFFEDEALVNRQIQANIADTFKRQSEAIARRRTASKATAAAIVRKAHGNFRSVQNRQRGRGRGSRGGRRSRFSEGSARYEDDDDFYDDDKADTATSGDDTQSEPEPNPKRRRAKVQTYSSGEENSENESLSAATRDQHEAPASFSGDESPTDSLARESDADASPTNVEGMLKSWAKGTRSSRYGSVSGVPVANGNRSMKSLPRTKELADALLADARADKEDEFVVHLNLQPLIDGSDDEDTLPSLKRPHLCCPPKMTVHHLCKFLATRLSPPPEADLEILVESKTEQVSIPKPPLRLSKLSKGKAWASRGDSGKEVMLLSSGHTLESVLCDFWDYHGNLELLYRRKG

>CrRING Cre04.g213800.t1.3 *Chalamydomonas reinhardtii*

MPPLQQGSVPVALARLADDTRCPVCFGILKHTVTSTVCLHRFCAQCIDHCLAGRPPDHKDCPVCRTNLHSRRSLRPDPSFDRLLRALYGDVAAYGAAEDALVAECNAAAAQSAAAAQEEALRRAQKAAQAEALAANWPYYHRGGGKGGAGSGGGGGAGAGCVRTGSTPSAHAIARQCSNTTTSTTSHDTGGAGTGAAAAAGSGHGHGHAAAKRSREPSLDAAAAGAGSSNGCGSPRVKRRPSAAQLPGPTGSSLLPPACGSTTGAGTAAAAIGGAADGAAAPGMSRRFSVQQQQLQPQQLQQQQQRAAVTGIGRRASSILGSPPPAAAAVASGPTAGATAAANTPSRGGSARGGSPQPSPQPRGGHHHPHPHAHHQAPSGGGGGGGDDVAMVPSSPGLGHGSAHAAAAPPPQGLGPAASASLASLTSLLPRGAQLAVPTARCAAELSYRHPSMVVLQLRPAPLEAVAAAAGEAAAVAAAATGLAGGGGGAGGGAAAGGGCGGRVLRSATLPPLEKPFLLVPLKWTVAHIAQLLSQWLPPRRLPATTAGGAAGAAATSAAATAGELAPCPASCIRLEPATGCPRHHRYLRGCTTLGALAAELRAPGSGGGGGGGGGGGGAGGDGWPAGDRSRSREGSSTFSSASGGGGGGALSRRGSRSTPGVGEGAAGSGGGGGGGGSGDYSHLVLYYSVEPTMS

>VcRING Vocar20001790m *Volvox carteri*

MAGQGTIGLELLAARRRGHLDVVLVPVGGGGLIGGVASVLKAADPSIQVIGCQPAASDVMARSVAAGRIVPEVEVKNGGDDADGGESGWSTLSDATAGGVEYGSITLQPCMDCVDEWVTVSEAEIADALIGLLEEQSKLVEGTPGGSGIMWVTGAAACALAVFKRLAPRLAGKTVVLVCCGILKETVTSTVCLHRFCAACIDKCLAGRPPQHKDCPVCRANLHSRRSLRPDPNFDRLLRALYGDVVVYGQQEDSLVAAHNRAVAQAAREAQTAQLSRAQSEAAGAAAATAATAACPVKPVQQGTSQQSSERAAATPVAKRHHHRHHHYDDGHKQYDIPPDNRGAAAPTSRSPHGTRMRAGGTAVPVAMLKAPPSSPSPSSPPAHLTHHPRRGPRSAGAAAVQQVEPNAATAAAAMDVATRPAAPHGPLAVPAARSASELSFRHPGMVVVRLQPADPTGGGGGPPGTRAPLPALEKPFLLCPSKWTVGHIAQLLSQWIQQPGDVATAATARIPSAALRLVPAPGQLRGARRPLRSCTSLGALAAELPQIWEEGGGGGSRRGAASRGAGGGNPPLLVRFPRQASPPVCFLPSRSQPDPPSTCVLMIVTHWLRNLPGLRASQAAIACLNPTNQETGAKYQKQQIFPFIQQPVLITRNEPTTAVSHN

>HsRING1 Homo sapiens

MDGTEIAVSPRSLHSELMCPICLDMLKNTMTTKECLHRFCSDCIVTALRSGNKECPTCRKKLVSKRSLRPDPNFDALISKIYPSREEYEAHQDRVLIRLSRLHNQQALSSSIEEGLRMQAMHRAQRVRRPIPGSDQTTTMSGGEGEPGEGEGDGEDVSSDSAPDSAPGPAPKRPRGGGAGGSSVGTGGGGTGGVGGGAGSEDSGDRGGTLGGGTLGPPSPPGAPSPPEPGGEIELVFRPHPLLVEKGEYCQTRYVKTTGNATVDHLSKYLALRIALERRQQQEAGEPGGPGGGASDTGGPDGCGGEGGGAGGGDGPEEPALPSLEGVSEKQYTIYIAPGGGAFTTLNGSLTLELVNEKFWKVSRPLELCYAPTKDPK

**Core component: BMI1a/1b/1c**

>AtBMI1a AT1G06770 *Arabidopsis thaliana*

MMIKVKKETMRACLSCSICDNILRDATTISECLHTFCRKCIYEKITEDEIETCPVCNIDLGSTPLEKLRPDHNLQDLRAKIFALKRRKVKAPGIVSLPGKRKERSISSLVVSTPMVSAQAGTTRRRTKAPTRKELRNGSLAERTVKKEESSGDELLESTSSPDTLNKFTQNKRQSKKSCKESISNKENKDGDEPWDSKMDWKPLNFLVEVANGTKPLKSSASQGSGSKSEHANVSRNQFQGSKTKTKNKKRKCKREDDKSNNGDPTTSETVTPKRMRTTQRKRSATTLGDSRNLPQPDESSAKQERRNGPVWFSLVASNDQEGGTSLPQIPANFLRIRDGNTTVSFIQKYLMRKLDLESENEIEIKCMGEAVIPTLTLYNLVDLWLQKSSNHQRFAALVGSSAKDFTMVLVYARKLPECNM

>AtBMI1b AT2G30580 *Arabidopsis thaliana*

MEGDMVAKVKRETVVACMTCPLCDKLLRDATTISECLHTFCRKCIYEKITEDEIESCPVCDIDLGGTPLEKLRPDHILQDLRAKLFPLKRKKERAPEVVSSISLPAKRKERSISSLVVSTPRVSAQAGTTGKRTKAATRKDVRGSGSFTKRTVKKEEEFGDDHVESASSPETLKKFTQNKRQSSYANPNQSLSNRRNKDVDEPWDSKLHLWKPLNFLVDVANGTKDPKSELGNASHNDVQGSKTKTKDHKRKCKLEEEISNNGDPTTSETATLKRTRRTRRKRSSTFGDSRIPLLPGAASLKQERRNGHVWFSLVASSNQEGEASLPQIPANYLRIRDGNIPVSFIQKYLMRKLDLKSEDEVEITCMGEPVIPTLQLHSLVDLWLETTSKHQRVAASIGSSAKEFVMVLVYSRKLPECNN

>AtBMI1c AT3G23060 *Arabidopsis thaliana*

MLTKVLSKEVKPCLACPICTNPFKDATTISECLHTFCRSCIRNKFINERVNACPVCNVNLGVFPLEKLRSDCTWQDLKLKIYRAMMESLKKAGPKTVAASVKSSKKKRKSRTSLRVSSSRVSSSPDTPLEPANVVVEPPNVVVEEKHRETVLALQSTRKPIITFQKRGRKASLPKKIDSKPEPELPPKEPKIKNLFDLNNEPEDNGLDEAEGSTFQEVVPKEKDLCKPIFSLSVTLNINDTPPDIVEPEISSDDDTEESVEPIQNKCVVNRETKEVPVQVNQNSLLISSDRDREDNSGQKLKTNGAATSRSRKKKGKKPVEKSYSLRPRIGRRTVNPAAGTTTPEAPVSVEEEMKVEEGRNNNPVWFSLKPSKTQNIEMLLPPI

>BsBMI1a Bostr.2265s0001.1 *Boechera stricta*

LCDNILRDATTISECLHTFCRKCIYEKITEDEIESCPVCNIDLGGTPLEKLRPDHNLQDLRAKIFPLKRKKVKAPEIVSLPAKRKERSISSLVVTTPRVSAQTGTTGRRTKAATRKELRNGSLAERSVKKEESSGDELLESTSSPGTLNKFNQNKRQLKKSCKETFSNKENKDGDEPWDSKLDWKPLNFLVEVANRTKPPKSSASQGSGSKSEHALASRNQFQGNKTKTKDQKRKCKREDEKSNNGDPTTSETATPKRLRRTRLKRSATTFGDSRNLPQPNASAKQERRNSPVWFSLVTSNDK

>BsBMI1b Bostr.24513s0177.1 *Boechera stricta*

MEGDMVAKVKRETVVACMTCPLCDKLLRDATTISECLHTFCRKCIYEKITEDEIESCPVCDIDLGGTPLEKLRPDHILQDLRAKLFPLKRKKERAPETVSSITLPARRKERSISSLVVSTPRVSAQAGTTGKRTKTATRKDVRGSGSFTKRTVKKEEEFGDDHTESGSSPETLKKFTQNKRQVKKSSYAEPNQSHSNRRNKDVDEPWDSKLHLWKPLNFLVEVANGTEDPKSEHGNASHNDVQGSKTKTKDHKRKCKLEEEISNNGDPTTPETATLKRTRRTRRKRSSTFGDSRIPPLPGAASLKQERRNGHVWFSLVASNNQEGETSLPQIPANYLRIKDGNIPVSFIQKYLMRKLDLKSETEVEITCMGEPVIPTLQLHSLVELWLETTSKHQRVAASIGSSAKEFVMVLVYARKLPECTN

>BsBMI1c Bostr.19424s0348.1 *Boechera stricta*

MPSKVKKKEVEECFTCPLCTNLFDRPTTISECLHTFCRNCIHDKLMTERLKYCPVCNVDLGVFPLDKLRSDYSLEDMMRKIFPPKTKSVKAGPETVVASLKSSKKKEKPLTSLLASSPRVSLSPDAPLEPNVVVEEKQSEKVEALPRIQTSAKPIITFKKRERKAPILKKNDSGMESEQPHKEAEVKKFQFDLNNEPDNGLDEAEASTYQNCIPKEKDSEKDNEAIPVLFDLCKSLSDAKEVAPLNINDTPVIVEPVITSNGIPISGGDTEEGVDPIHNNCVVNTETEEVPVQVNQNSILISSDRDTEEVVSGQKLKNNGKGKKECATSRSRKKKGKKQVEKSYSLRPRKDGRTINPAASTPEAAISVEAEQKVKGRNNPVWFSLVPARTQNKDLLLPPISARYLRVKDRNMTVSYVKKYLMVKLGLESEDQVEIWLRDEPVSSSQTLHNLVDWWVQTTPLPKRKSAMVGSSAADFVMVLYYSFKSEFSCSE

>BrBMI1a Bra015525 *Brassica rapa*

MSCSNSRNTVDEHGNGPMLHTTGQKPHAGVRLEMTVPETQYTQRVFGSPSSRGPSVPPPPMTMRQQNPIPGESTSDTHAEADVMRSLRFYLVLEEIVVGGGGSGGIIHFHWSDIPTGDVYHHIVNVKGRVYVRKCIYEKITEDEIECCPVCNIDLGGTPLDKLRPDHNLQDLRAKIFPLKGRKVKAPEPARKKERSMSYLVDVTKTKASDQAGRRRTKTVTRKELLQDSASLAEKEEESLLKGHLLESTSSPNKFTQNKKDSDEPWDPESYWKPLNFLVEVANRTQTLKSSSASQGPGSKSEKANASSHKQIQPRVKDHKSRYKREYEKSAKREMRDGPVWFSLVASTDQ

DGDIPVSFIQKYLMRKLNLQSETEVEIRCMGEAVIPALKLQKLVDLWLHRGSKRQKIDAWIGSSAQRIT

>BrBMI1b Bra018304.1 *Brassica rapa*

MEGEMVAKVKRETVVACMTCSLCDNLLRDATTISECLHTWVSHALLYPAFCRKCIYEKITEDEIESCPVCDIDLGGAPLEKLRPDHILQDLKAKIFTPKRKRERAPEVVSSITLPARRKERSISSLVVDTPRVSAQTGTTGKRTKSLMRKDVRGSGSFTKRTVKKEEEIGDDHTESGSSPETLKDFTQSKRQSSYADPSQSLSNRRNKDGAEPWDSKLNLWKPLNFLVDVANGTKSEHGNASHADVQGNKTKTKDYKRKCKLEEEIRNNGDPTTSETATVKRTRRTRRERSSTFGDSRIPPLPDSESLKQERRKGPVWFSLVASNNQLTDFREGETSLPQIPANYLRIRDGNIPVSFIQKYLMRKLDLKSETEVEIRCMGEAVIPTLELHSLVELWLETTSKHERVAATIGSSAKEFVMVLAYARKVPECNN

>BrBMI1c Bra001901.1 *Brassica rapa*

MVMEKKMTVLKAKRQDMKACFSCPICNNLYDRATTISECLHTYIKNLFFYLYLGNTVCKRCIEDKLVVENLKACPVCNVDLGVAPLDKLRSDNMWDELRIKVFKQKPKSVKAAAATIETASASLTYSRKKKTVTSPRASNSPEPTPDGALESEKLEEEMEAVSLNQSSCALVSNFRRRVRKNLAPKRNENPLEPQQLHVESDNGFEIVLNFEQDNGLAGALTSNGCIQKVPVSSDDLGKPLSNGKESNGTALNVETVITTNGNTEEVTGEANENNVLVSSENVPKEVSGEKPKSNGVEKQENVSTISAGKKGKGKAYAQRVLRPRKQGNMSSDGASEAAVSKEAEDKVEGSNKKVWLSLIAAANQNTERPLLPQISNAYIRTDGNLTVSYVKKYLANKLGLQSEDQVEIWLRQEPVCSTQKLHNLVDWWVQSTPVAERKSAMVGSSGAEFVMVLHYSGSHLCE

>CgBMI1a Cagra.1671s0301.1 *Capsella grandiflora*

MVSKVKTETMRACLSCPLCDNILRDATTISECLHTFCRKCIYEKITEDEIESCPVCNIDLGGTPLEKLRPDHNLQDLRAKIFPLKRKKVKAPEIVSLPAKRKERSISSLVVTTPRVSAQTGTTGRRSKASTRKELRNGSLAERSVKKEESSGDELTNSPDTLNKFTQKKRQLKKSCKVSFSNKENKDGDEPWDSKLDWKPLNFLVEVANRTKPPKSSASQGSGTKSELASRNQFQGFKTKTKDPKSKCKLEDEKSNNGDPMTSETATPKRLRRTRRKRSATTFGDSRNLAQPDEASAQQERRNGPVWFSLVTSNDKEGETSLPQIPSSFLRIKDGNIPVSFIQKYLMGKLDIESETEIEITCMGEVMIPTLKLHDVVDLWLQKSSKHQRFAASIGSSGKDFMMVLAYARKPPKCNK

>CgBMI1b Cagra.0567s0022.1 *Capsella grandiflora*

MEGEMVVKVKRDTVVACMTCPLCENLLRDATTISECLHTFCRKCIYEKITEDEIESCPVCDIDLGGAPLEKLRPDHILQDLRAKLFPLKRKKERAPEIVSSVSLPARRKERSISSLVVSTPRVSAQAGTTGKRTKTGTRKDVRGSGSFTKRTVKKEEEFGDDQTESASSPETLQKFTQNRRQSSNAEPNQSLSNRRNKNADEPWDSKLHLWKPLNFLVEVANGTKDPKSEHGNASHSDVQGSKTKSKDHKRKCKLEEEISNSGDPATSETATLKRTRRTRRKRSSTFADSRISPLPCAASPKQERRNGPVWFSLVASTNQETSLPQIPANYLRIRDGTTPVSSVQKYLMKKLDLKSENEVEITCMGEPVLPTLQLHSLVELWLETTPKEQRVTASVGSSAKEFVMVLVYSRKLPECYN

>OrBMI1a orange1.1g013534m *Citrus sinensis*

MRVEREMASNNLVVKVKRETIAACMTCPICNTLLRDATTISECLHTFCRKCIYDKISDEEIECCPVCNIDLGCVPLEKLRPDHTLQDVRAKIFPLKRRKVKAPEAVPSVTLPVRRKERSLSSLVVSAPRVSAQTTMTRRRSNAAARKAAALRGSNLSTDKPPKKEEDSVEDNEDGASSPETLNKLSQNVKPMQSSSSAQPSQPISSKEKDNDAEQCEGKSDLWKPLNFLVEVASRTRSLKSNSLGSEAKAEPTTITDNEAQVHKTKNKEDKCKSKSGNKNNNPDPTTSETINPKRLRRIRRKTAAAFGDSNISPQAVLDAAGAKYERRNGPIWFSLVASEDQRGSLPLPQISSSYLRIKDRNIPVSFIQKYLMMKLDLPSESEVEIKCMGQPVIPTLQLYNLVDLWLQTASTSDRVPAMIGSSAKDFVMVLTYARKVRDIR

>OrBMI1b orange1.1g017736m *Citrus sinensis*

MDTVRTTARHVVKVKRETLEACMTCPLCNSLLREATTISLCLHTFCRKCIYEKLSDEEADCCPVCNIDLGCLPVEKLRPDHNLQDIRAKIFPFKRRKVQAPEVMPSISLPAKRKERSLSSLVVSTPKVPQHTGLTGKRTKGSTRKAAALRGCGFMLEDKKESSIEDHPMSSSSPDSLNKNAQNRRQESYKAEPSSEQGPNKVGEITEAKADLWTPLNCLVEAANRTKSSKSNSQGPSLAKTDLHNFPDSEVFMPETKVKVELPNSPGGEICSSKTKMKEHGLKTKYQNDKNGIGLHPGPMKRRRRAAAKKREVGTEESCAAAQAMLDAAGAKYKRRNTPIWFSLVASEDQKGDGSLPQISTCYLRIK

>OrBMI1c orange1.1g017653m *Citrus sinensis*

MATTEQTLKVNREKLVACMTCPLCSKLFRDATTISECLHSFCRKCIYEKITEEEIDSCPVCNTDLGCAPLEKLRADHNLQDLRIKIFPSKRRNLDAPDSVSSVPLPARRKEISLSSLAISTPKSPVKSSSSGRRSKPVPKKTLVQEEYTSPIEEPIKDVEDPPELSSEPLCRNTQTKRQILSAAESSIQHTPDKGTEDIARPFDGKSDLWKPLNVLVEAATRPRKSLGRPKKAAVSAGLNVSAQAVVDTNQRFDGRFGPIWFSLVASDEQEGDEPLPQISSCYLRVKDGRLPVSFIKRYIVKKLNLISEAEVEISLRGQPVLSTLELHNLINWWVQTSSASERIQTVVGSSAKDFVMVLSYGRKAQPP

>CsBMI1a Cucsa.168400.1 *Cucumis sativus*

MANRVVKVKREAIAACITCPLCNKLLKEATTISECLHTFCRKCISDKISDEEIENCPVCNIDLGCAPLEKLRPDHNLEDLRAKIFPSKRRKVKTPEVAPVVLPPVRRKERSLSSLVVNSPRVSSHATTTGRRTAAAAARIASILRTPRVSSEKRVKKEDDSVDDRSESSSSFETSDKFNQNKRTDSSPTKSTIPLRSKETENGVNSVERNLDIWKPLNCLVEVANRSKCSKSNSQGFETKVEAAEANGSEAQASKSRNREGKRKQKRENGKTRADPVSPETEKPKKLRRVRQKRESFYGDSSLTPQVVLDASSARHEIKAGPIWLSLIASDQEGDVSLPQIPAKYLRIKDRNLPVSFVQKYLMRKLDLPSESEVEVKCMGHPVVPTLDLHSLVDLWLQTASTSEKIPASIGSSAEDFIMVLYYARKTIVS

>CsBMI1b Cucsa.133170.1 *Cucumis sativus*

MAGQVVRVKREILEACMTCPLCNKLLKEATTISLCLHTFCRKCIYEKLSDDEVDCCPVCDIDLGCLPVEKLRPDHNLQDIRAKIFPLKRRKICAPEISPLASLPVKRKERSLSSLVVNTPKVSMQSGGLTGRRSKNVGRTAAALRRCNFGTEEPLKKEEDSGEDHTTSSSSSDYLKNVRFRQRRQDSSMPEPSNSLRHEHLKNNVEAVEGKADLWTPLNCLVEAANRTKSTKLNFQGSSMAKLEPSNVADGDVDAEETKEKALSLGAPNYGLFMPKARNKEHGSNPKAKDNHNNGTASLPETMKRKRLRATARNKAAASAELSSPAQLVLDASAAKCRRNSPIWFTLIASEDRKGGFPLPQISTPYLRIKDGKMPVSSIQKYLVKKLDLKSEAEVSSLLPSKQVKKTNQTSTAKKTPASVGSSAKDFVMVLSYCRKVQSP

>CsBMI1c Cucsa.254880.1 *Cucumis sativus*

MATATATVKLPTEKLMGCLTCPLCHNLFTNATTISECLHTFCRDCIYEKIAEEELEGCPVCNTNLGGVPLEKLRADHTMDDLREKIFSCKWRKEKEPEETQLSSSSSVSLPTKRKEGPVSILEIDNTYTTSDSGTSKKLSASTAQESLTEQQYLSTMIDHTKLNFDQNKQNNGQVMERIIDDLNKKEASVTCSNDCKIQDTLPKAETIQIDDSYCNVQERGYKPIMERGERESHGTSGSIEHGKLQDPLGDIDADRQCNKSSSPIWFQLVASDHQEGNEPLPQISSNYLRVSDGSIPVSFIQKYLAKKLGLASEIEVEISFKGQPVSSTLHLHDIVELWRHTTTKVELIQISVGSSAKDFVMVLSYGR

>EsBMI1a Thhalv10007754m *Eutrema salsugineum*

MVSQVKRETMKACLSCPLCDHILRDATTISECLHTFCRKCIYEKITEDEIESCPVCNIDLGSTPLEKLRPDHNLQDLRAKIFPLKRRKVKAPEIVSLPARRKERSMSSLVVSTPSVSAQTGRRTKAATRKELRDSGSLAERTVKKEESFGDDLLENTSSPDKFTQNKKEVKKSYAESFSNKEKKDGDEPWDSKMDWKPLNFLVDVANRTKPLKSSASQGQGSKSEHAKASHKQFQGRKTKFKDQKSRCEREDETTNHSDPTTSETATPKRMRRTERKRSATNFGDTRNSPQQDESSAKRERRYGPVWFSLVASSDQEVCLPQIPANFLRIRDGNIPVSFIQKYLMRKLDLESESEIEIRCMGEAVMPTLELQKVVDLWLQRSSNRQRFAASIGSSAKDYMMVLVYARKLPECNK

>EsBMI1b Thhalv10016711m *Eutrema salsugineum*

MEGDMVAKVKRETVVACMTCPLCDKLLRDATTISECLHTFCRKCIYEKITEDEIECCPVCDIDLGGTPLEKLRPDHILQDLRAKIFPLKRKRERAPEVVSSITLPARRKERSISSLVVSTPRVSAQTGTTGKRTKSARGSGSFTKRTVKKEEEVGDDHTESASSPETLKKFTQNKRQSSYAEPNQSLSNRRNKDVNEPWDSKVHLWKPLNFLVDVANGTKPLKSSSSQGLDPKSEHGDASHNDVSKTKTKDHKRKCKLEEEISKNGDPTTSETATLKRTRRTRRKRSSTFGDSSIPPLLDGASLKLDRRNGPVWFSLVASNNQEGETSLPQIPANYLRIRDGNIPVSFIQKYLMRKLDLKSETEVEIRCMGEPVIPTLQLHSLVELWLQTTSKHQRVAASIGSSAKEFVMVLVYARKLLEGNN

>EsBMI1c Thhalv10022423m *Eutrema salsugineum*

MERKITLKAKKQDMERCFTCLICNKLYVRATSINECLHTFCRKCIEDKLIEENLKACPICNADLGVAPLDRLRTDNTWEDLRLNVFKPKPNSVKAETETVAASLQYSRKKKKSVSSLLATPSREPASPESDPDAPLEPEKIGEETEAVPVPLIQSLSRPASKFRKRPRKNPRPKKSETRLEPEQPHKESDNGFLNIEQDNGLARASTSKEYNSEKVNKEIPILSDVLSKPLSTAMEVTPIKSNGIGKANQDNVSSCGGDKEEVSGVKSEECAEENQNTVLNHSEGNNTVTGIANQNSVLVSSDCHTEEVSGEKPENNNGEDQNQENVSTSGKISGEKGKAKAYSPPVLRPRRQRKMNSDGTSRNAIPMELENRAGASNSPVWFSLVAAGNQNTDRPLPQISPLYLRVTNGNLPVSYVKKYLVKKLNLESEDEVDIWLRQEPVCSTQRLHNLVDWWIQTTPISERRSAMVGSSGAEFVMVLYYSRSHLFC

>FvBMI1a mrna08209 *Fragaria vesca*

MANQVVKVQREKLEACMTCPLCNKLFKDATTISECLHTCQKCIVEKITEEDIENCPICKTDLGSVPLEKLRPDHSWQDVRAKIFPSKKRKVSEPESTPSAFPARRKERSLSSLVVSTPRVSSTPATMTGKRTKAVARKASTLQGSSLLVERSANNEDESAEDHADSQSSAEASKKSGENTRPSSISAEPNETVSNKESENGAESWDGKDLWKPLNCLVEVANRTKSLKPNSQGSERVEPAISPGETQVRKFKSKENKQKSKVDDEKNNTHLPSPETNKPKKLRRARQKREASGEPSVTPQAVLDAASGKPERRVGPIWFSLVASEEQEGEECLPQIPTNYLRIKDGSVPVSFIQKYLMRKLDLTSETEIEIKCMGQPVSPSLEMYNLVDLWLQTASPTQRIPASIGSSAKDFVMVLGSKDKRIPTEPLVYDPPCIVRHVVGKWPLPLLYLIKDFTYFKTKGLSALIHHAVDYSFISGLKMAPQAPTLHHLFFADESPLYGTANANECQNFRTILSLYELASDVQLVDEHDRYLGLPLHVGKSKTAKFEYIKEKVTKKLCASFFWGDTDEKKKVQWRSWERMCLTKAEGGMGFKIIYAYNLAMLAKQGWRILSKPHSLIAQIFKARYYPHSSFLEAELGEAPSFSWRSIMQGRKVLKADLQWKIGDGKNGVIWNDKWLPLSTPEFIQKPVDCTLTRVNELINEHTREWHSNVITATFPPYIAAKIMCIPLSRKTTSDRLIWFPEKKGMFSVKTAYWIARNQVLSEVLTTTSSGNPFSVLWKKLWAAKVPGKVKKNTIKPIAVENSTLTVKKVWKPASNGNMILNVDTAFLPNQHREGIGGVLRDAAGNFIAAFTRSISHTASPKQCELLAIREGMDMLYSLQTCNVQIHSECQEAVAEVKTPDYELLANGGIVDDIRLVQQKLMNVCLKHIPRTCNIIAHRLAAIAFKDNLTCTGLDYPPDCITDILQIEIERLH

>FvBMI1b mrna16580 *Fragaria vesca*

MAGHVVKVKREALVACMTCPLCHKLLKEATTISLCLHTKCILEKLSDEEGDCCPVCEIDLGCMPVEKLRPDHNLQDIRAKIFPLKRRKINAPESPSVTLPVKRKERSLSSLVVSTRKVPVQAGLTGKRSRALTRKAAALRECSFSVEGTSKREDSSEDHPRSSSSPDAQDKMQDSSMPENSYEQKPKKDDVEVIEGKADLWTPLNCLVEAANRTKSSKSNPQGQLPAKSEPLNALDSISELYMPENNTDAESPDALESHVHVPKVKIKEPENSTRGKKNINAAAVTGSMKRKRFHAGNRNRGAPSVESGDSTQDVRGAKHNRRNNPIWFSLVAAENWDGKMPVTFIQKYLVKKLELTSEAEVEIMCRGQPVHPTLQLHNLVDLWFRNGSTTKKLPATVGSSAKDFVMGGGSETEVTWEDQQNINKFGRLNNRFHELEDEIKVAKSRNLAAAGILPAFTLGYEHLSLESNDNLEDAGNELILTDEDVVRFQIGEVFAHVPKEEVENRIEEMKEVTSKSLEKLYEEKESVLAQMTELKQILYGKFKDSINLEED*

>GrBMI1a Gorai.007G142500.1 *Gossypium raimondii*

MANQVVKVRRETIAACMTCPLCNKLLRDATTISECLHTFCRKCIYVKIEDEELECCPICNIDLGCVPLEKLRPDHNLQDVRSKIFPFRRRKVKAPEVLPLVTMPTRRKERSLSSLVVNAPKVSTQAALTGRRTKAVARKANALRGSSFSVERPLKREDDSMEEHQESASSPETLNKFTQNKRQCISSAEPSQHLNKDAKNGDESWDGKLDLWKPLNCLVEVANRTKSFKSSSQGSDPKLEPTHVPNIEAQMCKSKNKEDKCKTKIEDEKNITGPATSETVTPKKLRRIRRKRASGFGDSVLSPQAVLDAYDPKHGRRIGPVWFSLVASEDQEGGAPLPQIPANYLRIKDGNMPVSFIQKYLMKKLDLTDEAEVEIKCMGQPVLPTLQLYNLVDLWLQTASTSQRVAASVGSCAKDFVMVLAYARKVSGQQ

>GrBMI1b Gorai.013G104800.1 *Gossypium raimondii*

MANQVVKVKREAIAACMTCPLCNKLLRDATTISECLHTFCRKCIYDKIEEEELECCPICNIDLGCVPLEKLRPDHNLQDVRTKIFPLKRRKVKAPEVVPPVTIPTRRKERSLSSLVVNAPKVSSQTTMTGRRTKAFTRKAGALRHSSFPIEKPVKGDEDFMEDHQEIASSPETLNKFTQNKRQCTSSAEHIQNMNKEAENGGKAWDGKLDLWKPLNCLVEVANRTKSFKSNSQGSDSKLEPSCVPTIEAHTCKSKHREYKCKTKLEDERTSAGPATSETVTPKKLRRVSRKRASGFGDSGISPQAVLDAAGPKHERRIGPVWFSLVASENQEGDAPLPQISANYLRIKDGNIPVSFIQKYLKKKLNLTDEAEVEIKCMGQPVVPTLQLYNLVDLWLQTAATSQQVPATIGSSAKEFVMVLAYARKAPDE

>GrBMI1c Gorai.004G074300.1 *Gossypium raimondii*

MANQVVKVRREAVAACMTCPLCNTLVKDATTISECLHTFCRKCIFDKIEEEDLECCPICNIDLGSVPLEKLRPDHNLQDVRAKIFPLKGRKAKSPEVLPPVTMPTRRKERSLSSLVVNAPKVSTQTTMTGRRTKAVARKAGSLRGSSFSVEKPVKRERDSVEDHKESASSPETLNKFTQNKRQCTSSAEPSQQKDKGVENGGESRDGKLDLWKPLDCLAEVANRSKSFKSNSQGCDSKLELEPTHVINPEAQTCRNRHRKDNGKTKVEEEKNNAAPATTSGIVNPKKLRRIRQKSGSGFGDSGISLQAVLDAAGPRHEKRIGPVWFSLVASEDQVGEVPLPQIPANFLRIKDGNIPVSFIKKYLMKKLHLADEDEVEIKFMGQPVVPTLQLYNLVHLWLQKASTSQRVPASVGSSAKDFIMVLAYARKAPQQL

>GrBMI1d Gorai.007G278000.1 *Gossypium raimondii*

MMAGHHRVVKVKRETLESCMTCPLCNKLLKEATTISLCLHTFCRKCIYEKLSDEGMDCCPVCDIELGCLPVDKLRPDHNLQDVRAKIFPYKRRKISAPEVMPAASPPVKRKERSLSSLVVNTPKVPMQRGLTGRRTKPTTRKRIAAFRGCSFSVEESLKKEDSAEDHPSGSSSPDSFHKISQSKRQDSMAQPSSEHRPNEDTDDVEVMEGKADLWTPLNCLVEAANRKSSKLNSQGSTASMTEQHNGPDCCSHAPEAKPSPQSPAVPDGKLSIHKSKSKEHRNNSVILEEENGTNLIKRPVKRRRLHAEAQKKVAASNRIMLDALGSKWNRKNNPIWFSLVACEGQIGHTSLPQISACYLRIKDGKMPVSFIQKYLVKKLDLSSEAEVEIMCRGQPVLPSLQLHNLVDLWFRTASTAKKVPASVGSSAKDFVMVLSYCRKVQAP

>GrBMI1e Gorai.001G015000.1 *Gossypium raimondii*

MSNDYINVQRRPLMEFLTCPLCSNIYREATTICVCLHTFCKQCIYEKVDKEKTHYCPVCDAYLGSIPQHMLRNDHKLEKLVKQIFPLEKPNEDNTENRFSNHSMNPIMKQNELDNSEPTVREFSLERSRKGLCWEHLGGRKDGETYGTEVQVKRRRRMQRKPYCKSLIGQREEGLKIGTANEMSKTNTSGLEKPTDSLNRCGEIVYLNQYSGCSPRPVWFCLLASQETNDMALPQIPKPFISTKNGDLAISIVNKYLAMKLNLKHESEVEVMCLGHPLMPTLTLNNLIDTWLEAVSDIEPVPAEEGDGRNFLMELTYRRSMKQCTLQ

>PtBMI1a Potri.002G044100 *Populus trichocarpa*

MGNQNHQRHHQVVKVRRETIAACMTCLLCNKLLRDATTISECLHTFCRKCIYRRISNEGLDSCPICNINLGCVPLEKLRPDHNLQDVRSKIFPYKKRKVEAPEVAESVALPVTRRKERSLSSLVVCTPKVSTHTTTTGRRTKPFPRKAAALRGTNFSIEKPNNKEHVPVEDSPESSSSPETLKKPNQNIKQNSPCTEPSQPDPDEAENGTEPRDGKSDLWQPLNFLVEVANRTKSFKSSPQLNDAKSESRPVHDNEPRALRTKFKGNKDKSKVKDEKNNIDNVSEGPVEPKRLRRIRQKRAVFNNISGISSPAVLDTAAAKQERRSGTVWFSLLASEQEGEAPLPQIPSSYLRLKDGNVPVSFIQKYLMKKLDLASEAEVEIRCMGRPVIPTLLLYNLVDQWLQTVPKTEQVPVTAGFSAKDYVMVLAYARKVPNQ

>PtBMI1b Potri.005G219000 *Populus trichocarpa*

MGNQNHQHRVVKVRRETIEACMTCPLCNKLLRDATTISECLHTFCRKCIYQRISDEGLDSCPICNINLGCIPLEKLRPDHNLQDVRSKIFPYKRRKVEAPEVVESVTLPVIRRKERSLSSLVVSTPKVSTQTTTTGRRTKPFPRKAAALRGSGFSIEKPIKKEHDRAEDSPESSSSPETPKKFNQNTRQNSSSAEPSQPAPDDEAENGAEPRDGKSDLWQPLNFLVEVANRTKSFKSIPQVNDAKLESRPVRENEPQVHKTKFKENKDKSKVKDEKNNIDNVSEGTVEPKRLRRIRRKKAAFNDVSGISSPAVLHTAAAKQERRSGPVWFSLLASEEQEGDAPLPQIPSSYLRLKDGNVPVSFIQKYLVKKLDLTSEVEVEIRCMGRPVIPTLLLCNLVDQWLQAAPKPEQVPVSAGSSAKDFVMVLAYARKVPNP

>PtBMI1c Potri.010G077800 *Populus trichocarpa*

MMLGQVVKVQREEIAACMTCPLCNKLFRDATTISECLHTFCRKCIYKKITDEELDSCPVCDTELGCSPLEKLRADHSWQDLRAKIFLSNRKKAKEPETVSLVPEDERSLSSLVVSTPKISVKSFLTGKRSKPIARKRESPVLIKELVKKVDDYYESLSSPETLSKIAQTKRQNSSTAESPKQHKPNKVSEDGVKPCKGKADFWKPLNCSVEGSSKTKSNKSELQEILVQIKKLDAQEKAQSLKTSVKEHGDKSKVNGEESNSTSWPSVSVKSRRLQGMQQKRAAPSEGLNIPAQTIVDANSKCDTRLSPIWFSLVASDHEQGGSAPLPQISSCYLRVKDGSLPVSYIKKYLAQKLGLVREAEVEISMRGQPVVSTLQLHNLVDWWLQTASASERIRTTVGSSAKDFVMVLSYGRKAHPP

>PtBMI1d Potri.013G042000 *Populus trichocarpa*

MAGQTVKVQRQVLEACMTCPLCNKLLKEATTISLCLHSFCRKCIYEKLSDEEVDCCPVCNIDLGCLPVEKLRPDHNLQDIRAKVFPFKRRKVNAPEIMPSIALPAKRKERSLSSLVVSTPKVPIQNGLTGRRSKAGARKAAALRGCNFTVEESKKEDSAEDNPMSPSSPGSPVKSIQKRRLDDDVEIIEGKADLWTPLNCLVEAANRTKSSKSHSQGLSLVKSGMLDDPDCEPHLYETKSRAESPGGHHNEVYMSKTKNKEHGQGIGIQDDKNGKNSLPISVKRRRLTAARKRAALSEGLSASAQAMIDAAGAKSNRRNGPIWFSLVASEDQKEDASLPQISTCYLRIKDGKMPVSFIQKYLVKKLDLDSETEVEILCRGQPVGPTLPLQNLVDLWFRTGSTSKKVPASVGSSAKDFVMVLSYCRKVQES

>PtBMI1e Potri.005G054400 *Populus trichocarpa*

MSGQIVKVKREILEACMTCPLCNKLLKEATTIFSCLHTFCRKCIYEKLSDEEVDCCPVCNMNLGCLPVDKLRADHNLQDIRAKIFPFKRRKVKAPEIIPSIALPAKRKERSLSSLVVSTSKVPIQTGLTGRRSKAGARKAAALRGCSFTVEESKNEDSAKDNLSSPGSPVKSIQKRRLDSSVAEPSTEQRPNDDGEDDDVQMIEGKADLWTPLNCLVEAANRTKSSNDIYMAKTKNEHGQGIRVLDDKNGTNSLPVSVKRRRLTAAQKRAAMSEGLGASAQAMVDAAAAKSNRRNGPIWFSLVASEDQKRDASLPQISACYLRIQDGKMPVSFIQKYLVKKLDLGSETEVEIMCRGQPVSPTLRLQNLVNLWFCTGSTSKKVSASVGSSAKDFVMVLSYCRKVQES

>BdBMI1a Bradi1g05390.1 *Brachypodium distachyon*

MQDAVLACAALTAAADVAPASAAGGGGGGGGDGGVVRVKRSALVACLTCPLCRRLLRDAATITECLHTFCRKCISEEFINKEVCRCPTCNIDLGCAPEEKLRVDHSLLYVRSKIFPYKRRKVKDQEVISPITSPVKRKERSLSSLTGHGPRVSIQKCLTKRRTKASCLRRFSLGSSKDATKKVGGWRPLGCQLRVGKDRKSLKSDSEDVNRSRTKSGDPDDGAPSNQAKAREHLKRYGNLAKKTGSRKVFTLKGKKKRFKANHPDKMRRLRALWFHLVAAFDQKGQPLPQLPTKFLRIKDVELPASYIHKYLAHKLNLSSEAEVEMVCGGKKVDPGMTLHDLADCWLDKGPKGRVRSSLDSPATGFVTTLFYSRPELLPAPTIPS

>BdBMI1b Bradi1g14270.1 *Brachypodium distachyon*

MQPAPASPEGNPPPPLEGAAPAEEPANDNADAAAVKEAPAAVLEDEEAKEQEEDGDGRREEEEPRRAGRGRKRGRRGGGGGAARGVVMVRRELLARCMTCPLCNRLLRDATTISECLHTFCRRCIYQKFNDEEVESCPVCKIDLGCTPVEKLRADHSLHDVRSKIFPFKRKKIKAEDVATPISLPSKRKERSISSLVVPTPKLAPTGLTGRRTRAVTRKAAAALRGLGPNSGNPVKKENDISDKHAHSSSLPANLGKAPKTRRQISSNAEASNHSSNKDAEDSDMADNAELWRPLNCLVEAANRTKSFRSSLHSPVIKREQINESPNSTYGNKTKSREHLQKPKIEDNKKNAPVSPVIVKRKPGTGRRRRALRIPADGMPEGEVTPNEKRFNSIWFSLVASFEQKGDPPLPQIPSHYLRIKDANVPASSIQKYLMQKLSLPSESEVEINCCGQLVNPTQPLRNLVELWVRGRSTQTTQAMIGSSAEEFVMVLTYGRPKAIAP

>BdBMI1c Bradi4g02647.1 *Brachypodium distachyon*

MQSGSASPGKAPVPPEDLPPPAAAGKEAMAEARGEEVKEDGAKTDAERGGARPRGRPRGRPRRQRPAAEESESGVVKVKKENLAPRITCPLCQRYLREATTICECLHTFCRKCIYKKLAVEELNHCPVCKIDLGCAPAEKLRADHNLQALRSKLIPVKGKKINAEVESPVTAPVKIKERSISSLVVDPPRLTTSLTGRRTRAVTRKAAAALRGLGPILDPVGKDNDNTNKHADNISLLDSLSKVPQTRRKASQAETSSHHSSKDKADHGKDLDKAELWKPLNCLVDAASKTKSFRSSPQSSAVKGAPSNESPSNGHVSREKSGEHLRKSIFQGDKKDTPLSEMMLKRKGPGRGRPATSVAATTQKVPDVRALNSIWFSLIASFEQKGFPQLAQIPTHYLRINDGSIPASSIQRYIMQKLSLQSESEVELTCCGQSVNPAQPVRNLIERWLRFGPSRPLQTVVGSSGGEYVMVISYGRPK

>BdBMI1d Bradi1g09140.1 *Brachypodium distachyon*

MGSRRGEICVRKGLLAGPLTCPLCRGLLRDAHAFTECVHTFCRECIMKKIDDEEIESCPVCNIYLGIAPEEKLRPDNNIQALRKRLFPLKRAEFDASNFPTVTSPLKGKQRSLSSLVVETPKVAAQPVLTGKRTKATRTTFRATSSLSNNGTVKLLITEGHDHETGKISASKSTKMTTSAIKKQIKSDIVASSQPCPQDRKNSKTMDMEELCKPLSSFLEASGTKSLRSNLKSHAAAAKEDKIKSTNGKVTITETRVREASREKSLKLGPKSHAAATKEDKIKSTKSEQVANTGTSVGSHSNKLTLRKEKNGDSDNLWKSPEGDYGQVLLGSTSTVYLHDGITTPVWFSLVTSPHQSEKLLPQIPNAYLRIKDGTLQVFSVQRYIMQKLELASDDEVEILCHGMPICPLMMLKDLLELWLSRQPQHEVQVPVGAPAKQFVMVLGYRRRS

>OsBMI1a Os03g43360 *Oryza sativa*

MQPAPASPPKADGGEDEEEECSRAVVKEEPHHQQEEDDDDAAAAADGGEDEKEKVEEEEVEERGRRRRGRPGRKRGRRSGGGGGSAAAAAAARGGVVMVKRELLARCMTCPLCGRLLRDATTVSECLHTFCRKCIYEKLNDEEVESCPVCKIDLGCTPVEKLRADHNLQDVRSKIFPFKRKKISADEVAAPVLLPSKRKERSISSLVVDTPTVTPTGLTGRRTRAVTRKAAALRGLGPGIDDPVKKEIDNGEKHAQNSSLPTNLGKVPQTRRQMSSNAEASNHSSNKDTEGDRKDLADKTDELWRPLNCLVEAANRTKSSRSSSQSPFVKREQLSDSPGSTSVNKTKSREYMQKSKIEDDKKDVPLLKRKNQRTGRRRELHAQSDSKPEAAATQNEKKFSSIWFSLVASFEQEGDPPLPQIPSHYLRIKDGNIPASSIQKYLMQKLGLPNEAEVEINCCGQPVNPTQPLCNLVEVWLRGRSTQTTQTMIGSPAKEFVMVLTYGRPKAITP

>OsBMI1b Os03g58390 *Oryza sativa*

MQGATPGDAAGEEVGGGGDVVMVRRASVAACLTCPLCGRLLRDATTISECLHTFCRKCIHEEFVDKESCCCPTCNIDLGCAPLEKLRVDHSMQFVRSRIFPFKRRKVENPEIICPVASPVKRKERSLSSLTIPAPQVSIQKCLTKRRTKASCLRNFPLHSTSRGSKDTSKKLGGWRPLGCQLKLGKDKKSLKSSVKDTNRTKSKSGDTDDGAPASKAKAREPFTRYGRAAKRTGRKKLLMLKNKKKRFKAKQPSKKRRFRALWFYLLAAFDQRGVPTLPQLPAKYLRIKDVDLPASIIQKYLAQKLNLSSETEVEVLCGGKVVNQGMTLHDLADCWLEKGPKSRMRSSVGSPATGFMVTLFYRRPDVDVSSSPAPPQPDTESCHS

>OsBMI1c Os03g53080 *Oryza sativa*

MGAARGGVRRVRREALVACMTCPLCKGLLREATAITECLHTFCKECIMEKIDDEEVDHCPVCNIDLGCDPEEKLRPDHNVQDIRNKVFPLKVKKVGAPKAPTVTLPVKRKQRSLSSLVVDTPRVAVQTGLTGRRTKTARRTAVSHVNSPGNNGTIKLANKSEGRDHKTQKISAAQSAKMTKTGNKKKNNTDVDVTIQSSSEDRKDDHTIDKEDLKKPLNSLVDTANRTKFFRSGPKGQAAKEDKIKNSIKLLAEDDTEDKLVVTGRKVMPCSNKLKVKEENNRSPSQSASSKDKTTSDYELRKGQHADSQQGQIGSTRTGALHDGITRPVWFLLVPSPDQKQDPRLPQLPTYYVRIKDGSLQTSLIQRYIMNKLDLASEDEVEITCHGEAISPSTTLQGLLELWLKSSPVEQVQASLGAQAKEFVMELGYRRPQRPPSS

>ZmBMI1a GRMZM2G457544 *Zea mays*

MESAVLACEAPTAAAAAAPGAGGDRGAEGVVMLRRSGLAACLTCPLCGRLLRDAATITECLHTFCRKCISEEFVNKEVCCCPVCSIDLGCAPLEKLRIDHSLQYVRSKVFPSKKRKVEDPEVTSPITSPIKTKVRSLSSLTKHAPQMSMQKCLRKRRTKASCLQNLPLHSTLRGSSNITKKSGGWRPLGCHFRAAKKKRSRPKSDDIKTAGKRTDDPVDVTLASQAKTRKQITRRGNLEKRTGTKKVLILKGKQKNTKTKNTPNKRRLPALWFYLLAAFDQKGHPPLPQIPSKFLRIKDVNLPASFIQKYLAQKLNLSSETEVEIFCGGRPVSPGITLHDLADHWIGKRSKGRVRSSVGTPAAEFVLKVFYARSGAPLPKTESNQD

>ZmBMI1b GRMZM2G141379 *Zea mays*

MQPGPTSPPHESPSPPHEVTPQEEPATLPLPGEEAQPQGQADDGDAVKAAAGAREDRNDRSGATASARGRKRRRRGGTSSTSPSPFPSPAAAAGTTRGVVMVKRDLLARCMTCPLCRRLLRDATTISECLHTFCRKCIYQKFDDEEVECCPVCKIDLGCTPTEKLRADHSLQDVRSKLFPFKRKKINAEEVSSSISLPTKIKERSISSLVVDTPKVKPTGLTGRRTRAVARKAAAVATATVTLHELRPVIEDPVKKEIDSCDSHPHDSNLPASSSKAPQIRRQISPNVEASNRSSNKDTEGDSNDELADKSELWRPLNCLVEAANRTKSFRSSSQSPVVKGEQPNGSTSSTFASKAKARENLEKSKIQDDKKDVPMPPVLPKRRAQGTARKKRDRLQAPTDVKPDAAVAQNAKKFSSIWFSLIASFDQQGDPPLPQIPSHYLRIKDGNIPASSILKYLMQKLSLPSESEVEIKCCEQPVNPSQPLRNLVELWLKGRATQT

TQTMTGSSAKEFVMVLTYGRPKAPAM

>ZmBMI1c GRMZM2G180195 *Zea mays*

MQTTGPASPDKPPVVDPEADDKVEEGARAKEDDAAKVSGEDEDEEEQEGRRGRRQRRRRGGAAGDSSVAMVKRELLARCLTCPLCDRLLRKATTISECLHTFCRNCIYNKINDEDLDHCPVCKIDLGCTPVDKLRADHNIQDVRSKVFPFKRKKVNAEEAESPIMLPVKVKERSISSLVVNTPRVTPAASTGRRTRVVTRKAAALRGLGPIIVDPLKKDNDNSNKQTDNSSLLDSLSKIPQTRRQLLSNGDTSSHPSVKDKADDNKDFDKSELWKPLNCLVEAASKTKPLTSAQSPALKGEFRESPSSEHFSRTKVKVEDNKSDDPEPIVLLRKRGRPGRKRKNPLPETNAASTATAIQARKAFSPIWFSLIASFDQKGNPPLPQIPAHYLRIKDGSIPASSIQKYIMQKLSLVSESEVEISCCGQTVGNLGQPVRNLVERWLRVGPARPLHTVIGSSGGDYVMVISYGRPKSASS

>ZmBMI1d GRMZM2G116574 *Zea mays*

MQTSETTGTASPPAVEPEAEDKVEEGARANEEAAAKVGGDDEDDWESEHEGRRGRRQRCRRGGAAGDSAVAMVKRELLTRCLTCPLCDHLLRQATTISECLHTFCRKCIYKKLNDEDLDHCPVCKIDLGCTPVEKLRADHNIQDVRSKFFPFKRKKVNAEEAESPIMLPVKVKERSISSLVVNTPRVTPAALTGRRTRAVTRKAAALRGLGPIIVDPLKKDNDNPNKQTDNSSLLDSLNKIPQTRRQLLSNGDTTSHLSVKDKAGDNKDLDKSEFWKPLNCLVEAASKTKPLTSAPRPALKGDKPRKSPSSDSEHSSRTKTREPLQKSKAEVDKSDPGPIVMLRKRGRPGRKRKNPLPETNADSTATAIQARKALSPIWFSLIASFDQKGVPPLPQIPAHYLRIKDGSIPASSIQKYIVQKLSLLSESEVEISCCGQTVNPGQPVRNLVERWLRVGPARPLHTVIGSSGGDYVMVISYGRPKSASC

>ZmBMI1e GRMZM2G018798 *Zea mays*

MGAARGLARVRREALAACMTCPLCRGLLREATAITVCLHTFCRECILEKINDEEIDCCPVCNIDLGCDPEEKLRPDHNLQDIRNKVFPIKKINVDSPKALTTLPAKRKQRSISSLVVDTPSVVKQTGLTGKRTKAKRRAAASLATSPVNNGTMKLPSKSENGDQKTDKSCPSQSTKAATSANKTENQDQKKTKKISAKQSTRAATPAKKKQRNTDAQVSSKPSSEKRKNSKTADKDGLRKSSKVPRSTPKIHAVNEEQIKEKEGEPPTRKGEADNKVVIPGTSVREHSNNSNLKEKNDGTSPEPSPLKDKTATEDSYRGSLGSAKGLHGPVTTPVWFSLISLPNQKEDPQLPQLPKTYMRIKDGSLQISSIQRYIMKKLDLANENEVEIICHGEPICPSSTLRGLMELWHRRQPTEPVEASVGAPAEEFVMVLGYRCHRRHPYLAPGTTVAVPPELPS

>ZmBMI1f GRMZM2G147809 *Zea mays*

MRAARRLARVRREALAACMTCPLCRGLLREATAVALCLHTFCRDCIVEKINDDDADCCPVCNIDLGCDPEEKLRPDHNLQDIRNKVFPIKKINADSPKSLTTLPAKRKQRSLSSLVVDTPRSVVKRTGLTGKRTKAKRKTVASSAASPINNGTMQLPTKFENRDQKTDKSCASQSTNVASAANKTENQDLKKTRKTSAKQSTRAATSANRKQQNTDVDVSSKPSSENRKNGTTADKDELRKYSKIPRSTPKIHAVNEEQIKKKEDELPTRKAEADNKVVIPGTTVREHSNNSNLKEKDNGSSPESSPLKDKTTADDDSYQCLLGSASDVRDPITTPVWFLLISLPNQKEDPQLPQLSKSYMRIKDRSLQISSVQRYIMKKLDLANENEVEIICHGEPICPSSTLHGLMELWHRRQPAEPVEALVGAPAKEFVIELGFRRRRHPYLAPGTVAVPPEPS

>SmBMI1a 438486 *Selaginella moellendorffii*

MEVVKVARAPLVACLTCPLCSSLLDEATTICECLHSFCRECIHLKLSEDDTQSCPICDVYLGVAPLEKLRPDPQLDELRNKLFSPGAPRVRVPGDASPLPRLKRKERSLSSLGVFSAPSSSRRRKPSRPQQQHYTRVKREITSSSEEDEEHKSESSDSGNHAKKPTREDKGKSVAAGYHSPLAYLAELADSDHAGGSSRQQNQPSSSVGRDIKRQATTGRFSKLSRPTKGVPRRRGGTSANNGGELYKATLPPRFQVVTADNGVWFALKPAANQSDDSVLPAITTPYVRITDGRLPVSLVKKYIATKLNLGNETDVELNCRGQPIVSSLPVENVQRIWLAKPRDDQTPSKLDTSLELLMTLTYKRPGGNPTNATAAAT

>SmBMI1b 440578 *Selaginella moellendorffii*

MPYSSKEPCETMARVSKAPIVACLTCPLCGNLFREATTISECLHTFCKDCIHDRFSDEETNSCPMCNVDLGTVPLEKLRADPQLDDVQAKIFNLNPKKRPAAPVAASPPSHTPARRKERSLSSLGVTSVPSSSRRKSRRKAWVTESSESYEEEEDDDDNSSDAADESPEDTMYCSETLDSDTKSPMRSGSKRQRATRGNSVGNAEAKQSGGSFLSPLACLAELAHADEKGRASSGLMKEARGREASSLRATKNVRRSSQAKAAANGGGGFKCQTLPSPSTQSNGLWFTLTTTNQGEEGLPQIKSPYLRIKDGKVPVSFVKKYLVRKLDLKSESEVEITCKGQPVVSSLPLESVRKIWLTKDQLSSTPEDRASALGKDFLMVLSYGRNRRAAAVS

>PpBMI1a Pp1s127_49V6 Physcomitrella patens

MPYSGRVGVTYKEVRIPKAPLLACLTCPLCNYLIREATTISECLHTFCKACITAELSNGESECCPMCHVGLGTLPLEKLRADHQLNDLKEKLFPSNVKKRKLGLAIGSPGTSNATKRKERSLYSLGVKGTLAVNPGYANRKTRALPGTHGGSEPSSIDEELEDDDDDEDDLSEDEPSTTLKLSYNGGSRRSRSPDEVPEAKVYPHSEPRRRTEDLASTPKADAEHLRGASKSKSFSMSKMGHRLKTLSNGNKTSGSLSGSKEILKNPRGSRKHNGSSNSGGAGRETDAINPMDVLAQVANADAAREDIGSGPSSPSPGLHTTGNGSKPARGQKDVKEGVLAKVGSNHKDTSAANSNGQSLQARLRQTLNGVIERPSIIRNTPRPVSGSPVAGQSSRAPSNGIWFHLESGDLQANEDALPPLSYPYVRIKDGKLPVSMVKKYLVQKFAHKVKTEMEVEITCRGQPVVSSLPLEGIRDIWFSAQVSDQTQTQTQALPPTQSQGEPSTAPANGSGPSEPASAADFLMVLTYKRHRKPRIC

>PpBMI1b Pp1s233_110V6 Physcomitrella patens

MPYSGRKGVTYKEVRIPKAPLLTCLTCPLCNYLIWEATTISECLHTFCKDCIAAELTNGESECCPVCHVGLGTLPLEKLRADHQLNDLKEKLFPSNVKKRKLGLVLGSPETSNATRRKERSLYSLGVKGTPADNPGYANRKTRVLPGNHGSSEPSSIDEDLEDDDDDDVDDLSEDEPSTTLKLSYNGRCTRSRSPDEAPDVKVYPHSEPRGRMEDLASTLTGDAEFLRASISGHFTTSKLTHRLKTLSNCNKKSGSLLGSRESLKNARGSRKNNGSSNSGRADREADAINTLDVLAQVANADAAREDMGSGPPSPTLPYMHTPSSGSKSVPGQRDVREGVLAKVGSNLKDSSPATSNGQSLQVRLRQTLNGVVERPSIIRNTARPVLGTPVSGQSSRTTSTGIWFHLESGDLQANEDALPPLSYPYVRIKDGKLPVSMVKKYLVQKLSHKVKTEMDVEITCRGQPVVSSLPLEGIRDIWFSAQVSDQAQTLLSTQAQGEASTTPANGSGPLEPPSATDFLMVLTYRRHRQLRIC

>VcBMI Vocar20006591m.g *Volvox carteri*

MGSQTQAIQELTQNAETAEGRHDCKRRRRGHERLFLSSGPLGSGAAGVPQEHSLDVSQPDWWQPQPRATLDPDDYQDRPRTSLVACLSCALCHQLLQEAIASLECGHTYCYDCIEARVDIGGNHNVCPVAGCGVVLGPSPFDHHRLVYDTLLDSLVQKIFPRPDLDAALSARRADREEATRLARAELVYLLVVFALAELLYSYFSPPIMARAMFM

>HsBMI1 Homo sapiens

MHRTTRIKITELNPHLMCVLCGGYFIDATTIIECLHSFCKTCIVRYLETSKYCPICDVQVHKTRPLLNIRSDKTLQDIVYKLVPGLFKNEMKRRRDFYAAHPSADAANGSNEDRGEVADEDKRIITDDEIISLSIEFFDQNRLDRKVNKDKEKSKEEVNDKRYLRCPAAMTVMHLRKFLRSKMDIPNTFQIDVMYEEEPLKDYYTLMDIAIYTWRRNGPLPLKYRVRPTCKRMKISHQRDGLTNAGELESDSGSDKANSPAGGIPSTSSCLPSPSTPVQSPHPQFPHIS STMNGTSNSP SGNHQSSFAN RPRKSSVNGS SATSSG

**Core component: LHP1**

>AtLHP1 AT5G17690 *Arabidopsis thaliana*

MKGASGAVKKKPQVLNEAGEAETAVETVGESRKISGDGGFGSDDGGGGGGGGSGESILREIGDDRPTEDGDEEEEEDEDEDDGGDEEDEEGEGEGGQEERPKLDEGFYEIEAIRRKRVRKGKVQYLIKWRGWPETANTWEPLENLQSIADVIDAFEGSLKPGKPGRKRKRKYAGPHSQMKKKQRLTSTSHDATEKSDSSTSLNNSSLPDIPDPLDLSGSSLLNRDVEAKNAYVSNQVEANSGSVGMARQVRLIDNEKEYDPTLNELRGPVNNSNGAGCSQGGGIGSEGDNVRPNGLLKVYPKELDKNSRFIGAKRRKSGSVKRFKQDGSTSNNHTAPTDQNLTPDLTTLDSFGRIARMGNEYPGVMENCNLSQKTKIEELDITKILKPMSFTASVSDNVQEVLVTFLALRSDGKEALVDNRFLKAHNPHLLIEFYEQHLKYNRTP

>BsLHP1 Bostr.26527s0439.1 *Boechera stricta*

MKGASVKKKTQVLNEAGEAETAVETVGESRKISGDGGFVSDDGGGGGSGESILREMGDDRRTEDGEEDEEEDEDEDDGGDEEDEGEGEGEGGQEERPKLDEGYFEIEAIRRKRVRKGKVQYLIKWRGWPETANTWEPLENLQSIADVIDAFEGSLRPGKPGRKRKRKYGGPHSQMKKKQQRLTSTSHDAAEKSDSSTSLNNSSLPDIPGPLDLSGSSLLKGDGEAKNAYASSQVEANSGSVGMVRQVRLIEDEKEYDPTLNELRGPVNNSNGAGCSQGGGIGSEGDNVRPNGLLKVYPKELDKNSRFIGAKRRKSGSVKRFKQDGSTSNNHTTPTDQNLTPDLTTLDSFGRIARIGNEYPGVMENNNLSQKTKIELDITKILKPMSFSASVSDNVQDVLVTFLALRSDGKEVMVDNRFLKAHNPHLLIEFYEQHLKYNRTS

>BrLHP1a Bra013958 *Brassica rapa*

MKGAGVKKKTQVVNDAGEAETAVETAGGSGKRSGDGGFGSDDGGGGDSVLREMGDDRRTEDEEEEEDDEDEEDEEDGGGGGGGKEERPKLDEGFFEIEAIRRKRVRKGKVQYLIKWRGWPETANTWEPKENLQSIADVIDAFEGSLKPGKPGRKPGRKRKHHGGSNSNTQLKKKQQRLTSTTSHDASERSDSFTSLNNSSLPNIRGPLNDDLCGSGDGEAAYAAANQVEANSSRRSVGMVGEEKDYDPTLSELRGPVVNSNGAAGCSQGGGGGIDNVRPNGLLKVYPKNSCGVIGAKRRKSGSVKRFKQDASTSNNNNSNNHTTAATDQNVTQELATLDSFGGVARIGNEYPGVLENNNLSQKSKVEELDIAKILKPVKFSSSVTNNVQDVLVTFLALRSDGEEVMVDNRFLKAHNPLLLIEFYEQHLKYNPER

>BrLHP1b Bra023629 *Brassica rapa*

MKGASVKKKTHVVNDAGEAETVGGSGKRSGGDGSEDGASILGEMGDDDMRTEDGEEEEEDEEEGGEEEAKEERSKLDEGYFEIEAIRRKRVRKGKVQYLIKWRGWPETANTWEPLENLHSIADVIDSFEGSLRPGKPGRKKKRKYAGPYSQLKKKQQRFGYDAAEKSESSTSLNNSSLPGIRGPLDLSGYVATTNQGEANSGSVGMVRRVSLKEYDPTLNELRGPGIGCEGDSVRANGFLKEFDKNSGFIGAKRRKSGSVKRFKQDGTTSNNNNNHTTDQNLTPDLDSFGRMVEPHHNNNLSQKSKAEELDIVRIIKPVRFSSSITNNVQDALVTFSALRSDGKEVTVDNRFLKAHNPLLLIEFYEQHLKYNPER

>BrLHP1c Bra006417 *Brassica rapa*

MSDTPLDSNTQNMEGAGVKNDAGEADTAVETVGGTGDEGGRGGTIPGEIGDDRRTEDVEEKEDEEEEEEGKEDGSKVAEEFYEVEAIRHKRVHKGEVQYLIKWKGWPETDNTWEPLENLQSLSASIDAFEKRLKKADKKRKSQYGGSNSQSKKKQQQPLTSTPPDASERTDPTLNELRGPPVINSDGAGSSQEGGEGIGSEGDNARTNGLLEGKRKEKSGVRGAKRRKSIAKKYTIPDETTTSNNQPTTATDQNETPNLDIVRIIKPVEVITSITNNVQDSLVTFSVQRADGEEVTVDNKFLRAHNHHLLLDFYEQHITYNSES

>CgLHP1 Cagra.6254s0015.1 *Capsella grandiflora*

MKGASLKKKTQVLNEAGDAETAVEAVGETRKISGDGGFVSDDGGGGVGAGAGAGGGSGESILREMGDDRRTEDGDEDEDEDEDEDDGGDEEDEGEGQGQGEREGQGGQEERQKLDDGYFEIEAIRRKRVRKGKVQYLIKWRGWPETANTWEPLENLQSIADVIDAFEGSLRPGKPGRKRKRKYGGPHSQMKKKQRLTSTSHEAAEKSDSSTSLNNSLPDIPGPLDLSGSSLLKEDGEAKNAYASSQVEANSGSVGMVRQVPLIEDEKEYDPTLNELRGPVNNSNGAGCSQGVGIGSEGDNVRPNGLLKVYPKELDKNSRFIGAKRRKSGSVKRFKQDGSTSNNHTTPTDQNLTPDLTTLDSFGRIARLGNEYPGVMENNNLSQKTKIEELDITKILKPMSFSASVSDNVQDVLVTFLALRSDGKEVMVDNRFLKAHNPHLLIEFYEQHLKYNRTS

>OrLHP1 orange1.1g042818m *Citrus sinensis*

MKVKGGGKRKVVNPALNDAEEANDDIENQENINYDSIDLIDVEYQENQEQEKENEKEKEKGKEKVGEEKNNEEEEEEEEEEEEGEDEDEDAVPQEERPKLDEGFFEIEAIRRKRVRKGQLQYLIKWRGWPENANTWEPLENLQSCSDVIDAFEESLRSGKSSRKRKRKGGGSSSLPKKKQARTFSAPYYVTGGVGQSLPADPLINAGLIDLSPSTQSIGSGHVGGNVGNVNNLRTAKQTNDNRLANGSKQIDGRNEEAEYDPKLSELKGMISNNEANADKLALHFQEARVSEGNGLTNGLSKADQVEPLHSNRRTGARRRKPSSVKRFKQDLASTKVIVTQDSTPGIAVGCDSADEQLGIGNSSHKSKHEGPINASAIVKILKPIDFSASVSDNMQDVVVTFMAVRSDGKEVMVDNKYLKANNPLLLINFYEQHLKYSTQ

>CsLHP1a Cucsa.362610.1 *Cucumis sativus*

MKFKGGVGRKKSSSPSMEVAVGSIQDAMDSGEVHGGNSDYANVNINNKPINGSEPSNSHLTETHQDQNLEEVDDDDGEGDEEDEQDGDEAAFASQRTNLDDGFYEIEAIRRKRVRKGQLQYLIKWRGWPETANTWEPLENLHTCSDFIEAFEQSLMTGKQRKRKRKHGVVHTQTKKRQHQQRGSFSAYNVTDVEISVVDQRLPSAPLNMSSLTNPHAHSQSLVYNHEGEKNGDVTAIERGKQTDIDNMGRKATQRSEWKKDEHEYDPKLSELKATVLTNIAITDKHVINFQDSRATENNGSAAGLSKGAFVEPVTDNRCTGARRRKSGSVRRFRHDSTLSALPRSQNAELTLAVVESGARVEPIGVENSGYHGESLSRNNKTDDARNEMSITKIIKPLGYSASVSNNMQDVLVTFVAMRSDGTEVVVDNKFLKAINPLLLINFYEQHLRYTTRS

>CsLHP1b Cucsa.257460.1 *Cucumis sativus*

MGRGKKKAAGSSEPETVALPIPDFTQSTHLNGDSAPSISNNNGSEPKILFPHSPSSLHNASVQIPLPIDDAGVVNGEDNVIPDVSASERTNLDEGFFEVEAIRRKRVRKGQLQYLVKWRGWPETENTWEPLDNLQSCFEFIEEYEERFCQSRSGKQRKRKRKDEDIESESQEEKDLQIIAIDNVTDVVISTLDDRLSAAPFNKKLHRDLPISQEPLDSIHEGELDGKFDGSRKKDEYDLKLIDFNASISGNMVDSEKKTVASNDVSLVYDVSKADCVVGSAQVSHSTGAKRRKSSRVKRFTKDSALPEQGLKQNAATVSIEPIDPSEQLGPQNPSSSGHSRNVSTITRIIRPVGYSVSVLNNIPDVIVTFLAVRSDGKEVTVNNKFLKANNPHLLINYYEQHLRYNPTL

>EsLHP1 Thhalv10013571m *Eutrema salsugineum*

MKGAGVKKKTQVVNDDGEADTAVEIAGDSRKISGDGGFGSEDGGGGGGGSGESILREMGDDRRTEDGEEEEEDDEDEDDGGEDEEEEERGDEGKEDRLKLDEGYFEIEAIRRKRVRKGKVQYLIKWRGWPETANTWEPFENLQSIADVIDAFEGSLRPGKPGRKRKRKYGGPNSQMKKKQHRVASTSHDAAEKSESSTSRNNSSLPNIPGSVDLSGSSLLKGDREAKNAYVSNQVEANSGSVGMVRQVSLVEEEKEYDPTLNELRGPVVNSNGAGCPQGGGIGSEGDNVRPNGLLKVYPKELDKNRGAKRRKSGSVKRFKQDASTSNNHTTTTDQDVTPELATLDSFGRIARIGNEYPGVLENNSLSQKSKTEELAIIKILKPVKFSASVTDNVQDVLVTFSALRSDGKEVMVDNRFLKAHNPLLLIDFYEQHLKYNRT

>FvLHP1 mrna03113 *Fragaria vesca*

MSSVELVESVVAKKYEFGEFLKLKIVSSQLHFSLCRIGGSVPSGLTQTQAHRERKHYSHLAVRFGGVTGASLRMRVKGGGRKRSWEAMPLDDGGGCLLFEDTADPNSAVFSVPEHQDTEPEPQIEPESQNQEEAEGVEGEDGDGEGEGEGEDGDGEEGEEGEAEAEGEEDENKERSTKLDEGFYEIEAIRRKRVRKGQLQYLIKWRGWPETANTWEPLENLQSVSDVIDAFEESLRTGKQRKRKRKSGTPHTQPKKKQQRSSDTPYNETDGEISMVDKALSSSAPNGTGVDGIPPPQQSFHSVGDGENNGHVNHTETSKKTDSENFCANVSHQISERREENEYDPKLSELKATVSTNNVSLDKLSVHFQEAKAPEVNGPTNGLSKVDCGEPVQGSRSTGAKRRKSGSVKRFKQEPHLSELGATQNAPTRVSTRYAGRVDQTGLGNLDYAGENSGRRNKIDEFKEAVRITKILKPMGYSTSISNNIRDVSVTFMAMRSDGTEVIVDNKFLKVNHPLLSCRVIQGVNGECNLSLARCLSSLSSAAQCWILVVAGESA

>GrLHP1 Gorai.007G347200.1 *Gossypium raimondii*

MKGGRKRVSQSEGGGRGGSVKEKKKGGTEEEIVETEGLGEKKEEEEEQEEEEDDDDEDDEEEEEEEEEEGKEEENGERKEDERTKLDDGFFEIEAIRRKRVRKGQLQYLIKWRGWPETANTWEPLENLQSCSDVIDAFEESLRSGKHSRKRKRKYGGPHTQSKKKQPCSSGSTYNVTGLELGGVDKTPLVPFDNSGIADLSASSTVIVLAREGERNGNSSNVRRAKRVKDNSSANGSKKIDETKDENDYDTKLSELKGALSSKGGNTDKLAIRFQEGKASEGDGPVNGLQKVDLGESVQSDRRTGAKRRKSGSVKRFTQDPASSGPNLTQNATNVHVGYAITDAEMEIVNLGLAADGSSHRSPIDNSLNVPVITKILKPVGFSASVSANTQDVSVTFLALRSDGKEVMVDNQYLKANNPLLLISFYEQHLKYSPPS

>PtLHP1a Potri.013G070400 *Populus trichocarpa*

MDRKKNTRSRQKDLCFVVRSVPSPTFTFIFTHTHTHSGRYRTLPSIFPSQTPNTKSPFSPPFPSKKDQTFLKKMKGKRKATANPVLADTAEGSSNSLIETQVREGNIGEEKEINWVDNGENREETEGEEEEEEDNDDEEEEEEDPKGKDEKGNLFDEERTKLDEGFFEIEAIRRKRVRKGQLQYLIKWRGWPETANTWEPLENLQSCSDVIDAFEESLRSGRSSRKRKRKHGAPHTPSKKKQPRSSAVYNVMDVEVSIADKHLPSAPLNNSLLADLPSPSQFIGLGHGGESSGDVNNIKTSKQTDENGSINGSKHIFERKEDNEYDPKLSELIGTIPNIDVNTNKFTIHFQEEKASEDNGIANGLPKVDYVDLVQNSRCTGAKKRKSGSVKRFKKDSVMCEPVFLPNSSGNFSVGSTGAAAQPGIENPSLTWGNSSHMPMTGNSINAFAITKILKPIGFSASVFDNVQDVLITFRALRSDGQEVTVDNKFLKANNPHLLINFYEQHLKYST

>PtLHP1b Potri.019G044400 *Populus trichocarpa*

MLNGFGQKLPSLNPRVSSVKSYQVLLTGLVSISVSLTRTHLVLVRVVGLGLCHLSTAVYIYLIVYINTDNQTQTPKQKKITFLPSKKNQTFLNTMKGKRKATANPVLTDAAEGSSSNLFETQVREGNVDEEEERIWAYNGENREETEGEEEEDDDDEEEEEEEEDSKGEQERDNLFDEERTKLDEGFFEIEAIRRKRVRKGQLQYLIKWRGWPETANTWEPLENLQSCADVIDAFEESLQSGKSFRKRKRKHGGPHTQSKKKQSRSSTSYNVLDVEISIADKHLPSAPLSSSLLGDLPSPLQFISSDPGGESNGEANNVKTFKQIDENGSMNGSKHIFPRKEDNEYDPKLSELRGTIPNIDVNTNNLAIHFQEEKTLEGNGLANGLPKVDYDPVQNSQRTGARKRKSGSVKRFKKDSVKPAFLHNSSANFSVGSTGGVAQLGIENPSLTWGNSSHMPTAENTINALAITKILKPTGFSASVSDNVQDVLITFRALRSDGQEVTVDNRFLKANNPHLLINFYEQHLKYST

>BdLHP1 3g24710 *Brachypodium distachyon*

MARSKRDSPVVVGSGDPVEDEEEMVAVATEVDEEEKGEEGEEEEWKEEEDDEEEEEEEEEEEEEWVQEEEEEEEEEADEAPAEDSVQAAAAEQGSPPVLAEGYYEIETIRRRRLRKGQLQYLVKWRGWPESANTWEPFENLKACSDFVDAFEKRQQNGKRKRKAMTTPVIGPNPSQGKRGRPRRSDPRSLPRTPAPVPKRLPLRTSSRRATNNSNKNSVGGHDASVSVVGQQMLRQNVTRESNSNILSVGSASQRAPLSVVVDQQDEPLVVNHDLSKVENSVQAPPSQAGQVTGAKKRKSGSVRRFKQDEATREQGELHNGKREKPGNEDVDSTEGETGDRNKGEDCANRIHITKIIKPVRYFATMTNDVQQVSITFKALRSDGQEVLVDDKELKANNPLVLINYYEQHLRYNPTS

>OsLHP1 Os10g17770 *Oryza sativa*

MARGKNHPDGEEEEAPAAAGEEEAPVEMDEEGEMEEEEEEEQGEGEGEERDEGEEEEEWEDAEEVEGGEESEQAAAEEEDSPLVVVEAEAAAPVVDGSPPKLAEGYYEIEDIRRRRLRKGKLQYLVKWRGWPESANTWEPLENLSACSDIIDAFEMRLQSPRPGRKRKRKITTTPVAGSNPSHGKRGRPRLDAKSHTRAPAPEPKQLPCRTSCRRATNCSSKTVAGLDASGSVVRNQLAQNIVQEGSSSVISRTPCQELPLSIRLTDQQNEHHLVNGSSNSENLVKVPPSQGGQVTGAKKRKSGNVRRFEQNKPTQGQGECGALVVAEDVGSTEGETGDKKKTEGCPNRVHITKIIKPVRFAAAVNNDVQQVSITFKALRSDGQEVMVDDKELKANNPLLLISYYEQCLRYNPTS

>ZmLHP1 GRMZM2G117100 *Zea mays*

MRRNRKDSGYPTAAPGEEDEAEELTEEGDEEEEEQTEEGCGEEDEVEAETPAQEAVEPPKLAEGYFEIEAIRRRRLRKGQLQYLVKWRGWPESANTWEPLENLKACSDIVDAFNKRSRSPRSCGKRKRKTPTTPTSDPNPSRGKRGRPPRSEARSMHSIHASEVKKLPCRTSSRRANNNSNKTSPGGLGAAVNLLGQRIVQEGSSGVVSVGFPSQEAPLSVSLTDQQDAQHPANGSLKMDNEIRAITPQGGQVTGAKKRKSGCVRRFKQDEVTHEQGDIRDRTSDKPGNETVDSTEGDPGDKNKGEDSGNQIHMPKIIKIIKPVRYFATVMDGVQQVAITFKALRSDGEMVFVDDKQLKAKEPLVLIDYYEQHLRYNPTS

>SmLHP1 88036 *Selaginella moellendorffii*

mekrkrksvsfyssqqwdkqrakrsrndddefyevesiqqmrlfegklhyyikwkgypeefntwephkhlancpqalkdfersqqalvaaevkaevkaevegekalppllaakphqdlgspslqstvanhavekilkamsystrelsngekdlnvtfkv lrsdgeeill dnkylrenyp qllldfyeqr vrnvrq

>PpLHP1 Pp1s220_98V6 *Physcomitrella patens*

MGKPRSAEQNAIEHGGDSAPVGGARLRPMQEVIRNSPRLKHKGRNKSGSFMADPPSKAMANRTKDRWKSQTPKSKSSGKVEKNIREKNVHDEDSEESNIDDHQNEENEDEDEDEDDSDELEEEQEEVLGEGMFEVEAIRKKRIRKGKKEFLIKWRNWPEKDNTWEPFSHVAKCRDILEEFEASQKARRGKRKFGTYTLDGAGKRKRSFMSNADEDAPSQSEGKDTVKSSTDSYAVPEEKERKTRHQAGLTERTASPSAEAARTLLDLPDEAPTTSKLAATPEVSKVIGDIVAGEENGADSLGRAVVIYETPRAAEVVEPDGGVVDGIPSVDTKSLGLNGTNSWADAAVPRFAYDDNTKFPSSMSQLITTRENEVLENKIGAHPAVADESRGLTEEKQLSKAVKLVEFGHSDEEMDIRNLGLVHDTVQGVTGSSKSEGGPVGSKRRKGAVPRRVPQDQDGVQTSLEDIKRELGQGLEEYHTTADKVSGRELATVARKDIPPEHKECQSAFQDNLSRKALSTPPPVEKKEIGALTTPVITHILKAISYSNSITHEKQEVLVQFKASRSDGEEVVVDNKFMRESYPLLLIDFYEQHLRYSNA

>HsCbx2

meelssvgeqvfaaecilskrlrkgkleylvkwrgwsskhnswepeenildprlllafqkkehekevqnrkrgkrprgrprkltamsscsrrsklkepdapsksksssssssstssssssdeeddsdldakrgprgrethpvpqkkaqilvakpelkdpirkkrgrkplppeqkatrrpvslakvlktarkdlgapasklppplsapvaglaalkahakeacggpsamatpenlaslmkgmasspgrggiswqssivhymnrmtqsqaqaasrlalkaqa tnkcglgldlkvrtqkgelgmsppgskipkapsggaveqkvgntggpphthgasrvpagc pgpqpaptqe lslqvldlqsvkngmpgvgllarhatatkgvpatnpapgkgtgsgligasgatmptdtskseklasravapptpaskrdcvkgsatpsgqesrtapgearkaatlpemsageessssdsdpdsasppstgqnpsvsvqtsqdwkptrsli ehvfvtdvta nlitvtvkes ptsvgffnlr hy

**Core component: EMF1**

>AtEMF1 AT5G11530.1 *Arabidopsis thaliana*

MGSSIKINSISIDLAGAANEIDMVKCDHFSMRGFVAETRERDLRKCWPFSEESVSLVDQQSYTLPTLSVPKFRWWHCMSCIKDIDAHGPKDCGLHSNSKAIGNSSVIESKSKFNSLTIIDHEKEKKTDIADNAIEEKVGVNCENDDQTATTFLKKARGRPMGASNVRSKSRKLVSPEQVGNNRSKEKLNKPSMDISSWKEKQNVDQAVTTFGSSEIAGVVEDTPPKATKNHKGIRGLMECDNGSSESINLAMSGLQRRKSRKVRLLSELLGNTKTSGGSNIRKEESALKKESVRGRKRKLLPENNYVSRILSTMGATSENASKSCDSDQGNSESTDSGFDRTPFKGKQRNRRFQVVDEFVPSLPCETSQEGIKEHDADPSKRSTPAHSLFTGNDSVPCPPGTQRTERKLSLPKKKTKKPVIDNGKSTVISFSNGIDGSQVNSHTGPSMNTVSQTRDLLNGKRVGGLFDNRLASDGYFRKYLSQVNDKPITSLHLQDNDYVRSRDAEPNCLRDFSSSSKSSSGGWLRTGVDIVDFRNNNHNTNRSSFSNLKLRYPPSSTEVADLSRVLQKDASGADRKGKTVMVQEHHGAPRSQSHDRKETTTEEQNNDDIPMEIVELMAKNQYERCLPDKEEDVSNKQPSQETAHKSKNALLIDLNETYDNGISLEDNNTSRPPKPCSSNARREEHFPMGRQQNSHDFFPISQPYVPSPFGIFPPTQENRASSIRFSGHNCQWLGNLPTVGNQNPSPSSFRVLRACDTCQSVPNQYREASHPIWPSSMIPPQSQYKPVSLNINQSTNPGTLSQASNNENTWNLNFVAANGKQKCGPNPEFSFGCKHAAGVSSSSSRPIDNFSSESSIPALHLLSLLDPRLRSTTPADQHGNTKFTKRHFPPANQSKEFIELQTGDSSKSAYSTKQIPFDLYSKRFTQEPSRKSFPITPPIGTSSLSFQNASWSPHHQEKKTKRKDTFAPVYNTHEKPVFASSNDQAKFQLLGASNSMMLPLKFHMTDKEKKQKRKAESCNNNASAGPVKNSSGPIVCSVNRNPADFTIPEPGNVYMLTGEHLKVRKRTTFKKKPAVCKQDAMKQTKKPVCPPTQNA

>BoEMF1 Bostr.20055s0085.1 *Boechera stricta*

MGSSIKINSISIDLAGAANEIDMVKCDHFSIRAFVAETRERDHRKCWPFSEESISLVDQQSYSLPSLSVPKFRWWHCISCIKDIDADGTKDCGLHSNSRTIGNSSVFPSKSKLNSLTVIDQEKERKTDIAGNAVEENLDENCERSQKDDQTATTFLKKVRAMDASTVRSKSRKLVSPEQVGNKRSKEKVSKSSMDISSWKEKQVVDQAVTTFGSSEIAGVVEDTPPKATKNHKGIRGLMECDNGSSESINLAMSGLQRRKSRKVRLLSELLGNTKTSGGSNIRKEESALKRESVRGRKRKLLHENNYVSRILSTIGATSENASKSCDSDQGNSESTDSGFERTPIKGKQRNRRFQVVDEFVPPIPCETSQEGIKEHDADPSKRSTPVHSLFTGKDSAPCPPSTQRTERKLGLAKKKTKKPVIDNGKSTLISFSTGSDGNQVKSQTGPSINAVSHARDLLNEKRVDSLFDNRLASEGYFRKYIPQPNDKPVISVHLQENDHVRLRDAEPNCLRDFSSSSKSSTGGWLRTGIDVVDFRSNNSNSDRSSFSNFMLRYAPSSTEVADVSRALQKDASGADRKGKTVMVQEHHGAPRSQSHDRKESTTEEQNDDIPMEIVELMAKNQYERCLPDKEDDVSNKQPSQETAHKSKNALLIDLNETYDNGISLEDNNTSRPPKPCGSNARREEHFPMGKQQNPHDFFPISQPYVPSPFGIFPPTQENRASSIRFSGHNCQWLGNLPTVANQNPSPSSFRVLRACDTCQSVPNQYREASHPIWPSSMIPPQSHHKPVSFNMDQSTKPGTLSHASNNENTWNLNFVAANGKQKCGPNSEFSFGCKHAAGVSSSSSSRPIDTFSSESSIPALHLLSLMDPRLRSTTPADQHGNTKFTKRHFPPVNQSKEFIELQTGDSSKTAYSTKQLPFDFYSKRFAHETSRKSFPIIPPLGTSSFSFQNAQASWSPHQEKKTKRKDTFSPVYNSHEKSVFASSNDQAKFQLLGASNSMMLPLKFHMTDKEKKQKRKAESCNNASAWPLKNGSGPIECSVNRNPADFTIPEPGNVYMLTGENLKVRKRTTFKKKPSLCKQDAMKQTKKPVCPATENA

>BrEMF1a Bra023327 *Brassica rapa*

MGSSIKINSISIDLAGAAKEIDMVKCDHFSIRGYVAETRERDHNKCWPFPEESVTLVDQENYSLPSLSAPKFRWWRCMSCIRDINADETKDCGLHPNSKSLSGKKQLDGNSSVILSQSKLNSLTIIDQEKERRTGIEDNAVEKKGDANCKRSQKDDHRATIFLKRGNNPSTDASTVKSKSRKLASQEHVRNKKAKVTDISSWKEKHNVGGQAVTTFGSSDIAGVVDDTPPKAIKNHNKDSLALTECDNGSSESINLAMTGLQRRKTRKVRRLSELLDQPETKTSGGKEEPSSSKRGRKRKVLPENNYVSRKLITVGATSENDSDQDYSTSSDSGFDRDLIKGKQKNRRFQVVDEFVPSVLPCETSQDLSKSTALSVPCPLSTQRTEKKLSLSKKKKHKPVSDNEKSTLISFGSQYTRDLLNDRLASEGYFRKPIPQLNVRPENDHVRSRDVEEPNRLGEFGSSSKPYTGGWLRTGVDANNNTDKLSFQNFNLRGTTSSTGADRKGKTVMFQEQQEPPKSQSHDRTEDQNDDIPMEIVELLAKNQYERCLPDKEEPSQETSHKPKNTLLIDLNETYDNGASLDDDNNISRPPKPCETNARR ESLDFFPISQPYVPSPFGIFPPPPPQDNRPSSIRFSGH

TCQWLGNVPAMSTHPPPYRVLRPCNTCQSGPHQYREPPPHPIWATTSVRPVSYNMDPSTKLGMLPQPPPPSDKNTWNLNFVAAAAANGKKKCGSSSELSFGCKHGGGVNNNSRPVEAFSSESSIPALHLLSLMDPKIGPNTPSDHQGNTKATQRHFPLASQPKGRVEIQTGDSSKQLPYDYYSKSFPMVPPLGASSFSFQKPQAPWIHHHQE KKTMRKEPVYSSNDQGRFQLLGASDSMKLPLKFHVMGKEKKQNRKAESCGDASAWPPRNSCGTIVCSVSRNPADFTIPEPGNVYMLTGESLKVRKRATYK KKTSLSKQDALKQTTENA

>BrEMF1b Bra006104 *Brassica rapa*

MSSSIKINSISIDLAGAANEINMVKCDHFSIRGFVAETRERDHRKCWPFSEESVTLGNQQSYPLPSLSVPKFRWWRCMSCIKDINADGIKDLGLHANSTSISGEKLDETSSVIPSQGKLNPLTITDQERERDNDIAGSAVVENENVNCERSQKDDQTATTLVKKVHPPSMDASTVRNKIRKLASREQVGNKRSKVSNSSTDTSSWKEKQNVTTFASSEIAGVVDDTPPKAIKNRHKDDKVSSESINLGFQRRKTRKVRLLSELIVDPETKATGGSNNIRQEESSSSSKARGRKRKVPPENNYVSRKLSKGGATSDQGDSDSADSGFDRDLIKGKKKNRRFQVVDEFVPSLPSQEGVHENDAAGPSKSALSKDKDSVPQRAEKKLKKKKNKPAIKDNEKSSLISFSSINTSYQSPRDLMNEERVGNSLDDRLAAEGYFRKPIPPQVNDRPENAHVRQRDAEASSLQEFGSSSRPNTGGWLRTGVDAVDFSTNNNNNTDKGFADLFSVLQKEASGADRKGKTVMVQEHHEPLGSQSHDRNENTPEEANDDITLEIAELMAKNQYERCLPDKEDDVSNKQPPTQEETPHRSKNALLIDLNETYDNETSLEDNNNNTSRPQQKPLDFFPLRQPYVPSSPFGIFPPPTQENRSNSIRFSSGHNNPQWLGNVPAMANQHPSSSSYRVLRGCNTCYQSVPHQYREPASSHQIWPPSIVQRPQCHHHQTPVSSFNMDQSTRLGPTNNNTWNLNFVTPNGKQRCDGNRSMDANESSIPALNLLSLMDPRLRSSNAPVDHHGNANFTRRHFPPSKERIGIETGDSSKTAYPSKQSPFNFYSNGYAPEASRKSFPIGPPLG TSSFSFQNAQAAAPWSHHHHHHQEQKNKSKDAVFPGSNDQGRFQILRGSNSVKLPLKEMNQKRRAESSSNASALPPKNSSASFVCSVNRNPADFTVAEPGNVYMLTSENLKVVRKRAPYKTKASLCKEDAMKQSKKTVGPVTENA

>BrEMF1c Bra009861 *Brassica rapa*

MEPPVKINSISVDLAKAPDEIDSGKCEHFSIRGFVAEIRGGDHRKCWPFSEDSVELRNHESYSLPSLSVVKSRWWRCGSCVKDINVKKINDSDCGLQSHSRSVKEKSLKAGSSAVPSRRRLNWLSVSDKKTKKNQVTAATLLKKVRRRGKDASTNKSKGSKLATPKQVKKRSKVSTEVVNGGEAFRSSDIAGVVDDTPDKATKKNLMSSHHVLEERDNNVSPQSRKLRKTRKVRLLSELLSTKSKETPSKRESTRGRKRKSCGDREDCTSAEMTNYASRILSTMGKTSENASKSCDSGEESRPSGESQSTDSGFNKEGKQRDSILQVVEADTAQMNLYDYASPVRSSFPGKEMVPCPLHTQRTEKEDTRRKRKAHITDYTKSTIITFGNNNMDDGNLVQPRRTDTLPGTTMPQSTRDFLNSKWLDNSFDRDKYTPQFDDRPFFQLPLQDPMHYKDGDGAMLKGIGTNHFRNFGSSFNSTNADAFLRTGVNVNFCSNIDTISSSSLLTDKLRHTSSTEVPYGSCSLQKDISTGHGKGKAPEHIDDIPMEIVELMAKNQYERCLPDREEDKQPTSSVSKNALLIDLNETYDNSMDNNNNTTRQPKPLAWNSDAATKEGQFMAGRQWISSVPFGVVNPPVSQFSGVVRPNSQWISSVPTTMANQQNPFRVLRACNTCQSVQQQQSREASPRPVWVSSTPAHHKPVSLNMQKFMDESTKVENPNTRNLNFTDNNGKQKLGFEGVSSRPLDTYSNESSIPAMHLLSLMDPRLRSNVPVDQSQNTSFAKPPVHQSKEFLGTQPGNSTKQWPFDLYSKRVVTPETSRGIIPPVGTSSISFQSEKTCAENADFRFQASWNHHQEKKDNEFGPTYNNSSQQKQVFTSSSSDPVRFQLLGASDSMKLPLKYHMKDKAKKHKSKNHNSNVSACPPTSTTCGRFVCIVSRNPAEFTIPDAPGNVYMIKGEDLKVSKQPSFRKKQNFCKQDALMQNKTSFASPGAENA

>BrEMF1d Bra008955 *Brassica rapa*

MRSSTKVSSIVINLTGANNEIGMVVCDGFSICDLIAEERETDPKKYWPFPEESASLVDKQSDSLPHWWPPSLGRTRNIDADGTNESGLPSNSTSISSTNGLNGSSSIIPSQSELNSRTAIDQERQRNIDVAGSAVVVNEDINCERSQVDDQRDNAVVETDDVNCERPQNDGQSDNAVVENENVNCERSQQDDQRDNDVAENEDVNCERSQKDDQRDNAAEENEDVNCERSQQNDQTDNNALVVNEDVNCERSQKDDQRVTRSSKKVHPPPFRTYALRNKTTKNYAVVEPIGNKRAEKQPNVEQLAVTEIAACAEDTQPEESESRSLFIGGAPSTRTRNVRTRRLAEVNGKRTVQKRKCRPGSSTTLSRNISTAGAASGNASKTVESAHGTESTESEFDKDPIKGKKKNVRFQVEDELDTEPEPEVDDEKDEDFYPWARRTKKVKIAKKKRATTAMNPSDKASSSVQPSLNETETVPSPPRDQGTEERVGTSLDDELASDGYVRKTNAPPVNERQENNRMRSSYVPVFGNPSIPNTGRGLGTGPGAIHFGSSSSNRTNQRNTSPSTQVAAQSTVPQKDASIPNTGRGLGTRSGATYFGGGSSSNNQTPPSTVAAAQSTVPQKDDSIPNTGRGLGTGPGAVYFGGGGSSNSNNRRSTPSSTQVAVPQKDASIPNDNSRRSTPPSTVVAAQSSVPQKDASVPNTSRLGSGPGAIYFGSSNNSSSQRSTPPSTVVAAQSSVPQKDASVPNTSRLGSGPGAIYFGRSNNTPPSTVVAARSSVPQDASVSDRKGKGVMVQSHDERENTQEVPPKTPFLFDLNETYEDVTSLEDNTNAAREEDRVPIRSNQNSVEIFTPRQNSYMASSSVMPPPVQESRRRSSILFPGHNPEWMGNVPMASPYHHPSPSTYQPMRVPPHHYGAPSPPVWASSMIPPQYHQHHFSPPAPFNMDYPSMFAQAPSSEPRNLSYYGANLLNQAMMSRMDPRFRSNTPVDHHGNFGFAPRHVHPVNQFNQFVELQCSHERSVYSRTISNQGRFQRRRNAASFSNASTSYAANSSGSSSCVVSRYPGEISEIEPGNIYLVHQEDLHVPERISEFQQADEEQAG PITESG

>CgEMF1 Cagra.7526s0001.1 *Capsella grandiflora*

MGSSIKINSISIDLAAAANEIDMVKCDHFSIRAFVAETRERDHRKCWPFSEESISLVDQQSYSLPSLSVPKFRWWHCMSCIKDIDADGTKDCGLHSNSRTIGNSSVIPSTSKLNSLTILDQEKEKKTDIAGNAVGENLDVNFERSQKDDQTAAAFLKKISARSMDASTVRSKSRKLASPEQVGNKKSKEKVNKTSMDISSWKEKQAVDQAVTTFGSSEIAGVVEDTPPKATKNHKGVRGLMECDNGSSESINLAMSGLQRRKSRKVRLLSELLGNTNTKTSGGGNIRKEESALKKESVRGRKRKSLPENNYVSRILSTNGATSENASKSCDSDQGKSESTDSGFERTPFKGKQRNRRFQVVDEFVPPLPCETSKEGITEHDADPSKRPTPVHSLFAGKDSAPCPPSTQRTEKKLNLAKKKTKKPVTDNGKSTLISFSNGIDGNQVKPQTGPSINAVSHTRDLLSEKRVDGLFDNRLASEGYCRKYIPQPNDKPAISLHLQDNDLRDAEPNCLRDFSSSSKSSAGGWLRTGVDVVDFRSNNDNTNRPSFSNFKLRYAPSSTEVADFSHVLQKDASGADRKGKTVMVQEHGAPRSQSHDRKETTTEEQNDDIPMEIVELMAKNQYERCLPDKEEDISNKQPSQETGHKSKNALLIDLNETYDNGISLEDNNTSRPPKPCASNARREEQFPMGKQQNSHDFFPISQPYVPSPFGIFPPTQENRASSIRFSGHNCQWLGNLPTVSNQNPSPSSFRVLRACDTCQSVPNQYREASHPIWPSSMIPPQSHHKPVSFNMDQSTKPGMLSQPSNNENTWNLNFVAANGKQKCGPNSEFSFGCKHAAGVSSRPTDTFSSESSIPALHLLSLMDPRLRSTTPADQHANTKFTKRHFPPVNQSKDFIELQTGDSSKTAYTTKQLPFDFYSKRFAQETSRKSFPIIPPLGTSSFSFQNAQASWSPHQEKKTKRKDTFTPVYNTNEKAGFVSSNDQAKFQLLGASNSMMLPLKFHMTDKEKKQKRKAESFNNASAWPLKISGPIVCSVNRNPADFTIPEPGNVYMLTGENLKVRKRTNFKKKPSLCKQDAMKQTKKPVCPPTENA

>OrEMF1 orange1.1g000866m *Citrus sinensis*

MERSIMGEENHQVDDSDLVSKSVGSSVKIDSISIDLVNNDEANAGKCEHFSIRGFVSEVRKKNWKLCWPFALDENHNKPEEEAHALPPLHVAKFRWWRCQNCLQELGSEGNTYGYGNGCNYCSSTSFKSTGTCPHMSSFGDAAMLQSAIGQARKLNISAGRKFDATASIATDLNVNDCCLPSHDDKNRKKSEPTNTPATVDESCPEDKMNHEVLESAGAPTAVTSGLMTGRHHAVEIGKQPSAEEKNKKLMIACGPSRVVGMVDEVLAAAKDLAIEHPSHELDECDNAPSDSDETLSGHDLQDHHHDSSSGSHRRKTRKVRLLTELLAENGDVKANLTRTESSPSNSVPGSSAGIDILSDPQGLVATQGNSIRPLDQNKKRKFPHDEEPDPLEKSSPNNFHRKNSTCTESAETIGALASLDTKEDADIDLHIGVNSRLNKFRFDRSPIGKKRNKSLVADEFLSLAPSGGNMLEDMQDRTGDSNNDSPSDSFLLESAQTAFISRGMYPFALPPQKSERSSSVCKKKSKTTQFDEWQAPLIPWSSDMLKEGPARRKDVEIKQMHSTISPFQSSQDATTGKGLQLSLNSCFTTQGYESKYFSPLEHSQFPWRGGGSKENGVMGKDIQTNCVGDSDYHSKSESVSFLRKGVHCDLSRENYRMSFLNEKQKFTPQVDAGGRSLMQPMDFCSRGNKGKSTEMNEHSALTMKPSDQQADKVSEQGVPDDIPMEIVELMAKNQYERCLPDVENEKMLETTINHSNAPIYAKEKLSFLSNETPQKPKPLAKIGRNGKIARPENGGTRKRKSVDCLPHIGRKHFNVSPLEQTHCASGFMPFAHCQGKLPNGIKFSDTSSSKHRISQNCQWIGNILGHRTSQTNLQTLGGCNSCQNVPQQGKETAHLWSSMIPPFIYDIHQKHASQSTNADMLSHCPSLHKGNVDGNHDLNFWNIDVTNIEKHSRTFDSESLSRPHAEYPFACKHSRSRPLDVYSNETIPAMHLLSLMDAQLPSGAPIGIDGNRKFLKRSSLPHDHHSKDFSGLSSGAYNTTSTMKPTQYDYYGKNHLSQSSPECFPAIQTVGASGFSFQHDKVVKKITGSTGQASLRYQERDKAKASDSPSQNKDNRSHQPVFITGVSGRGLGNIPVPNMQKIFVGTSDPAVFPLTFHEIENATKHKLESCNTTSTVLPPKSSSKTEICSVNRNPADFSMPEDENIYMIRGEDLKYRKSVFSENRPGLIKLDGRKRQKKLTAMKGKV

>CsEMF1a Cucsa.312630.1 *Cucumis sativus*

MLEKTRSDPVHRESVKNSKLLCGNEVAEVELGLRNLKVIDENLEGFDDEEQKTAHNEQTEVTRSPSGFKVIDQACNGERQRFPADIDGSYATASEHTEISVENDMQDHHIDKSGSLHRRKPRKVRLLTELLNENENVKTNHIDTEESPSHGTSEKSEGLKDLSVSQCPVAARKNVRCSGQTSKSKMPLNEDSLAAETSSSYNVYNKIQPLKGDVETNSFHASESENALIATDVRTKKSVLNKCGNDLKSLHDKKNKKIQIEACSPLDIPPGSGDNISDVSLKHNEFSSSAMDPFLLFGSRIEPISSLSKRKSKMPVIDDRRGFSWSNSMPRRDSASKEVELRNSDPVVVSCSSVLDECSEGLHLSLSSNLATSRNDKKSIFETEDGSHSLSSWQGRASVVRIKDTKTKKLKDSNDNSGVNKVEKSVQEHLAAQMKQSEQTVGNISEQRALDDIPMEIVELMAKNQYERCLDNTRNSKSLSKTSSKKARIMNFSYAYGSSDSLQEKNIPKWKPQVRNGRNNLHTVGDNVAYGKQSSGNYFSHTVGGHFSIDHLRQTIIPPEYSTFGHSQNKSSNPVNQYPGVAEDQESSHYRAQSFRVNNAHHPVSQNNEGVSHLWNEVPPNHHSYIPTTPRKVASQSTTVTANKNYPESSSRGSMNRGHNPKFFNPKVTNLEKDDGNYGLENFSRTSAKYPFYCHSNGIELPQNPRGSLDLYSNETMSAMHLLSLMDAKMQRSEMHENPKFTKKAFPHDHKAKDISGLDVGLHKAYDTINYSSDYYGEIHPLKKSHDCYHRPSMGGSSISPPMGNGSHEIVSDLTGKVALQCKQKEKTKCSTSTLNRAQKSQKSVLTSGQGSNEGVFPIHSLQKKSGGPSSSLVSMSGYPRLENPGQCIIERHGTKRMLEHSKVSSEFGICMINKNPAEFSIPEAGNKYMIGAEDLQFSKRISENTSDLNNMDGRKRKRNTKHAVVKQPALQHYRM

>CsEMF1b Cucsa.161390.1 *Cucumis sativus*

MMHRINVMEENNHHDGTDSRPARNFVQIDSIYIDLFSSDHICDDQKCELFSIRGYVSDMHKKDWKICSPFSDIIDNGHKLNEPIASVPSVLDPSFDAYQGKIHWQETSDKDADQGFLFDHNLGKFSNSSPNASKQDVISGRTIMADNVSNSYYDQKEKKLNVADRSDNCTVALISQSEPGCASHGVTEIELVSRNLTLKAAEESLAALQDGKQTPADCLNGQLTLLVSEKDDMVDVVHGHHTVKVQGNGDASMESNESTVSSSESAETVGNSPHNCHLGRLHRRRTPKIRLLTDLLGDNGNMVVKHVDQSSPSDGSPEASEQADVRFTSKCQVTIEEDASHPDHKRERRLARNGKCRHQEIPSSSSVDKQIQTWRGEIESSVSCLGTENAPSGMKSTMKGPWCSYKMDGNSSLRRKKSKKFPVVDPYSMSLTPSEVKDQCEIWEINENRSEVAVDSVAIFAHHNEFSCRIPHSISSNVIESKPGTSGNPNSSKEPVVFEGPTNVVPWNNRILWRGSVTQKDVETMNGNPAANPFPNFKKNEREWHPSLNNYSSLQKDHKGIRCRGENELSTFVPEQDDTSKVSQLNGNRTGSHRDPNYPHQASDVICGHGVDTVMNSKMTNLKMSLPRDPQTDNSQSQLQNKDLLRRGNGKRTIEAQEPLALKKRQINQRTDQPSDRGTSDDIPMEIVELMAKNQYERRLPDAENNYKHVSETGKFSRAVQVNNYDYVYRNGRELLQKPGNLKQNAQERNGGNGLICAREVVEARTHTPANYFSNIGESQFGISHLQQNHMLRCNDSIHSLEEPSNGMQYSSIGSKRKIRSEIRKCNGTTVESGPYNSKVQYSEGCIDHLPVSEQNIEAAYLWSTSSLMPDHMSNGYQNFPAHSTDSRKISSPRTFQMGNTNAQNHHNHHPTNLERHGRQKSTEAYSQRFAESSFCRHPNVVELQHNPVGSLELYSNEAISAMHLLSLMDARMQSNAPTTAGEKHRPSKKPPVPRTQKAEEFSATDICFNKTIQDMSQFSSAFHDEVCSSATNASTSTFQHSRGFGSGTNFSSQAVFRSQNGAKMKCSDSSSWSKDQKLSKSHFISGDDRTFPVNGIEKGLVNASNSEVFVLAHHMKRNSEECKLVAHTRTLQNEKSTSETEICCVNKNPADFSLPEAGNRYMIGAEDFNFGRTFLPKNRSGSICFNNRYKQQTFV

>EsEMF1a Thhalv10015703m *Eutrema salsugineum*

MRPSININSISVDLAGAANETDMENCNHFSMRSFVAETRQKEYRKCWPFDEESLRLVNQNYYLPSLSGPKFKWWRCTPCVRDINADETQGKAIVQNEDVNCERSQKNYQPVAATGIVIRTSPPRPTDVSTVRSKSRKVASPEQVSNKKLKEKVNKPSMDVSSRKEKQNVDQAVTTFESSEIAGVVEDAPSKATKNRRGSRVPMESDNVSSEGVSELQRRKTRKVRRMDDLLADDETKIIDLIRAKDSSSKMESGRGRKRKLLPENNYVRRELSTVGATSENASKSCEDAGQGDTELTEEEDPIRGKKKNRRFQVVDELLPTLPCETSQEGIRDADAGPSKSALPTPVQSLTKEKDSQRPEKKLASSKKKNNKPAIDNGKSTPIGSNSGGNQVKPRTGPSINASSQSTRDSLNEKSVGGSFEERLASEGYVRKSVPQLKEKPDLSLRLQSSGHVRSKEAEANYRQEIAGPSCELQMDGSVKEITTDQERNFPREVLELLAKNQYERTLPDKEDDVRNKQPQETAPPKSNGLLIDLNETYDNGNSLEDNVNNTSRPPQPSESNARKEERFPIGNQQQRSIGFAPVSQPFVPSPFARRPPPQPNRPSSIRFTGHNSQWHGNLPTMAPNPVPTPSSYRVLRPCNTCQSIPPYRETAAAPQPVWPSSMMPPQHRPVSFNVQQQPYFGMHPQNLNFNGMQRCGYTPDLSFGCKHAAAAASGASRANSSRTMDAFASDSSMPAMELLSHMDPRVRPNTPAGHHGNTNFTRRHFPPVNQFQTGESSRSAYQTKQLPFDPYGNRVAPPEPIRTTFPIVRPPPSFSIHNAEPSWSQQHQVNRSMRTDTVAPVYGSLENQVFPRRNEQGRIQLLGASGSLILPLKSHMAEKEKELKRKAESSSNASTRPLKISSGSMECSLNRNPADFTTLDEPGNVYMLTGEMLTGENSQARKRAPYKKKTSLAKKQ

>EsEMF1b Thhalv10003568m *Eutrema salsugineum*

MGSSIKINSISIDLADEVNAGKCEHFSIRGFVAEVRERDHRKCWPFAEDTVELVNDQSYSLPSLPVHKSRWWRCASCIRDIHAKEINNDSDCRLQSHSRETKLDVLSSAIPSKRKPNSLIVTDKKEKKNNVRTATLLKKVRRGAKDASTLKSKNRKLASPEQISKRSREKTNLSREIMETVTAFGSSEIAGVVDDTPDKTIKRYTHVSTERGECDNVSPESRNLLGLHRRKTRKVRLLSELLSNGNKEALSKRESVRGRKRKYSGDAAEMSNYASRILSTIGKTSENASKSCDSEDNTVSGADSRPIVENESTDSGFDREIIKGKKRNRKFQVVEDCFPETSQKDPYHYASNVQSSFAGKELVPCPLHTERTEKELSLAKKRKRKAMKDNKTSTVITFSNDMDDVVKLRKPDNFPRNTMPQSTRDFLNSKWLDSSLDKFLASDGHFNKYTPQLNDRLPLQEEMHYKEGGREMLKGIGTNHFQNFGSSFKSTNADGCLRTGVNVNFSSNRDTIISSSLNDKIRHTSSTEVADTSCALQKDFSLAHGKGKEVAVQELSVAHRSQCKTSEHIDDIPMEIVELMAKNQYERCLPDREEDKQPSQATTSVSKNALLIDLNETYDNSMDNTVRQPKPLGQNARNSDAAHFMTGKQSSLECFPTSYNVSFGVGNPPLTRDKQPSSVQFSGVLGPNSQWLGHVPTTMANQQNPSPSSYRVLRACNTCQSVQQQQFREASHPVWPSSLTSSPAHKPVSLSMQKFMDQPIKLGNPNTLSLNFSDNGKQKLGFEGVSSRPLDTYSNESSIPAMHLLSLMDPRLRSNVPVDQSRNTNFVKRPFCNQSKEFAAPRPEDCNQTAYSTKQWPFDLYSKTSATPEPCRENFSIIPPVGTSSISFQSEKISAGNAVFTPQASWNHHQEKKPKRKDKNFAPVYNNSKPIFTSSSSSEPGKFQLLGASDSMMLPLKFHMTDNAKKQKTKKAQCNNDVSACPPRSISGPYVCSVNRNPADFTIPDAPGNVYMIKGEDLKVKKHPGFKKKSSLYKQDVLKQNNSIAPNIENA

>GrEMF1a Gorai.001G027700.1 *Gossypium raimondii*

MESPIKIDSISIDLIKANGDIDTWKCEHFSIRGYASEMRRKDWKKSWPFASDGGQNTFKEQNVKLPPLLVPKFRWWCCQNCQQETGAEGSINEERNVNNNSSKLKSFGSCPLGDSVASSSGLLQAGKINVDSRKCDAIACLNVNTSTCHPLVSGKSNRKVENADKRVIGQTDILENNINKEIPNYAGLEVIASLMKQALRLDEKAASLQLCNPDLEENEVAGVKLLESNVECTVKDATATCETGKSACNQRMALVKGRGSHVKASTVHRVPDAIRTHIDEHSSLEFDDCDYASSESDEVLPGTETGSLHRRKNRKVRLLTELLVKNEDEKTNLTSTEDCPSSTIPDASIHMDSTSASQGQVAFQGNVTSGLARRRKRKMPRDEEWMPGELMSSPNNGHKNLRTFNRDAETAYGITSSDSEGTINRTSSQTPAKSNLVNLKVDRSPILGKKKNKKTQSIDECLSLRLSRENLQKERQKKPGDPTKSDATDIVLYKSNDVSAGSGFNPFTESVAKAEKKSNLLRKKSKMYQDHSQRTSPVPWNNGILREGPTSREDVEVRQIGNVAVPLEVTDDASPEKGLQFSFSNCFPAKRYDTKCSTPIRDGLQSLSSCQGRVLSEYDTGRKDLNMNHAGESTFPTKSQVDAYLGKGMRVDLNSNQNTYRIPFLNERQKHRSPAKVGSCSTMQQMDFSGTSTNGRTEFPDHATVAREHYDQHVEMVSEQGAADDIMEIAELMVKNQYEWCLPGTEIDKQLPETSNTKIQGVDLNKVYGNEEMNLFQETTDKPKAQAKNGRIGKFERGGNVGSSKQKSVDYFSHIDRNQYKISQLERSYPPAGFRPFPLCGEKPLNGVQFSATNSIRQNSAQNCQRLGNMIGQRSSHATVQALGVCNTCQSAPQQNKEVAHLWSSMISNSMPYVHSIPQKCADQIARLDVLSHCPSSLPKGNMSRNDDRNFLNVASNYEKHCRKFDSDALRRTHADYSFSCKHNAAGSVDLYSNETIPAMHLLSLMDAGLQSGAPVDVDGNQRFVKKTSFVPGHRPKELSSMASGGYRTNSMKHLSFDCYGKSHQPESFCECMSAAAAVSPTSFQHGKSFKKAPDFAGQFSMKSREKEKNKCPDSQRQSKNHRSQKTVSSNNGLNTTCGSIPVHSMSKLVLGTSDFTMFPMTLHPKESATKQKHKARTMSGTLFHPKSGSECGICLINRNPADFTVPEAGNMYMIGGEDLKFGREKAPSSGLGKLVGHKHERKLTVRKEHSRRTS

>GrEMF1b Gorai.001G027600.1 *Gossypium raimondii*

MFFLLIPFSRLAVKNRSWFKLSWSFLFISVILVTHSMETTTVVKETHHSCSSNLVAKSMESPIKIDSISIDLINANDDIDTRKCEHFSIRGYASEMRRKDWKKSWPFSLDGGQNIFKEQNCKLPPLLVPKFRWWCCQNCLRDIGAEGSINEERNVTNDSSKLKSFGSCPLVSSLGDSVVSSSGLLQAGKSNVDSRKCDAIACLNVNSSHPFVSGKSDKRVENTDVQVIGQTDILENNINKEIPKYAGIEVIASLMKQALCLDEKVASLQHHNPNLEDNEVAGVKLPESNVEHAVKDATEIRQTGKSACDQQMELVTGCGSHGIASTVHRVPDTFKIHTDGHSSLELDDCDYTSSDSDEVLPGTASGSVHRRKNRKVRLLTELLGKNKDEKTNLTSTEDSPSSTNPDASVHIDSVSASQGPVTFHGNVMSSLARRRKRKMPQDEEWMPGELMSSPNNGHKNLRTFNRDAETADGITSSDSEGTINRSSLQTPAKSNLVNFKVDRSPILGKKKNKKTQSIDQCPSLHLSRENLQKERQKKPGDATKSDATDIALYKSNDVSAGSGFNPLTLSAAMAEKKSNLLKKKSKMHLDHDRQASPVPWNNGILREGLTSREDVEIRQIGNVAVPLELTQDASPEKGVQFSLSNCFPAKRYDAKCSTSIRDGLQSLSSCQGCVLSEYDTRRKDLNMNHVGECTFPTKSQVDAFLWKGMHVDLNSNQITYGIPFLNETQKHRSPDEVGSCSTMLQMDFSVTSNNGRTMEFPDHATVAREHYDQRVEMVSEQGAADDIMEIAELMAKNQYERCLPDTEIDKQLPETSNTKIHQRVDLNKVYENEEMILFQETPDKLEAQAKNERIGKFARGDNVGSSKQKSVDYFSHIDRNQYKMSQLEQGYPPAGFRPFPLCGEKPLNGVQFSATNSIRQNSAQNCQQVRNMVGQRSSHANVQALGVCNTCQSAPPQNKEVAHLWSSMIPSSMSYVHSIPQKCADQVASLDVLSHCPSSLPKGNMSRNDDRNFLNLASNYEKHCRKFDSEALRRTHTDYSFSCKHNGAGPLDLYSNETIPAMHLLSLMDAGLQSGASVDVDGNQRFVKKTSFVPGHRPKEFSSMPSGGYRTNSMKHLSFDCYSKNHLPESFCECVSATPAVGPSTSFQHGKSFKKAPDFVGQISLKSREKEKNKCSDSQRQSKNHRSQKTSSSNSGLNTTCGSIPVHSLPKLALGTADFTMFPMTFHPKESATKQKHKAHSMSGTLFHPKSGSETGICHINRNPADFTVPEAGNKYMIGGEDLKFGREKAPSSGLVKLVGHKRERKHAIRKEHSRNRTS

>FvEMF1 mrna08809.1-v1.0-hybrid *Fragaria vesca*

MEKDILTDDNLNKRSDPIIASKSVGSFVKIDSISIDLDDANDDNSPAGKCEHFSIRGYVSEIRKKNWKICWPFALDGDLDKSDQEKSAMLPPLNAPKFRSWCCQNCLREIGSKNVAKSTDLIRHVGSDSKCTGFRMPPITATALLQSDCQQGPKSKIVEEKRFHAYTRTIVDGSETRPSSRIDKKKWNGEVSPTTVIANENSLANSMSHKMPRFSSAEREVNTNRMQERHNHVTEKLDCNGSVRVYKPGCESHEDASLELEITKNNSWIRNCTQNSETEYHSFASDKQKDSVNVSGLYKAGMIGETGNPIKVQTNVCRPLILDECDYASSENPEKSSGFHRKKSRKVRLLTELLCNKEDPKTDNSRKEDSPSNRINNTPEVESISQGQVSLQEAVRVGSGENKKRKLSEEEEKGGQETSHPKILSKKVKTLRRDGQATNAIAGTGLDEGASARIPLQADLKNNWVRNGNERIHTVGKKKAKKSHEFDAWSSLAPTAEKVPVEAQDKFENSSKNATNGASFRLMYVPSTVRGTELHSSKRDRKSGFGKRKGKMPQSDAGQASLSNTRKDAEIKPNGQGTVPFHLVEDASTQKGLDLSLNGYLATQRYDKNFPYQQEDGFPSLSTWKDSTCKLDEFMRKNVEVNYLANLNKPSTSMANPLSEDGVHGEPSKKVYTYSMPILNEEQNYSSQVEQGSCSLVQQMDISRGSKNQKIIDIEKNSGVPVSRKHSNHQAEMSEKGTIDDIPMEIVELMAKNQYERCLHETQNDKHVLEIPSTTRNPQMMEHTQVYGIGDWRVLEETSQKRKAQARNAKNGTRKNVASVQQKSVDYFSYINGNHFGLNRLDQMHCTTGFGPFSQSQKKPSPRGQFPAAGNSKCSCAQSCKWDGNLMGHGFSNSNLQSFAACNTCHSVPQSKEEAAHLWSPLISSLPLAYKNPQKGAAQSSNFKMLSHSTGALQKGNTAGDCDLSLNLNAPNFEKRNEAVGSETISRTKPEYSFTCKRNGTEPHQNSLGSLDLYSNETIPAMHLLSLMDAGMRSGTSLNVGGNPKYPKRSSSNDLSSKGYPGLDIGLYKATDTVNHPSSNYYGNNHLSEKSLNLFPTNPTFGASTSSFEHSKGFGRGSDFMTQVASSQRKEKIQRSHSPAQNRGPRSDKSLATDGGFGNNCTTIPVHTIPKGFLPVSGPMMFPLQYHSIANSRKHNLETPNANGTMKPPKTSLESSICCVNRNPADFSMPDVGNGYMIRGEDLKVGKKVSSEKRHGLYKVDQQKRQRNTNHIFEKQHARR

>PtEMF1 Potri.006G240500.1 *Populus trichocarpa*

MERTMLVEKNHPGSHSKLVSKVESSIRIDSITIDLDNVDEKVEAEKCSHFSMRGYVSEIRKRDWKICWPFVSDGDSNNYEEQACLLPPLHVPKFRFWRCQNCVWEVDATANCYGSTALKSCSTGFKSTKVCSHAPILGDDAMLPSDVQGAANQEIPEGTQADAFASLTNTSKCHHSQSIDKNERKTKDENVSNIGKSVGSEDNLKQENHRLACVATEVVSSPIQKTDLTDKIAAFKSKCINLCEPGCGHHEVVAAEFARNLNCMVNNATEICEAGKETSIDDQYKEIITRGASGEAGNIDDGALTADKDPVSRPSLELDEYDDPSSESTDIMVGNNSQDVHHENSSGLHRRKTRKVRLLTELLCENGDGDTDNQTQYSLPHAFPDASAGVDKVPVLQGEVAIQGKARRGLGQNRKRKLPQDEDSRSPEMRSTSKVCKEVRNSKRDGETAELSGGSESEEDAFGRMGLQTGMKSQWAKNKVDRSLVVSKKKNKKALSFDECLFSELSPEKAPIEIGEKISPEKATAVDDVLTKSVHNAFTGREMDFFPLHSSQMEKNVNDYKKKGKMPLFEDYQVSPSPWNHGILREGPVIRKDVGTIHAGLVPVPFHSAEDTYLEKGLDLSLNSYKTAQSYDGKHIPLVENRQSSLFTWQEGSSKNQAMRKATEIEHVGNFNFTSKIAQDAPFEKGIRSDPSTKRPSFKIPFLSEKQKYNFQVEIGGCSLMQKKDFCNTKSNEKTIGMQEHSAFPRKDINQRADKLSEQGALDDIPMEIVELMAKNQYERCLPDGEYEKRQLETTSSSRRSQMMNFSQVYGLGGLSLFHQETTQKQNPPARRNGIIKMGEMEESTKQKAVDFFSQADRNSFNMRRLEKTGSPVGFGPFLQHQEKPSSRVQHSACISNVQNISQNCKQIGDVVGNRSCYANFHTPGPCNTCHSIPQQSKEANHLWSSMMSNHMPFVYTIPPKCVTQSTNVNVFPHSSGSNLKENMNGDRELKFLNKNAANLGKQNRNFGSETLIRARSEYPFAGKHNGIELNQKPIGSLDLYSNETIPAMHLLSLMDAGVQSSAPINMDVNSKFLKRPSITHNPEPKEFSRLDTGAFKAVNTVKHPPPNHHGKNQLAENFRDHIPVIQTTAGASSSSILHDKGIRKATDFPIQVVQDKDKRKGSDSRTQNKVNRSQKSAYGGFGTNCGSIPAHNMQTMFYGASDSSMFPLPFRALEKPNKHKLESPANNRTVHAHKSSSETEVCSVNRNPADFTVPEAGNMYMIVGEDLKFEKEVPFVNGSRSLKLDGPKRQRKLPAVKGRGRPPMSRLS

**Associated factors: VRN1**

>AtVRN1a AT3G18990.1 *Arabidopsis thaliana*

MPRPFFHKLIFSSTIQEKRLRVPDKFVSKFKDELSVAVALTVPDGHVWRVGLRKADNKIWFQDGWQEFVDRYSIRIGYLLIFRYEGNSAFSVYIFNLSHSEINYHSTGLMDSAHNHFKRARLFEDLEDEDAEVIFPSSVYPSPLPESTVPANKGYASSAIQTLFTGPVKAEEPTPTPKIPKKRGRKKKNADPEEINSSAPRDDDPENRSKFYESASARKRTVTAEERERAINAAKTFEPTNPFFRVVLRPSYLYRGCIMYLPSGFAEKYLSGISGFIKVQLAEKQWPVRCLYKAGRAKFSQGWYEFTLENNLGEGDVCVFELLRTRDFVLKVTAFRVNEYV

>AtVRN1b AT1G49475.1 *Arabidopsis thaliana*

MRNMHTNRRSPGPITSAATQRRLKPEPEPTVKKFIKIILLSRIIEKMMKVPARFVRFGPKLTDNVTLQTPVGFKRSIRIKRIGDEVWFEKGWSEFAEAHSLSDGHFLFFHYEGDSCFRVVIFDVSASEIEYPLDDTDDNREEVMDDDEQGFTGFESSDDDGEVVDMDELLKKKKKKPRVNIKSENVIILD

>AtVRN1c AT3G18960.1 *Arabidopsis thaliana*

MVTTQNTKARVTSVSHRRSQQDPESPVKKFFKLVLPSTMKDKMMKIPPRFVKLQGSKLSEVVTLETPAGFKRSIKLKRIGEEIWFHEGWSEFAEAHSIEEGHFLLFEYKENSSFRVIIFNVSACETKYPLDAVHIIDSDDDIIDITGKEFVGTQGTRKNNQSVNGGDTEHKSKKRSRDIELDKILHDLDVMNPMHVLKEEEEDKRVFRG

>AtVRN1d AT4G01580.1 *Arabidopsis thaliana*

MVITRNMKARATSVSHRQSQQDPESPVKKFFKLVLPSTMKDKMMRIPPRFVKLQGSKLSEVVTLVTPAGYKRSIKLKRIGEEIWFHEGWSEFAEAHSIEEGHFLLFEYKKNSSFRVIIFNASACETNYPLDAVHIIDSDDDVIEITGKEFDTEHKSKKRPRDIEFDKILHDVDVMQVLKEEEEDKRVLRG

>AtRTV1 AT1G49480.1 *Arabidopsis thaliana*

MQMDSAQNQFNKRARLFEDPELKDAKVIYPSNPESTEPVNKGYGGSTAIQSFFKESKAEETPKVLKKRGRKKKNPNPEEVNSSTPGGDDSENRSKFYESASARKRTVTAEERERAVNAAKTFEPTNPYFRVVLRPSYLYRGCIMYLPSGFAEKYLSGISGFIKLQLGEKQWPVRCLYKAGRAKFSQGWYEFTLENNIGEGDVCVFELLRTRDFVLEVTAFRVNEYV

>BsVRN1a Bostr.19424s0762.1 *Boechera stricta*

MPRPFFHKLIFSSTIQEKRLRVPDKFVSKFKDELSVAVALTVPDGHVWRVGLRKADNKIWFQDGWQEFVDRYSIRIGYLLIFRYEGNSAFSVYIFNLSHSEINYHSTGLMDSAHNHFKRARLFEDLEDEDAEVIYPSSVYPSPLPESTMPANKGYTGSAIQSLFTGSVKAEETTPTPKIPKKRGRKKKNADPEEINSSAPRDDDPENRSKFYESASARKRTVTAEERERAINAAKTFEPTNPFFRVVLRPSYLYRGCIMYLPSGFAEKYLSGISGFIKVQLAEKQWPVRCLYKAGRAKFSQGWYEFTLENNLGEGDVCVFELLRTRDFVLKVTAFRVSEYV

>BsVRN1b Bostr.26675s0224.1 *Boechera stricta*

MPNTRRSSVRITSAASQRRLEPEPEPAVNKFFKIIHLSTIKEKIMKVPARYVRLGPKLTDNVSVETPVGFKRSIRIKRIGDEVWFEKGWSEFAEAHSLSEGHFLFFHYEGKSSFRVVICDVTASEIKYPLDKVYVIESDDDKEEVMEVVDDDEQGFAGFESSEDEGVIDLDELLKKKKPT

>BsVRN1c Bostr.19424s0764.1 *Boechera stricta*

MVTTRKTKARVTFSSHRRPPQDPESPLKTFFKLVLPSTVKKNMMRIPPKFVKLQGSKLPEVVTLETPVGFKRSIKLKRIGQEIWIHEGWSEFAKAHSIKEGHFLLFEYNENSSFRVIIFNGSACEIEYPLDVVHISDSDDDIIQITDKEFIGTQGKRENDQSVKGGDTEHKPNKRPRDIEFDKILHDVDALNSILVLKEEEEDERVFRG

>BsPTV1 Bostr.26675s0223.1 *Boechera stricta*

MDMDSAQNQFNKRARLFEDPEDKGAKVIYPSNSTSTEPVNKGYGGSTAIQSFFKDSKAEETPKVLKKRGRKKKNPNPEEINSSTPGGDDSENRSKLYESASARKRTVTAEERERAVNAAKTFEPANPFFRVVLRPSYLYRGCIMYLPSGFAEKYLSGISGFIKLQLGEKQWPVRCLYKAGRAKFSQGWYEFTLENNIGEGDVCVFELLRTRDFILKVTAFRVNEYV

>BrVRN1a Bra037544 *Brassica rapa*

MPRPFFHKLIFSSTIQEKRLRVPDKFVSKFKDELSVAVALTVPDGHVWRVGLRKADNNKIWFQDGWQEFVDRYSIRIGYLLIFRYEGNSAFSVYIYNLSHSEINYHSTGLMDSAHNHFKRARLFEDLEDEDVEVVHPSSLYPLQHPETTGHANKGHTSSAIQSFFAEPVKAEETTPTPKVPKKRGRKKKNADPEEINSSAPRDDDPENRSKFYESASARKRTVTAEERERAINAAKTFEPTNPFFRVVLRPSYLYRGCIMYLPSGFAEKYLSGISGFIKVQLGEKQWPVRCLYKAGRAKFSQGWYEFTLENDLGEGDVCVFELLRTRDFVLKVTAFRVNEYV

>BrVRN1b Bra014191 *Brassica rapa*

MNTTRRASARITSAASQRRQKPEPEPAVKKFIKVILPSTIKEKMMKIPARFVRLGPKLTDTVTIQTPVGFKRSIGIKRTGNEVWVDNGCSEFAEAHSISEGHFLYFCSEGNSSFRVMIFDVSASEIDYPMDKVHVIESDDDEVMEVMDTD DGEGFTRVDSSDNDSSDEEAIDLEKLLKKKPRVNVKFETINIGNVSRLKL KSCGFFSFFSCV

>BrVRN1c Bra022376 *Brassica rapa*

MPRPFFHKLIFSSTIQEKRLRVPDKFVSNFKDELSVAVALTVPDGHVWRVGLRKSDNNKIYFQDGWQEFVDRYSIRIGYLLIFRYEGNSAFSVYIYNLSHSEINYHSSALMDTAHTHLKRARLFEDLEDEDAAEVVYPSSSVYPSSQQHPEVTVAAIKGYASPAIQSFFAGPPVKAEEATPTPKVTKKRGRKKKNAVPGKAFFLSCFILGLIVMIGVIHPEEVNSSAPRDDDPESRSKFYESASARKRTVTAEERERAINAAKTFEPTNPFFRVVLRPSYLYRGCIMYLPSGFAEKYLSGISGFIKVQLGEKQWPVRCLYKAGRAKFSQGWYEFTLENNLGEGDVCVFELLRTRDFVLKVTAYRVNSI

>BrVRN1d Bra022374 *Brassica rapa*

MVTTRQKKAGVTTASHRRPPSEPDSSVKKFWNVVLPSTMKRNMMMIPPKFVNLQGSTLSEFVTVETPVGFRRSIKLKRIGEEIWLHGGWSEFAEAHSISEGHFLFFDYKGNSTFSVMIFHVSACEIDYPLDEVHISDSDSDSDDDVMDVTDEGFHGTQATRRNDQSVNGGGTEHNRTKRSRDDEFDKILNDLDGIKLLEEEEDGKRVFRGQQFF

>BrPTV1a Bra032286 *Brassica rapa*

MDSAQNEFNKRARLFEDRQKEETRVTHPLIPKSNAPLNEGYEGSTTTQSLFTDSKPEVATPKVPKKRGRKKKNPNPEEINSSTPENSSKFYKSASDRKRTVTAEERERAISAAKTYEPTNPFFRVVLRPSYLYRGCIMYLPSGFAEKYLSGIAGFIKLQLGEKQWPVRCLYKAGRAKFSQGWYEFTLENNIGEGDVCVFELLRTRDFVLKVTAFRVTQYV

>BrPTV1b Bra014192 *Brassica rapa*

MDSTHSEFNKRARLFEDHQNKDAKVIHPMIPESTTPLDKGYDASTTTQNLFNESKPEVATPKVLKKRGRKKKNPNPEEVNSSTPRGDDSENRSKFYESASARMRTVTAEERERAITAAKAYEPTNPFFRVVLRPSYLYRGCIMYLPSGFAEKYLSGISGFIKIQLGEKQWPVRCLYKAGRAKFSQGWYEFTLENNIGEGDVCVFELLSTRDLF

>BrPTV1c Bra001729 *Brassica rapa*

MGVLDEAFIQAPEHRPKTHLKHSDDHILSKEIPTIDLSSLQDPNCDKTALATEIAEACMRWGFFQVINHGLSLDLMRRVEKTVAEFFSLTLEEKRRVKRDEVNPMGYHDGEHTKNVRDWKEIFDFFLQDSTTVPATTEPEDTELRKLTNPWPQNPSDFREVCQEYAREVEKLAFKLLELISISLGLPGDRLTGYFKDQTSFLRFNHYPPCPNPELALGVGRHADAGVITVLAQDSVGGLQVSRRSDGQWFSVKPNPDAFIINIGNCMQRVPDKFVSRFKDELSVAVALTVPDGHVWRVGLRKADNNNKIWFQDGWQEFVDRYSIRIGYLLIFRYEGNSAFSVCIYNLPQSEINYHSTGLMDSASHNNHFKRPRLFEDLEDEDAETLHTTASAIQSFFTGPVKPEEATPTQTSKVPKKRGRKKKNADHPEEVNSSAPRDDDPESRSKFYESASARKRTVNAEERERAVNAAKTFEPTNPFFRVVLRPSYLYRGCIMYLPSGFAEKYLSGISGFIKVQLGEKQWPVRCLYKAGRAKFSQGWYEFTVENNLGEGDVCVFELLRTRDFVLKVTAYRVNEYV

>CgVRN1a Cagra.5105s0003.1 *Capsella grandiflora*

MPRPFFHKLIFSSTIQEKRLRVPDKFVSKFKDELSVAVALTVPDGHVWRVGLRKADNKIWFQDGWQEFVDRYSIRIGYLLIFRYEGNSAFSVYIFNLSHSEINYHSTGLMDSAHNHFKRARLFEDLEDEDAEVIYPSSLYPSTLPESTVQANKGYTGSAIQSLFSGSVKAEESMPTPKIPKKRGRKKKNADPEEINSSAPRDDDPENRSKFYESASARKRTVTAEERERAINAAKTFEPTNPFFRVVLRPSYLYRGCIMYLPSGFAEKYLSGISGFIKVQLAEKQWPVRCLYKAGRAKFSQGWYEFTLENNLGEGDVCVFELLRTRDFVLKVTAFRVSEYV

>CgVRN1c Cagra.5105s0005.1 *Capsella grandiflora*

MVTTRKTMARDTFSSHRRQPQDPESPVKKFFKLVLPSTMENNMMRIPPKFVKLQGSKFPDVVTLETPVGFKRSIKLKRIGQEIWFHEGWSEFAEAHSIKEGHFLLFEYDENSSFHVIIFNVSACEIDYPPDALHISDSDDDIVDITDKGFIGTQGTRNNDQSVNGEHKLKKRPRDIELDKILHEVDVLDSNQVLKEEEEDDKRVFRG

>CgPTV1 Cagra.4084s0003.1 *Capsella grandiflora*

MDMNAAQNHFNKRARLFEDPNNRDARVMYPSNPTSAEQMNRGYGGSAAIQSFFKDSKPEETPPKVLKKRGRKKKNPNPEDINSSTPGGDDSENRSKFYESASARKRTVTAEERERAVNAAKTFEPANPFFRVVLRPSYLYRGCIMYLPSGFAEKYLSGISGFIKLQLGEKQWPVRCLYKAGRAKFSQGWYEFTLENNIGEGDVCVFELLRTRDFILKVTAFRVNEYM

>OrVRN1a orange1.1g018533m *Citrus sinensis*

MPRPYFHKLILASTIRDKRLRIPENFVRNFKDDLSAAATLIVPNGMVSRVGLRRLDNKVWFYDGWQEFMERYFIRIGYFLVFRYEGNSAFNVYIFNLPSSEINYQPNALSNFEVPNHSKQYHIFAEMEDDDSEHVDQPTVNKTFNPPSFQNLLNSSKLSNSINGAGEANLQRLKVKLYSQDGETPKLKKPGRKRKIDPNVQASSAQEVHDGEMQFRFYESASARKRTVTAEERERAINAAKAFEPSNPFCRVVLRPSYLYKGCIMYLPSCFAEKHLNGVCGFIKLQLSDGKQWPVRCLYRGGRAKFSQGWYEFTVENRLGEGDVCVFEVLRAREFVLKVTVFRVSESAGFMSRH

>OrVRN1b orange1.1g017237m *Citrus sinensis*

MSEQQTTHGEAMSQPLAPYLPEEENQKRSCIFYKLIVPSILHDKKLRIPDKFVKKFGDELSTVAKLTIPSGRLWLVELRKLNKKLWFDIGWHEFVEHCSIRAGYFMIFRYQGNSDFNVYIYDLAKSEIEYPFNTCSSLEEEPSGNKQCSVPIDKDKGEDNSFGISKPSSSPCPVLSPSTINVEDEGAYYKKNKNSTSGVKLKYLHLAEEVHNQEATFQSPQDKGIIQFKSNVTNKSDEVGLRGRALTSEEKKSALRAAEIFKSNNPFFKVVLRPSYVYKSLLLHIPSIFARTYLNGIKGDVTLTGPNGKQWRVRCISQNGKAKFGQGWSEFVWDNNLDESDVCVFELVKTDDVTLKVTIFRVLQDARSEECQPSK

>OrVRN1c orange1.1g018886m *Citrus sinensis*

MPSSMKKTYPHFIDVILDSTIEDKKMKIPQNFVGRFGDELSNVATLTNPEGYVMRVGITRKDGNIWFDGGWNEFVEDHSIDVGYFVLFQYRKNSKFRVFIYNTTTCEIQYPSRNTFPPPRQNQATFDGSKSKNGCEMRGKTYRMEEVKVKEESDNVNDSMQDVIGTCNSEGSVHAKIHLSETQCSSVQETDLKIDSTKFKKAKHNLKDELRADSVDRVKLFALLEDMDIHICESRMTLEERQEAINVARLLKPEKPSFLVFLRASNMQLNYVYVPTSFARKYLNGEECVTVQDSDGRKLAVKVKQSRRKYLLTRWGKFFKKTNVKEGDILFFEMIQMKKILLKVSVFHA

>OrVRN1d orange1.1g022474m *Citrus sinensis*

MSSSAAETSSMFYSMILPSTIHNKMLRIPLKFVGQFGDELSSIAKLAVPDGRVWQVGLTKFGRKIWFDQGWQNFVESQSISAGYFLLFKYEKNSIFRVLIFDMTACEIDYPYEDDMACEMCVEISEEQFKHEAELLSRKRKMEDGLERDEPNAEPDLLVSLRKSGIYITEKYKELYSTEELERAVYIARAFKATNPKPNPLFMIIWKANDKPCREVYAPARFAYQYVDEDSKSVEVQAPSGRKWSLGIHWRATGGFFLAKGWAGVSDYVHLTEGDICIFELVR

HRDVVFKLHVFHQ

>OrVRN1e orange1.1g038275m *Citrus sinensis*

MQRIPPKFVRDFGHELSEDATLAVPNGRPWRVRLKRDEGGVWFDDGWYDFAKYYSVSAGYISIFKYGKNSNFRVLIFDFTACEINYKREEGSKLDKRSRQSAANYSFENFKAKHKYRECEPQIKKCRKEELVEKNESDDGGGGDIEYNLWDLLTEMGICISKKHRAFSTEESKRVLKIARSLNLKYPTSLIILPSSSITHNRVYVPATFAKHFASRDAKSMEVRVSNERHKFMNMNVCWRENGGFALSMTELVEDENLKEGDIFIFELISKRVAVLNLSIYHL

>OrVRN1f orange1.1g041048m *Citrus sinensis*

EGKRSCIFYKLIVPSILQDKKLRIPDKFVQKFGDELSSIAKFTIPSGRMWFVELKKCNKQLWFDIGWHEFIEHCSIHSGYFLIFKYQGNSNFNVYIFDLAISEIEYPSLEEPSDSKQCHVPIEKDKEKNSSLRILPPLRAPCPDPFSPATKVLDEGVCYKCNKDSTSGVKIEYLHIPKDEHNQETAFHCPQDKGIQFKNTSDEVGLRWRAVTTEEKKRTVHAAEMYKSSNPLFRVILRPSYVYRTLLLHVPTSFARKYLNGIKGYITIIDSNGKQWPVRCIFKNGGAKFSKGWPEFVWENNLDESDVCVFELIKSNDVTLKATIFRVLEDARPVY

>OrVRN1g orange1.1g042100m *Citrus sinensis*

MAPANFSGLFSSQNSSKTAHPNFQGCFHSGTPAKLLLKTSRSYFDSRTTAQQTLELSIPISPSSLEIPPRFVKVVLPSTLRDQKMRIPNKIVRKIGNELSDVAHITIPNGYVWQVKLKKEGRKVRSDYGWQDFVEAYSISVGSLVLFEYESNSTFQAHIYDETACEINYPSSNEESHTSGEESHP

>OrVRN1h orange1.1g042153m *Citrus sinensis*

RIPKNFVKNFGCELSKAATLIDPNGRISEVRIRKNGKTIWFHHGWYDFIKYYSISAGYLVVFKYVKNSTFHVLIFDMTACEINYPSKSEESKNEEFVEKEEPYEDNECDLQDFLEEMGICIARKHRIVTTEQSKRAVKFVESLELKNPSFMVILQQSDINHHRVYVPVKFAKEFFCRDTKSIKILVSNENEKPHQINWIQKGGFVFTMVRIMKSKKLKEGDICIFELIRKNKFLLKLYVLKGK

>OrVRN1i orange1.1g043499m *Citrus sinensis*

MPEDMHQQLDNFRMLRRLIKIFNEPDPNNRWKLKGASTIKQKFLIQKGLYTTVANNNVKQMAGGNRRAETSHFFKVILPSTVQEKKLRIPRKFVKRFGDELSAVATLNVPNGRVWQVGLRKDGRKIWLQDGWDNFAEYHSIAVGYFLVFKYAKNSTFDVLVFDMTACEIDYPYDYEETESEEGDEMETENSVEILSFTKMNTPPVQENQVKPGSKPEKKKIGGIKLETATNSREKKRCPTHGNEHGKLKKAAYHEVETDSSDGDQVFEEMGIFMSEGHRYLSVEERQSLVTAVRLFKPQNPSFVDILRSKKRYSYMYVPSKFSKKHLIRGTRSIKLQDSDGKEWPAQLTWSSGCGIKGGWPAFSKYKNLKQGHVCVFELIKAKDILLKVSIHASSE

>OrVRN1j orange1.1g043961m *Citrus sinensis*

MAESITAEPFRFFKIIEETLQDKKLRIPGDFVKKFGDELCDVATLRVPNGCVWKVGLTREGRKIWFNDGWHDFVKNHSIFNDYFLVFEYAKNSTFDVLIFDKTACEIEYTCAELENEKQNDGKKSKLREVAYEDEYKFVAVLEEMGICISGAYKFLSVEERQRIIYASRLFRLENPSFFS

>OrVRN1k orange1.1g044463m *Citrus sinensis*

MTERAAATWLFEQQWRKRLNPGADVSSPHFFKIIFPFVLAEKKLRIPRDFVKKFGHELSNGAQLTVPDGSVWQVALTRKGKDIWFHYGWEDFVESYSISAGYFLFFSYEKNSTFHIIIFNLDACEVDYLYVGEESMKDEQNSDPENEKSVEHVEQQSRKRKMEEVAEMDEPNSDNNFNLSVVLKKMGIYVTPRYRRFSAEESKRAISFARIVQPKNSSFMVILEQHFKSCRDV

>CsVRN1a Cucsa.093850.1 *Cucumis sativus*

KMPSKFIRMFGKELSSSVVLIVPNGGVWEVGLEKFNGQIWFSHSWNKFVDYYSIDYGFLLIFKYEGNSSFHVLIFDTTTFEIQYPHHDGMKLENAVEKSDYAISISSSHDCSDQFIDDNDDDNECRYELHTTKRSKIKLESCDHEFMSKRFKYIPSSFGKKYLSREDEIIEIQGRSSERGRWKIWCKGASAKRMGVGWGVFRKESNLRVGDVVVFELVKMNKNRVMKFTVFSSGSI

>CsVRN1b Cucsa.102080.1 *Cucumis sativus*

MTCYDFYHFFLPPINRGNKKMIPKTFVEDYGKALSNWVNLKLSDGLEWKVGLRKATNGALWLEKGWDKFSEHYCLEFGSLLIFTLLNGRRSSNFEVTIFDPTGVETKYVSSSPQLKEDSDTNADSVFKKCYNTPQQLHAEEECNNVSRETPSCKKRVVPKHEVKASRKKQQSPRKVETTQRFSSKSDHKPSFKVVMRRNNVQGRFNMNIPRRFAGAYFSPKMQSASLQVGNKKWDVSIKKYACSHVRFAAGWGTFHSENGLEDGDTCLFEMVNTKLCVLKVSIFRKVSTSVSMD

>CsVRN1c Cucsa.102090.1 *Cucumis sativus*

MPSSPKFFRIVLHRNLEDPKMMIPKKFVEDYGKLLSNSVNLKLSDGKEWRVGLRRATNGAVWLEEGWDKFSEHYCLEFGLLLVFKLFDGRRSSNFKVTIFDPTGVETKFISPSPQIKEESDSDSDESLESLTLHGDLKKRKNVSVSCSQSRRKMRKDDLFTVKTELEEEEEEEECKYIFREIPRCKERVIPKHEAKVSRKEQPSPEKVETVQRFSSKSDRKPSFEVVMRQSNVQGRFNMVSPSCFISPPLNSLV

>CsVRN1d Cucsa.109990.1 *Cucumis sativus*

MDNSLTAMPESNEQEGAQSFNQTSPARRAGLFYKLVVPSILQDKKLKIPNKFAKKFGGDILDLVTLVAPNGYRWVLELKRHGRSMWFEDGWHEFVKHHCIQVGQLLVFRFEGNSVFNFYMFNLTAIPNGPCNTSNASIEQNDGEQCPDTLGKEAEYKKLVEILGTGSPDPSPRPSVKDLVCEFPDQQKFNGSCNGTSIKNFMHWFDTENLHPLKDFDNPLKHLDKLRMQLLNSNRDIGIQFDGDELAKARENHDFQLNQSSDEREEGAMKKKLKLEPIDYYNDNEPIDEKKCGNVPHKINRMAFGVEEFKFGNPFCWIVMRQSYIRRGFHLHIPSKFAEKYLKGVWGDITLQVSSGKQWRVRCIREGPGTKLTRGWADFVVDNDLKEEDVCVFELINMKDIVMQVTVFRVHGDPTKT

>CsVRN1e Cucsa.110000.1 *Cucumis sativus*

RIPETFVRMIRDELSAVATLTVPDGHVWRVGLRKADNKFWFEDGWQGFLEHYSIRVGYLLVFRYEGNSSFCVFIFNLNTSEINYQSAALSNNQRNNYSIQNRIFEEMEDYDIPEAIPSNQSMNSAFKLNHVSPNKGILHSPLQYLPSCFAEKNLSGVSGFIKLQTPDGRQWPVRCLYKVGRAKLSQGWYEFCLENNLGEGDVCVFELLRMREIVLKVTMFRVIEEGGRMANPNPASMMNPPPLRSVSHIKLIRN

>CsVRN1f Cucsa.366500.1 *Cucumis sativus*

MDSFNYWKNRPRKALDNPHFFKVVMASALKDQKLEIPTKFVREYGVKAFATNQICLKVAADGRKWNVGLTKSNDGTRVWFHDHGWQTFVEFYSVGLEYFLVFKYERQSSSFYVVIFDRTATEIEYPIKIICLDDAEEKRSVPKETHSKTGEVNFGAVELMDNKKKLWPTTTNQRERAMASASAFQSMTLNPSFMCKMSTSHIHPGKSLYTKKVCRDAYRKVHGEHSSNFRWNWEGSCCWYMTKMMKKRAELISGWRKFKKDNNLKVGDFCVFEMIGLSSTRISFKVEIF

>CsVRN1g Cucsa.366510.1 *Cucumis sativus*

MASSSHYRSNSIVKQSSGALHNPHFFKVVLDDALKEQKLEIPRKFVREYGLKALLKDQICLKVRDGKEWNVGLTKSNNGTRVWLHYGWEKLVGFYSVKSGYFLVFKYENPSPSFYVVIFDHTATEIEYPVKKTRLDKAKEKVKMETYFDDDCLDLDFKDGDLSSGVKKPTNSRWRPPSTGSQQARAMAEANRFQSMTLNPSFICKIWPSHIHRTKSLSIPKNFADMNLEDSITKIILKASDGRMWSGECGFYWTPKMQRMTFYKYGWKSFIRDNNLAVGDFCVFEMTGKNSTTVAFKVESFRAS

>CsVRN1h Cucsa.366520.1 *Cucumis sativus*

MANLFRSPNPHFFKVILHQTLTQQKLDLPNKFVKNNGGSLLFNKVTLFLPDGANWKIQLKKLDGKICFRRGWPEFVQFYSVQPGHFLVFQLKGICCFNVLIFDTSATEIDYPIRRLLDLIPKSRNEDEEESIQILNEMVLKKRRMEKEEETPPAYRSLRGLKKMMRKNAKVKIAKNRGITSDEDEGDLEMDNSRRQSNHRGIVKKRASWFKSRRNNPSFMVTMRPSYIQTGNYLSLPRRFGEKYIKESVDVKLEVGDGRCWRVWCGVRWAFTRRRTELKGGWKRFAVDNELKEGDICVFELMKKNGSVLTTSFIGIVSIQEVYTCFRIERVDST

>CsVRN1i Cucsa.366590.1 *Cucumis sativus*

MGLFTSPNPHFFKIVLHQNLTQQKLDLPNKFVKNNAHSLLFNNATLSLPDGSKWNFQLTRLDGKICFLRGWPEFVNFYAVQPGYFLVFQLKGICCFNVLIFDTSATEIDYPMRRLPVIVPKSESDDEGESIQILHEESDEHLPISEYNEREDGRSSPESMRPMNPRKTQQVLTENQLVVVRRASSFKCRTKNPSFMVTMRPSYIQTGNCLSLPRIFSERYIKESVDVKLEVGDGRIWKVWCGVRWAFTRRRTELKGGWKRFAVDNELEEGDICVFELMNKTPKREGERSLNN

>EsVRN1a Thhalv10021075m *Eutrema salsugineum*

MPRPFFHKLIFSSTIQEKRLRVPDKFVSKFKDELSVAVALTVPDGHVWRVGLRKAENKIWFQDGWQEFVDRYSIRIGYLLIFRYEGNSAFSVYIFNLSHSEINYHSTGLMDSAHNHFKRARLFEDLEDEDAEVIYPSSVYPSPLPDSTVPANKGYSGSAIQSLFTGPVKAEEATPTPKVPKKRGRKKKNADPEEINSSAPRDDDPENRSKFYESASARKRTVTAEERERAINAAKTFEPTNPFFRVVLRPSYLYRGCIMYLPSGFAEKYLSGISGFIKVQLAEKQ

WPVRCLYKAGRAKFSQGWYEFTLENNLGEGDVCVFELLRTRDFVLKVTAFRVNEYV

>EsVRN1b Thhalv10011760m *Eutrema salsugineum*

MKTSRRSSVRSTSAAYQRRLEPEPKPALDRFMKIILHSTIKEKMMKIPARFVRLGPKLTDTVTIETPAGFKRSIRIKRVGDEVWFENGWSEFAKAHSLSKGHFLYFYYKGNSSFRVVIFDVSALEIDYPLDKVDVIESDDDDDDEVMEVMDLEEEEEEEEEGFSGVESSDDDSCSVIDLEEMLKKKPRVNIKSEQITTDDVEEIDSMDDIEEYEIALTRRKRSS

>EsVRN1c Thhalv10021527m *Eutrema salsugineum*

MVTTRGTQARVTAVSHRRPPTKPESPVKNFFKVVLPSTMKSNMMRIPTRFVKLQGSKLSEVVTLESPVGFKRSIKLKRIGEEIWFQEGWSEFAEAHSISEGHFLIFKYKGNSSFLVTIFDVSACEIDYPLDEVHISDSDDEVVDITDAEFLPTQGTGGDDQSVKGGASEDKRKNRPRDNEFEKILNDVDGINPIVLEEEEEEKRVFRE

>EsPTV1 Thhalv10012062m *Eutrema salsugineum*

MDSAHNQLNKRARLFGDLENKDAKVIYPPIPASTAPLNKGSDGSTATQSLFAESKPEETPKVLKKRGRKKKNPNPEEMNSSTPGGDDSENRSKFYESASARKRTVTAEERERAINAAKTFEPTNPFFRVVLRPSYLYRGCIMYLPSGFAEKYLSGISGFIKLQLGEKQWPVRCLYKAGRAKFSQGWYEFTLENNIGEGDVCVFELLKTRDFVLKVTAFRVNQYV

>GrVRN1a Gorai.002G006500.1 *Gossypium raimondii*

MPRPFFHKLILSSTLQDKKLRIPDNFVKKFKDELSVAAALTVPDGHVWRVGIRKGDNKVWFQEGWPEFLDRYYIRIGYFLIFRYEGNSAFSVSIFNLYNSEINYQSNALLGSQYNHGKSYPFDELEDDECMSSAMQHLFGGSKLNHCVNWSGEVNLNAAKSANNQPIRVKLRTSGSETPPPKKPGRKKQKFDPNDQDLSVGHEDDSEMRYRFYESASARKRTVTAEERERAMNAAKAFEPSNPFCRVVLRPSYLYRGCIMYLPSCFAEHLSGVSGFIKLQLP

DGKQWSVRCLYRGGKAKFSQGWYEFTVENNLGEGDVCVFELLRSREFVLKVTVFRVMDGAGLMHRSQYNAN

>GrVRN1b Gorai.003G020100.1 *Gossypium raimondii*

MPCNFVKENLMKKHCSVILCNSSGKTWIATFKQRKIGKKLTSYLIAGCGTFARDNNIQVGDVCASELINSIHISFKVVIYQGQHANCHQSLAVTDVFHLVKRKAPSCAPSYASQGCPEPLTALEKAKAFQTAGAFKSENPFFVIVLQPSHVHGNKLSVPMNFARKYLTMMHKKVIHLLSDGNSWPVIYDPRFEWSYVFLCNGWHRFAVDNNLEVGDVCVFELTGGIETSMKVTIYKKQAIEDENLG

>GrVRN1c Gorai.003G020300.1 *Gossypium raimondii*

MASSSHQQGNSHLKFISSSPYFLKIILQDTIQNGKLFVKNHGNSMSSPAMLSVPSGAVWKVELTKSDGKIWLENGWLEFSNHYSLDIGHLLVFRYDGNSNFRVIIFDKSASEIQYPYTSNNHSRSSEILKLNINESKDDGSI

>GrVRN1d Gorai.003G020500.1 *Gossypium raimondii*

MAASHQQGNDHLKFISNSPCFFKIILQDTIQNGKLGIPRKFVRNHGNSMSSQQAQAWKVELTKCGGKIWFENGWLEFSNHYSLELGHLLVFRYDGNSNFHVIIFDRTASEIQYPYTSNNQKQSNEIPKQNINESKTECNGKSGFLAQQVSHNGCPAVKGDKRTNHPVIQRMKGREKVKALEKAINTFKSKNPFFPGSRSKG

>GrVRN1e Gorai.003G020800.1 *Gossypium raimondii*

MAASSHQQGNGHLKYISDSPYFFKIILQDNIQNGKLGIPKKFVKNHGNGMSSPAMFSVPSGEVWKVELTKCDGKIWCENGWLEFSNHYSLYIGHLLVFRYDGNSNFHVIIFDRTATEIQYPYTSNYHRQSNVILEQNIDQSRKPGEESQLPRPRPRPQSHKMVRSTKSAMETETEAECNGKPDFPARNGDTSTNHNTVRRLNTCEKVKALERARNTFKSENPFFLVVIQPDYVGLSLGKRYRLAIPADFVRENLMKEHCSITLCHSSGKTWMVTFKQQKGQK

LYSFLQTGWVTFVRDNNIQVGDVCAFEAFL

>GrVRN1f Gorai.005G240900.1 *Gossypium raimondii*

MPRPFFHKLILSTTLQDKKLRIPDNFIKKFRDELSVAAALTVPDGHVWRVGIKKVDNKAWFKEGWQEFVERYYIRVGYFLIFRYEGNSAFSVSIFDLYNSEINYQTNALVGTQYNLGRQYPFEELEDDECVSPAMPNLFGRSKLNCINWSGEVNHHAPKGVNNQPIRVKLHSSGAELPKLKKPGRKKQKFQPSEEDSSLGHEDDMEMRNRFYESASARKRIVTAEERERAINAAKAFEPTNPFCRVVLRPSYLYRGCIMYLPSCFAEQHLSGVSGFIKLQLPDGRQWPVRCLYRGGRAKFSQGWYEFTLENNLGEGDVCVFELLRSREFVLKVTVFRVMESAGLMDRSQ

>GrVRN1g Gorai.008G071400.1 *Gossypium raimondii*

MKVQTYPVVKNASKACGTQALGSIVSRVKTRSLVSDAKRSCQQSARLPSCREFIDLTDSYIESLDDSPLDQKTKKKMTSPSFQPCKLRTNPSESNQAKGIKLEKEKKSLNFQYLTKEVGGELKCSVKNDSGRRSSARRRPKPDPVYGKQRACVGASAFRTSNPSFSVVIQPSYIGSSSALHIPVEFVKRYLKKSGEMVLRVADGRIWVVEYRRTASNKGRKAKFGSRSWGQFAKDNQLEVGDVCLFELMNENGNLLEVAIHRKHLLIEID

>GrVRN1h Gorai.008G071500.1 *Gossypium raimondii*

MAHLFTNHALHIRKYVTAACTIAAACIQAVCTQQNHHNSRHFNKNITATWLAIFQHKYYSNPKNKDRNAYLYGGWREFVEDNHLNVGDVCVFELIKLPEILMKVVINLLVENTSKACDSLAFGSIDSRVNTRSLVSDAEPTCQQSLCPSSSGESKDLTDSYIETLDDSPLDQETKKLTPRSIQPCELKYSAEDDSGRTSGAQRCLKPDPVYQKQRACIGGGAFRTSNSSFSVVIHPSYVGSCSALHIPKEFGKRYLKNSGEMMLRIEDGKTWTVEYERRARN

KGRKAEFKSNSWGQFTIDNELEVGDVCVFELINENGNLLEVAFRKLYSSKLINY

>GrVRN1i Gorai.012G150900.1 *Gossypium raimondii*

MFMCYDVYMHLNHSSPDFIICQITKPNLIWVLQRIPDNFVKKFGDELSVAAALTVPDGHVWRVGIRKADKKVWFHEGWQEFVERYYIRAGYLLVFRYEGNSCFCVSIFSLYNSEINYQTNAFVGTQYYHRKPYPFEELEDDECISPALQNLFSRSKVNNCMNWSGEINFRALKGMNSQSFRGAVLSKPNKPGRKKQKFDHAEPDSSVGREYDVYTNFRLYESASARKRTVTTEERERAINTAKSFEPMNPFCRVVLRPSYLYRGCIMYLPSCFAEKYLSGVSGFIKLQLPDGRQWPVRCRYRGGKAKFSQGWYEFTLENNLGEGDVCIFELLRSREFVLKVTVFRVRESAPVSMKCRPELNQLTYSEHTLPDDVKPIRVDKSMPF

>GrVRN1j Gorai.012G151000.1 *Gossypium raimondii*

MSQPVGPSLPLNNSSCIFYKLIVASILQDKKLRIPNKFVKKFGDELSSVATLTVPGGRQWLVELREEDKRIWLDNGWNTFVEYYSICIGYFVVFKYEGNSHFGVHVYNLKSSEINYLSNNSREPGDFAEITGSSSYFLVDKDVDESLDHEKKKYKISTGLHQENELRDLRATFQSTLDKGIQFNGVELMSIGDEGGPCFSNETLRYTIKQEVEPSMDEQEPFRKFKVKEELPTLDSPRVLRRRRDVTTEEKQGAFHAASMFKPDNPFCRIILRPSYVYKGVLLHLPRCFARRYLNGVDGVIMLQIPEGKKWPVQCVYCNDNLKFSKGWAEFVLDNNLDEGDVCIFELINTKEIVLKVTIFRALRD

>GrVRN1k Gorai.013G039700.1 *Gossypium raimondii*

MSQPTGPSLPHKKSCIFYKLMVASILHDKKLLQCSKQLKTCCVVIKLNDVSKKIPNKFVKKFGHELSSIATLNVPSGRLWLVELRKENKRVWLDCGWTVFVEYYSICSGYFLVFRYDGNSHFNVHIYNLNASEINYQSNGLNDSREPGHDKHLKDVEDGAFAQILRSQPTSSSSCFLIDDDFDECVDHDRKKRKNSTFLDQKNNVDDLRATVQSTRDKGIQFNGVELTSAADEGGLNFLNGTQKNTKEIKQEIEPDIDEYKSLGKFIVKEELPAMNSPRSDHKKRRDATAEGKQIALRAAAMFKPDNPFCRVILRPSYVYKGIFLHIPRCFALRYLNGVDGIVTLQVSEGKKWPVRCIYGQSSWKFSKGWAEFVLDNNLDEGDVCVFELISTKEIVLKVTIFRVLEDGVAVNQL

>GrVRN1l Gorai.013G039800.1 *Gossypium raimondii*

MKEIPRSVKQYPFEQLQDNECISPALQNMFGGSKLSNCINWGGDVNRQNPKAVNNQPIRGSGAMKPEPKKRGRKRKFDPNVQDSSAGREDDADMRLRCYESASARKRTVTAEERERAINAAKAFEPINPFCRVVLRPSYLYRGCIMYLPSCFAEKHLSGVSDSIKLQLPDGRQWSVRCRYKGGKAKFSRGWYEFTLENNLGEGDVCVFELLRSREIVLKVTVFRVMESGGLMHRSQ

>GrVRN1m Gorai.013G040000.1 *Gossypium raimondii*

MPRPFFHKLILSTTLQEKKLRIPDNFVKKFRDELSVAAALTVPDGHVWRVGIKKVDNKVWFQEGWQEFLERYYIRVGYVLVFRYEGNSAFSVSIFNLYNSEINYQTNALVGTQYNHGKQYPFEQLEDDECISPALQNLFGGSKLNNCINWGGDANLQTSKGVNNQPIRVKLHTSGSGAMKPEPKKRGRKRKFDPNVQDSSAGREDDVDMRFRCYESASARKRTVTAEERERAINAAKAFEPTNPFCRVVLRPSYLYRGCIMYLPSCFAEKHLSGVSGSIKLQLPDGRQWSVRCLYKGGKAKFSQGWYEFTLENNLGEGDVCVFELLRSREFVLKVTVFRVMESGGLMHRSQ

>FvVRN1a mrna09434.1-v1.0-hybrid *Fragaria vesca*

MATMSRPMASRSNREGRGHAFPVDSPSFCLKIVTGVDLQDGKELPEPAVRKYGNCMEDSIFLKVPNCETSWPVELKKTIRHSRIWLQKGWEQFTDFYSVDQDYFIVFSYEGKHSHFQVHIFHNNNMEIDYPICGGGGGGCMSNLQGSSFPSASSAARDQNSNNLQGFHGANKYKVDKPRFWITMTASYLSVYLNIPAHFTIKNLCGIGSSCNVTLQIEGKRTWTVLCSVSKCGLKARFNPAGWKAFVKDNQLEAGDHCRFELIGERKLKVVIS

>FvVRN1b mrna13990.1-v1.0-hybrid *Fragaria vesca*

MASSKRRKGNSLAIQEKTPGFFKVVLNKALQDGKLEIKGFVASKYRDFLANSVILKDPNGAKWPVKLTRSDGSTWLEKGWLKFAHFYSLEEGYFLFFSYECVDSCFHVRIFSKNYIEINYPFKSSQHEEPNLGGGDGVKFSSADSFKSTFPFCVIKVQPTYVKYYHLQIPISYAKNFICRGQTRNVDLRIPKGKTWSAKSSVTKQAGRSEIARIHGGWSKFAKDNQLRVGDDIVLEMIKQQPKTTFEVHIFRA

>FvVRN1c mrna14412.1-v1.0-hybrid *Fragaria vesca*

MGSLRKHQTFSSTTPQFFQIILEDTSRDIKIRIPKKFVMKYGQDVSNSARLKLPSGAEWQVELTRCNGKVWFEKGWPEFSKFFSLDYGNFLVFRYEGNSVFQVCIFDRTATEIDYPIAMPGMEETDHEDEEGDDISIEILEDSPLVPRPEVKREKSPLPCPPSYKKMRTSWSDKTVETMSENDVGGSSPTRKCLNRTLEDLGEFQSLNNDEKARALLGADGFISEKAHFKVVMQPSYVQQSYLGLPAKFFKENQIKAAAYVTLRVSNGKTWSVKFNYEQSKATLQHGWLAFVKDNCLKVGDVCVFVLIKDINLLFQVEIFRATIFPVLAGHSRGAAVHVEDNRSSMKVESDCGMDCGMASVNPSPLDIGNKNKGSETSGEVAERHCSFLRVPRDNLEAASKFIPKNPFFRVTIRAYHLARSGVNVPANFMKKSIKRLQEKQTVMLQVKNSLWPVNLVPRTQGSAGRLCSGWITFAREHDLKEVVVLPTFYNRDDAMASLILSGSISEGKECVTKGADRGSQSCSWENSERSGEGRREELRRKAKMSLVDYASSDDDVSEEEDDKNKENEPAQAPMEEPHPPSRSQPQSVVSSYQRPESAAHLSAPSVQKLPDASQLFDSPDFSPNMMSGSYHSSHVGGESASRKRESNSIASSIPRSKVPRGNLPHSRNVPDTLGGMLVPPQLNGRSNVVTEDVSKLFVKKPVGGSPQ

>FvVRN1d mrna21463.1-v1.0-hybrid *Fragaria vesca*

MATGRQPSFFKVLKGDDFCEKLKIPRAFLMHFDGTVPEQCQLRIRTQTWLVDVERVHRNGNSEFYVHIYGIDACKREFAEVAREVSDSAKWNYVSRIEEADSDDASVQAGDDDDDKEEDDDEDDDFENEADSEDKDEYVEEEVESDRDEGKSDDSIDILDEFPQCSKVETKRTSSGLHKRKINCSQAKAQSIKRRASGWSSNQRIKALKQNGTGELIARHRAADFMSKSVDPSCLIVMSPSYVKGSCVHFPREFSYRHLKMKCSHINLRVNKKSWSLNIYQGIRNKSFKLQAGWPEFVRENNLKEGDVCVFVLNETIRYVFDVTIFRTTEAADCTLPGE

>FvVRN1e mrna31955.1-v1.0-hybrid *Fragaria vesca*

MKRPYFEKLILSSTLQAKQLKIPENFAKKFRDEFSTYVTLSVPGGHVWRIGLKKAAENKYWFQDGWQDFIERYSIRIGYFLIFRYEGNSSFIVNIFNLTTAEINYPPNALSGTGGTVCSNQYQVFEEMEDEDSVEIMGSSPSSIVTNTMRDKLFGDVANQLTPGKNYSPPSLQNLFNGAKHNWTDAGKLHTPKVTGLQGNNSTQDNGTERKKSKKAVDEVKSCTPGEGTQSDKKTVRKRRKRDPNVQESSAQHEQDVEIRFRFYESASARKRTVTAEERERAVNLAKTFEPVNPFCRVVLRPSYLYRGCIMYLPSCFAEKNLSGVSGFIKLQSADGKQWPVRCLYRGGRAKLSQGWYEFTVENNLLEGDVCVFELLKTKEIVLKVNVFRVQEEDAGFVTQSYGNQPSGQNIIQAIPLRN

>PtVRN1a Potri.004G146900.1 *Populus trichocarpa*

MEYEGKATSHDTHLPSVGKCTLGSCQSTHCCCHSTNTQCCVRKGADLKSKKLIFQSDLYHYVNCLPHSLPHSIPVLPYPKSQIKMPRPFFQKLILPGTIREKRLRIPDNFVKKFGHDLLGFARLIVPGGHVSRIGLIKADEKLWFHDGWQQFVERFAIHIGYFLIFQYEGNAIFNVHIFNLPTSEINYHSNSLSGKIYLAFEELEDDDSAASSGIPTTQLIVNKSYNPPALQNLLSGSKLNNCLNWGGEENMHLTKSANVSQVANESARNVFAQYNEHKNSQEEVKLYSPDGETPKLKKRGRKRLKVDPNEQQLSSPNEDDGEMSFRFYESASARKRTVTAEERERAMNAAKAYAPDNPYCRVVLRPSYLYRGCIMYLPSGFAEKNLNGLSGFMKLQLPDGKQWPVRCLYRGGRAKFSQGWYEFTLENNLGEGDVCVFELLKSRDVVLKVTVFRVLEDGGLMNHP

>PtVRN1b Potri.004G147000.1 *Populus trichocarpa*

MSQAAVKPDGSDSPARKSCMFYKLMVASILQDKKLKIPKKFVNKYGDELSSVATLTVPCGRICLVELQKVNGKLWFHKGWHEFVECYSIRVGYFLVFIYEGKSNFNVHMFDLTVSEIKNPCNSLSQLQESSHDNPCLLPNEKDDGLEKVLGFRPPSPNLLSSITSKNCNEYIHCNWIQSTSTASLEKPHVRTDVYNMRENFQSSRDIGTQFNGMELTSTEDGAGSVIPGITRKTRGRKRMSENSIQNVKLKSTHVRNTSETLTRRRRAVTPEEKERTIRAAHMFRSDNPFFRVILRPSYVYRGFLLHIPSSFARTFLNTVTGFVTLQVSDGKQWPVRCSFKDGKAKLGQGWTEFVWENNLEEGDVCIFELIHAKEIVLKVAVFRVLEDAAPTGQLSN

>PtVRN1c Potri.004G147200.1 *Populus trichocarpa*

MAPRKEVRPTRSLQADQRPLWHFFKIITQSTLKDKKLRIPNKFARKFGEELSDVAKVVLPNGHSWQIGLTKSNNSVSFDDGWLQFLEQHSVGYGHLLVFGYRGCSNFNALIFDKTACEIPYHRCRGGTSGGKINYDEKCSPYDVDVMKDEGTIASIDSQYCCALESGVFDEDAGDSRQRHPSKPPPSENNAQERPCFECSGDKRGKIPVKKELIVMAELDDTDESKRRKLSKKCRLPRPHGSLIDETKVNKGKSKTKFAPSYADETDFSPRCGEDTDIIVCGFAKASEESKKAIHAARMFRPKNPSFMVLLRSYNKCFVAVPAEFSKRHLSGVSEHIKLQVSDGRQWPLRLNKTQRARMIISRGWNEFKRENNLKEGDVCVFELIKNKKFSLQVSMFRAVDGSGPSN

>PtVRN1d Potri.007G035700.1 *Populus trichocarpa*

MASCSKRDNNGSLFTPKKTHFIRIILSDIMKNQKIAIPRRFLRKHGESLSKSAVIKVPSAATWKVEFLKHKDEVSFAKGWEMFIAYYSIDYGDFLVFEHEWNSLFTVFIIDKSFTEVGYPWNNTDAAESNQEDKLEDHEDDDDDDASVEFIDISSGSQETGKGTKGKEKLGCSRNSEEYEGDHGGRPAENKCHPSGTPLSAARDSPGFYFTWTAKCTTKSAIKRKEE

>PtVRN1e Potri.009G108500.1 *Populus trichocarpa*

MPRPFFQKLILSSTIREKRLRIPDNFVKKFGHDISSFVRLIVPGGHVSRIGLIKADDKLWFHDGWQQFVERFAIHVGYFLIFRYEGNAIFNVHIFNLPTSEINYHSNSLSGKRYLAFEELDNDENAENSGIPPPQLIVNKSYNPPSLQNLFSGSKLNNCINWSGEDTMRLTKGSNISQVANESARNVGAHYNELYNSQDEVKLYSPDGETPKPKKRGRKRLKADPNEQQLSSPHDDDSEMRFRFYESASARKRTVTAEERERAMNAAKAYAPDNPYCRVVLRPSYLYRGCIMYLPSGFAEKNLNGVSGFIILQLPDGKQWPVRCLYRGGRAKFSQGWYEFTLENNLGEGDVCIFELLKSRDVVLKVTLFRVLEDGGLMNHP

>PtVRN1f Potri.010G191500.1 *Populus trichocarpa*

MSSRKEGGPSRSRDLRSHSQIESPLCHFFKIVFPSTLKDKKLRIPRKFVEKFGEGLSDIAKVAVPNGNEWQVGITKEHNNIWFDEGWQEFVEHHSIGSGYLVVFRYRGDSNFSVLIFDMTACEIQYRRMRPTGGEGMNDAEKCSFYDEDEMKDEGSVESLDTHYCRALKSRVFNLNAREGGSSKGRGPSSETTVKNEMTYIDDTSESRRGKSLKKHRMSAPHGETKAKKSKSKSRLGENELLPECEAIEFVPRGFAKASEKSKRAIHAARMFKPKSPSFMVMLRRYNFYNHFLYVPLEFAQRHLSDAPRCIKLQVSDGREWPIQINRNQCRYLSISKGWNEFSQENNLKEGDVCVFELINKEKFVLKVAIFHELEDNVPSD

>PtVRN1g Potri.012G093200.1 *Populus trichocarpa*

MTSKEEACPMCTQNCLMTHGNRTNSRTLVSSFFKVLIGGPFLTVLYFPPKFAPMVSSLNGQETFLEDSSGQRWKVKVSILNDSFVLQEGWSAFASDHGLELGDFIIFNYIMGSHFEVHIYDKSACERLDFSERRNQKERTGNNTQNFNTEYGQCLAKDKGSMNVHGSSTPNLAGEEICRGQSKLKNDVLKAKMVAKNTSTNKKETRAEKIVSKAECVEESRDMINRELGKKHEDYGETLVDLSNWGLSKANLGIEGSKKTRAGTKKLSHHAGASFKSQKEAGLGSKDTR

>PtVRN1h Potri.014G057100.1 *Populus trichocarpa*

MGSPFKGGNGSFMFNADKPHFFKIFFKKLLLMGSFLYQGIPKKFVRLYGKGLSNKALLEVPNGTVSEVEFFKSDGKIWLQNGWKEFAEHYSLALGSLLVFEYKKSCHFHVLILDKTTMEIDYSFSMTDGDEEPDLEGEFQQPRTEETDDDPIPKSFAQKYFKNDHGDAVLCVLDGRTWPVKYFVYPGNGKAKTQIRSGWNKFTWDNYLEVSDVCVFELTKCIEMSFKVIVVRANKDDSHRLAVANQAKPKRSVLIDIDPEFPRDGEDGTSSSHHKYGTKEKGRKSEEDDSLPPEKKIKNSLPHKMANNSRKDAGSGLKTKRLAPLSAIEKEKALRRAYAFKSENPFFFIAIQPAYVCSGANMSIPFKFADRYFKEKNGEVILQVSKDPKLW

>PtVRN1i Potri.018G124500.1 *Populus trichocarpa*

MGFSRERERDGSLFKPGEYRFIKRIHDDIPENKPWIPRKFLRKIGNVLSKSATLEDPNGTLWRIELLKSADGMVRFQKGWQDFADFYSLKKGDLLVFEYKGNSRFSVSIYKEMDCPAGSIDSVSSNQFGHFEEDMEDEDYLEFLAKLPKQKPEVSYSFSKPASDSPSCMIIKSGRSRKRLKS

**Associated factors: VAL1/2/3**

>AtVAL1 AT2G30470.1 *Arabidopsis thaliana*

MFEVKMGSKMCMNASCGTTSTVEWKKGWPLRSGLLADLCYRCGSAYESSLFCEQFHKDQSGWRECYLCSKRLHCGCIASKVTIELMDYGGVGCSTCACCHQLNLNTRGENPGVFSRLPMKTLADRQHVNGESGGRNEGDLFSQPLVMGGDKREEFMPHRGFGKLMSPESTTTGHRLDAAGEMHESSPLQPSLNMGLAVNPFSPSFATEAVEGMKHISPSQSNMVHCSASNILQKPSRPAISTPPVASKSAQARIGRPPVEGRGRGHLLPRYWPKYTDKEVQQISGNLNLNIVPLFEKTLSASDAGRIGRLVLPKACAEAYFPPISQSEGIPLKIQDVRGREWTFQFRYWPNNNSRMYVLEGVTPCIQSMMLQAGDTVTFSRVDPGGKLIMGSRKAANAGDMQGCGLTNGTSTEDTSSSGVTENPPSINGSSCISLIPKELNGMPENLNSETNGGRIGDDPTRVKEKKRTRTIGAKNKRLLLHSEESMELRLTWEEAQDLLRPSPSVKPTIVVIEEQEIEEYDEPPVFGKRTIVTTKPSGEQERWATCDDCSKWRRLPVDALLSFKWTCIDNVWDVSRCSCSAPEESLKELENVLKVGREHKKRRTGESQAAKSQQEPCGLDALASAAVLGDTIGEPEVATTTRHPRHRAGCSCIVCIQPPSGKGRHKPTCGCTVCSTVKRRFKTLMMRRKKKQLERDVTAAEDKKKKDMELAESDKSKEEKEVNTARIDLNSDPYNKEDVEAVAVEKEESRKRAIGQCSGVVAQDASDVLGVTELEGEGKNVREEPRVSS

>AtVAL2 AT4G32010.1 *Arabidopsis thaliana*

MESIKVCMNALCGAASTSGEWKKGWPMRSGDLASLCDKCGCAYEQSIFCEVFHAKESGWRECNSCDKRLHCGCIASRFMMELLENGGVTCISCAKKSGLISMNVSHESNGKDFPSFASAEHVGSVLERTNLKHLLHFQRIDPTHSSLQMKQEESLLPSSLDALRHKTERKELSAQPNLSISLGPTLMTSPFHDAAVDDRSKTNSIFQLAPRSRQLLPKPANSAPIAAGMEPSGSLVSQIHVARPPPEGRGKTQLLPRYWPRITDQELLQLSGQYPHLSNSKIIPLFEKVLSASDAGRIGRLVLPKACAEAYFPPISLPEGLPLKIQDIKGKEWVFQFRFWPNNNSRMYVLEGVTPCIQSMQLQAGDTVTFSRTEPEGKLVMGYRKATNSTATQMFKGSSEPNLNMFSNSLNPGCGDINWSKLEKSEDMAKDNLFLQSSLTSARKRVRNIGTKSKRLLIDSVDVLELKITWEEAQELLRPPQSTKPSIFTLENQDFEEYDEPPVFGKRTLFVSRQTGEQEQWVQCDACGKWRQLPVDILLPPKWSCSDNLLDPGRSSCSAPDELSPREQDTLVRQSKEFKRRRLASSNEKLNQSQDASALNSLGNAGITTTGEQGEITVAATTKHPRHRAGCSCIVCSQPPSGKGKHKPSCTCTVCEAVKRRFRTLMLRKRNKGEAGQASQQAQSQSECRDETEVESIPAVELAAGENIDLNSDPGASRVSMMRLLQAAAFPLEAYLKQKAISNTAGEQQSSDMVSTEHGSSSAAQETEKDTTNGAHDPVN

>AtVAL3 AT4G21550.1 *Arabidopsis thaliana*

MLSSSSMSSSSLSARFCFNHECFEFKLDHCRPGWRLRSGDFVDLCDRCASAYEQGKFCDVFHQRASGWRCCESCGKRIHCGCIASASAYTLMDAGGIECLACARKKFALGPNFSPSPSFLFQSPISEKFKDLSINWSSSTRSNQISYQPPSCLDPSVLQFDFRNRGGNNEFSQPASKERVTACTMEKKRGMNDMIGKLMSENSKHYRVSPFPNVNVYHPLISLKEGPCGTQLAFPVPITTPIEKTGHSRLDGSNLWHTRNSSPLSRLHNDLNGGADSPFESKSRNVMAHLETPGKYQVVPRFWPKVSYKNQVLQNQSKESESVVTPLFEKILSATDTGKRLVLPKKYAEAFLPQLSHTKGVPLTVQDPMGKEWRFQFRFWPSSKGRIYVLEGVTPFIQTLQLQAGDTVIFSRLDPERKLILGFRKASITQSSDQADPADMHSPFEVKKSAYITKETPGVECSSGKKKSSMMITRSKRQKVEKGDDNLLKLTWEEAQGFLLPPPNLTPSRVVIEDYEFEEYEEAPIIGKPTDVAGSTCTEVEGLLISPTTTKHPRHRDGCTCIICIQSPSGIGPKHDRCCSCAVCDTNKRRRRSLLLRREKKQMEKEDNARKLLEQLNSDNGLHQSANNSENHERHASPLKVQLDLNFKPEKDEESLPGSNKTTKSETLPHDDTVKSSFTSPSSSSAHSQNNKEDEGKLKTTTEIADTTTTSSM

>BsVAL1 Bostr.24513s0191.1 *Boechera stricta*

MFEVKMGSKMCMNASCGTTSTVEWKKGWPLRSGLLADLCYRCGSAYESSLFCEQFHKDQSGWRECYLCNKRLHCGCIASKAMIELMDYGGVGCSTCACCHQLNLNKRGENPGVFSRLPMKPLADRQHVNGESGMNIDGGRNEADLFSQSQRCESQPLVMGGDKREEFIPRRGFGKLMNSENTTTGYRLDAAEMHESSPSQPSLNMGLAVLPYGPSFSTEGIEGKKHIGASQSNMVHCSASNILHKPSKPSISTPPGASKSAQARIGRPPVEGRGKGHLLPRYWPKYTDKEVQQISGNLNLNIVPLFEKTLSASDAGRIGRLVLPKACAEAYFPPISQSEGIPLKIQDVKGKEWTFQFRYWPNNNSRMYVLEGVTPCIQSMMLQAGDTVTFSRVDPGGKLIMGSRKAANAGDMQGCGLTNCTSTEDTSSSGVTENPPSINGSSCPSLIPKELNGMPDNLSSPYGGSSTKKSENNGGRIGDDPTRVKEKKRTRTIGTKNKRLLLHSEESMELRLTWEEAQDLLRPSPSAKPTIVIIEEHEFEEFDEPPVFGKRTIVTTRPSGEQERWASCDDCSKWRRLPVNALLSLKWTCIDNVWDVSRCSCSAPEENLEGLENVLKVGRDYKKRRTGESQAAKSEQEPSGLDALASAAVLGDIIGEPEVATTTRHPRHRAGCSCIVCIQPPSGKGRHKPTCGCNVCSTVKRRFKTLMMRRKKKQLERDGPAAEDKEEKDMEQAESDKNKAETEVNTARIDLNSDPYNKEDVEAVAVEKDESRKIGQCSGVAQNGDVLGVKELEGEAEKISEDPRGSR

>BsVAL2 Bostr.7867s1055.1 *Boechera stricta*

MESTKVCMNALCGAASTSGEWKKGWPMRSGDLASLCDKCGSAYEQSIFCQVFHAKDSGWRECNSCDKRLHCGCIASRFMMEFRDNGGVTCISCAKKSALLSMNVSQESNGRDVPSFASAEHVGSVLERTNLKHLLHFQRIGPTQSSLQMKQEESLLPSRLDTLRHTTERKELQELSAQPNLSISLGPTLMTSPFHDAVVDDRSKTTSIFQLAPRSRQLLPKPANSAPTAAGMEPNGSLVSQIHVARPPPEGRGKTQLLPRYWPRITDQELQQLSGHSNSKIIPLFEKVLSASDAGRIGRLVLPKACAEAYFPPISQPEGLPLKIQDIKGKEWVFQFRFWPNNNSRMYVLEGVTPCIQSMQLQAGDTVTFSRTEPEGKLVMGYRKATNSTATQMFKGSSEPNLNMFSNNLNLGCGDINWSKLEKSEDMGKDNLFLQSSLTSARKRVRNIGTKSKRLLIDSVDVLELKITWEEAQELMRPPQSAKPSIFTLEDQDFEEYDEPPVFGKRTVFVSRQTGEQEQWVQCDACGKWRRLPVDTLLPPKWFCSDNLLDPARSSCSAPDELSPREQDTLVRQSKEFKRRRLASSNEKLNQAQEASAVDTLANAGITTTGEQGEIAVAATTKHPRHRAGCSCIVCSQPPSGKGKHKPSCTCTVCEAVKRRFKTLMMRKRNRGEAGQASQQAQSESRDETEVESIPAVEAAAGGNIDLNSDPGASRVSMMSLLQAAAFPLEAYLKQKAISNTAAEQQSSDMVSTEHGSSSAAQEQDKDTNGAHEPVS

>BsVAL3 Bostr.10689s0100.1 *Boechera stricta*

MLSSMSSSRFCFNHECFEFKLDHYRPGWRLRTGDFADLCDRCASVYEQGKFCDVFHLKASGWRCCESCGKRIHCGCIVSASAFMLLDAGGIECLACTRKKVALGPNFSPSPSFLFQSPVSEKFKDLSINWSSSTRSNQISYQPPSCLGASVLQFDLRNRGDNNEFIQPTSKERATACSMEKKRGMNDMIGKLMSENSKNYKVSPFPNVNVYHPLISIKEGSCGTQLVFPVPITTPIEKTGHSRLDGSNLWHTPNCSPLSRLHNDLNGGADLPFESKSRNVGTHLDTPGKYQVVPRYFPKVSYKNQVLQNQSKESESVVTHLFEKMLSASDTGRVGRLVLPKKCAEAFLPQISHSEGVPLKIQDPMGKEWTFQFRFWPNNNSRMYVLEGVTPCIQSLQLQTGDTVIFSRLDPGRKLILGFRKASVAQSSDQETDSTNNNRDTCANRDAEPADMHSLSKVKNSAYITKETPGVEFSSSKKKSSMMFTRGKRQKVEKGDHIELKLTWEEAQGFLLPPPNLTPSRVVIEDYEFEEYEDAPIFGKPTDDTSFRVPCTADKKLVAEQHDEEAMEEVEGLLMLPKTTTKHPRHRNGCTCIVCIQSPSGTGPKHDRCCSCVVCETVKRRRRSLLLRREKKQMGKEDDAHKELEQPNSDNELCQSANNSENHERHASPLKGQLDLNFKPEKNGEYLPCSNKTTKKKTLHHDDTVKSSFKSPSSSSDHSQINKEDEAELKKNTEIADTTTTSSI

>BrVAL1a Bra022801 *Brassica rapa*

MFEVKTGSKVCMNASCGSTSTVEWKKGWPLRSGSLADLCFRCGSAYETSLFCETFHLEQSGWRDCYLCNKRLHCGCIASKLMVEFMDYGGVGCTSCTNCHQLNLNKRGENPVVFSRLAMNSPHTNGESGISIRSEADLFSQPLVNGDDKREEFMAHRGFANFMKPDNNTNNNNAGEMHEPSQPSLNVALATLPYSPSFANPVGGNKLMAAASQSHSHIGQCSASSILQKPSKSVPGTPPGTSKSAQARIGRPPVEGRGKGHLLPRYWPKYTDKELQQISGNLNLNIVPLFEKTLSASDAGRIGRLVLPKACAEAYFPPISQSEGIPLKIQDVRGKEWTFQFRFWPNNNSRMYVLEGVTPCIQSMMLQAGDTVTFSRVDPGGKLIMGARKAAYTVDMQGCGLTNGTSNEDTSSSGVTENPTSINASSQIPEELKGVPEHLSSPYGVGSSLKKSEMNGGRICDDPSRVKDKKRTRTIGAKNKRLLLRSEELRVTWEESQELLRPSPNAKPTVVVVEDHEFEEFDEPPVFGKRTILTSKPSGEQERWASCDDCTKWRRLPIDALLPAKWTCSDNVWDVSRCSCSAPEESLKDLENVLRAGKEYKKRRIQAAKTEEEPSGLDALASAASAAVLGDALGGDSEVATTTRHPRHRVGCYCIVCIQPPSGKGRHKPTCGCTVCSTVKRRFKTLMMRRKKKQLERDEITAAAEAYEEHNNKEAAERGDTDENNGEKEGRIDLNSDPYNREDVEAEKEDTKGRECSGVADEVLGLTELGGEAASCEELKAAT

>BrVAL1b Bra018313 *Brassica rapa*

MGSKVCMNASCGSTSSVEWKKGWPLRSGALADLCFRCGSAYETSRFCETFHMEQSGWRECYLCNKRLHCGCIASKLVVEFMDYGGVGCSTCTNSHSKRGENPGVFSRLPMNMQQTNGESGMSIDGVVRSEANLFSQPLVSGDDKREEFTPHRGFGNLMKQDNNVTTTGYTHESSSSSPAQPSLNMALATLPYSPSFATPVVDGNKLMGAGGGGVASSQSRLFQCSASSILQKPSKSVLGTPPPGTSKSAQARIGRPPVEGRGKGHLLPRYWPKYTDKELQQISGNLNLNIVPLFEKTLSA SDAGRIGRLVLPKACAEAYFPPISQSEGIPLKVQDVRGKEWTFQFRFWPN NNSRMYVLEGVTPCIQSMRLQAGDTVTFSRVDPGGKLIMGARKATYTVDMQGCGFANGASNEDTSSSGVTENLTSINAPSCPSQMLEGLPEHLGSPHGGNGVKKSEINGGDDQSRGKEKKRTRTLGAKNKRLLLHSEESMELRVTWEEAQELLRPSANAKPTVVVIEEHEFEEFEEPPVFGKRTIVVTSRPSGEQERWGSCDDCSKWRRLPVDALLPAKWTCSDNVWDSSRCSCSAPEESLKELENVLRAGKEYKKRRIGVSQTARNEQEPSGLDALASAAVLGDALDESEVATTTRHPR HRVGCSCIVCIQPPSGKGRHKPTCGCTVCSTVKRRFKTLMMRRKKKQLEC DGTAAVDEENKEGVEPEKNEGEKEGRIDLNSDPYNREDAEAVAVEKREESKKSEGGVSWGVSQGGGVLGETEVGGGEAEKTTSEEQKVTS

>BrVAL1c Bra021635 *Brassica rapa*

MCMNASCGSTSTVEWKKGWPLRSGALADLCFRCGSAYETSLFCETFHLDQSGWRECYLCNKRLHCGCIASKLVVEFMDFGGVGCTTCTNCHQPNLNNRGENPGLFSRLPMNRQHTNGESGMNVGKADLFSQPLVQGDDKREEFMPHRGFSNLMKPDNATTTGYRHDANGTHESSPTPSQPPSLNMPMAALPYSLNFATATKKLMDAASQSHTVQSSASGILQTPSKSVPGTPSGPGTSKSAQARIGRPPNEGRGRSHLLPRYWPKYTDKELQQISGNLNLNIVPLFEKTLSASDAGRIGRLVLPKACAEAYFPPISQSEGIPLKIQDVRGKEWTFQFRFWPNNNSRMYVLEGVTPCIQSMMLQAGDTVTFSRVDPGGKLIMGFRKAAHTGVIQGCGLTNGTSNEDTSSSGVTETPTSVNASSCPAQTPEELKGLPEHLNSNHGGSSLKKSEVNGVKDKKRTRTVGAKHKRMLWRSEEAMEVRITWEETQELIRPSPSEKPTVVVIEEHEFEEFNEPPVFGKRTIVTSRPSGEQERWGSCDDCSKWRRLPVDALLPAKWTCSDNVWDESRCSCSAPEESLKELENVLRMSKEYKKRRSGVSQTVRTEEELTGLDALASAAALGDTLGEEEEPATTTRHPRHRVGCSCIVCIQPPSGKGRHNSSCLCTVCSTVKRRFKTLMMRRKKKQLEREEIEAAVAAAADQENKEDGKTGRIDLNSDPCNRGDVEAVGVEEKDESEKGEAGGCLGVAQADDVVGVTELEGEGEKIGEESKGSS

>BrVAL2 Bra023968 *Brassica rapa*

MESTKVCMNAQCGSTSTSGEWKRGWPMRSGELASLCDKCGSAYEQSIFCQVFHAEESGWRECNSCDKRLHCGCIASRFMMEVVDNGGVTCISCAKKSGLFSMNVESNGREFPTFASAEHVSSVLERTNLKHLLHFQRISPTQPFLQMKQEESLLPARLEALRHNTEKKESAQPNLSISLGPTLMTSPFHDVDDRSKTTTPIFQLASRSRQLLPKPANSAPTTAPPMEPNGSLVSQIHVARPPPEGRGKTQLLPRYWPRITDQELQQLSGQYPHLSNSKIIPLFEKVLSASDAGRIGRLVLPKACAEAYFPPISQPEGLPLKIQDIKGKEWVFQFRFWPNNNSRMYVLEGVTPCIQSMQLQAGDTVTFSRTEPEGKLVMGYRKATNSTASQMFKGSSEPNLNMFSNNLSSGCGDINWSKLDKPEDMSKDGLMLQPSLISARKRVRNIGTKSKRLLIDSVDVLELRLTWEEAQELLRPPQSAKPSICTVEDHDFEEYDEPPVFGKRTVFVSRQTGEQEQWVQCDACAKWRRLPVDTLLPPKWLCSDNLLDPGRSSCSAPDELTPREQDTLLRLSKEFKRRRLASSNQEEASALDTLANAAITTTGEQGETEVAATTKHPRHRAGCSCIVCSQPPSGKGKHKPSCTCTVCEAVKRRFKTLMMRKRNRGEAGQASQQAQSDQCREETEAESIPAVELPAAGGNIDLNSDPASRVSMMSLLQAATFPLEVYLKQKGVPNTAADQQSSDIVSTENG SSSAAQEHDRDTSGAPEPMN

>BrVAL3 Bra013535 *Brassica rapa*

MTPSSYSARFCFNRECPDFNRECYRPGWRLRNGDFADLCNRCATAYEQGRFCDIFHQRASGWRCCESCGKRIHCGCVVSAPAFTLLDAGGIECLTCARKKVSVSPIADKFQDLSIDWNSSTRSYRPPNLSGPSILQSDLHNRGDCYEFNQPTSKDKATAYSTEKHRGMNDLMGRLMSVNSNNHTNSILYNQKAGPNCKVPTCPNVNAYPPLISLKEGPLGAQRAFPVTTPVETNGHLGLGGRYLWHKDNSSPLSHLHNDVNRGADSPLESKNWNFGIHLDTPGKYQVVPRYSPKIPYKNQVLQNLSNESVSVVTPLFEKILSVSDAGRVGRMVLPKKCAEAFLPQISQTDGVPLTVHDSTGKEWTFQFRFWVNNNSRMYFLEGITPCIQSMQLQAGDTVIFSRVDPEKKLIMGFRKASVAQSSVQETELNNNRESCTNGDAEPIDIHPPNGKKKSSMTTTRSKRQKVEKGELSEVKLTWEEAQGFILPPPNLTPSIITIEGIEFEEYEDAPIIGKPNTGFGSTCSANGRLLAEQDDEEAKDEAEGLLMSPKSSSKHPRHRNGCTCIVCLQSPSGSSPKHGRRCSCTVCDTVRRRRETLLQRKKKQQQIEVENKTHKELESPNSDEERHQSANNSGTTSKDHEPSKAQIDL NFQPEKDEESPPPRSKTTTKDKSLHHDEASFKPPRSSSSAHNKLHVESF

>CgVAL1 Cagra.0567s0011.1 *Capsella grandiflora*

MFEVKMGSKMCMNASCGTTSSVEWKKGWPLRSGLLADLCQRCGSVYESSTFCEKFHKDQSGWRECYLCNKKLHCGCIASKVMIELMDYGGVGCSTCACRQKLNFNNSGENPGVFSRLPMKSLADRQYVNGESGMNIEGGRNEADLFSQPQRCESQPLVMGGDKREEFIPHRGFGNLMSSENTNTGFRLDAVEMHESSPAQPSLNMGLSVLPYSPSFSTEGIEGKKHIGASQSNMVHCSASNILQKPSKPAMSTPPVASKSAQARIGRPPVEGRGKGHLLPRYWPKYTDKEVQQISGNLNLNIVPLFEKTLSASDAGRIGRLVLPKACAEAYFPPISQSEGIPLKIQDVKGKEWTFQFRYWPNNNSRMYVLEGVTPCIQSMMLQAGDTVTFSRVDPGGKLIMGSRKAANAGDIQSCGLTNGTSTEDTSSSGVTENPPSINGSSCPSLIPKESNGMPENLSSPYGGNGTKNENNGVRVADDPTRVKEKKRTRTIGTKTKRLLLHSEESMELRITWEEAQDLFRPSPSAKPTIVVIEGHEFEEFDEPPVCGKKTIVTTKPSGEQERWAACDDCSKWRRLPVNALLSLKWTCIDNVWDVSRCSCSAPEESLKELENALKAGREYKKRRTGESQAAKSEQEPSGLDALASAAVLGDTMGEPEVATTTRHPRHRAGCSCIVCIQPPSGKGRHKPNCACTVCSTVKRRFKTLMMRRKKKQLERDGPAAEEKEKKDMEQAESDKNKAEEEVNTARIDLNSDPYNKEDVEAVAGEKDESRKRKIGQSSGVAQEGDVVGVKVLEGEAEKIGEDPKGSS

>CgVAL2 Cagra.12385s0007.1 *Capsella grandiflora*

MESTKVCMNALCGASSASGEWKKGWPMRSGDLASLCDKCGSAYEQSIFCQVFHAEESGWRECNLCDKRLHCGCIASRFMMELLDNGSVTCISCAKKSALFSMNVSQESNGRDSSFASAEHVGSVLERTNLKHLLDFQRIGPTQSSIQMKQEESLLPSRLDALRHKTERKELQELSAQPNLSISLGPTLMTSPFHDAVIDDRSKTTSIFQLAPRSRQLLPKPANSAPTAAGMEPNGSLVSQIHVARPPPEGRGKTQLLPRYWPRITDQELQILSGHSNSKIIPLFEKVLSASDAGRIGRLVLPKACAEAYFPPISLPEGLPLKIQDIKGKEWVFQFRFWPNNNSRMYVLEGVTPCIQSMQLQAGDTVTFSRTEPEGKLVMGYRKATNSTATQMFKGSSEPNLNIFSNNLNSGCGDISWSKLEKAEDMGKDNLFLQSSLTSSRKRVRNIGSKSKRLLIDSVDVLELKVTWDEAQELMRPPQSAKPSIITLENQDFEEYDEPPVFGKKTVFVARQTGEQEQWVQCDACGKWRRLPVDTLLPPKWLCSDNHLDPARSSCSAPDELSPREQDTLVRQSKEFKRRRLAASNEKLNQSQEASAVETLANAGITTTSEQGEIAVAATTKHPRHRAGCSCIVCSQPPSGKGKHKPTCTCTVCEAVKRRFRTLMMRKKNRGEAGQASQQAQSESRDETEVESIPAAEAASGDNIDLNSDPGGSRVSMMGLLQAAAFPLEAYLKQKAISNTGGEQQSSDMVSTEHGSSSAAQEQEKDTNGVHEPVS

>CgVAL3 Cagra.3519s0007.1 *Capsella grandiflora*

MFSSSSMSSSSSSFSSRFCFNHECFEFKLDHCRPGWRLRTGDFVDLCDRCASLYEQGKFCDVFHQKASGWRCCESCGKRIHCGCIVSASAYMLLDAGGIECLACARKKVALGPNFPPSPSFLFQSPISEKFKDLSINWSSSTRSNQISFQPPSCLGPSVLQFDLRNRGDNNEFSQPTSKERATACSMEKKRGINNLIGKLMSENMQKYKVSPFPNGPNINVYHPCGTQLAFPVPITTPIEKTGHSRLSGSHLWQTPNCSPLSHLHYDLNGGADSLLESKSRNVRTQLDAPGKYQVVPQYWPKVSNKNQQNQSKESESIVTHLFEKILSASDTGRVGRLVVPKKCAEAFFPQISHPEGVPLKIQDPMGKEWTFQFRFWPNNNSRIYVLEGVNPCIQSLGLQTGDSVIFSRLDPERKLILGFRKAAIAQSSDQETNSTNNNRDTCTNRDAEPADMHSLSEVKKSTYITKETPGVEFSSGKKNSSMMITRGKRRKVEKGDHIELRLTWEEAQGFLLPPPNLTPSKVVIDDYEFEEYEDAPIIGKPIDDTGFRSTCTPDQKLVAQQQNEEAMEEAERLLMSPKTTTKHPRHRNGCSCIVCIQSPSGAGPKHDRRCSCAVCEAVKRRRHSLLLRREKKQMGKEDNAHKELEQPNSDDELQQSANNSENHECHTSPLKVQLDLNFKPEKDGESLPDSNKTSKKKTLHHDDTLNSSFNSPSSSTAHSQINREEEELKKNTEIADTTTSSI

>OrVAL1 orange1.1g039386m *Citrus sinensis*

MGPRICMNPKCRTANTHEWKKGWLLRSGVCADLCYDCGSAYENFIFCNTFHLEEPGWRECNFCSKRLHCGCRASNSFLELLDYGGVGCRSCAMSPRLHLIQRDEIPNGFGALTKKDSDDTQTPMLENRVVGDGTAEGKLTQLCRIMEANEPSFLAPFQRGDTIVSLGQEKREELRLPFVEVGTGFSSPTKLSSRSSKFTKPDGSRSMLDVRDMPESLAQRSSSMSLGVPAGCSNFVPPFSNGAADGREPCKAHPSFQQGQRSRPILPKPSKTGLTISSETKKSTASQLRIARPPAEGRGKNHLLPRYWPRITDQELQQLSGDLNSTIVPLFEKILSASDAGRIGRLVLPKACAEAYFPHISQSEGVPLRVQDVKGKEWVFQFRFWPNNNSRMYVLEGVTPCIQSMQLRAGDTSMIHSILTRDDKITFSRIDPGGKLVMGFRKAPIPGDMQDAQTSAITNGCPGESFLSGVTENLPTVSGYSGHFQMLKGSKDPHIDALSEHLSLAEGGNGWHKSENHGQKTNEDSPQKSLLGMEKKRTRNIGSKSKRLLMHSEEAMELRLTWEEAQDLLRPSPSARTKHCHY

>OrVAL2 orange1.1g002708m *Citrus sinensis*

MESRTCMNGKCRASSSIEWRKGWPLQSGGFAVLCDKCGSAFEKLIFCDEFHSKDSGWRKCASCSKRLHCGCIASLSLIQLLDGGGVWCINCAKNPGLDSIPGDDPNGFGTLKTDNAGDLPSTSVDNQLGGSDDKFKLLQLGNSSESVGLRHLLQFRNDDLDGSFRKVKPEEAAKSDISKANIGAKDIYGPLAHTNLSITLGSPGINSNSFPSAVVDEKEHSKTSAIIHQGPKSRHLLPKPPKLALATGSEANAGISQIRVARPPAEGRGRNQLLPRYWPRITDQELQQLSGDSNSTIVPLFEKVLSASDAGRIGRLVLPKACAEAYFPPISQPEGLPLRIQDVKGKEWVFQFRFWPNNNSRMYVLEGVTPCIQSMQLQAGDTVTFSRMDPEGKLVMGFRKASNAVSVQDTQPSAIPNGGHSSESFFSGVFENLSILSGYSGVLQSLKGSTDPHLSALSKQLNSPPGDINWVKSEKHEDKTREGLLPPSMLVPERKRSRNIGSKRKRLLIDRLDVLELKLTWEEAQDMLYPPPSVMPSIVTVEDHVFEEYEDPPVFGKRSIFIVRTSGGQEQWAQCDGCSKWRRLPVDVLLPPKWTCMDNVWDHNRCSCSAPDELTPREVENLLRLNKDFKKRKIATSHRLNQEHEPSGLDALSNAAILGENMGDPGTASVATTTKHPRHRPGCSCIVCIQPPSGKGKHKPTCTCLVCMTVKRRFKTLMMRKKKRQSEREEEVAQRNQPTWGPKEEAEVDSSSKHVSSHLDPSENEARSANELESKSQNNNLSGKLAESSKAELDLNCHPDREEVQAGLNRVSMMKLLQVASHPLETYLKQNGLTSLTSDQQASSGTHAPPQAAGESEGQLNELASATQERESGGEDNCEPVSDQIPDDPE

>OrVAL3 orange1.1g002489m *Citrus sinensis*

MKHKTHPCNPVALSFISVSVFIFNLDLTCLSCNYCYLVLQILNIRCFVFCAAILTLILCVYRSIYEEGRFCDTFHVNASGWRCCESCGKRVHCGCITSVHAFTLLDAGGIECMTCARKNVLVAPTPSWPPSLFYQTPFPERIKDLSVKNWTQLAGSGPVPWRQAPSLFNSSIPQPELRPRLPYEVDLSAGIDRINASERLSVPSLEKRKLEDFSERLMNGGLKSGSRDIPENANAGWNQFRASRSNCDMQPSSCLNKPQQSSTLKDDSSTPHFGLAVSYASPSETNSQIGVSGSHLRPVVQPPLVKQFHGNLPNGADSLGETQVRNGRPRVDARGRSQLLPRYWPRFTDQDLQQISGDSNSVITPLFEKMLSASDAGRIGRLVLPKKCAEAYFPPISQPEGLPLKVQDSKGKEWIFQFRFWPNNNSRMYVLEGVTPCIQNMQLQAGDIVTFSRLEPEGKLVMGFRKASSASASDQDNEANKAGTGIPANGHAELADPSSWSKVDKSGYIATEALGAKSSISRKRKNTTLGSKSKRLKIENEDVIELKLTWEEAQGLLRPPPNNVPSVVVIEGYEFEEYEDAPILGKPTIFATDNVGEKIQWVQCEDCSKWRKVPANARLPSKWTCSGNLWDPERSVCSVAQELREEQLEDLIAPNNPASSKKLKAAKQEPDCVEALEGLDTLANLAILGEGEGLTASSQATTKHPRHRPGCSCIVCIQPPSGKGPKHKQTCTCNVCLTVKRRFHTLMLRREKKQSEKDAETSRKKQQQQKLPVPEKSADDDPLSCSKTGNNSPNEKKVVSEGSDDDSSRIKSSTSPFKGQIDLNIQPEREEELSPGSDSGSMIRLLQDATEKYLRQQRLSSSGVNTSSVDNEGLQGGVTGEKISNGITLDGSHQDTDEDHHGSLSVKASASISATG

>CsVAL1 Cucsa.111990.1 *Cucumis sativus*

QIIDVLTTGLPKWQFNYLIDNYFLQEFEVLLASHHDVCAVIAEFLLHLPFRDKGHFWWFERIRRDEISNGFDAVTGGNVGLLRPASVKDQVVGNGINEEKLLQLCNIMEANEPDHFQQSQRVDRSASPTQNRGENLRNPFGEVGSSFFNMNKIPVNCQPSVGSFTYSKLDTSRPHLELKDMKESLTQPSLSITLGVPLGSAAHEDEKSILPFQQGQRSRPIFPKLIKTGTTVNSEARKGMAPLVRIARPPAEGRGKNQLLPRYWPRITDQELEQLSGDLNSTIVPLFEKVLSASDAGRIGRLVLPKACAEAYFPPISQSEGLPVKVQDVKGNEWTFQFRFWPNNNSRMYVLEGVTPCIQSMQLRAGDTGNLSIIPQGLNKVSALQGSSPIIDSNLLLNPTQFNMKVLVVVLIRFHKKSFFTQI

>CsVAL2 Cucsa.177010.1 *Cucumis sativus*

MALRTCMNVSCRASSSTEWRKGWALRSGDFATLCDKCGSAYDQSIFCDIFHLKDSGWRECTSCGKHLHCGCIASKFLMELHDSGGVNCISCAKSLGLHTTSTSEKLNGLGTSQVQNSGDLQSFLVEDGNNDRITLIQSGNKAEGNELRQSHLSPSNNKNVSLVQMKHEENCPSVRDVGYVCSSESTQVTNGLNEFAKQEICKGDSGTKLAYDSLALGCGNSNPLPGVSIDEKEASKPSSPLLLGSRSRHLIPKPARSVFNVGLESNTSMVSQLRVARPPAEGRGRNQLLPRYWPKITDQELQQISGASNSTVVPLFEKMLSASDAGRIGRLVVPKACAEAYFPPISQPEGLPIRIQDVKGKEWVFQFRFWPNNNSRMYVLEGVTPCIQSMQLQAGDTVTFSRMDPEGKLIMGFRKASSSSIMQDSHPSALSTSGHSSEFFSSVFENLPLLSGHSSLLQSLKGNMDPQLNLLPGYFNQPGNDTNWQKAEKQDDQSRECLLLSSMMVPERKRTRNIGSKSKRLHIDCQEALELRLTWEEVQDLLCPPPTVKPSKFMVEDHEFEEYEEPPVFGKMSIFVVHSTRGQEQWAQCDDCSKWRRLPIDVLLPSKWTCLENIWDQSRSSCSTLEELTTRELENILRLNKEFKRQRTLAFSGPIQDHESSGLDALANAATLGDNGSDPGTASVATTTKHPRHRPGCSCIVCIQPPSGKGKHKPTCMCNVCMTVKRRFKTLMMRKKKRQSEREAEIAQKNQLKWSSREESELDNTSRHASLNLDPSANEAQLMTNEPRSQSNLVETCKDQLDLNCQPDQENEVEGVPNRVSMMSLVQIASLPLETYLKQNGLTSLLPELQASSASHAPAQGTNEMEGAVNDDGCFASAAQDQESEGEQICGKDQS

>CsVAL3 Cucsa.045980.1 *Cucumis sativus*

MSSALTTASSSSSPLSSKSCYNSDCKELRPDRSRKGWRLRTGDFAELCDRCASAYEEGRFCETFHLNASGWRCCESCGKRVHCGCIVSAHAFTLLDPGGIECMTCARKNVILPLNPAWPPSLLFHSALPDRLKELSVKNWSQLAGSGPVPWRQAPSMFNSSLPSGELHHRAPYEVDISAALNKLNTSERLPVSLEKRKNEDFSERFLNGSLKPCGQDLCENGTAGGIKCDDKPSSCSNMPKQSSFVKEDSSTMQYGLNIPYAPPNEPSARGRISGTHLRPTPLSSLPKQIHTNLQNGADSSNETQLRNGRPRGESRGKNYLLPRYWPRFTDQELQQISVDSNSVITPLFEKMLSASDAGRIGRLVLPKKCAEAYFPSISQPEGLPLKVQDAKGKEWIFQFRFWPNNNSRMYVLEGVTPCIQSMQLQAGDTVTFSRLEPEGKLVMGFRKASATADQENETNKTKNGAPVHGDVSNIKVKAELADPNSWTKVDKSGYIAKEVLGAKPSISRKRKNSTLGSKSKRLRIDNEDMIELKITWEEAQGLLRPPPNQVPNILVIEGFEFEAYEEAPVLGKPSIIPPDNTGERIQWTQCEDCLKWRKLPASALLPSKWTCSDNSWEPERSFCSAPQELSTEQLEELLSPGNSVAPVKKMKAAKLEPDNVEALEGLDTLANLAILGEGEASQTPGQATTKHPRHRPGCSCIVCIQPPSGKGPKHKQTCTCNVCLTVKRRFRTLMLRREKKQFEKEAETMRQRHKFQDEMFPDRSMDEESLTCSNTSTSKLMEEGKMNDGSDEDPNRNKPSTSPFKGQIDLNMQPEREEELSPGSDSGSMMKMLQDTGDRFLEQQRSNSGGTRSSSSDPLEPGGEREHKGESSSNVIDLSSNNLDADKDHPATLSLNPSASMSTTG

>EsVAL1 Thhalv10016263m *Eutrema salsugineum*

MFEVKMGSKMCMNASCGSTSTVEWKKGWPLRSGALADLCYRCGSAYESSLFCETFHMDQSGWRECYLCSKRLHCGCIASKLMVELMDYGGVGCTTCASCHQLNLNKRSENPGLFSRLPLKTLAERQHINGESGMSIDGGRNEADLFSQSQRCDSQPLVPGVDKREDFMPPHRGFGILKSENSTGYRLDAGEMHESSPLQPSLNMALAVHPYSPSFASTPVEGKKHIGPSQSHIVQCSASSILQKPSKSVLGTPPGTSKSAQARIGRPPVEGRGKGHLLPRYWPKYTDKEVQQISGNLNLNIVPLFEKTLSASDAGRIGRLVLPKACAEAYFPPISQSEGIPLKIQDVRGKEWTFQFRFWPNNNSRMYVLEGVTPCIQSMMLLAGDTVTFSRVDPGGKLIMGSRKAAHNTGDMQGYGLTNGTSNEDTSSSGVTENPSSINASSCPSQIPEELKGLPENLNGGSCSKKSEINGGTMCDDPPRPKDKKRTRTIGAKNKRLLLHSEESMELRLTWEEAQDLLRPAPSAKPTIVVIEEHEFEEFDEPPVFGKRTIITSRPSGEQERWASCDDCSKWRRLPVDALLSVKWTCSDNVWDVSRCSCSAPEESLKELENVLRAGKDCKKRRIGVSQTAKTGQEPSGLDALASAAVLGDALGEPEVATTTRHPRHRVGCSCIVCIQPPSGKGRHKPNCGCNVCSTVRRRFKTLMMRRKKKQLERDGPAAAAAEDQENAEAKQGDSDKSKAEEEVKTGRIDLNSDPYNREDIEAVAVEKEGSEGREVGQCSGVAQGGDVLGVTELVGEAEKISEEPKGSS

>EsVAL2 Thhalv10024481m *Eutrema salsugineum*

MESTKVCMNAQCGAASTSGEWKRGWPMRSGELASLCDKCGSAYEQSIFCQVFHAEESGWRECNSCDKRLHCGCIASRFMMELLDNGGVSCISCAKKSGFFSMNVNHEANGRGFPSFASPEHGTNLKHLLHFQRIGSTQSFFQMKQEDSLLPALRHKTEKQELSAQPNLSISLGFTSPFHDAVVDDTSKATPIFQLAPRSRQLLPKPANSAPTSAGMEPNGSLVSQIHVARPPPEGRGKTQLLPRYWPRITDQELQQLSGQYPHLSNSKIIPLFEKVLSASDAGRIGRLVLPKACAEAYFPPISQPEGLPLKIQDIKGKEWVFQFRFWPNNNSRMYVLEGVTPCIQSMQLQAGDTVTFSRTEPEGKLVMGYRKATNSTAAQMFKGSSEPNLSMFNNNLSPGCGDINWSKLEKPEDMAKDNLLLQPSLTSARKRVRNIGNKSKRLLIDSVDALDLRVTWEETQDLLRPPQSVKPSICTVEDYDFEEYDEPPVFGKRTVFVSRQTGEQEQWVQCDACGKWRRLPVDTLLPPKWLCSDNVLDPGRSSCSAPDELSPREQDTLIRLSKEFKRRRLASSNEKLNQEQEASALDTLANAAITTTGGQGDTAVAATTKHPRHRAGCSCIVCSQPPSGKGKHKPSCTCTVCEAVKRRFKTLMMRKRNREEAGQASQQAQSECRDETEVESIPAVEAAAGGNIDLNTDPGASRVSMMSLLQAATFPLESYLKLKAIIPNTAAEQQSSDMVSTEHGSSSAAQEHDRDTSGAPESLN

>EsVAL3 Thhalv10024526m *Eutrema salsugineum*

MSSSSSLSSRFCFNRECSEFKLEHYRPGWRLQTGDFADLCHRCASAYEQGKFCDIFHQRASGWRCCESCGKRIHCGCIVSASAFMLLDVGGIECLACARKKVALGPNYLPQLPIFFQSPIAEKFKDLSINWNSSTGSNQISCRPPSFLGQSVLQFDLHNRGDSYEFSRPTSKDRATACSIEKKWGMNDLMGKLMSEKSKSHTSDILNNQNAGPNCEVLPSSNVNVYRPLISLKEGPCGTQLAYPVPLTTPIETNVHSRLNGRSLWHTPKSSSLNFLRNDLNGGADSLCSLTHLDTPGKYQVVPRYWPKVSYKNQVPQNLSKESESVVIPLFEKMLSASDTGRVGRLVLPKKYAEAFLPRLSNTEGVPLKVQDSMGKVWTFQFRFWPSNNSRIYVLEGVTPCIQSMQLQAGDTVIFSRVDPERKLIMGFRKASVAQSADQETDSTNNNRDSCTNGDGESIDNHSPSRAKKSAYKAKETPGVGFSSRKKKSSMMITRSKRQKVEKGDHTELKLTWEEAQGFILPPPSLTPSIIKIEGFEFEEYKDAPIIGKPTTGSTCSANKKLLAEQYDEEAMEETEGLMMSPKETTKHPRHRRGCTCIVCIQSPSGTSPKHDNCCSCTVCDAVKRRRRSLLLGRKNKQIEKENKAQKELESLNSDEELHQSANNSGTTSKSHEHDASPQKGQIDLNFQPEKEEESLHKTTKDKSLHHDDTSFKPSSSSSAHSQIEKQD

>GrVAL1 Gorai.003G152700.1 *Gossypium raimondii*

MTLKGDLILIFDFDLSSLCWMGSKICMNSSCGAATSNEWKKGWPLRSGGFAHLCYPCGSAYEDGVYCDTFHLEESGWRECRICGNHLHCGCIASNYLLELLDYGGVGCISCAKSSRLHTVKRIQTHGDEIPEGVGAVPMNNAGSSAVEGKAVSDHVDERTLAPLCKSMEANECNLLPQSQRGDANASLGQHRGEEVICPTEKVGAGFSNATQPYVRPANFAKLDNARSALDVRDIQDSLPQPYLSMSLGGSSANPNFLLPFSSGLADGKEPSKTWSSFQQGQQSRRILPKPSKNGLTTSSELNKGMIPQARITRPPVEGRGKNHLLPRYWPRITDQELQKLSGDLKSTIVPLFEKVLSASDAGRIGRLVLPKACAEAYFPPISQSEGLPLRIQDVNGKEWTFQFRFWPNNNSRMYVLEGVTPCIQSMQLRAGDTVTFSRIDPGGKLVMGFRKAANSDTQTPKGGKDLLGNALSEHFGLPDGNISRSRDEDHGVRANENSVHQLAMNAEKKKTRNIGSKSKRLLMHSVDALELRLTWEEAQDLLRPPPSVRPSIVTIEDHEFEEYDEPPVFGKRTIFGAWPSGEQEQWAQCDDCSKWRRLPVDVLLPSKWKCSDNVWDSSRCACSAPEETGPKELENILKVGTDLKKAKVQESPKVAPEPEPTGLDALASAAVLGDKMGDVGESSVGATTKHPRHRPGCTCIVCIQPPSGKGKHKANCTCNVCLTVKRRFKTLMLRKKKRQSEREAEISERDNKKQMDETELKDTRNDHSENEGSRSRIEGEVAEASTGEIDLNCHPIREDVQLEGGLNMMSFVEATSLPVENYIKQNGIESLKTEQQQQGSLGSHFQSKVNRENERPLSDEEFLASVGWEHVGHKEPCLERNSLQ

>GrVAL2a Gorai.005G004300.1 *Gossypium raimondii*

MALKSCMNVLCGASTSIEWRNGWTLRSGDFANLCDKCGSAYEQSIFCDVFHSKDSGWRDCNSCGKPLHCGCIASRFLLELLDGGGINCVSCAKKSGFNHMIEDEKPNGFGMVKADAGQLHSTSADNQLSGASIENLQLMQLSNNAESIGLRQLLQLHNDDSSGSLGQMKQDEFLPPPREIGSTCLTSINQASNGSVEAVKPTTSKVNIFDSLPQTNLSISLGGSLGNQNGFPSSVVDEKSKMSSVLQQASKSRHLLPKPPRSVLATGLEMNAGTVPQIRVARPPAEGRGRNQLLPRYWPRITDQELQQISGDSNSTIVPLFEKVLSASDAGRIGRLVLPKACAEAYFPPISQPEGLPLRIQDVKGKEWVFQFRFWPNNNSRMYVLEGVTPCIQSMQLRAGDTVTFSRKDPEGKLVMGFRKATNTAVVQETLPPAIPNGTLSSESYFSGVFENLPIISGYSGLLQSLKGSADPHLNGLSKHSSLAGGDISGHKSDMHEDRIREDLLLTSMLTPERKRTRNIGSKSKRLLIDSQDALELKLTWEEAQDLLHPPPSIKPSIVTIEDHDFEEYDEPPVFGKRSIFAVRSTGGQEQWAQCDNCSKWRRVPVDALLPPKWTCADNNWDQSRSSCSLPDELTPRELEILLRLNTDFTKRRIIAFHRQTQEYESSPGLNALANAAILGDNVADSGTTSVATTTKHPRHRPGCSCIVCIQPPSGKGKHKPTCTCNVCLTVKRRFKTLMMRKKKRQSEREAEIALRNQQAWGPREEAEVESSSKHVSPHHDPSENEARSVNKLESKNQSNNNKLVSKLVEANKGRIDLNCDPCRDDDSQFGSSTRMSMMNLLHVASLPLETYLKENGLTSLVSDQQAISTSHAPPQTGNTSEPYDDQCSHSATEEHETRDEVNT

>GrVAL2b Gorai.011G127500.1 *Gossypium raimondii*

MASKSCMNGLCGATTSIEWRKGWALRSGDFANLCDKCGSAYEQSIFCDIFHSKDAGWRECSSCGKRLHCGCIVSRSLLELLDSGGGIICISCAKKSGLNPMIEDEKPNGFGIVKIDAGQLHSISADNQLISISNENLKLMQLSNNAESIGLRQLLQLHNDDSSRSLLQMKQEEVLPPAIDIGSTCLSNTNQASNGSVQAVKPAIFKANISETLPQTNLSISLGSSLGNQNVFPGSVVDEKGKMSSVLQQASKSLHLLPKPPKPVLAGLEVNAGMVPQIRVARPPVEGRGRNQLLPRYWPRITDQELQQISGDSNSTIVPLFEKVLSASDAGRIGRLVLPKACAEAYFPPISQPEGLPLRIQDVKGKEWVFQFRFWPNNNSRMYVLEGVTPCMQSMQLQAGDTVTFSRMDPEGKLVMGFRKATNNAVVQENLPSAIPNGTLSSESLFSGVFENLPIISGYSGLLQSPKGSTDPHLNALSKHLSSTGGDISWNKSGKHEDRIREGLLLPSMLTPERKRTRNIGSKSKRLLIDSQDALELKLTWEEAQDLLRPPPSIKPSVVTIEDHDFEEYDEPPVFGKRSIFAVRSTGGQEQWAQCDSCSKWRRLPVDVLLPPKWTCADNNWDQSRSSCSAPEELTPRELENLLRLNRDFKKRRIAAFTRPTQEHESSSGLDALANAAILGDNADNSGTTSVATTTKHPRHRPGCSCIVCIQPPSGKGKHKPTCTCVVCMTVKRRFKTLTMRKKKRQSEREAEIAQRNQQAWGPSEEAEVDSSAKHVSSSHLN

PFENEARSANELESKSQSSNKLVEANKGQIDLNCDPDREDDSQLGPNRTSMTSLVRVASLPLETYLKENGLTNLVYEQQGNSASNAPPQSMSGTVEGETQENSCFPSATEEPESKDEENGETGSDRVDDNNDKDP

>GrVAL2c Gorai.002G075900.1 *Gossypium raimondii*

MAAPTVFIGTCFHCQSQSDVFFSGWQLRDGSFALLCHPCGKVWYCFAGAFYPPFVNSAFLFPLLRIMFEEQAEEKWSRNPGVCCHSLSSHTIYCFLSYRRLSSAFVEGRFCETFHPEASGWRECDSCNKGIHCGCIMAAHSYAILDFGGVKCLECCLNEALALHRNSPTFSNPEEMQASDSHPKASDDTEETVTGVDPIASPDSVALNASPDKIGTPTPGAVPAAETEGESSVNSPAGTKKSRKKKGRKNRKDASKQHQIQARAKSSLIPLFEKKLTASDVDTRNGRLVLPKRCAEISGQQGIFLTVQDTKGNDWEVFYRYWSNTNGKMYVLEGLKDYMIMMEWEAGDTVTFYKREEDEKLFMGFKKYQAPESAQKASSLNLVLFIEGVGSSFLYMLSSVSELLLSRKMQVIGMNDKKTKAYILSE

>GrVAL3a Gorai.009G173100.1 *Gossypium raimondii*

MTSTPGAASSSKICFNSDCKDLKSEIPRKGWRLRTGEFAELCDRCAFAFEEGRFCDTFHLNASGWRTCESCGKRVHCGCIVSVHAFTLLDAGGIECVACARKNVVMGSSSSWSPSLIFHSSLSERFKEYSAKGWTQLAGSGPVPWRQAPSLFNSPVTQPELPSRVPYEVDLSTGIDRLNVCDRLSTPSLEKKKVEDFSERLMNGTLKPGTRDIHEKGNAGINCEEQRSPCLTKFQSSLKEEPNPQFGLAVPYTSPDEANGQNGVSGTHLRPNPQPPLAKQFHSNLQNGTDSSGETQIRNGRPRPDGRGKNNLFPRYWPRFTDQDLQQITGDSNSVITPLFEKMLSASDAGRIGRLVLPKKCAEAHFPPISQPEGLPLKVQDSKGKEWIFQFRFWPNNNSRMYVLEGVTPCIQNMQVQAGDVVTFSRLEPGGKLVMGFRKASTASASDQDNEAKNSNGLSMHGDAEMADPTSWSKVDKSGYIAKEALGAKVAVSRKRKNSMLGSKSKRLRIDNEDLIELKLTWEEAQGLLRPPPNHVASVVVIEGFEFEEYGDAPILGKPTIFATDNMGEKIQWAQCEDCFKWRRLPSNVLLPSKWTCSSNSWDPERSSCSAIQELTAEQLENLLPQCNPAASKKMKAAKQEMENVDALEGLDTLANLAILGEGESLPTSSQATTKHPRHRPGCSCIVCIQPPSGKGPKHKQTCTCNVCETVKRRFRTLMMRREKKQSQKEAETTSKKQQTSLPDKVPDDDPPPCTNAENSSPKPIKIASEGSEDDPNRVKSSISPFKGQIDLNIQPEREEELSPGSDSGSMMRLLQDATDKHLRQQSTLTSGGNSNSEVSQTQPGGGPEGEKISNSVNLGASHQDIDRDHPVFSIKTSAPTSATG

>GrVAL3b Gorai.011G022400.1 *Gossypium raimondii*

MTSTSVATSSSRICFNSVCNDLKSERPRKGWQLRTGELAELCDRCASIYDEGRFCDAFHLNASGWRTCESCGKRVHCGCIVSAYAFALLDAGGILCIACSRKNAVLGPNSSWPPSLLFNSSLPERFKDCSAKGWSQLTGSGPAPWRQAPGLFNSSTSQPELHSRVPYEVDLSTGIDRLNVSERLSIPSLEKKKNEDFPERLMNGPLKPSAHDIHENGNTGINCEEQHTSCLTKPQLPSLKEDSSNPPFGLAVPYTSIDEANGQMGVSGTHLRPNPQPSLAKQFHSNPHNGLDSSGETQMRNGRPRADGRGRNQLFPRYWPRFTDQDLQQISADSNSIITPLFEKMLSASDAGRIGRLVLPKKCAEAYFPPISQPEGLPLKVQDSKGKEWIFQFRFWPNNNSRMYVLEGVTPCIQNMQLQAGDVVTFSRLEPGGKLVMGFRKASAASASEQENEAKNSNGVSTHGDAELADPSSWSKVDKSGYIAKEALGTKVAVPRKRKNSVLGSKSKRLRIDNEDMIELKLTWEEAQGLLRPPPNHVANVVSIEGFEFEEYEDAPVLGKPTIFATDKSGEKIQWAQCEDCFKWRRLPSDVLLPSKWTCSSNSWDPERSSCSATQELTAEELENLLPHCNLAASKKMKAAKQESENVDALEGLDTLANLAILGEGDVLPASQATTKHPRHRPGCSCIVCIQPPSGKGPKHKQTCTCNVCQTVKRRFRTLMLRREKKQSQKEADATRKKQQPSLPDKVVGDEPLPCTTSAGNSSLNPEKVVSENLDDDTNRLKSSSSPFKGQIDLNIQPEREEELSPGSDSGGTMRLLQDATDKYHRQQSILSSSGNSNIEVTQTQPGSGTELGKINSSIDLRASHRDADMDHPAIFPIKTFAPTSATG

>FvVAL1 mrna28098.1-v1.0-hybrid *Fragaria vesca*

MGTKICMNVSCGTSNTHEWKNGWPLRSGGFAHLCYKCGAAYEKSVFCDTFHIGETGWRDCSSCHKPLHCGCVASRSLYECLDYGGVGCIGCANSSQPRVIPRNDVLNGFGGLALSNAGDRNSSSVEYRTVGDTVDEGKFLQLCGTVDEGKLLQLCKIMEANESTLLPQSPTGEESVFLESNLLPQPQRDDKIESLGQTKGQEVIHQIGEVSPGFFSPTQSSIGSLTFAAQPDNGRTMLEVNHMSIPSSQPSLSMSLCAPSATSNFIQPFSGGHMDIRDQSKTPSAFQQVKPRPILPRPLQPPLPPVSSETNRSVRIARPPTEGRGRNQLLPRYWPRITDQELQKLSGALNSTIVPLFEKVLSASDAGRIGRLVLPKACAEAYFPPISHSEGLPLRIQDVKGNEWTFQFRFWPNNNSRMYVLEGVTPCIQSMQLQAGDTVTFSRIDPGNKLVIGFRKASQSVNMQGPQTSVLQNGTPGETSFSSENLATGSGDSSLFHMNKGSKDPPLNSPSDHHLAEWDTYMQNRENSGHRTSEDLLQPASNSEKKRTRNIGPKSKRLLMHSEDVMELRLTWEEAQDLLRPPPSVKPSIVTIEDFEFEEYDEPPVFGKRSIFTAGPSKRREQWAQCDDCSKWRMLPVDVLLPPKWTCSENSWDSSRSSCTAPEEMSSKQLDNLLSSSVSLKGLKKRRKIIEKKNAEEQEPSGLDALASAAILGDNVRESGEQSVGATTKHPRHRPGCTCIVCIQPPSGKGKHKPSCKCNVCLTVKRRFTTMMQRKYEKRQLEREAENSQRNNGNNKDESEVNGTTSGDAMLHRNNSANNSQRNNNNHKDESEINGTTSGDAALHRNHSSENGASSSQSRTQADAAESSSAGQIDLNCEPSGLFRNPTLQDLFKLAKAASAARPSEKYTNENSLRTMMDEEQAGLASCSLKQANGDNERQLPNEAHLSSVSWDCPSIGDKVYREPDLE

>FvVAL2 mrna13877.1-v1.0-hybrid *Fragaria vesca*

MDPSACMNAYCGSSSSIEWKKGWALRSGRFANLCHKCGGLLEVLSPWSVDSLLFQRCALFLTAAWYLWPRLHCGCIASRSLLDFLDGGGVKCTHCTKNSEPHPIASDEKPDGPGTSKISELKSTPSDNHLDRSNVDNVKLIQLENDKECNGLRNLLQSQNNETVGLLQKMKQDDVPAPVVEIGGTGLSIFNQTSNVSSEGCKPVIYRGNLGINDMYESLPHTNLSMSLGAPSGYANPFPGIVVDEHTRTSSLFLQGARSRHLLPKPPKLALATGLEENSTMASQSRVARPPAEGRGRNQLLPRYWPRITDQELQQISGDPNSTIVPLFEKMLSASDAGRIGRLVLPKACAEAYFPPISQPEGLPLRIQDVKGKEWVFQFRFWPNNNSRMYVLEGVTPCIQSMQLQAGDTVTFSRMDPEGKLIMGFRKASNSASMQDTHLSAIHNGAHSSQTFFSGVIENLPVISGYSGLLQSTKGMDPHLSALSKQLTTAHGDLSWHKSENPESRAREGLLLQSLVVPERKRTRNIGSKSKRLLIDSQDVLEVKLTWEEAQDLLRPPPAVNPSTVMIEDLEFEEYEEPPVFGKRSIFIVRSTGEHEQWAQCDGCSKWRRLPVDVLLPSKWMCTDNVWDQNRCSCSAPDELTPKELESFLRLSKEFKKRRMATNHNPTQEHESSGLDALANAAILGDNVADPGTASVATTTKHPRHRPGCSCIVCIQPPSGKGKHKPSCTCNVCMTVKRRFKTLMINKKKRQSEREAEIAGRNQLAWGPRDDAEVDSTSRHLSSHLDPSDNEAKSPNELESKSQLKMAESGKGKLDLNCHPGREVDLPAEPSQLSMMSLLQVATLPLDSYLKQTGLTSLVTEQQTSSSPPVPPQATEENEEQLNGDQCLVSIVQDQESGGEERQDQSQEDPL

>FvVAL3 mrna30690.1-v1.0-hybrid *Fragaria vesca*

MYRTEMLMVSDHRSTTKISYRRKGRSRKSAKPKRHLPQAQLVQNVSAVIPDYQQRQFQYFQNPETKISYRRKGRSRKSAKPKRHLPQPQPQIQLVQNASAVIPDYQQRQFQYFQNPETRPDPLFSTRGLYGWPIYHGAMSGVVGFDTVHGNGGSVGFGYGYNGVVGDGGAQNRGAGVYHEISNDRGFSGSFVGESVEVRPVPDHNNNSNSSSVVSSGGTFPMEHVLVASSTNSSSVGRSSEASDQHLGAAPACIEGADMMMMSSGYSSPQSEARTSMSSSAASSSSSSSKCCYNSECGELKPEHPRKAWRLRTGDCAELCDRCAVIYEEGRFCETFHVKASGWRCCESCGKRVHCGCIVSAQAFTLLDPGGIECVTCARKYIVLTSNPAWAPTLLFHSPFSERPKDMSGKNWTQLAGSGPVPWRQAPSFFNSSISSPDLHTRVPYDIDGSMGINKLNAGERISVHPLEKKKAEDFSERLMKVNLAIGTPVTFENGNSGNKRMEQSRSCLNVSQQPISLKEEQSTPQFVLSVPYVSASDTNGQKNGQVVVTGTNLQPALPMANQFHGIRGASYNGVELSGDTQIRYAKSGLDARGRLSRYWPRFTDKELQQISGDSNSVITPLFEKMLSASDAGKIGRLVLPKKCAEAYFPPISHPEGLPLTVHDSLGKEWVFQFRFWPNNNSRMYVLEGVTPCIQSMQLQAGDIVTFSRLEPGGKLVMGCRKALTAPSDQENDTNKMGNGASANADVGITDTKSGEVFSNAELAVRSTVAKGDKSGYVSKDVVGAKSLISRKRKNSILGSKSKRLRIEKEDLIELKITWEEAQGLLRPPPKHVPTVVVIEGFEFEEYEDGPILGKPTLFTTGSKGEKIQWAQCEVCCKWRKLPVNALLPSKWTCSDNSWDPDRSLCSAAQELTIEELEDLLPRNTVAPKNVKASKQDPDSIEALEGLDTLADLAILGEGESLPSAAQATTKHPRHRPGCSCIVCIQPPSGKGPKHKQTCICNVCSTVKRRFRTLMMRREKNKSEKEAETTRKKQQQQQFQDDDTLLSGNTGSSSPNNRVEVLDLSDDDFNRTKSPTPPYKGQIDLNIQPEREEDLSPGSDSGGMIKLLQDATERYLRQQRQSLSISVGGERSLGNKSQLAGIEAGGEPLGNIAILGNNNHDGGNDHPATLSIKASASMTAT

>PtVAL1a Potri.013G157500.1 *Populus trichocarpa*

MGTKICMNASCQTATTHEWKRGWPLRSGGHALLCYNCGSAYEDSLFCDTFHSEEPGWRECNICSKHLHCGCIASKFLLELLDYGGVGCASCARSSRLHLMQSDEIPNGFSFLARNNAGDSESIPAENTVAGNSNDEGGLAQLCRLLEANEPSLLHPSKRANTNGGLGQFRQEEIMHAIGDIGTGFSNVTQPSIGSPKFSKPNNTSSLLDLRDMHSSLSQPSLSMALGAPSGTTNFVTFPDGAVEGKEQRKTPSSFQQGQRSRPILPKPSKPGLSVSSENNIGAASELRIARPPAEGRGKNQLLPRYWPRITDQELQQLSGDLNSNIVPLFEKILSASDAGRIGRLVLPKACAEAYFPAISQSEGIPLRIQDIKGREWTFQFRFWPNNNSRMYVLEGVTPCIHSMQLKAGDTITFSRIDPGGKLVMGFRKSTNNDEDTQASGLLDGTASGETSFSGTVETLLTDPYQNALSKRLKLADGDIGWNNSENHRGRINGDLLQQTTAPTEKKRTQNIGPKSKRLFMHSEDAMELRLTWEEAQDLLRPPPSVKPTIVTIEDHEFEEYDEPPVFGKRTIFTSRSSGGQEQWAQCDDCSKWRNLPVDALLPPKWTCSENAWDSSRCTCSAPEEMTSKDLDNVLGVSKDFKKRRNLRSQKRFQERESSGLDALATVAVLGDNLDDSGDPSVGATTKHPRHRLGCTCIVCIQPPSGKGKHKPTCTCNVCMTVKRRFKTLMLRKKKRQSEREAEISQKDNMDHKEESEMNGTMSHEVLHINNPDTEGNQSQRQIEKPETSAGQIDLNCHPNSEDTSLEIPGLSTMNLVDTANSPLDNYIKQNGLSSFVWDQQGGPAQKSGESLRRLSDEAFLASIGWTHESIRD

>PtVAL1b Potri.019G130300.1 *Populus trichocarpa*

MGTKICMNASCQTTTTHEWKRGWPLRSGGHALLCFNCGSAYEDSLFCDTFHSEEPGWRECNICTKRLHCGCIASKFLLELLDYGGVGCSSCARSSRLHSMQSDEIPNGYGSLTRNNAGDLESIPVESTVAANDYDEGGLAQLCRLIEASEPSLLHPSERANANGCLGQFRQEEIRHAIGDIGTGFSNMTLPSVGSSKFTNPDNMSSLLDMRDMHCSLSEPSLSMALGAPSGTTNFAPFPGGAVEGREQGKTPSSFQQGQRSRPILPKPSKPGLLSSSENNKGSASELRIARPPAEGRGKNQLLPRYWPRITDQELQQLSGDLNSNIVPLFEKILSASDAGRIGRLVLPKACAEAYFPPISQSEGIPLKIQDIKGREWTFQFRFWPNNNSRMYVLEGVTPCIQSMQLKAGDTITFSRIDPGGKLVMGFRKSTNNNEDIQDAQASGLPDGTASGETSLSADGYIGWNNSENHGGGINGDLLQQTTAPTEKKRTRNIGPKSKRLLMHSEDAMELRLTWEEAQDLLRPPPSVKPTIVTIEDHEFEEYDEPPVFGKRTIFTSRSPGRQEQWAQCDDCSKWRKLPVDALLPPKWTCSENAWDSSRCTCSVPEEMTPKDLDNLLRVSKDFKKRRILESQKRFQNCEPSGLDALASAAVLGDNLDDSGEPSVGATTKHPRHRPGCTCIVCIQPPSGKGKHKPTCTCNVCMTVKRRFKTLMLRKKKRQSEREAETSQKDNMDRKESEANGTMSHEVLPINNPETEVGQSKTQTEKPETSAGQIDLNCHPNREDMPLDMPGLSTMNLVDLANTPLDNYIKQNGLSSLVWDQEGGQAQHSGESLRRLSDEAFLASIGWAHESRRD

>PtVAL2a Potri.006G108300.1 *Populus trichocarpa*

MASSSSSIKSCMNATCGVSTSNSGGWRKGWALRSGDFAILCDNCGSAYEQSIFCEVFHSKDSGWRECTSCSKRLHCGCIASRSLLELLDGGGVNCTSCSRTSGVGPMNGDEKPNGFGKPKVDTVGELHSASADSQLAAETKLMQLGNCIDGIGTRNLLQLQSDETNGSYRKMKQEDILPPVGEIASTIFSNFNQASNASCQTAKPEIHRTVTAAKDLYESLAQTNLSMSLGSSLGNPNLFPGGVVDERVPSKASSPLQQGPRSRHLLPKPPKSALSMDANAGMVSQIRVARPPAEGRGRNQLLPRYWPRITDQELQQISGDPNSTIVPLFEKVLSASDAGRIGRLVLPKACAEAYFPPISQPEGLPLRIQDVKGKEWVFQFRFWPNNNSRMYVLEGVTPCIQSMKLQAGDTVTFSRMDPEGKLVMGFRKASNSIAMQDTQPSAIPNGVPSSESYFSGVFENLPIISGYSGLLQSLKGSTDTHLSALSKHLHSASGDISWNKSEKQEDRTRDGLLLPSLMVPERKRTRNIGSKSKRLLIDSLDAFELKLTWEEAQDLLRPAPSVKPSIVTIEDHDFEEYEEPPVFGKRSIFIVRSIGGQEQWAQCDSCSKWRRLPVDVLLPPKWTCVDNAWDQSRCSCSAPDELAPRELENLLRLNKDFKKRKITSSHQPAQELESSGLDALANAAILGDVGEQSTTAVVATTTKHPRHRPGCSCIVCIQPPSGKGKHKPTCTCNVCMTVKRRFKTLMMRKKKRQSEREAEIAQKTQHLVGPKDEAEIESSSKLASIPRDPSDNEARSGNELESKGQSNNLSNKLADSGKGHLDLNCHPDREEDSQAGLSRMSMTSFLQVATLPLDTYLKQNGLASLSEQQASSASHVPPQTGENEGKINDDCQPATAAPEQESGGEENDEPGPDQSQNDPV

>PtVAL2b Potri.016G136500.1 *Populus trichocarpa*

MASSSIKSCMNATCGVSTSSSGGWRKGWALRSGDFAILCDNCGSAYEQSVFCEVFHSKDSGWRECTSCGKRLHCGCIASKSLLELLDGGGVNCTSCSKSAGVSSVNGDEKTNGFGMSKVDDAGELQSASADNQLTTETKLMQLGNCIDRIATRNLLQLQSSETDGSYRKMKQEDIIPPVGEIASTSFLNFNHISNASSQTAKPEIHKTTAAKDLYESLAQTNLSISLGSSLGNPNPFPGGVVDERVLAKASSPLQQGPRSRHLLPKPPKPALVLDANAGMVSQIRVARPPAEGRGRNQLLPRYWPRITDQELQQISGDPNSTIVPLFEKVLSASDAGRIGRLVLPKACAEAYFPPISQPEGLPLRIQDVKGKEWVFQFRFWPNNNSRMYVLEGVTPCIQSMKLQAGDTVTFSRMDPEGKLVMGFRKASNSIAMQDTQPSAIPNGVPSSESYFSGVFENLPIISGYSGLLHSLKGSTDTHLSALSKHLHSASGDISWHKSEKQEARTRDGLLLPSLLAPERKRLRNIGSKSKRLLIDSLDALELKVTWEEAQDLLRPEPSIKPSIVTIEDHDFEEYEEPPVFGKTSIFVVRSIGGQEQWAQCDSCSKWRRLPIDVLLPPKWTCVDNAWDQSRCSCSAPDELAPRELENLLRLTKDFKKRRITSSHRPAQEHESSGLDALANAAILGDAGEQSTTAVAATTKHPRHRPGCSCIVCIQPPSGKGKHKPTCTCNVCMTVKRRFKTLMMRKKKRQSEREAEIAQRIQHMSGPKDEADVESSSKLASTPMDPSDNEARSGNELESKSQTNNLSNKLADSGKGHLDLNCHPGREEDSQAGLARMSMTSLLQVASLPLETYLKQNGLASLSEQQASSASHVPPQAGENGGRIDEDCQPASVAQEQESGGEEDDEPGPDQSQTDLL

>PtVAL3a Potri.004G035300.1 *Populus trichocarpa*

MSSSPPHLPTSTTATTANNNNTSCSSSSTALSRKCFNSDCTDFKSRKGWRLRSGDFAELCDRCASAYEEGSFCETFHLRASGWRCCESCGKGVHCGCIVSIQTFTLLDAGGIACMACERKSFVLESNPQILLTATSADVTSNPAWSPLFYHAPFPERLKDLSVKSWSQLAGSGPVPWRQAPSLFNPSAAQSELQTRMPYEVDRLNTGERFSAPSLEKRKVEDFSEKFINGNLRIRLQDIVENGNAGIIGEEQPQPSSSLMEDASGQQFGITIPYKSISESNSQIEGSVNALQPAPPPPFTKHFHGSLHNGVDSSVDGHIRNGKPRTDARGRSQLLPRYWPRFTDGELQQISGNSNSVIKPLFEKMLSASDAGRIGRLVLPKKCAEAYFPPISQPEGLPLRVQDSKGKEWIFQFRFWPNNNSRMYVLEGVTPCIQNMQLQAGDIVTFSRLEPEGKLVMGFRKATSAPPSDQDNETSQTGNGVSTKGDAELDPSPWSKVDKSGYIAKEVLEGKSSIRKRKSSTLGSKSKRLRIENEDMIELKLTWEEAQGLLRPPPDHVPSIVAIEGFEFEEYEDAPVLGKPTIFATDNVGQKIQWVQCEDCLKWRKLPANALLPSKWACSSNTWDPERSSCSVAQELTAEQLEDLLPSCNLVVTSKRSKDAKKDIDRVEALEGLDTLANLAILGEGEAFPASSQATTKHPRHRPGCSCIVCIQPPSGKGPKHKQTCTCNVCQTVKRRFKTLMMKREKKQSEKEAETKQSEKEAETTRKRQQEPSAEKLLDDEPSPSSNTGSESGSPNKKKTVSEGSDDDPSRMKSSTSPFKGQIDLNIQPEREDELSPGSDSGGMMRMLQDATETYLRMQRFLSSDGDNNPSGNHMLSSGGTREKVSDVIMLGSSHQDADKDHPSAFSMNASASTPATG

>PtVAL3b Potri.011G043600.1 *Populus trichocarpa*

MSSSSSSLAATSTTATATNNNNNNTSCCSSSSTVLSRKCFNSDCTDFKSRKGWRLRSGDFADLCDRCASAHEEGRFCETFHSNASGWRSCESCGKRVHCGCIVSLQAFTLLDAGGIACMACARKSFVLTPNPAWPLFYHSPFPERLKDLSVKSWSQLAGSGPVPWRQAPSLFNSSATQSELQPRMPYEVDRLNAGERFSALSLEKRKLEDFPERFINGSPRNHLQDIVENGNAGIIDDEQPQMLSSLREDISAQQFGITIPYASPSKLKGQIEGSVNPLLPAPLPLFAKRVHGTLHKGVDSSVDGQIRNGRPRIDARGKSQLLPRYWPRFTDEELQQISGNPNSVIKPLFEKMLSASDAGRIGRLVLPKKCAEAYFPPISQPEGLPLRVQDSKGKEWIFQFRFWPNNNSRMYVLEGVTPCIQNMQLQAGDIVTFSRLEPEGKLVMGFRKASSASLSDQDNETSQIGDGVSANGDAELGPSPWSKVDKSGYIAKEVLEAKSSIKKRKSSTLGSKSKRLRIENEDMIELKLTWEEAQGLLRPAPDHVPSIVVIEGFEFEEYEDAPVLGKPTIFAMDNVGHKIQWVQCEDCLKWRKLPLNVLLPSKWTCSGNTWDSERSSCSAAQDLTSEQLENLLPSRNLVTSKRLKAAKKDIDNVEALGGLDTLANLAILGEGEALLASSQATTKHPRHRPGCSCIVCIQPPSGKGPKHKQTCTCNVCQTVKRRFKTLMMKREKKQSEKEAETTRKQQESSAEKLLDDDPSPSSNAGSASGSPNKKKAVSEVSDDDPNRMKSSTSPFKGQIDLNIQPEREDELSPGSDSGGMMRMLQDASERYLRMQRFLSSDGDNIPASNHSVSSGGADEKAGSGIMLGGSHQDVSEDHPSAFSLIASESASASTPAT

>BdVAL2 Bradi1g17680.1 *Brachypodium distachyon*

MAMAAVPAKRCMNTACGAAAPGVGGAAGEWRKGWPLRSGGFAVLCDKCGLAYEQLVFCDIFHPQESGWRDCSFCGKRLHCGCAASKIFYGLLDSGGVQCMNCMQNSRPQPVSCQVAPKLFLSPNNQRLFGKSDELLPGRPLESPPLMLDSRNDDIAIVAKSNHPFMVKNIEPGQSSNIFRQKEIENGARQIKWEQPTLSIGDMGRMPFLIKAQSTLESPQSQCTPRDDIRDPTADSTTSESLSEACLSMSLAITNNGNKTEATSTMERPSLSPTTLFSDGRELATSLSPFQHAQRARHYLTRPPRVAEGAAFDPMKDGFPHLRVARPPAEGRGRNQLLPRYWPRITDQELQQISGDSNSTIVPLFEKVLSASDAGRIGRLVLPKACAEAYFPPISQPEGRPLTIQDSKGKEWHFQFRFWPNNNSRMYVLEGVTPCIQSLQLQAGDTVTFSRIEPGGKLVMGFRKATNTVSLPDSQISAIANGSLLGDTFFSSPSENLSIVSGYSGFLQSMKGATDHHPSSLFDHHANSADGDVSWLKTDRFGGRSDEGSLQFLQRRSRNIGSKSRRLLMDAEDALELKLSWEEAQELLRPAPSAKPTVVMIEDYEFEEYDEPPVFAKRSIFTRRSTGEQDQWIQCDDCSKWRRLPLNVIIASKWTCPDNTWDPKSCSCSTPQELATKDLHSILQQYEDIRRRKSSYFLKQNIPEMDASSFDALAAATVFGEVGNQGASSVATTTKHPRHRPGCTCIVCIQPPSGKGPKHNPACTCNVCMTVRRRFKTLMMRKKQRQSEREEAEASKKITWMSRDEPEGSSLSRSPQTLDPARDGDVTMLDKVDMNKGHIDLNFHPASRDEVSMVGLLEVASRPLESYMKQNGLVSLAGEQASSSTQPPMVLPQAAPVGSEEQTPDEARVMSVVEEREREPTDSMAIDEAAGENYGIAATDNAATA

>BdVAL3 Bradi1g24720.1 *Brachypodium distachyon*

MSSPAPPRPPPPSAPTPATAPTATPMSVQPPPPQPKPPPPQQPSGSSAPPPPQFHPPASHQQPQPHPAASHQQPQPHTPVSHQQPQQPHPAGSHQQQQQQPHPPSSHQQRPRICFNTHCKDPKSEGPRRRGWRLRSGDFAELCDRCYGSFEQGSFCETFHSEVAGWRNCEACGKRLHCGCIVSIHAYALLDAGGVDCILCTRKSYAAAAMAPNQMCPTPTMHMPQNVADKKDSFVKSWRPPGGPYPSQWRQNSLWSVSGIQSDLQQRLAYEFDRPSGSEKLPPGRTFIHAQEKKFDDMHDRPTTPAGMNQIIRDRHADGHGQQTSMDPAHSYALYQREGPNPNSLHDHSHHGGESDSTSARKGIISEGCSSNIVSSSFKLDSRHPLESLLKENISLLPVGVGCTITNCPPVNGRNDIVRIIPHQPTSQTPSSAASSAQKQFYSHTVIDPEYQSHFRNGKPRMDAKARSQLLPRYWPRITDKELQHLSADSKSVITPLFEKMLSASDAGRIGRLVLPKKCAEAYFPPISQPEGLPLKVQDGSGKEWVFQFRFWPNNNSRMYVLEGVTPCIQSMHLQAGDIVTFSRIDPEGKLIMGFRKSTTQEQILRQEQPTKPANAAVTAPPEVNVNVARPHEVNTENKNISPVDQAAVGKVENGGVAQKEGPGTARSSPGSLKRKATSVGPKIKRFRMDNEESMELKITWEEAQELLRPPLKAPSVVIVDGHEFEEYEEPPILGRRTYFVTDQSGENHQWAQCEDCSKWRKLPVGALLPSKWTCSDNKWDPERTSCESAQEATTEELAELFPIKAGAAKKPKARIEPDSIDVSDGLDTLANLAILGEGESLPSQPTTKHPRHRPGCSCIVCIQPPSGKGPKHKQTCTCNVCMTVRRRFKTLMLRREKRLSEKDTEEPRRKEVPQTGSDPPLGSTSPTSSPQKADANPDDAEDMVVDHRMSSSPVKNQIDLNIQPEREDEQSPKSNAVGAARLPRDNPT

>OsVAL2 LOC_Os07g48200.1 *Oryza sativa*

MAGMAAAAAALVAAKRCMNAACGAPAPSPAGGEWRKGWPLRSGGFAVLCDKCGLAYEQLVFCDIFHQKESGWRDCSFCGKRLHCGCIASKNSFDLLDSGGVQCVTCIKNSAVQSVPSPVVPKLFSSQNNQRLFGKSDDLLSGRPLETSSLMVDARNDDLTIIAKNNLPFMVKNVEAGQSSNILRQKELENGARQIKWELPTLSIGDMGRIPFLTRSQSALESRRDENKDPTTESTTSESLSEACLNMSLGIASNGNKLEATSTVERPMLSPTTGFPEGRELTTALSPFQHAQRARHFLTRPPRVGEGAVFDPTKDMLPHLRVARPPAEGRGRNQLLPRYWPRITDQELQQISGDSNSTIVPLFEKVLSASDAGRIGRLVLPKACAEAYFPPISQPEGRPLTIQDAKGKEWHFQFRFWPNNNSRMYVLEGVTPCIQSLQLQAGDTVTFSRIEPGGKLVMGFRKATNTVSLPDSQISAIANGSILGDTLFSSTNENLAIVSGYSGFLQSIKGAADLHTSSIYDHHVNSADGDVSWLKTDKFGSRPDEGSLQFLKRGRNIGSKSRRLSMDAEEAWELKLYWDEVQELLRPAPTAKPTVVMIEDYEIEEYDEPPVFAKRSIFTIRSTGEQDQWIQCDDCSKWRRLPLNVIVASKWTCADNTIDSKSCSCSAPEELTPKELHIVLQQYEDMRRRRNSFGFKQNIPEMDAVSLDAFATAAVYGDVGNQGSPSVATTTKHPRHRPGCTCIVCIQPPSGKGPKHNPACTCNVCMTVRRRFKTLMMRKKQRQSEREEAEASKKIAWMNRDEPEGSSLSRSPQTVDTTRDGDVTMFDKVDINKGHIDLNFHPTAVRDEERHGGQPRVSMVSLLEVANRPLENYMKQNGLTSLAGEQGSSSTCTGAATVPQPALVESEERTSNNDGGRVATAEQPESMAVDEAGDNQPDKAAGDSAAALA

>OsVAL3 LOC_Os07g37610.1 *Oryza sativa*

MSSPAQPPPTRPPVAAPPPSLAAAAPISVQPPPLQPKPPPHPQQPPQAVVSVGVGPPPPTPQHQQQQQGPPGHAPPQQRPRICFNAHCKDPKSDGPRRRGWRLRNGDFAELCDRCYHSFEHGGFCETFHLEVAGWRNCESCGKRLHCGCIVSVHAFVHLDAGGVECVMCARKSHAAMAPSQIWSSSMHMAQNVADRKDNFVKSWRPPAGQFSSQWRQNNMWSMSTMQSDLQQRLAFEFDRPSGSEKLLPGRTFIHAHEKKFDDMHDRSTTPAGMNQIMRERYANGHTQHTTLDPTYAYTLYHREGTNPNLHDHSHHAGENDHLTARKGVTSDPCSSVSTTFKLDSHHPSILKDDPSAVPAGLSSNFSSANGPKDHIRIGPTQQQQQMASSSLQKQFYSHSVIDNDFQAQLRNGRPRMDAKARSQLLPRYWPRITDQELQHLSGDSNSVITPLFEKMLSASDAGRIGRLVLPKKCAEAYFPAISQAEGLPLKVQDATGKEWVFQFRFWPNNNSRMYVLEGVTPCIQSMQLQAGDTVTFSRIDPEGKLVMGFRKATNLSAEQDQPTKPANGVLPPPEANNKVVVPDSSPNAAVPRPIKVNTESKSSSPVEQATACKIDKGALPQKEGPGTSSSSPLPVKRKATSVGPKIKRFHMDSEESMELKITWEEAQELLRPPPKAPSIVVVDGHEFEEYEEPPILGRRTYFVTDQSGENHQWAQCEDCSKWRKLPVDALLPSKWTCSDNKWDSERSSCDSAQEINMEELGEMIPIKPGAAKKTKGKVDTDNIDVSDGLDTLANLAILGEGESLPSQPTTRHPRHRPGCSCIVCIQPPSGKGPKHKQTCTCNVCMTVRRRFRTLMMRREKRQQSEKDSGVPRKREPGQSSEPVPQSGSGAHPTSTSSPHQRADTNGEGPEDMSIDNKRTSSPVKNQIDLNSQPEREDEQSPKSDATRLLRDNPT

>ZmVAL2a GRMZM2G172621_T01 *Zea mays*

MAMAAAAAAATKRCMNPACGAPASVLGAGGDWRKGWPLRSGCFALLCDKCGLAYEQFVFCDIFHQKESGWRDCSFCGKRLHCGCVASKNSYDLLDSGGVHCVTCMKNSLAQSASGQVVPKLFPSPINLRFFGKSDELLSSRNFEQPPSLTLDSRNDDIAFVNKSNHLFMVKGIEAGQSSNILRQKEIENGSRQIKWEQPTLSFGDMGRPFLTRSQSALESPQCTRRDDNKDPTTDSTSESFSEACLSMSLGIANNVNRMEATSTAERPVLLSTTAIAEGRELATTLSPFQHAQRARHFLTRPPRIGEGAAFDPTRDMFPHLRVARPPADGRGRNQLLPRYWPRITDQELQQISGDSNSTIVPLFEKVLSASDAGRIGRLVLPKACAEAYFPPISQPEGRPLTIQDARGKEWHFQFRFWPNNNSRMYVLEGVTPCIQSLQLQAGDTVTFSRIDPGGKLVMGFRKATNTVSLPDSQISAIANGSLLSETLFSTANENIGVVSGYPGFLHSIKGAADLHPNSLYDHHMNLVDGDVSWNKADKFGSRPDEGSLQFLQKRSHNIGSKSRRFLIDAEDAMELKLTWEEAQELLRPAPTAKPTVVMIEDYEFEEYDEPPVFAKRSIFTIRATGEQDQWIQCDDCSKWRRLPLNVIVAPKWTCTDNSWDSKCCSCSAPEELTPRELQSVLQQYEEMRRRKGSYGLKLNVAEMDASSLDAFATAAVFGEVGNQGSASVATTTKHPRHRPGCTCIVCIQPPSGKGPKHSPACTCNVCMTVRRRFKTLMMRKKQRQSEREEAEAGKKIAWVNRDEPEGSSLSRSPQTLDSTRDNSDVTATMLDKVSDVNKQGHIDVDLNLHPAAPAVQAAQQQPRPVSMMGLLEVAGRPLDNYMKQNGLTSLVGEQGGGSSSTATVPPPALVESEERTSNEGLVPSASAEREPNAMAVDEAGENQQDKAADDDDDAVAAAAT

>ZmVAL2b GRMZM2G158162_T01 *Zea mays*

MAMAGAGAAAAAAKRCMNPACGALPASSVVVGAGGDWRKGWPLRSGGFALLCDKCGLAYEQFVFCDIFHQKESGWRDCSFCGKRLHCGCVASKNSYDLLDSGGIQCVTCMKNSAAQSASGQVVPKLFPCPNNLLFFGKSDELLSSRKFEQPPSLMLDSRNDDIAIVNKSSHLFMVSGIEAGQSSNILRQKEIENGPRQIKWEQPTLNIGDMGRPLLTRSQSALESPQCTRRDDNKDPTTDSTTSESFPEACLSMNLGIANNGNRMEATSTAERPMLSPTTAIAEGRELTTALSPFQHAQRARHFLTRPPRVGEGAAFDPTRDMFPHLRVARPPAEGRGRNQLLPRYWPRITDQELQQISGDSNSTIVPLFEKVLSASDAGRIGRLVLPKACAEAYFPPISQPEGRPLTIQDARGKEWHFQFRFWPNNNSRMYVLEGVTPCIQSLQLQAGDTVTFSRIDPGGKLVMGFRKATNTVSLPDSQISAIANGSLLSETLFSTANESIGVVSGYPGFLHSIKGAADFHPSSLYDHHINSADGDVSWNKADKFGSRPDEGSLQFLQKRSRNIGSKSRRFLIDAEDAMELKLTWEEAQELLRPAPTAKPTVVMIEDYEFEEYDEPPVFAKRSIFTIRATGEQDQWIQCDECSKWRRLPLNVIVASKWTCTDNSWDPKCCSCSAPEELTPKELQSVMQQYEEMRRRKGSYGLKLNVAEMDASSFDALATGAVFGEVGNQGTASVATTTRHPRHRPGCTCIVCIQPPSGKGPKHNPACTCNVCMTVRRRFKTLMMRKKQRQSEREEAEASKKIAWVNRDEPEGSSLSRSPQTLDTTRDSSDVTMFDKVAADVNKGHIDLNFHPAAPAVRGAGDQGQNGAQQPRAVSMMGLLEVASRPLDNYMKQNGLTSLAGEQGGGSSSTATVPPAPVESEDRTSDEGRVASAEREADAMAIDEAGENQQDKPADSAAAAAT

>ZmVAL3 GRMZM2G008356_T01 *Zea mays*

MSSPGPQQAPPAQPPPPQAAAPPTAAPATVSATSISVQLPPIQPKPPPQPGPTSGPQPPTPAQLLNLGPQPPLYRGPICWNSYCKDPDPNSFGRRGWKVRSGPPYSVYADLCGRCYSQFEQGIYCETFHSEEGGWRNCESCGRRVHCGCIVSIHKYQLRDAGGVDCAKCARSTRAAMAPPSPVWTNPIHSSQNVSDRKDITVKSWRPPAGQISSQWRQTNLWSVSSIQSDLQQRLAFEFDRPSGSEKLLPGRTFIHSQERKFDDMHDRPATPAGMNHIMRERDPNGHGQTTNMDPAYSYTLYHRDGSHPNNLHDSNHHCGENDSLSSRKVAMPEASTSVDAGFKLDSHHTSNLKDDPPSLSVGLASNFAPQNGPKDHIRIAPTQQQSQMTSSSLQKQFYSHAVTGYNEFQAQMRNGRPRMDSKARSQLLPRYWPRITDQELQHLSSDSNSVITPLFEKMLSASDAGRIGRLVLPKKCAETYFPPISQPEGLPLKVQDASGKEWIFQFRFWPNNNSRMYVLEGVTPCIQSMQLQAGDTVTFSRIDPEGKLIMGFRKATNNSFEQEQATKPANGALATSEANGKVSAPDSSPNAAVSRQNKLNTETKSSSPVEQATALKMDKDGLTQKEGPGTGSSSPGSVKRKTTILGQKNKRLRMDNEESMELKITWEEAQELLRPPPKAPSIVIVDGHEFEEYEEPPVLGRKTYFVADKSGSNHQWAQCEDCSKWRKLPIDALLPSKWTCSDNKWDPERCSCESAQEISIEELAEFIPIKPAKKPKLKIESDAIDASDGLDTLANLAILGEGEALPSQPTTKHPRHRPGCSCIVCIQPPSGKGPKHKQTCTCNVCMTVRRRFRTLMLRREKKATKDLSRKKETGQSSEKATQQAVSGPPGANATATRSAQKADGNTDGPEDMAVDHKVTTSSPVKNHIDLNIQPERDDEQSPKSGAAGLLSRDNAT

>SmVAL1 88108 *Selaginella moellendorffii*

MEPTRHKACMNVRCEAIESAAWKRGWRLRSGDRADLCTDCGSAFELSRFCEKFHAYDPGWRSCIACGKGLHCGCIASIHLFVLLDTGGVSCRGCVDLRESRNVVRSFFFLFSDFPFDLGKEQEENLILKPKSEVDFEQKMAATGSEDEKVRGVAEELSAKREVTYMEEMEKLAVTRESPGTQTEVASLNTSLGLGMGIKVDDTAAGNLFQTESPASGGEAKENPSKYSPIPYRPRHRQVGKQPVPPRVPLSGAAPEPTNDPRVARPPADSKGRNQLLPRYWPRITDQEIQQFTSGDSKITPLFEKVLSASDAGRIGRLVLPKACAEAYFPTISQAEGLPLRINDISGREWQFQFRFWPNNNSRMYVLEGVTPCIQAMHLQAGDTVTFSRLEPEGKLIMGYRKAQDSGDSEYPGAKQTDYKSKAKETQSTLSADKRRGRPLGAKSKRLRLDSEDSLELKTSWEEAQELLRPPPGVTPSFVTIDGHQFEEYEEPPVLTKKTVTRTKPSGEQDRWVQCDDCAKFRRVPLDIFIHTRWTCTDNVWDLKRANCSAAKELSNEDMDQLMDSMSGKAQQRISPSGLDALATAAAFGDEKAASPPPPAATTKHPRHRPGCTCIVCIQPPSGKGPKHKPTCVCNVCLTVKRRFKTLMMRRKKRQSEREAETARKKKAWDKDDGAESAKSHERRDDPAVAVNLDAHDWHAREKHCPSVADQASRSQCVTACNVPGAEGRREERRKDIWMLLPEVALKKKCLMLVTGDKTYRVVTHSLEHIWTTSALANQRHPLLSQSRKRSSMLIGLCKHSCEYEGAGETSFVLYYLQGCQQSP

>PpVAL1a Pp3c3_23050V3.1 *Physcomitrella patens*

MSINSPSSTNPNLLPISGKFSAANTTFLFDKVVSATDCRSTGHFVLPKRKVEEHFPPLSKPGGVWMTLIDDAGKEWSFEFCFWYSKESRIYYLKRFYPYVQATNLRGGDTVYFSRLEPEGTLLMGVRKQKLASPKHGRAIKPKNELEAKVLSSRDLKVSSEEGKSLLDDGALLEDHPATPSTDDASVDYDLCKSTDKKKMMRLTSPPPISFDTQSVDHIATGEAVATRKRRSITPESSEQINMKPGSQSSRGKRLGLTMEDYYEWEDIQDLFRAPPGAIATYTCVEGHEIEEYKEPPVLIKKTFYSAEIQEDDQWVQCDDCGCWRRLPADAFVHARWVCSDNEWDSRRARCNAPQELSDHEMDRLLEGLSEADFAGSETVSKLESYDSDSGIPPFIPSEEEDSYCKQPQTWNEAYGQMQKKASQLVLNKKCKSVTSGKELSNGGEDLLRSTDLINLSSRSPASMFGYETPHPSTKAGKGHQMCTGCSKPIGSAAQGCKRCGTLTEYGIRSRAQFSAGYRQ

>PpVAL1b Pp3c12_12690V3.1 *Physcomitrella patens*

MLTGDFPSSSQSRVRHAQFTNYIALPCGEEPMDTRGIFQSSFGKSTPAHGDITKKSSSALASTLSTCQVPGGASLLVGCVLGKSSSKPKHQIRGSIKRNPSERKKELLVTSSHDRGPTLNGKGRPGTMALPRSISINALRISNLNSPQPSQKFLGTTMTFLFDKVASITDCRSTGHFVLPKRKVEEHFPPINKPGGIWMTLVDAAGKEWSFEFCFWHSKESRIYYFKKFYPYVQSTDLCGGDTVFFSRLEPQGTLFMGFRKQKPSPPKDQSKRREQSNGGPICLANDWSPSPDELANPQEKPVTMSIDEGNCKYASERGTSQCKRKRKKEFPPVSLSKRTEVVDHLAADKALVEKKSTTRLSVSPEAPQHVDKISGVLGSKGKRLRVNVDEYSEWKDTQDLLRPAPGALPSVVTIEGHDFEEYEKPPILIKRSCCSLEARGESQWVKCDDCGSWRRLPADAFVPEKWNCSDNDRDLTRAYCNAPQEPIHHDSDQLQRFGLDVEGRIKDESESREEVYEYDWATPEQHHSARADACDYKQPTLWSAAMTLIDGSLRRGTDSYSQLEKCSTYTSDSDSLDQSPSSALQSPRLEQFCELIEESSPMSTNSHATEAGRDLEVSTDCDEPIDSAGNGKRYEMTTTECYDFLGPPGK

>PpVAL2a Pp3c11_21940V3.2 *Physcomitrella patens*

MDCGGEGTESDKNVSLDRTKAGGAVGGGAAAGSSQSAGQGSLRVARPPGEGRGRNQLLPRYWPRITDQELKQINSGDTQTTITPLFEKMLSASDAGRIGRLVLPKACAEAYFPPIHQPEGLPLRIQDVTGRDWVFQFRFWPNNNSRMYVLEGVTPCIQSMKLHAGDTVTFSRLEADGKLVMGYRKAPTSLSSQDAGATRVGPNASNGLSNPSSAVIQTADGWASNVGAPKSKESGVSIGQLWSSQMDRKRGRPLGSKSKRLRLDSIDSMLLKSNWEEAQELLRPAPSASSTVITIDGHEFEEYSEPPVLAKRTFITKAPSGAQEQWAQCDDCGTWRRVPVDAFVPARWSCSQNSWDQTRAQCSAAQEVSSDKLEVLLGPGPTSEGGKQEYAPKVTPTSREENVSTTSAAAGLDTLAQAASTSLSPVRTTKHPRHRPGCTCIVCIQPPSGKGPKHKASCICNVCVTVKRRFRTLMQRRKKRQCEREFEAFRNKREWQEEDASEIADIGRAGTVDAANDVDDDLPTPPVCSRDGVSEDGPVKGAIDLNIQPDHEERVSMVRLLQDAHLPLSAYLHQQRLSSLYVGDLVSDHVSNDATSSHEDTLSESSSKAEADNELEVAGHVRHEAQRTQTSLGYVEQPNFVESWDARGVKPSEFSGDSVHSSSSKFEFTMMNNVAASSAKKHVTGAGRGRQICIGCGEPIGSAAKERKNLDAELGQKYMFRCCLCLNL

>PpVAL2b Pp3c7_7990V3.3 *Physcomitrella patens*

MTHLESGKVVDPAVRGWCEGFGAALAACQSDGGAWQPSSAARTSLSPPTGLIRPWSAPETDISMKSKDAADSVSREHMTLEVVEYGGEGTEIDRNPCSDRTKAGGAAGGSGSGSSQGPLRVARPPGEGRGRNQLLPRYWPRITDQELKQINSGDMQTTITPLFEKMLSASDAGRIGRLVLPKACAEAYFPPIHQPEGLPLRIQDVTGRDWVFQFRFWPNNNSRMYVLEGVTPCIQSMKLHAGDTVTFSRLEADGKLVMGYRKAPTSLSSQDAGATRVGANSNTGFSNPSSVVIQSADGWGSNVGGSKSKESGISIGNLWSSQMDRKRGRPLGSKSKRLRLDSIDSMLLKSNWEEAQELLRPAPSASSTVVTIDGHEFEEYSEPPVLSKRTFISNAPSGAQEQWAQCDDCGTWRRVPVDAFVPARWSCSQNTWDLTRAQCSAAQEVSSDKLEVLLGPGPTSEGGKQECAPKLTSTSKDENAPTTSAAAGLDALAQAASTSLSPARTTKHPRHRPGCTCIVCIQPPSGKGPKHKASCICNVCLTVKRRFRTLMQRRKKRQCEREFEAFRNKREWHEEDTSDLVGVVGRSGTADAANIVDDNLSTALIRSRDGGSEDGPLKGAIDLNIQPDHEERVSMSNQTVWRAGMQEESSPQSFQEIQCSQAHQN

>PpVAL2c Pp3c4_10850V3.1 *Physcomitrella patens*

MLGGDFPTLSQSQVRHAQFSNHIGSTCGEESMERRGTFHSFFGKSTPTQEGTTKKSSSALASTLSTCQIPVLGHHVCDFNSVSKSPGGTSILVGCVLGKAIVKPKQQIRGSVKRNPGEDRKELLATSPLNNGPTLNGKGRTGILTFPRSISVNGPRIGNLNSPPPSQKLFFSLGTTMTFLFDKVASVTDCRSTGHFVLPKRKVEEHFPPINKPGGIWMTLVDATGKEWSFEFCFWHSKESRIYYFKKFYPYVQSTDLRGGDTVFFSRLEPQGTLFIGYRKQKPPPPKQMKALNCGASKDQFKEREHSNGGLTYLANDWSPSLEEVATRQKKPTKLSADDANVDYALDRGIIQRKKKRKKEFPPLSLAQRTKVIDHLAVGEALIDKKSTSGRSSTPDTFQHDDRIAGVLGSKGKRLRVNADEYAEWKEMQDLLRPAPGALPTVLTIEGHDFEEYEAPPVLITRTYHSPEAHGENQWVKCNDCGSWRRLSADAFVPAGWICSDSDLDQGRAYCNAPQELSDHEIHQLLGLGLDAGKYQCHLNCVVFHISSKQPDFLVIVYNEQEGRTETQEDLNEDGDWTTPEHLYSAQADVCAYKQPTLWSAAMALVDGSLHRGTESYSELKSCESDTSVPEFTDHGPGMALQSPRLKQFCDLIEDSSSRRSTFLETRGRKVYENPTDSDEFVDPAGAAKGYEIPRADCYDFLGPVGKVLTPRSEIQPVTQHPLTTM

>CrVAL1 Cre13.g562400.t1.2 *Chalamydomonas reinhardtii*

MGDQVGGIVLPTTGDAGDGAGPPNFAALAGLLGGHPGAAAGFAPAPGAPGQPHPALSGLALSGMSHLAHMDPSTALLLSGLTAQQPGGGDAAAAQMQFLHNPTAAYAAAAAAAAAAAAAGQPAGVPVVDHGHHHHAHHAHPVAGGADGGASGVKVEHDGGGGGGVGDGGEENAAVRAAAAAAAAADPSDPEAVAAALAGRMAALGASVIFEKSLTASDVSGGGRVVVPKSIAEQYFPRLEAPSGVTISAADLEGRAYTFKWRFWVNNSSRMYLLEGAGELHRNYGLEVGDVMVFAQKQDGSLVVAGRCANKADMVKKQPVKRPNTAAAATAAVQAQPGPAAGRGGGRDARSGRTPARTSTPAAELAAPVTAQSPGAAAAAAAAAAAAATAAGVAMGGVRNGAGMGAGAGAAAAAADRGRKRKGPGSGAGQWAGGAVDGGRGGAARSGGRAGGGGGYPGYGYEGGAGVGAGGEEVCLSVLDMEAPSDGVFRAVVLPANGTSGGHASTGAGTGGSSPGVGVGVALARNNRWTATLDVAGELYQAYFDCRDDAVEALTAAGASP

>VcVAL1 Vocar.0028s0123.1 *Volvox carteri*

MSQPAETTGQDGLPSGGLVTGLPSTAPQAIPPSGHIQFDPAMTHLLSGIAAQLQAAAAAAASASPAAGPAQVDPPQPQPQTAGTPQAAAPQPQSPPSTSPYAAPDGLALKPEAVPAFGGLGGLGGLGLDLTHPGPPLLACPSVPGPGPGLTPGFGGGSTLAAATAAATAGGAATVAATAPKSAGAPFDPEALAAALTARMSAAGASIIFEKALTASDVSGGGRVVVPKSIAEQYFPKLEQPSGVTISATDLDGRSYTFKWRFWVNNSSRMYLLEGAGELHRNYGLEVGDVMVFAQKADGSLMVAGRAASKSDQVKRPPAKRPATAAVAGGSSGGGGAGAGGGGGGQAPRSRDARAPRPRPAVAASAGAAAEGAAAAAAAAQPIPGRLTAGGGPSTAVASADASLLRGGGGGGRKRKVSSSAATAAAATGAAVGGLAADPTRFRVRPGDEGLTSLAVLDMEAPSDGIFRAVVQHVSPVSLGVALARNNRWTATLEVAGEFYQAFFDSREDAVEAVTAAGASP

**Associated factors: AL1-7**

>AtAL1 At5g05610 *Arabidopsis thaliana*

maaessnprtveeifkdfsgrrsgflralsvdvdkfyslcdpemenlclyghpngtwevnlpaeevppelpepalginfardgmqrkdwlslvavhsdcwllsvssyfgarlnrnerkrlfslindlptlfevvtgrkpikdgkpsmdlgsksrngvkrsiegqtkstpklmeesyededdehgdtlcgs cggnytndef wiccdvcerw yhgkcvkitp akaesikqyk cpscctkkgrq

>AtAL2 AT3G11200 *Arabidopsis thaliana*

maaaavssnprtveeifkdysarraallraltkdvddfysqcdpekenlclyghpneswevnlpaeevppelpepalginfardgmqrkdwlslvavhsdcwllsvsfyfgarlnrnerkrlfslindlptlfdvvtgrkamkdnkpssdsgsksrngtkrsidgqtksstpklmeesyeeeeeedehgdtlcgscgghytneefwiccd vcerwyhgkc vkitpakaes ikqykcppccakkgrq

>AtAL3 AT3G42790 *Arabidopsis thaliana*

meggaalynprtveevfkdfkgrrtaivkalttdvqefyqqcdpekenlclyglpneewevnlpaeevpp elpepalginfardglsekewlslvaihsdawllsvsfyfgsrfsfhkeerkrlfnmindvptifevvtgmakakdkssaanqngnksksnskvrtsegkssktkqpkeedeeideddeddhgetlcgacgdsdgadefwiccdlcekwf hgkcvkitpa raehikqykcpscsnkrara

>AtAL4 AT5G26210 *Arabidopsis thaliana*

meaggaynprtveevfrdfkgrragmikalttdvqeffrlcdpekenlclyghpnehwevnlpaeevppelpepvlginfardgmaekdwlslvavhsdawllavafffgarfgfdkadrkrlfnmvndl ptifevvagt akkqgkdkssvsnnssnrskssskrgsesrakfskpepkddeeeeeegveeededeqgetqcgacgesyaadefwiccdlcemwfhgkcv kitparaehikqykcpscsn krars

>AtAL5 AT5G20510 *Arabidopsis thaliana*

meggtahysprtveevfrdfkgrragiiqalttdvedffqqcdpekqnlclygfpnevwevnlpaeevppelpepalginfardgmqernwlslvavhsdawllsvsfyfgsrfgfdradrkrlfsminevptvyevvtgnaekqtkempssanqngnrsksnskmrgleskssktihakdeeegleleegeeeedededehgetlcgac gdnyasdefwiccdmcekwf hgecvkitparaehikhykc ptcsnkrarp

>AtAL6 AT2G02470 *Arabidopsis thaliana*

megithpiprtveevfsdfrgrraglikaltndmvkfyqtcdpekenlclyglpnetwevnlpveevppelpepalginfardgmqekdwvslvavhsdswllsvafyfgarfgfgknerkrlfqminelptifevvsgnakqskdlsvnnnnskskpsgvksrqseslskvakmsspppkeeeeeedesedeseddeqgavcgacgdny gtdefwiccd acekwfhgkc vkitpakaehikhykcptcs nkrarp

>AtAL7 AT1G14510 *Arabidopsis thaliana*

megiqhpiprtveevfsdfrgrraglikalstdvqkfyhqcdpekenlclyglpnetwevnlpveevppe lpepalginfardgmqekdwislvavhsdswlisvafyfgarfgfgknerkrlfqmindlptifevvtgnakqskdqsanhnssrskssggkprhseshtkaskmsppprkedesgdededdeqgavcga cgdnyggdef wiccdacekwfhgkcvkitp akaehikhykcpscttskkm ka

>BsAL1 Bostr.13129s0409.1 *Boechera stricta*

MAADSSNPRTVEEIFKDFSGRRSGFLRALSVDVDKFYSLCDPEMENLCLYGHPNGTWEVSLPAEEVPPELPEPALGINFARDGMQRKDWLSLVAVHSDCWLLSVSSYFGARLNRNERKRLFSLINDLPTLFEVVTDRKPIKDNKPSMDLGSKSRNGVKRSIEGQTKSTLKLMEESYEDEDDEHGDTLCGSCGGNYTNDEFWICCDVCERWYHGKCVKITPAKAESIKQYKCPSCCTKKGRQ

>BsAL2 Bostr.19424s1053.1 *Boechera stricta*

MAAAAVSSNPRSVEEIFKDYTARRTALLRALTKDVDDFYSQCDPEKENLCLYGHPNESWEVNLPAEEVPPELPEPALGINFARDGMQRKDWLSLVAVHSDCWLLSVSFYFGARLNRNERKRLFSLINDLPTLFDVVTGRKPIKDNKPNSDSGSKSRNGTKRSIDGQTKSSTPKLMEESYEEEEEEDEHGDTLCGSCGGNYTTDEFWICCDVCERWYHGKCVKITPAKAESIKQYKCPPCCAKKGRQ

>BsAL3 Bostr.0556s0654.1 *Boechera stricta*

MEGGAALYNPRTVEEVFKDFKGRRAAIVKALTTDVQEFYQQCDPEKENLCLYGLPNEEWEVNLPAEEVPPELPEPALGINFARDGLSEKEWLSLVAIHSDAWLLSVSFYFGSRFSFHKEERKRLFNMINDVPTIFEVVTGMAKKQTKDKPSAANQNGNKSKSNSKVRTSDGKSSKTMQANEEDEGVDEEDEDDHGDTLCGACGDSDGADEFWICCDLCEKWFHGKCVKITPARAEHIKQYKCPSCSNKRARA

>BsAL4 Bostr.29827s0195.1 *Boechera stricta*

MDAGGAYNPRTVEEVFRDFKGRRAGMIKALTTDVQEFYRLCDPEKENLCLYGHPNEHWEVNLPAEEVPPELPEPVLGINFARDGMAEKDWLSLVAVHSDAWLLAVAFFFGARFGFDKADRKRLFNMVNDLPTIFEVVTGTAKKQGKDKSSVSNNSSNRSKSNSKRGSDSRAKFSKAEPKDEEDEEGVEEEDEDEQGETQCGACGESYAADEFWICCDLCEMWFHGKCVKITPARAEHIKQYKCPSCSNKRARV

>BsAL5 Bostr.26527s0153.1 *Boechera stricta*

MEGGGGGGAAHYNPRTVEEVFRDFKGRRAGIIQALTTDVEDFFQQCDPEKENLCLYGFPNEVWEVNLPAEEVPPELPEPALGINFARDGMQEKDWLSLVAVHSDAWLLSVSFYFGSRFGFDKADRKRLFSMINEVPTVFEVVTGNAKKQTKEKPSSANQNGNRSKSNSKVRGLDGKSSKTILAKDEEQGVEKEDEEEEEDEDEHGETLCGACGDNYASDEFWICCDMCEKWFHGKCVKITPAKAEHIKHYKCPSCSNKRARP

>BsAL6 Bostr.0556s0070.1  *Boechera stricta*

MEGITHPIPRTVEEVFSDFRGRRAGLIKALTNDMVKFYQTCDPEKENLCLYGLPNETWEVNLPVEEVPPELPEPALGINFARDGMQEKDWVSLVAVHSDSWLLSVAFYFGARFGFGKNERKRLFQMINELPTIFEVVTGNAKQSKDQSGNNNNSKSKSTGVKSRQSESLTKVAKMSSPPPKDEDESEDEEEDDEQGAVCGACGDSYGTDEFWICCDACEKWFHGKCVKITPAKAEHIKHYKCPSCSNKRARP

>BsAL7 Bostr.24340s0015.1  *Boechera stricta*

MEGIQHPIPRTVEEVFSDFRGRRAGLIKALSTDVQKFYHQCDPEKENLCLYGLPNETWEVNLPVEEVPPELPEPALGINFARDGMLEKDWISLVAVHSDSWLISVAFYFGARFGFGKNERKRLFQMINDLPTIFEVVTGNAKQSKDQSANHNSNKSKSNGGKPRHSESHTKASKMSPPPREEDESGDEEEDDEQGAVCGACGDNYGGDEFWICCDACEKWFHGKCVKITPAKAEHIKHYKCPSCSTSKKIKA

>BrAL1 Bra009121 *Brassica rapa*

MAGESSNPGTVEEIFKDFRGRRSAFLQALSVDVDKFYSLCNPEMENLCLYGHPNGTWEVNLPAEEVPPELPEPALGINFARDGMQRQDWLSLVAVHSDCWLLSVSSYFGARLSRNEKNRLFSLINDLPTLFEVVTGRKTIKDKPSMDHESKYQNGLKRSIEGEMKITRKLMEESCEDEEDEHGDTLCGSCGGHYLNVEFWICCDVCERWYHGKCVKITPAKAESMKQYKCPSCCTKKGRQ

>BrAL2a Bra001393 *Brassica rapa*

MAAVSSNPRTVEEIFKDYSARRSALLRALTKDVDDFYSQCDPEKENLCLYGHPNESWEVNLPAEEVPPELPEPALGINFARDGMQRKDWLSLVAVHSYIYCGLTHVYGRLDCCYFSSFLHLWKRLFSLINDLPTLFDVVTGRKPIKDNKPSSDSGSKSRNGTKRSIEGQTKSPTPRLMEESYEDEDEEEDEHGDTLCGICGGNYTQDEFWICCDVCERWYHGKCVKITPAKADSIKQYKCPPCCAKKGRQ

>BrAL2b Bra034860 *Brassica rapa*

MAAVSSNPRTVEEIFKDYTSRRSALLRALTKDVDDFYSQCDPEKENLCLYGHPNESWEVNLPAEEVPPELPEPALGINFARDGMQRKDWLSLVAVHSDCWLLSVSFYFGARLNRNERKRLFSLINDLPTLFDVVTGRKPIKDNKPSSDSGSKSRNGTKRSIEGQPKSTTPKPMEGSYEDEDEDEEEDEHGDTLCGICGGNYTQDEFWICCDVCERWYHGKCVKITPAKADSIKQYKCPPCCAKKGRQ

>BrAL2c Bra034169 *Brassica rapa*

MAAAVSSNPRTVEEIFKDYTSRRAALLRALTKDVDGFYSQCDPEKENLCLYGHPNESWEVNLPAEEVPPELPEPALGINFARDGMQRKDWLSLVAVHSDCWLLSVSFYFGARLNRNERKRLFSLINDLPTLFDVVTGRKPIKDNKPSSDSGSKSRNGGTKRSIEGQPKSTTPKLMEERYEEEEDEEEEDEHGDTLCGSCGGNYTQDEFWIGCDVCERWYHGKCVKITPAKADSIKQYKCPPCCAKKGGRQ

>BrAL3 Bra034950 *Brassica rapa*

MEGGAGLYNPRTVEEVFRDFKGRRAAIVKALTSDVGEFYQQCDPEKENLCLYGLPNEQWEVNLPAEEVPPELPEPALGINFARDGLSEKEWLSLVAIHSDTWLLSVSFYFGSRFAFDKADRKRLFDMINGVPTIFEVVTGNMKKQTKEKSSAANRYGNLSKSDSKVRSSDGKNSKAVQASNEEDGSEEEDEDEHGETLCGACGDGDGTGTDDFWICCDVCEVWFHGKCVKITPARAEHIKQYKCPACSNKRARP

>BrAL4a Bra036568 *Brassica rapa*

MDGGGAYNPRTVEEVFRDFKGRRSGMIKALTSDVQEFYRLCDPEKENLCLYGRPDEHWEVNLPAEEVPPELPEPVLGINFARDGMQEKDWLSLVAVHSDAWLLAVAFFFGARFGFDKADRKRLFNMMNDLPSIFEVVAGTAKKPSKEKSSVSNNSSNRSKSNSKRGSEPRPKLTKPEPKDEEEEEEEGVEEEEDEDDEQGETQCGACGESYAADEFWICCDLCENWFHGKCVKITPARAEHIKQYKCPSCSNKRARS

>BrAL4b Bra009887 *Brassica rapa*

MDGGGDGGGAAYNPRTVEEVYRDFKGRRNGMIKALTTDVQEFYRLCDPEKENLCLYGHPNEHWEVNLPAEEVPPELPEPVLGINFARDGMMEKDWLSLVAVHSDAWLLAVAFFFGARFGFDKADRKRLFNMMNDLPSIFEVVAGTAKKASKEKSSVSNNSSNRSKSNSKRGSEPKPKYSKPEPKDEEEEEGVEEEDEDDDDEQGETQCGACGESYAADEFWICCDLCENWFHGKCVKITPARAEHIKQYKCPSCSNKRARS

>BrAL4c Bra020543 *Brassica rapa*

MNRDGAYNPRTVEEVFRDYNGRRNGMIKALTTDVHEFHRLCDPEKENLCLYGLPSEDWEVNLPAEEVPPELPEPVLGINFARNGMLEKDWLSLVAVHSDAWLMAVAFFFGARFGFDKADRKRLFNMVNDLPTVFEVVTDFLARKLSKEKYSVSNNSSNRSKSNSKRGGSEARPIAKPAPKEEEEEEEENDDDDEEGDTACGACGETYARDEFWICCDMCENWFHGKCVKITPARAEHIKQYKCPSCSNNKRGRS

>BrAL5a Bra002307 *Brassica rapa*

MEGGGGGAQYNPRTVEEVFRDFKGRRAAILRALTTDVQEFFQQCDPEKDNLCLYGFPNEVWEVNLPAEEVPPELPEPALGINFARDGMMEKDWLSLVAVHSDAWLLSVSFFFGSKFGFDKVDRKRLFNMINEVPTIFEVVTGTAKNQTKEKASSANQNGNRSKSNSKVRGLDGKSSKTIQAMEEGGLEEEEEEKEKEEDEEEHGETLCGACGDNYASDEFWICCDMCEKWFHGTCVKITPARAEHIKHYKCPSCSNKRARPQNVLFHLLVLAIYLSIHHPKL

>BrAL5b Bra020107 *Brassica rapa*

MEGGGGARYNPRTVEEVFRDFKGRRAGILRALTTDVKEFFQQCNPEKDNLCLYGFPNEVWEVNLPAEEVPPELPEPALGINFARDGMQEKEWISLVAVHSDAWLLSVSFYFGSRFGFDKADRKRLFNMINEVPTVFEVVTGSAKKQTEEQPSSVNRNGNRSKSNSKVRDLEVKSSRTIEVNDEEEGVEEEEEDEEEHGETLCGACGDNYASDFWICCDMCEKWFHGKCVKITPARAEHIKQYKCPSCSNKRARP

>BrAL6a Bra017415 *Brassica rapa*

MEVITHPTPRTVDEVFTDFRGRRAGLIKALTTDMVKFYQTCDPEKENLCLYGLPNETWEVNLPVEEVPPELPEPALGINFARDGMQEKDWVSLVAVHSDSWLLSVAFYFGARFGFGKNERKKLFQMINELPTIFEIVTGNAKQSKDLSGNNNTSSKSKSNGLKVPKMSSPPPKEEDESEDEDEDDEQGAVCGACGDSDGTDEFWICCDACEKWFHGKCVKITPAKAEHIKHYKCPSCSNKRARP

>BrAL6b Bra024817 *Brassica rapa*

MEGSNIITQHTPRTVEEVFSDFRGRRAGLIKALTIDMVKFYQTCDPEKENFCLYGLPNETWEVNLPAEEVPPELPEPALGINFARDGMQEKDWVSLVAVHSDSWLLSVAFYFGARFGFVKNERKRLFQMINDLPTIFEVVTGNAKQSKDHSGTNNNNSISKPSGLKVSKMMSSPPPKEEDESEDEEEEQGAVCGACGDNYGTDEFWICCDACEKWFHGRCVKITPAKAEHIKHYKCPSCTNKRARP

>BrAL6c Bra026210 *Brassica rapa*

MEGRAAEDVFKDFRGRRAGLIKALTTDVRKFYDKCDPANENLCLYGLPNGSWELNLPVDEVPPELPEPALGINFSRDGMPEENWVSLIAVHSDSWLIAVAFYFGARFSFDKNERMRLFKMINDLPTIFEVVTGNAKQSKDQSGNSGRSKSGGVKARQSESQTKAAKRSPPPPSREESESGDDDGEEGEGQGEFCGVCGESDDGEEEFWIWCDTCEKWFHGKCVKITPARAEHIKHYKCPSCCGSNKKHRA

>BrAL7a Bra016698 *Brassica rapa*

MEGIQNPIHRTVEEVFSDFKGRRAGLLKALTTDGQKFFLQCDPEKENLCLYGLPNETWEVNLPVDEVPPELPEPALGINFARDGMPEKDWITLVAVHSDSWLISVAFYFGARFGFGKNERKRLFQMINELPTIFELITGNAKQSKDQSANHNSSRSKSSGVKPRQSESHTKASKMSPPPREDDESGEDEEDDEQGAVCGACGDNYDDFWICCDACEKWFHGKCVKITPAKAEHIKHYKCPTCSTNKKMRA

>BrAL7b Bra026825 *Brassica rapa*

MEGIQNPVVSRSVEEVFGDFRGRRAGLLKALSTDVRKFYHECDPDKENLCLYGLPNETWEVNLPVDEVPPELPEPALGINFARDGMPEKDWISLVAVHSDSWLISVAFYFGARFGFGKNERKRLFQMINDLPTIFEVITGNAKQTKDQSANNHNSSRTKPSGVKASKTSPPPREDDDSGDDEEDDEQGAVCGACGDNHDDFWICCDACEKWFHGKCVKITPAKAEHIKHYKCPTCSINKKIKAS

>CgAL1 Cagra.1504s0063.1 *Capsella grandiflora*

MAAESSNPRTVEEIFKDFSGRRSGFLRALSGDVDKFYSLCDPEMENLCLYGHPNGTWEVNLPAEEVPPELPEPALGINFARDGMQRKDWLSLVAVHSDCWLLSVSSYFGARLNRNERKRLFSLINDLPTLFEVVTGRKPIKDNKPSMDLGSKSRNGVKRSIEGQTKSTPKLMEESYEDEDDEHGDTLCGSCGGNYTNDEFWICCDVCERWYHGKCVKITPAKAESIKQYKCPSCCTKKGRQ

>CgAL2 Cagra.1655s0051.1 *Capsella grandiflora*

MAAAAVSSNPRTVEEIFKDYTARRTALLRALTKDVDDFYSQCDPEKENLCLYGHPNESWEVNLPAEEVPPELPEPALGINFARDGMQRKDWLSLVAVHSDCWLLSVSFYFGARLNRNERKRLFSLINDLPTLFDVVTGRKPIKDNKPSSDSGSKSRNGTKRSIDGQTKSSTPKLMEESYEDEEEEDEHGDTLCGSCGGNYTTDEFWICCDVCERWYHGKCVKITPAKAESIKQYKCPPCCAKKGRQ

>CgAL4 Cagra.0998s0009.1 *Capsella grandiflora*

MDAGGAAYNPRTVEEVYRDFKGRRAGMIKALTTDVQEFYRLCDPEKENLCLYGHPNENWEVNLPAEEVPPELPEPVLGINFARDGMAEKDWLSLVAVHSDAWLLAVAFFFGARFGFDKPDRKRLFNLVNDLPTIFEVVTGTEKKQGKDKSSVSNNSSNRSKSKSNSKRGSDSRAKFSKPEPKEEEEEEDVEDEEDEDEDEQGETQCGACGESYAADEFWICCDLCEMWFHGKCVKITPARAEHIKQYKCPSCSNKRARA

>CgAL5 Cagra.0179s0029.1 *Capsella grandiflora*

MDGGGGGAAHYNPRTVEEVFRDFKGRRAGIIQALTTDVEDFFQQCDPEKENLCLYGFPNEVWEVNLPAEEVPPELPEPALGINFARDGMQEKDWLSLVAVHSDAWLLSVSFYFGSRFGFDKADRKRLFNMINEVPTVFEVVTGNAKRQTKEKPSSANQNDNRSKSNSKLRGLDGKSSKTIQAKEEEQGSEEVEVEEEEEEEEEEEEDEDEHGETLCGACGDNYASDEFWICCDMCEKWFHGKCVKITPARAEHIKHYKCPSCSNKRARP

>CgAL6 Cagra.0534s0045.1 *Capsella grandiflora*

MEGISHPIPRTVEEVFSDFRGRRAGLIKALTNDMVKFYQTCDPEKENLCLYGLPNETWEVNLPVEEVPPELPEPALGINFARDGMQEKDWVSLVAVHSDSWLLSVAFYFGARFGFGKNERKRLFQMINELPTIFEVVTGNAKQSKDQSGNNNNSKSKSSGIKSRHSESLTKVAKMSSPPPKDDDESEDEDEDDEQGAVCGACGDNYGTDEFWICCDACEKWFHGKCVKITPAKAESIKHYKCPSCSNKRARP

>CgAL7 Cagra.3501s0049.1 *Capsella grandiflora*

MEGIQHPIPRTVEEVFSDFRGRRAGLIKALSTDVQKFYHQCDPEKENLCLYGLPNETWEVNLPVEEVPPELPEPALGINFARDGMLEKDWISLVAVHSDSWLISVAFYFGARFGFGKNERKRLFQMINDLPTIFEVVTGNSKQSKDQSANHNSSKSKSSGGKPRHSESHTKASKMSPPPREEDESGDEEEDDEQGAVCGACGDNYGGDEFWICCDACEKWFHGKCVKITPAKAENIKHYKCPSCSTSKKMKA

>OrAL1a orange1.1g026158m *Citrus sinensis*

MSSATSSPRTVEEIFKDFKARRSALVRALTYDVDQFYSQCDPEKENLCLYGHPNESWEVTMPADEVPPEIPEPALGINFSRDGMCKKDWLSLVAVHSDCWLVAVAFYFGARLNGNERKRLYSLINDLPTLFEVVTGRISVKDNQPGADGRSKSWNSTKRSIDGQARSKHELLEESLGEVDDAENDETFCGSCGGSYNSAQFWIGCDICERWYHGKCVKITPAKAENIKQYKCPSCSTKKARH

>OrAL1b orange1.1g026373m *Citrus sinensis*

MEMASSPRTVEEIYKDFCARRAGVVRALTNDVDEFYGLCDPDKENLCLYGHPNESWEVTLPAEEVPPELPEPALGINFARDGMNRKDWLSLVAVHTDSWLLSVAFYLGARLNRNERKRLFSLINDQPTVFEVVTERKPIKDKPSVDSSSKSRGSTKRSNDGQVKSNPKLADESFDDEEDEHSETLCGSCGGNYNADEFWIGCDICERWFHGKCVKITPAKAENIKQYKCPSCSMKRGRQ

>OrAL4 orange1.1g025377m *Citrus sinensis*

MDGGGGYNPRTVEEVFGDFKGRRAGMIKALTTEVEEFYHQCDPEKENLCLYGFPSEQWEVNLPAEEVPPELPEPALGINFARDGMQEKDWLSLVAVHSDAWLLSVAFYFGARFGFDKSDRKRLFNMINELPTIFEVVTGTTKKQAKEKSSVSNHSSSKSKSNSKRGSETQAKFSKAVQSKDEEDEGLEEEDEEEHGETLCGACGENYAADEFWICCDVCEKWFHGKCVKITPARAEHIKQYKCPSCSNKRARP

>CsAL1a Cucsa.368540.1 *Cucumis sativus*

MASISSSPRSVEDIFKDYNARRTGLVRALTYDVDEFYSLCDPEKENLCLYGHPNESWEVTLPAEEVPSELPEPALGINFARDGMKRRDWLSLVAVHSDCWLLSVAFYFGAQLNRNERKRLFSMINDLPTLFEVASGRKAVKDKPSMDSGSKSRNSTKRTLDGSTRNSNPKLLEESYGEDEDEHGDTLCGSCGGNYNADEFWIGCDICEKWFHGKCVRITPAKAENIKQYKCPSCSTKRGRL

>CsAL1b Cucsa.176400.1 *Cucumis sativus*

MEIPSTTPKTVEEIFKDYTARRAALVRALAHDVDEFYGLCDPDKENLCLYGHPNETWEVVLPVEEVPPELPEPALGINFARDGMNRKDWLSLVAVHSDSWLLSVAFYFGARLNRNERKRLFSLMNDLPTVFEVVTERKPVKEKPSVDSGSRSQGSSKRSNDGQVKSNPKLAEQSCEEDVDEHSETLCGSCGENYSADEFWIGCDICERWYHGKCVRITPAKADSIKQYKCPSCSMKKGRQ

>CsAL4 Cucsa.056090.1 *Cucumis sativus*

MDGGAPYNPRTVEEVFRDFKGRRAGLIKALTTDVEEFYQQCDPEKENLCLYGFPSEQWEVNLPAEEVPPELPEPALGINFARDGMQEKDWLSLVAVHSDVWLLSVAFYFGARFGFDKTDRKRLFNMINDLPTIFEVVTGTAKKQVKDKSSVSNHSSNKSKSNSKRSSEPQIKTTKAVQSKDEEEEGGLEEEEEEEHGETLCGACGENYASDEFWICCDICEKWFHGKCVKITPARAEHIKQYKCPSCSNKRIRP

>CsAL5 Cucsa.360370.1 *Cucumis sativus*

MDGGGAVHNRSVEDVFSDFRGRRNGVIKALTVDVEEFYQQCDPEKENLCLYGFPNEVWEVNLPAEEVPPELPEPALGINFARDGMQERDWLALVAVHSDSWLLSVAYYFGARFGFDKSDRRRLFNMINDLPTVFEVVTGIAKKQVKEKSSTANGSKSKSSFKSREAEMQGMYARQSQAREEVETLYEEDEDEHGDTLCGACGENYASDEFWICCDICEKWFHGKCVRITPAKAEHIKQYKCPSCSNKRSRI

>CsAL6 Cucsa.260440.1 *Cucumis sativus*

MEGMPHPIPRTVEEVYGDFKGRRAGLIKALTTDVEKFYQQCDPEKENLCLYGLPNETWEVNLPVEEVPPELPEPALGINFARDGMQEKDWLSLVAVHSDSWLLAVAFYFGARFGFGKTERKRLFQMINDLPSVFEVVTGNGKQSKEQSATHNNGSKSKSSGKMSRQLESHSKGVKMSPPPKEDEDSGDEEEEEEDDEQGATCGACGDNYGNDEFWICCDACERWFHGKCVKITPAKAEHIKQYKCPSCSNKRARV

>EsAL1 Thhalv10014538m *Eutrema salsugineum*

MAAESSNPRTVEEIFKDFSGRRSGFLKALSVDVDKFYSLCDPEMENLCLYGHPNGTWEVNLPAEEVPPELPEPALGINFARDGMQRKDWLSLVAVHSDCWLLSVSSYFGARLNRNERKRLFSLINDLPTLFEVVTGRKPTKDKPSMDLGSKSKNGVKRSIEGQTKSTPKLMEESYEDEEDEHGDTLCGSCGGNYTNDEFWICCDVCERWYHGKCVKITPAKAEGIKQYKCPSCCSKKGRQ

>EsAL2 Thhalv10021406m *Eutrema salsugineum*

MAAVSSNPRTVEEIFKDYTARRSALLRALTKDVDDFYSQCDPEKENLCLYGHPNESWEVNLPAEEVPPELPEPALGINFARDGMQRKDWLSLVAVHSDCWLLSVSFYFGARLNRNERKRLFSLINDLPTLFDVVTGRKPIKDNKPSSDSGSKSRNGTKRSIEGQTKSTTPKLTEESYEDEEEEDEHGNTLCGSCGGNYTEDEFWICCDVCERWYHGKCVKITPAKADSIKQYKCPPCCAKKGRQ

>EsAL4 Thhalv10004808m *Eutrema salsugineum*

MDGGAAYNPRTVEEVFRDFKGRRAGMIKALTTDVQEFYRLCDPEKENLCLYGHPNEHWEVNLPAEEVPPELPEPVLGINFARDGMLEKDWLSLVAVHSDAWLLAVSFFFGARFGFDKADRKRLFNMMNDLPTIFEVVAGTAKKQSKDKSSFSNNSSNRSKSNSKRGSEPRPKFSKAEPKDEEEEEGVEEEDEDEQGETQCGACGESYAADEFWICCDLCENWFHGKCVKITPARAEHIKQYKCPSCSNKRARS

>EsAL5 Thhalv10014469m *Eutrema salsugineum*

MEGGGAQYNPRTVEEVFRDFKGRRAGIVRALTTDVEDFFQQCDPEKENLCLYGFPNEVWEVNLPAEEVPPELPEPALGINFARDGMQEKDWLSLVAVHSDAWLLAVSFYFGSRSGFDKADRKRLFNMINELPSIFEVVTGNAKKQTKEKTSSANQNGNKSKSTSKVRGLDGKHSRTMQVKDEEEGVEEEEEDEDEHGETLCGACGDNYASDEFWICCDMCEKWFHGKCVKITPARAEHIKHYKCPSCSNKRARS

>EsAL6 Thhalv10004815m *Eutrema salsugineum*

MEGITHPIPRTVEEVFSDFRGRRAGLIKALTNDMVKFYQTCDPEKENLCLYGLPNETWEVNLPVEEVPPELPEPALGINFARDGMQEKDWVSLVAVHSDSWLLSVAFYFGARFGFGKNERKRLFQMINELPTIFEVVTGNAKQSKDLSANNNNSKSKSSGIKSRQSESLTKVSKMSSPPPKEDDESEDEEEDDEQGAVCGACGDSYGTDEFWICCDACEKWFHGKCVKITPAKAEHIKQYKCPSCSNKRARP

>ESAL7 Thhalv10008569m *Eutrema salsugineum*

MEGIQNPIPRTVEEVFSDFRGRRAGLIKALSTDVQKFFHQCDPEKENLCLYGLPNETWEVNLPVEEVPPELPEPALGINFARDGMPEKDWISLVAVHSDSWLIAVAFYFGARFGFGKNERKRLFQMINDLPTIFEVVTGNAKPAKDQSANHNSSRSKSSSGKPRHSESHTKASKMSPPPREEDDSGDEEEDDEQGAVCGACGDNYGGDEFWICCDACEKWFHGKCVKITPAKAEHIKHYKCPSCSTNKKIKA

>FvAL1 mrna18348.1-v1.0-hybrid *Fragaria vesca*

MEMATSPRTVEEIFKDYGARRAAVVRALTYDVDEFYGLCDPEKENLCLYGHPNETWEVTLPAEEVPPELPEPALGINFARDGMNRKDWLSLVAVHSDSWLLSVAFYFGARLNRSERKRLFSLINDLPTVFEVVTDRKPAKDKPSVDSGSKSRGSTKRSGDGMMKSNPKLADESFEEEDDEHSETLCGSCGGNYNADEFWIGCDICERWFHGKCVKITPAKAENIKQYKCPSCSLKRSRQ

>FvAL4 mrna07518.1-v1.0-hybrid *Fragaria vesca*

MAMNGAGAGAAYNARTVEEVFRDFKGRRTGLIKALTTDAEEFHQQCDPEKENLSLYAFPNEHWEVNLPAEEVPPEIPEPALGINFARDGMAEKDWLALVAVHSDAWLLSVAFYFGSRFGFDKHDRGVRGVDQGTKGAKNGKKLFGMINELPTIFEVVTGVAKKEVKDKPSSNHGSNKSKLNSKARGSESQARHLEVMPAKDEDEGLDEDEEDEHEEETCGACGRGGPSSLDEPWILCDFCETWFHMKCVKVTPAKAKQIKQYKCPSCSNKRARGIFEELYIRTTTKKAPVVLSKTTSAACGFVTRNTEGNTVAAVSRNIGKAAKLQ

>FvAL5 mrna15410.1-v1.0-hybrid *Fragaria vesca*

MAGARNYPRQTVEGVFRDFEGRRAGMIKAFTTDFEEFYEQCDPEEENLCLYGFPSEQWKVDLPEEEVPPELPEPTIGINFARDGMHKMDWLALVAVHGDAWLLAVAFYFASRFRFGKTDRKCLFNMINDLPSLFEVVTCTAEKKSSSSETMQSKYEHEEEKSVQDMEGGDPLSFEEVFRDFKGRRAGLLKAFTTEFEEFYQQCDPEKENVCLYGIPSEQWEVDLPEEEVPPELPHPAIGINFARDGMHKKDWLSLVAVHSDAWLLAVAFNFGARFKFNKTNRERLFDEINNLPTLFEVVESSIQKEVKKKSSSSENGSNESTSNLKDGSESQGKDAEVMQMQSKKEAEDSEESFEDERNMEVHCAGGCGLFCSFVGSICGMWQLTIGNGYGPCPSSEVKYFAVFSLLFFSVTPFSSLIATTAMDVAAPYSSRTVEEVFRDFKARRAGLLKALTTDVNLFFQQCDPGQPLRFHFRCVIEKENLCLYGFPSEQWKVNLPAEEVPPELPEPALGINFARDGMQEKDWLALVAVHSDAWLLAVAFYFGARFQFDKNDRKRLFNMINELPTIFEIVTGSAKKVKEKSTNSNHGNNKPKSNSKRGSESQGKYAKVMQAREDYEDGTDDEDNDDEHGETLCGACGENYASDEFWICCDVCEKWFHGKCVKITPARAEHIKQYKCPSCSNKRARP

>FvAL6 mrna29135.1-v1.0-hybrid *Fragaria vesca*

MDFCSELTTATRLSNGTLILVEAVEGVHIQTHAVLRQAWIEKLTPCLVINKIDRLISELEMSPREAYIRERMEGLPQHPAPRTVEEVFSDFRGRRAGLIKALTTDVQKFYQQCDPDKENLCLYGLPTEAWEVNLPVEEVPPELPEPALGINFARDGMAEKDWLSLVAVHSDSWLLAVAFYFGARFGFGKNDRKKLFQMINDLPTIFEVVTGSGRQPTNESALHHNSSKSKSSGKPSRQPEINPSKGVKMSPPAKEDEESGEEEEEDDEQGATCGACGDNYGADEFWICCDVCERWFHGKCVKITPAKAEHIKQYKCPSCSSKRARV

>GrAL1a Gorai.013G017700.1 *Gossypium raimondii*

MASSSPRTVEEIFKDYSARRSGLVRALTYDVDDFYSQCDPDKENLCLYGHPNEAWEVALPAEEVPPELPEPALGINFARDGMNRKDWLSLVAVHSDCWLLSVSFYFGARLNRNERKRLFSMINDLPTVFEVVTGRKPIKDKPTVESGSKSRNSTKRSIDGQPRSNPKLVDENYEEDEEEQGDTFCGICGGGYNSDEFWIGCDNCERWYHGKCVKITPAKAELIKFYKCPLCTKKARQ

>GrAL1b Gorai.011G289200.1 *Gossypium raimondii*

MASSSLRTVEEIFKDYNARRSALVRALTYDVDDFYSQCDPDKENLCLYGHPNEAWEVALPAEEVPSELPEPALGINFARDGMNKKDWLSLVAVHSDCWLLSVAFYFGARLNRNERKRLFSMMNDLPTVFEVVTGQKPVKDKPTVESGSKSRNSTKPNEDDKEEQGDTFCGSCGGGYNSDEFWIGCDNCERWYHGKCVKITPAKAEMIKVYKCPSCQKKARQ

>GrAL1c Gorai.010G196600.1 *Gossypium raimondii*

MASSSPRTVEEIFKDYNARHSALVRALTYDVDDFYSQCDPDKENLCLYGHPNEAWEVALPAEEVPPELPEPALGINFARDGMNRKDWLSLVAVHSDCWLLSVAFYFGARLNRNERKRLFSMINDLPTIFEVVTGRKQVKDKPTVESGNKSRNSTKRSLDGQPRSNPKIADNSYEEDEEEQGDTFCGICGGGYNSDEFWIGCDNCERWYHGKCVKITPAKAEMIKFYNCPLCQKKVRQ

>GrAL1d Gorai.012G114500.1 *Gossypium raimondii*

MASSARTVEEIFKDYTARRTAILRALTLDVDHFYGLCGPDRENLCLYGHPNESWEVSLPAEEVPAELPEPALGINFARDGMNRKDWLSLVAVHSDSWLISLAFYLAARLNRNERKRLFSMMNDLPTVFEVVTERKPVKDKPSIESGSKSQGSTKRSSDGQVKSNPKIADADYKDNEDEHGETLCGSCGGNYNADEFWIGCDVCERWFHGKCVKITPAKAESIKQYKCPSCSMRSGRH

>GrAL4a Gorai.011G057500.1 *Gossypium raimondii*

MEGGGAQYNPRTVEEVFRDFKGRRAGMIKALTTDVEEFYQQCDPEKENLCLYGFPSEQWEVNLPAEEVPPELPEPALGINFARDGMQEKDWLSLVAVHSDAWLLSVAFYFGARFGFDKADRKRLFNMINDLPTIFEVVTGGVAKKQTKEKSLVSNHSSNKSKSNSKVRESQAKHSKASQPKDEEQGLDEEDEDELGETLCGACGENYASDEFWICCDACEKWFHGKCVKITPARAEHIKQYKCPSCSNKRARP

>GrAL4b Gorai.009G041700.1 *Gossypium raimondii*

MWLGRVAQVSELHGHRHGLGHGCMSLIRMSTRPELILLLKTKTKIPLCKSFISLLFLSLSDRLLPFSLPLCYSISTLQHNFSFKEKTKKGRKVSARIMDGGVPYNPRTVEEIFRDFKGRRAGMIKALTTDVEEFYQQCDPEKENLCLYGFPSEQWEVNLPAEEVPPELPEPALGINFARDGMQEKDWLSLVAVHSDSWLLSVAFYFGARFGFDKADRKRLFNMINDLPTVFEVVTGAAKKQTKEKSLVSNHSGSKSKSNSKFLSFPLCLEKLQRGSEPLPKYSKAVPSKDEDDDGLEEEEEEHGETLCGACGESDGADEFWICCDICEKWFHGKCVKITPARAEHIKQYKCPSCSNKRARP

>GrAL4c Gorai.001G021300.1 *Gossypium raimondii*

MDAYNPRTVEEVFRDFKGRRAGMIKALTTDVGEFFQQCDPEKENLCLYGFPSEQWEVNLPAEEVPPELPEPALGINFARDGMQERDWLSLVAVHSDAWLLSVAFYFGARFGFYKTDRERLFNMINDLPTIFEVVTGSAKKHTKEKLLVSNHSSKKSKSNSKQGSEAQTKYSKAVPSQDEVDDGIEEEDDDEHGEALCGACGENYADDEFWICCDICEKWFHGKCVKITPARAEHIKQYKCPSCSGNKSARPS

>GrAL4d Gorai.010G097500.1 *Gossypium raimondii*

MDGGASYNPRTVEEVFRDFKGRRAAMIKALTTGISLLSLFSYSFVIFLKERPLSFLSYLSKFVCFFMLITFSGVVLLADVEEFYKQCDPEKENLCLYGYPSEQWEVNLPAEEVPPELPEPALGINFARDGMQEKDWLSLVAVHSDAWLLAVAFYFGARFGFDKADRKRLFNMINDLPTIFEVVTGATKKQTKEKSSVSNHSSNKSKSNSKRGSESQPKYSKAAASKDEVEDGMEDEDDEEHGETLCGACGENYAADEFWICCDICEKWFHGKCVKITPARAE

HIKQYKCPSCSNKRARP

>GrAL4e Gorai.013G226400.1 *Gossypium raimondii*

MDGGTLHSLRTVEEVFKDFKGRRAGIIKALTADVGEFFEQCDPEKENLCLYGLPTEQWEVTLPAEEVPPELPEPALGINFARDGMQEKDWLALVAVHSDAWLLAVAHYFGARFGFDKADRKRLFTMINDLPTIFEIVSGSAKKQTKEKSSVSNHSSNKSKSSGKARGSESAKYSKSVQLKDEEEGLDEEEEEEHGDTLCGACGENYASDEFWICCDICEKWFHGKCVKITPARAEHIKQYKCPSCSNKRARP

>GrAL6a Gorai.005G120300.1 *Gossypium raimondii*

MEGVPHPIPRTVEEVFNDFRGRRSGLIKALTTDVDKFYQQCDPEKENLCLYGLPNETWEVNLPVEEVPPELPEPALGINFARDGMQEKDWLSLVAVHSDSWLLAVAFYFGARFSFGKNERKRLFRMINELPTIFEVVTGNVKQPTEQYANHNSSGKSKSSAKVVQSSKIETWMFFSVVFSSPLYVLLLIDVTFMASYACCWQSRQSESESKVVKMSASKDEDESGEDEEDDEQGATCGACGDSYGTDEFWICCDICERWFHGKCVKITPAKAEHIKQYKCPS

CSSKRARV

>GrAL6b Gorai.010G143400.1 *Gossypium raimondii*

MEGIPHPIPRTVEEVFNDFKGRRSGLIKALTTDVDKFYQQCDPEKENLCLYGLPNETWEVNLPVEEVPPELPEPALGINFARDGMQEKDWLSLVAVHSDSWLLAVAFYFGARFGFGKNERKRLFQMINDLPTIFEVVTGNVKQLKDQSANHNGSSKSKSSAKSRQSEPQSKMVKMSPPSKDEDESGEEDEEDDEQGATCGACGDSYGTDEFWICCDICERWFHGKCVKITPAKAEHIKQYKCPSCSSKRARV

>GrAL6c Gorai.009G168100.1 *Gossypium raimondii*

MEGIPHPIPRTVEEVFNDFKGRRAGLIKALTTDVDKFYQQCDPEKENLCLYGLPNETWEVNLPVEEVPPELPEPALGINFARDGMQEKDWLSLVAVHSDSWLLAVAFYFGARFGFGKNERKRLFLMINDLPSIFEVVTGNVKQSKDQSANHNSSGKSKSSAKVSRQSEPQSKMVKMSPPSKDEDESGEEEEEDDEQGATCGACGDSYGTDEFWICCDICERWFHGKCVKITPAKAEHIKQYKCPSCSSKRARV

>PtAL1a Potri.008G069200 *Populus trichocarpa*

MASILTSPRTVEEIFKDYSARRSALVRALTIEADEVYIQCDPEKENLCLYGHPTESWEVTLPAEEVPPELPEPALGINFARDGMSRKDWLSLVAVHSDCWLLSMAFYFGARLNRNDRKRLFSMINDLPTLFEIVTGRKPAEDKPSAESGSKSRNNTKRSIDGQARSNSKLSYVEDEDEHGDTICGSCAGNYNADEFWIGCDICERWYHGKCVKITPAKAESIKQYKCPSCSTKKSRH

>PtAL1b Potri.010G188100 *Populus trichocarpa*

MASPRTVEEIFKDYNARRSALVRALTIEADEVYLQCDPEKENLCLYGHPNESWEVTLPAEEVPPELPEPALGINFARDGMTRKDWLSLVAVHSDSWLLSVGFYFGARLNRNERKRLFSMVNDLPTLFEIVTGRKPVEDKPSADGGSKSRNNTKRSTDGQARSNSKLSYVEDEDEHGDTLCGSCGGNYNADEFWIGCDICERWYHGKCVKITPAKAESIKQYKCPSCSTKKSRH

>PtAL1c Potri.006G100900 *Populus trichocarpa*

MEMTSSPRTVEEIFKDFSARRAAVVRALTYDVDGFYGLCDPDKENLCLYGHPNETWEVTLPAEEVPPELPEPALGINFARDGMNRKDWLSLVAVHSDSWLLSVAFYLGARLNRNERKRLFSLINDLPTVFEVVTERKPVKEKPSVDSGSKSRGSIKRSSDGLTKSNPKLTEDSFEEEEDEHTQTLCGSCGGNYNSDEFWIGCDVCERWYHGKCVKITPAKAESIKQYKCPSCMKRSRP

>PtAL1d Potri.016G116900 *Populus trichocarpa*

MEMASSARTVEEIFKDFSARRAAVVRALTHDVDAFYGLCDPDKENLCLYGHPSETWEVALPAEEVPPELPEPALGINFARDGMNRKDWLSLVAVHSDSWLLSVAFYLGARLNRNERKRLFSLINDLPTVFEVVTERKPVKEKPSVDSGSKSRGSIKRSSDGQMKSNPKLMEDSYEDEEDHTETLCGSCGGNYNADEFWIGCDVCERWYHGKCVKITPAKADSIKQYKCPSCMKRSRS

>PtAL4a Potri.006G145300 *Populus trichocarpa*

MDGGAQYNPRTVEEVFRDLKGRRAGMIKALTTDVEEFYQRCDPEKENLCLYGFPNEQWEVNLPAEEVPPELPEPALGINFARDGMQEKDWLSLVAVHSDAWLLAVAFYFGSRFGFDKTDRQKRKGKRLFTMINDLPTIFEIVTGAVKKQAKEKSSVSNHSSNKSKSSSKGRASDSVKYLKGQPKDEEEGLDEEEEEHGDTLCGACGENYAADEFWICCDICEKWFHGKCVKITPARAEHIKQYKCPSCSNKRARP

>PtAL4b Potri.018G050200 *Populus trichocarpa*

MDGGASYNPRTVEEVFRDFKGRRAGMIKALTTDVEEFYQQCDPEKENLCLYGFPSEQWEVNLPAEEVPPELPEPALGINFARDGMQEKDWLSLVAVHSDAWLLSVAFYFGSRFGFDKADRKRLFSMINDLPTIFEVVTGTAKKQVKEKSSVSNHSSNKTKSNSKQRGSESQGKYSKAMQAKDEDDEGLDEEDEEDHGETLCGACGENYASDEFWICCDICEKWFHGKCVKITPARAEHIKQYKCPSCSNKRARP

>PtAL4c Potri.006G223500 *Populus trichocarpa*

MDGGASYNPRTVEEVFRDFKGRRAGMIKALTTDVEEFYQQCDPEKENLCLYGFPSEQWEVNLPAEEVPPELPEPALGINFARDGMQEKDWLSLVAVHSDAWLLSVSFYFGSRFGFDKADRKRLFNMINDLPTVFEVVTGTAKKQVKEKSSVSNHSSNKTKSNSKRGSESQGKFSKVMQAKDEDGEGLDEEDEEEHGETLCGACGENYASDEFWICCDICEKWFHGKCVKITPARAEHIKQYKCPSCSNKRARP

>PtAL6a Potri.003G044700 *Populus trichocarpa*

MEAIPHPIPRTVEEVFSDFKGRRSGLIKALTTDVEKFYQQCDPDKENLCLYGLPNETWEVNLPVEEVPPELPEPALGINFARDGMQEKDWLSLVAVHSDSWLLAVAFYFGARFGFGKNERKRLFQMINELPTIFEVVSGNVKQPKDQSATHNNSGKSKSSGKMQSRQPESQTKAVKVSAPPKEDYESGEEEEEDDEQGATCGACGESYGTDEFWICCDICEKWFHGKCVKITPAKAEHIKQYKCPSCSGKRARV

>PtAL6b Potri.004G230500 *Populus trichocarpa*

MEGTPHPIPRTVEEVFSDFKGRRSGLIKALTSDVEKFYQQCDPDKENLCLYGLPNETWEVNLPVEEVPPELPEPALGINFARDGMQEKDWLSLVAVHSDSWLLAVAFYFGARFGFGKNERKRLFQMINELPTIFEAVSGNVKQPKDQTATHNNSGKSKSSGKMQSRQPESQTKAIKVSAPPKEDYESGEEEEEDDEQGATCGACGESYGTDEFWICCDMCEKWFHGKCVKITPAKAEHIKQYKCPSCSGKRARV

>BdAL1a Bradi1g03900.1 *Brachypodium distachyon*

METARGPAAASRPRTVEEIFKDFSNRRLGLVRALTSDVEQFYGLCDPDKENLCLYGNPDGSWSVTLPAEEVPSELPEPALGINFARNGMHRRDWLSLVAVHSDSWVLAVAFFYGARLNANERKRLFSMINDLPNVYESMVDRKQSRAKSGVDGNGKSRHSSKQTKDGRAKNSRVLAQEHAEEDDEEEEHSETFCGTCGGLYDESEFWIGCDICGRWFHGKCVRITPAKAEHIKQYKCPDCSGSKKIR

>BdAL1b Bradi1g53660.1 *Brachypodium distachyon*

MEMAPAPVSPTPRTVDDIYKDFSNRRTALVRALTVDVDDFYGFCDPEKENLCLYGYPNGSWEVALPAEEVPPEMPEPALGINFARDGMNRRDWLSLVAVHADSWLLAVAFFFGARLNANDRKRLFSMINDQSTVLESLSERKHGRDNKSGGDNSGKSRHSAKRANNDVQTKNPRPVAVDVAYEDDDEHSETLCGTCSGRYNASEFWIGCDICERWFHGKCVRITPAKAEHIKHYKCPDCSSKKSRQ

>BdAL2 Bradi2g34640.1 *Brachypodium distachyon*

MDASYRRAGAGGGGGSAPRSVEDIFKDYRARRSAILRALTTDVEEFYAQCDPDKENLCLYGYANEAWEVALPAEEVPTELPEPALGINFARDGMKRSDWLALVAVHSDSWLVSVAFYYAARLTRNDRKRLFGMMNDFATIYEVVSGMRQSKDRDRSGGIDNSSRNKLQVKHTSEAVPPLPPPRVENNVREADEGYDEDDGDHSETLCGTCGGIYSAEEFWIGCDVCERWYHGKCVKITPAKAESIKQYKCPSCSSKRPRQ

>BdAL3 Bradi1g21740.1 *Brachypodium distachyon*

MDAQYNPRTVEEVFRDYKGRRNGLARALTVDVEDFFRQCDPEKENLCLYGFPNEHWEVNLPAEEVPPELPEPALGINFARDGMQEKDWLSMVAVHSDAWLLSVAFYFGARFGFDKNDRKRLFGMINDLPTIFDVVSGKSKTKAPSNNNHSNSKSKSSNKMQKTSEPRAKQPKPQPKEEDREDEAPDAGQDGGAIAGGGDEHGETLCGACGDNYGTDEFWIGCDMCERWFHGKCVKITPAKAEHIKQYKCPSCTGTNGSGSNKRARLSS

>BdAL6a Bradi2g25120.1 *Brachypodium distachyon*

MDPGAGAGAPYASRTAEEVFRDFRGRRAGMIKALTQEVEKFYQLCDPEKENLCLYGYPNETWEVTLPAEEVPPEIPEPALGINFARDGMNEKDWLALVAVHSDSWLLSVSFYFAARFGFDKEARRRLFNMINNLPTIFEVVTGAAKKQTKEKGPNSTNKNNKPSTKIPSRPESHSKAPKVVAPPKDEDDSGEDYGEEEEEERDNTLCGTCGTNDGKDEFWICCDNCERWYHGKCVKITPARAEHIKHYKCPDCSNKRARA

>BdAL6b Bradi2g57280.1 *Brachypodium distachyon*

MEGAGGGAAYPSRTAEEVFRDLRGRRAGMIKALTEEVEKFYKLCDPEKENLCLYGYPNETWEVTLPAEEVPPEIPEPALGINFARDGMNGKDWLALVAVHSDSWLLAVAFYFGARFGFDRETRRRLFNMVNNLPTIYEVVTGVAKKQAKEKTPNSSSKSNKPTSKVQSRVEPRSSSKAKVSAPKDEEDSGDEDGDEVEEEHDNTLCGTCGTNDGKDEFWICCDNCEKWYHGKCVKITPARAEHIKHYRCPECTNGNSNKRLKP

>BdAL6c Bradi3g46110.1 *Brachypodium distachyon*

MDDAGGGLASAAVHHNARSPEDVFRDYRARRAGIVKALTTDVEKFYQQCDPEKENLCLYGLPNETWEVTLPAEEVPPELPEPALGINFARDGMIEKDWLSLVAVHSDAWLLSVAFYFGARFGFDKEARRRLFTMVNGLPTVYEIVTGVAKKQPKPSNGGSKSSKSNSKPSRQSNSNSKPAKLSHPKDEEDNGQEDAQDEEQAYLCGSCGETYANGEFWICCDICEKWFHGKCVRITPAKAEHIKHYKCPACSSKRSRE

>BdAL7a Bradi4g40230.1 *Brachypodium distachyon*

MDNGGGGGMMRATGQGAVRRTPEDVFRDYRARRAGLIKALTVDVDKFYLMCDPEKENLCLYGLPNETWEVNLPAEEVPPELPEPALGINFARDGMNDKDWLSLVAAHSDSWLLSVAFYFGARFGFDRDSRKRLFSMINNLNTIFEVVTGSDKIQPKEKTPKNGSKSNKSGSKPARQPEPNPRSSKIPLPEDNEESEGEEEKEQEDHESTMCGACGENYGQEEFWICCDLCEKWFHGKCVKITPAKAEHIKHYKCPNCSSSSKRARA

>BdAL7b Bradi4g21040.1 *Brachypodium distachyon*

MDGGGTHRTPEDVFRDFRARRAGMIKALTTDVERFYQQCDPEKENLCLYGLPNETWEVNLPAEEVPPELPEPALGINFARDGMDEKDWLSLVAVHSDAWLLAVAFYFGARFGFDKESRKRLFSMINNLSTIYEVVTGTAKKQVKEKNPKSSSKSNKSGTKLSRQPEPNSRGPKMPPPKDEDDSGGEEEEGEEHENALCGACGDNYGQDEFWICCDACETWFHGKCVKITPAKAEHIKHYKCPNCSSSSKRARA

>OsAL1a Os03g60390.1 *Oryza sativa*

MEMAPAAQVASNPRTVEDIFKDYSARRGALVRALTSDVDEFFGLCDPDKENLCLYGLANGSWEVALPAEEVPPELPEPALGINFARDGMNRRDWLSLVAVHSDSWLVSVAFFFAARLNGNERKRLFNMINDLPTVYEALVDRKHVRDRSGVDSSGKSKHSTKRTGEGQVKRSRVVAEEYEDDDEEHNETFCGTCGGLYNANEFWIGCDICERWFHGKCVRITPAKAEHIKHYKCPDCSSSSSKKTRL

>OsAL1b Os07g12910.1 *Oryza sativa*

MEMAAPVSPAPRTVEDIFKDFSGRRAGLVRALTVDVDEFYGFCDPEKENLCLYGHPNGRWEVALPAEEVPPELPEPALGINFARDGMHRRDWLSLVAVHSDSWLLSVAFFFGARLNGNERKRLFSLINDHPTVLEALSDRKHGRDNKSGADNGSKSRHSGKRANDVQTKTSRPAVVDDGYDEEEHSETLCGTCGGRYNANEFWIGCDICERWFHGKCVRITPAKAEHIKHYKCPDCSSSKKSRQ

>OsAL2 Os05g07040.1 *Oryza sativa*

MDASYRRDGRGGGGGGGGGGSAPRSVEDIFKDFRARRTAILRALTHDVEDFYAQCDPEKENLCLYGYANEAWQVALPAEEVPTELPEPALGINFARDGMNRRDWLALVAVHSDSWLVSVAFYYAARLNRNDRKRLFGMMNDLPTVYEVVSGSRQSKERDRSGMDNSSRNKISSKHTSDVARVENNIKEEDEGYDEDDGDHSETLCGTCGGIYSADEFWIGCDVCERWYHGKCVKITPAKAESIKQYKCPSCSSKRPRQ

>OsAL5 Os07g41740.1 *Oryza sativa*

MDAQYNPRTVEEVFRDFKGRRAGLVRALTADVEDFFRQCDPEKENLCLYGFPNEHWEVNLPAEEVPPELPEPALGINFARDGMQEKDWLSMVAVHSDAWLLSVAFYFGARFGFDKNDRKRLFGMINDLPTIFEVVSGKSKAKPPSANNHSNSKSKSSNKTKSSEPRAKQPKPQPQPPVKNEGREEEGGPDDEEGGGGGGGGGREEEHGETLCGACGESYGADEFWICCDICEKWFHGKCVKITPAKAEHIKQYKCPSCSGGNGGGGGVSGNGKRARPS

>OsAL6a Os05g34640.1 *Oryza sativa*

MDGGSGGPYTSRTAEEVFRDFRGRRAGMIKALTTDVEKFYQLCDPEKENLCLYGYPNETWEVTLPAEEVPPEIPEPALGINFARDGMNEKDWLALVAVHSDSWLLAVAFYFAARFGFDKEARRRLFNMINNLPTIFEVVTGAAKKQTKEKAPNSTNKPNKPSSKMQPRPESHSKAPKPPAPPKDDDESGDEYADEEEEERDNTLCGSCGTNDGKDEFWICCDSCERWYHGKCVKITPARAEHIKHYKCPDCGNKRARA

>OsAL6b Os04g36730.1 *Oryza sativa*

MDGGYGSVTIVHDARSPEDVFQDFCGRRSGIVKALTIEVEKFYKQCDPEKENLCLYGLPNGTWAVTLPADEVPPELPEPALGINFARDGMQEKDWLSLIAVHSDSWLLSVAFYFGARFGFDKKARERLFMMTSSLPTVFEVVSGGVNTQSKTANGSSKNKSGSKPPKRPNSDSKPQKQVQAKYEEENGGRGNGGDEDQAETICGACGEAYANGEFWICCDICETWFHGKCVRITPAKAEHIKHYKCPGCSNKRTRE

>OsAL6c Os02g35600.1 *Oryza sativa*

MDEGGGAGAAAAAAGNAAGAAVHHNARSAEDVFRDFRARRAGIVKALTTDVEKFYRQCDPEKENLCLYGLPNETWDVTLPAEEVPPELPEPALGINFARDGMIEKDWLSLVAVHSDAWLLSVAFYFGARFGFDKEARRRLFTMINGLPTVYEVVTGIAKKQTKVSNGSSKSNKSNPKPSKQSNSNSKPAKPPQPKDEEDSGPEGTEDEDQAYMCGACGETYANGEFWICCDVCEKWFHGKCVRITPAKAEHIKQYKCPGCSSKRSRE

>OsAL7 Os11g14010.1 *Oryza sativa*

MDGGGAHRTPEDVFRDFRARRAGMIKALTTDVEKFYQQCDPEKENLCLYGLPNETWEVNLPAEEVPPELPEPALGINFARDGMDEKDWLSLVAVHSDTWLLAVAFYFGARFGFDKESRKRLFSMINNLPTIYEVVTGTAKKQSKEKTPKTSGKSNKSGTKPSRQPEPNSRGPKMPPPKDEDDSGGEEEEEEEDHENTLCGACGDNYGQDEFWICCDACETWFHGKCVKITPAKAEHIKHYKCPNCSSSSKRARA

>ZmAL1a GRMZM2G110952 *Zea mays*

MDMAPASVYFNPRSVEEIFKDFSGRRAGLVRALTSDVDDFCRLCDPDKENLCLYGLPNGSWEVSPPAEEVPPELPEPALGINFARDGMQRRDWLTLVAVHSDSWLISVAFFYGARLNGNDRKRLFSMMSDLPSVLEAFADRKHGRDRSGVDSSGKSRHSSKRGKDGHAKSFRAAAPAAKEYDEDDDEEDEEEHTETFCGSCGGLYNASEFWIGCDICERWFHGKCVRITPAKADHIKHYKCPDCSSKKMRQ

>ZmAL1b AC225147.4_FG003 *Zea mays*

MDMAPAAVSSNPRSVEEIFKDFSGRRAGLVRALTSDVDDFCSFCDPDKENLCLYGLPNGSWEVSPPADEVPPELPEPALGINFARDGMQRRDWLSLVAVHSDSWLISVAFFYGARLNANDRKRLFSMISDLPSVFEAFADRKHVRDRSGVDSSGKSRHSSKRGNDGHAKNSRAAAPAAKEYDDDDDEEDEEHTETFCGSCGGLYNANEFWIGCDICERWFHGKCVRITPAKADHIKHYKCPDCSSKKIRQ

>ZmAL1c GRMZM2G047316 *Zea mays*

MAGPVSSAPRTVEDIYKDYAARRAGLVRALTSDVDGFYSMCDPEKENLCLYGLPNGGWEVSLPVEEVPPEMPEPALGINFARDGMRRRDWLSLVAVHSDAWLVSVAFFFAAKLNGNDRKRLFNMINDHPSVYEIMADRKGRENNPGVDNSSKSRHSTKRSNDGKIKNSRVAVGECRYENDEDHSETLCGSCSGLYNSSEFWIGCDICERWFHGKCVRITPAKAEQIKHYKCPDCSYKKSRQ

>ZmAL2a GRMZM5G893976 *Zea mays*

MADAASGRGHVVYPPRSAEDIFKDYRARRSAILRALTHEVEDFYAQCDPDKENLCLYGYANEAWEVALPAEEVPTELPEPALGINFARDGMNRRDWLALVAVHSDSWLLSVAFYYAARLNRNDRKRLFGMINELQTVFEVVSGARQQQSKERSSMDNGGRAKPVKIENNGKVTDEAYEDDSDHGETLCGTCGGIYNADEFWIGCDMCERWYHGKCVKITPAKAESIKHYKCPSCSSKRARQ

>ZmAL2b GRMZM2G017142 *Zea mays*

MDASYRRAGTGSGSAPRSVEDIYKDYRSRRSAILRALTHDVEEFYALCDPEKENLCLYGYANEAWEVALPAEEVPTELPEPALGINFARDGMNRGDWLALVAVHSDSWLVSVAFYYAARLNRSDRKRLFGMMNDLPTVFEVVSSGVKQSKERDRSGTDNGGRNKLSVKQTSEPRLENNAREPDEGYDEDDSNHSETLCGTCGGIYSADEFWIGCDVCEKWYHGKCVKITPAKAESIKQYKCPSCCNSKRPRPI

>ZmAL2c GRMZM2G148810 *Zea mays*

MPPTAAPVPEAVLLPAPSRTSTRTTAPAAPPSSAPSPTTSRSSTRCAIQVSLLPLAISPPWPSIRFGCELMVCGWGWMSLEKENLCLYGYANEAWEVALPAEEVPTELPEPALGINFARDGMNRRDWLALVAVHSDSWLISVAFYYAARLNRSDRKRLFGMMNDLPTVFEVVSGGVKQSKERDRSGTDNSGRNKLSAKQTSEPRLENNAREPDEGYDEDDGYHSETLCGTCGGIYSADEFWIGCDVCEKWYHGKCVKITPAKAESIKQYKCPSCCNSKRPRPL

>ZmAL5 GRMZM2G038066 *Zea mays*

MDSGYNPRTVEEVFRDFKGRRAGIIRALTTDAEDFFKQCDPEKENLCLYGFPNESWEVNLPAEEVPPDLPEPALGINFARDGMQEKEWLSMVAAHSDAWLLSVAFYFGARFGFNKNDRKRLYSLIDDLPMAFEIVSGKSETKAPAPPSSSNHSNIKPKSNNKKKPPEPKVKQPKPRAPAEEGEEEDGSASEGEHGETLCGACKESYGPDEFWICCDLCEKWFHGKCVKITAAKAEHIKQYKCPSCTGGGGVSNSGTKRARPS

>ZmAL6a GRMZM2G080917 *Zea mays*

MDGGGSGPAPNAAHTAEEVFRDYKARRAGMIKALTTDVERFFKLCDPEKENLCLYGYPDETWEVTLPAEEVPPEIPEPALGINFARDGMNEKDWLALVAVHSDSWLLSVAFYFGARFGFDREARRRLFSLINNMPTIFEVVTGAVKKQQAKEKTPNSSSKSNKPSSKVQSRAESRSKAKVPKDEEESGDDDGDEEAEEHDNTLCGTCGTNDGKDQFWICCDNCEKWYHGKCVKITPARAEHIKQYKCPDCTNKRVRA

>ZmAL6b GRMZM2G172001 *Zea mays*

MNGGGSGLAPNAAHTADEVFRDYKGRRAGMIKALTTDVERFFKLCDPGECARSLWFLAPGCGVIAEEASVVFLLGLRCFEKENLCLYGYPDETWEVTLPAEEVPPEIPEPALGINFARDGMNEKDWLALVAVHSDSWLLSVAFYFGARFGFDRETRRRLFSLINNMPTIFEVVTGAAKKQAKEKTPNSSSKSNRPSSKVQSRAESRSKAKVPQDEEESGDDDEDEEADEHNNTLCGTCGTNDSKDQFWICCDNCEKWYHGKCVKITPARAEHIKQYKCPDCTNKRARA

>ZmAL6c GRMZM2G016817 *Zea mays*

MDPGAGAHYSARTAEEVFRDFRGRRAGMIKALTNDVEKFYQLCDPEKENLCLYGYPNETWEVTLPAEEVPPEIPEPALGINFARDGMNEKDWLALVAVHSDAWLLAVAFYFAARFGFDKEARRRLFNMINNLPTIFEVATGVAKKQNKEKEPNNTSKSNKPSSKMTTRPESHLKATKVAPPKDEDDESGEEYEEEEERDNTLCGSCGTNDGKDEFWICCDSCERWYHGKCVKITPARAEHIKHYKCPDCSNKRARA

>ZmAL6d GRMZM2G158918 *Zea mays*

MDPGAGAHYSVRTAEEVFRDFRGRRAGMIKALTNDVEKFYQLCDPEKENLCLYGYPNETWEVTLPAEEVPPEIPEPALGINFARDGMNEKDWLALVAVHSDSWLLAVAFYFAARFGFDKEARRRLFNMINNLPTIFEVVTGVANKQNKEKGPNSTSKSNKTSSKMTSRPESHSKATKVAVPPKDDDDESGEEYEEEERDNTLCGSCGTNDGKDEFWICCDSCERWYHGKCVKITPARAEHIKHYKCPDCNNKRARA

>ZmAL6e GRMZM2G063864 *Zea mays*

MDGDGGGAGALHHHTRSPEDVFRDFRARRAGIVKALTTDVEKFYQQCDPEKENLCLYGLPNETWEVTLPAEEVPPELPEPALGINFARDGMVEKDWLSLVAVHSDAWLLSVAFYFGARFGFDKDARRRLFTMINNLPTVYEVVTGVAKKQSKAPNGSSKSSKPNSKPSKLTNSNSKPAKPAHPKEEEDSGREDAEEDQAYLCGSCGESYANGEFWICCDVCEKWFHGKCVRITPAKAEHIKQYKCPSCSTKRSRE

>ZmAL6f GRMZM2G107807 *Zea mays*

MDGDREGAAAVHHHARSPEDVFRDFRARRAGIVKALTTAASVVRADVEKFYQQCDPEKENLCLYGLPNETWEVTLPAEEVPPELPEPALGINFARDGMVEKDWLSLVAVHSDAWLLSVAFYFGARFGFDKDARRRLFTMINNLPTVYEVVTGVAKKQSKAPNGSSKSSKSNSKPSKQINSNSKPVKPAHPNEEEDSGREDAEEDQAYLCGSCGESYANGEFWICCDICEKWFHGKCVRITPAKAEHIKQYKCPSCSTKRSRE

>ZmAL7a GRMZM2G115424 *Zea mays*

MDGGAGFPGTPVPRSPEDVFRDYRARQAGLIRALTTDVEKFYVMCDPEKDNLCLYGLPNETWEVNLPAEEVPPELPEPALGINFARDGMNEKDWLSLVAVHSDSWLMSVAFYFGARFGFDKESRKRLFTMINNLPSIYEVVTGTAKKESKEKTPKSSNKTNKSGSKPSRQVEPNSRVPKMPPPKDEESEGEEGEPQEDHESALCGACGLGYDDFWICCDLCETWFHGKCVKITPNKAEHIKQYKCPSCTGSKRAKA

>ZmAL7b GRMZM2G153087 *Zea mays*

MDGGAGFPGTQPVSRSPEDVFRDYRARQAGLIRALTTDVEKFYVMCDPEKENLCLYGLPNETWEINLPAEEVPPELPEPALGINFARDGMDEKDWLSLVAVHSDSWLMSVAFYFGARFGFDKESRKRLFTMINNLPSIYEVVTGTAKKEPKEKTPKSNIKTNKSGSKPSRHAEPNSRVPKMPPPKDEESEEEEGEPQEDQESALCGACGLGYDDFWICCDLCETWFHGKCVKITPAKADHIKQYKCPSCTGSKRAKV

>ZmAL7c GRMZM2G008259 *Zea mays*

MDGGGTHRTPEDVFRDFRARRAGMIKALTTDVEKFYQQCDPEKENLCLYGLPNETWEVNLPAEEVPPELPEPALGINFARDGMDEKDWLSLVAVHSDAWLLAVAFYFGARFGFDKESRKRLFVMINNLPTIYEVVTGTAKKQTKEKTPKSSSKSNKAGPKPPRQPEPNSRGSKMPPPKDEDDSGGEEEEEEEDHENTLCGSCGDNYGQDEFWICCDACETWFHGKCVKITPAKAEHIKHYKCPNCSGSGKRARA

>SmAL3a Sm99900 *Selaginella moellendorffii*

MEGGAVANPRTVEEVFKDFKGRRAGMLKALSGDVEEFYRQCDPEKENLCLYGLPDETWEVNLPAEEVPPELPEPALGINFARDGMLRKDWLSLVAVHSDAWLYAVAFYHGARLDKSERKRLFGMMNELPTVFDTVTGRKPVKEKSSVNNSGSKSKTSVNEGGVKSSKAPPKEEDEAFDDEDEEHGDTQCGTCGGNYTSDEFWIGCDVCEKWFHGKCVKITPARAEHIKKYKCPSCSNNKRARG

>SmAL3b Sm187338 *Selaginella moellendorffii*

MESPATVEAIFEDFRGRRAGIVKALTTDVSIFCDECDPVKDLCLYGLPDGEWKVTLPVEEVPPELPEPSLGINFAKDGMKRTDWLILVAVHSDSWLYSVAFYHAARLHKADRQRLFGMINNLPTIHEVLAKPANKSSSKTKSKKAARGSSNNSNRRRKKEEDEDEEEEEQDEEEVEENSEEEEEEAFCGICADPHNTSQFWIACDSCRKWYHGSCVKVNASKAAGIKSYNCPSCAKKRARH

>SmAL5 Sm268058 *Selaginella moellendorffii*

MENAAPYNPRTVEEIFTDFKKRRAGMVKALTEDVEQFCNECDPDKENLCLYGFPDESWEVNFPAEEVPPELPEPALGINFARDGMERKAWLSLVAVHSDAWLIAVAFFYGAHFDRADRKRLFSLMNSLPTIYDTVTGKKQAQEKTNGNSSSKRASSAKSKKPPSSDEEDEDSPYDEDEEEHGDTFCGTCNGPYNADKKEFWIGCDTCQKWFHGSCVKVTPARAEHIKQYKCPSCSKRARV

>PpAL3a Phpat.001G036100.2.p *Physcomitrella patens*

MEGASISANPRTVDEVFKDFKGRRSGMLKALITDVEDFYRQCDPEKENLCLYGFPDEAWEVNLPAEEVPPELPEPALGINFARDGMQRKDWLALVAVHSDAWLLAVAFYYGARFDKNERKRLFNMINELPTVFDVVTGKKPVKEKLAVNNISGTKAKPAAKVQVTQAKPAKPAPLPQKDEEDALDDEDEEEHGDTFCGSCGGPYTADEFWIGCDICEKWYHGKCVKITPARAEHIKQYKCPSCTNKRART

>PpAL3b Phpat.007G100900.2.p *Physcomitrella patens*

MEGASMSTNPRTVDEVFKDFKGRRSGMLKALTTDVEEFYRQCDPEKENLCLYGFPDEAWEVNLPAEEVPPELPEPALGINFARDGMQRKDWLSLVAVHSDAWLLAVAFYYGARFDKNERKRLFTMINELPTVFDVVTGKKPVKEKPAVNSSGTKAKSATKVQVTQAKPAKPAPPPPKDEEEPLDDEDDEEHGDTFCGSCGGPYTADEFWIGCDICEKWFHGKCVKITPARAEHIKQYKCPSCSNKRART

>PpAL3c Phpat.002G128900.3.p *Physcomitrella patens*

MEGASMSTNPRTVDEVFKDFKGRRLGMLKALTADVEEFYRQCDPEKENLCLYGFPEEAWEVNLPAEEVPPELPEPALGINFARDGMQRKDWLSLVAVHSDAWLLAVAFYYGARFDKNERKRLFNMINELPTVFDVVTGKKPVKDKPAVNNSSGTKTKSATKVEVAQAKPAKPVPPIQKDEEDAFDDEDEEEHGDTFCGSCGGPYTADEFWIGCDICEKWFHGKCVKITPARAEHIKQYKCPSCSNKRART

>PpAL3d Phpat.011G027500.1.p *Physcomitrella patens*

MEGGSMTLNPRSVDEVFKDFKGRRSGMLKALTTDVEEFYLQCDPEKENLCLYGFPDEAWEVNLPAEEVPPELPEPALGINFARDGMQRKDWLSLVAVHSDAWLLAVAFYYGARFDKNERKRLFNMVNELPTVFDVVTGKKPVKDKPAVNNNSGNKVKSATKVQQVTQAKPAKPTPPPPKEEEDPLDDEDEEEHGDTFCGSCGGPYTADEFWIGCDICEKWFHGKCVKITPARAEHIKQYKCPSCSNKRART

>PpAL3e Phpat.006G104200.1.p *Physcomitrella patens*

MEGASVSTNPRSVDEVFKDYRGRRGGMLKALIADVEEFYRQCDPEKENLCLYGYPDESWEVNLPADEVPPELPEPALGINFARDGMQRKDWLSLVAVHSDAWLLAVAFYYGARFDKNERKRLFNLINELPTVFDVVSGKKPVKEKASVNNNNNSNNNNNNNNNNNNSGGNKIKSAAKVQRVVEPVKPKPAPPSPQDEDYFEDDEEEHGDTTCGTCGGSYTADEFWIGCDICEKWYHGKCVKITPARAEHIKQYKCPSCSNKRART

>PpAL3f Phpat.006G087600.2.p *Physcomitrella patens*

MEGASVSTNPRSVDEVFKDFRGRRAGMLKALTSEVEEFYRQCDPEKENLCLYGYPEETWEVNLPAEEVPPELPEPALGINFARDGMQRKDWLSLVAVHSDAWLLAVAFYYGARFDKNERKRLFNSINELPTVFDVVTGKKPVKEKASVVVNHNNNNNNNNNSGRNKTKSAAKVQRTAEPVKAKPAPPPPKEDDDLEDEDEEEHGDTFCGTCGGSYTADEFWIGCDICEKWYHGKCVKITPARAEHIKQYKCPACSNKRART

>PpAL3g Phpat.005G019700.1.p *Physcomitrella patens*

MEGASVSTNPRSVEEVFKDFRGRRAGMLKALTTEVEEFYRQCDPEKENLCLYGFPEETWEVNLPAEEVPPELPEPALGINFARDGMQRKDWLSLVAVHSDAWLLAVAFYYGARFDKIERKRLFNLINELPTVFDVVTGKKPVKEKSSVNNNVNSNSNNNNNSGGNKAKSAAKVQRAAETAKAKPAPPPPKEDDDLEDEDEEEHGDTFCGTCGGSYTAEEFWIGCDICEKWYHGKCVKITPARAEHIKQYKCPACSNKRAPSFP

>CrAL1 Cre10.g441050.t1.2 *Chlamydomonas reinhardtii*

MSRSPRAIYEDYVGRRKGILRALTTDIDRFWSQCDPQKENLCLYAYQDGTWACDLPAEEVPPEAPEPALGINFARDGMERKDWISLVAVHSDSWLLALAFYKGARLNRDERDELFGLINKLPTCYEVVSGRVKQTNGGPTTNAGGMKRPGGPGGPSRASARARMSDDEADEGGASGDWEDGEGDPCPACGRLYRTDEFWIACDACDTWYCGRCAKMTEKKAAQMKHWRCGQCAGPQ

>VcAL Vocar20008424m *Volvox carteri*

MASRSPRDIYEDYLGRRKAILRALTQDAEKFWHQCDPQKENLCLYGYNDGTWAVDLPAEEVPPEAPEPALGINFARDGMERSAWISLVAVHSDSWLLALAFYKGARLNREEREELFSLINKLPTCYEVVSGRVKQSVGGPTTNVGGLKRPGGPGGPSRTAPARAARQGDDDGEDIAGGSADWEDGEGDPCPSCGRLYRTEEFWIACDFCDTWYCGRCAKMTEKKASQMKQWRCNQCSGIQ

**Associated factors: ING1/2**

>AtING1 AT3G24010.1 *Arabidopsis thaliana*

MSFAEEFEANLVSLAHVLQKKYALLRDLDKSLQENQRQNEQRCEKEIEDIRRGRAGNITPNTSLTKFSEEALDEQKHSVRIADEKVTLAMQAYDLVDMHVQQLDQYMKKSDEVIRKEKEAAAATLELENNGKAGNAGEGGRGGRKKTRLATAASTAAASTGMTSSNMDLDLPVDPNEPTYCICNQVSFGEMVACDNNACKIEWFHFGCVGLKEQPKGKWYCPECATVKKSRKGR

>AtING2 AT1G54390.2 *Arabidopsis thaliana*

MAIARTGVYVDDYLEYASTFPAELQRLLNTVRELDERSQSLINQTRQQTKYCLGLASQSSKKGNGNHYNNGGLDEEETIEKMRKEIESSQENALSLCTEKVLLARQAYDLIDSHVKRLDEDLNNFAEDLKQEGKIPPDEPSVLPPLPIVPKAEKRKSFYGTPQPKKIDYRDRDWDRDRDFELMPPPGSNRKDLMPIEEQPIDPNEPTYCVCHQVSFGDMIACDNENVSLLSQHLIIYFLMYSCYDETFCSCFDSAKEVNGFTIHALASHLRPDSKGNGTAPPADSSHSHTNIICTCEKVPLASSLAPSLHDQCHQTHLSCYPTSSKCK

>BsING1 Bostr.19424s0207.1 *Boechera stricta*

MSFAEEFEANLVSLAHVLQKKYALLHDLDKSLQENLRQNEQRCEKEIEDIRRGRTGNITPNTSLTQFSEEALDEQKHSVRIADEKVALAMQAYDLVDMHVQQLDQYMKKSDEEIRKEKEAAAAALDLENSGKAGKAGEGGRGGRKKTRLATAASTAATSTGVSSSNMDLDLPVDPNEPTYCICNQVSYGEMIACDNNECKIEWFHFGCVGLKEQPKGKWYCPECATVKKSRKGR

>BsING2 Bostr.13404s0006.1 *Boechera stricta*

MAIARTGVYVDDYLEYASTFPAELQRLLNTVRELDERSQSLINQTRQQTKYCLGLASQSSKKGNGNHYNNGLDEEDTIEKMRKEIESSQENALSLCTEKVLLARQAYDLIDSHVKRLDEDLNNFAEDLKQEGKIPQDEPSVLPPLPIVPKPEKRKSFYGTPQSKKIDHRDREWDRDRDFELMPPPGSNRKDLTPIDEQPIDPNEPTYCVCHQVSFGDMIACDNENCQGGEWFHYACVGLTPETRFKGKWYCPTCRLLPQSH

>BrING1 Bra015016  *Brassica rapa*

MSFADEFESNLVSLAHVLQKKYALLRDLDKSLQENQRQNEQRCEKEIEDIRRGRTANVTPNAHFSEEAIDEQKHSVRIADEKVALAMQAYDLVDMHVQQLDQYMKKSNEQMRREKENASASNLENSGEAGKAGEGRRGGRKKTRLATAASTAAVASASTGMTSTGMDLDLPVDPNEPTYCICNQVSYGEMIACDNNECKIEWFHFGCVGLKDQPKGKWYCPDCATVKKSRKGR

>BrING2 Bra040776 *Brassica rapa*

MAIARTGVYVDDYLEYASTFPAELQRLLNTVRELDERSQSLINQTRQQTKYCLGLASQSSKKGSGNNNHYNTGLDDDETVEKMRKEIESSQENALSLCTEKVLLARQAYDLIDSHLKRLDEDLNNFAEDLKQEGKIPTDEPSVLPPLPIVPKQEKRKSFYGTPQPKKIDYRDREWDRDRDFELMPPPGSHRKDFTPIDEQPIDPNEPTYCVCHQVSFGDMIACDNENCQGGEWFHYTCVGLTPETRFKGKWYCPTCRLLPQSH

>CgING1 Cagra.0026s0010.1 *Capsella grandiflora*

MSFAEEFEANLVSLAHVLQKKYALLRDLDKSLQENQRQNEQRCEKEIEDIRRGRTGNITPNTSLTQFSEEALDEQKHSVRIADEKVALAMQAYDLVDMHVQQLDQYMKKSDEEIRKEKEAAAAALDLENSGKAGKAGEGGRGGRKKTRLATAASTAAASTAVTSTNMDLDLPVDPNEPTYCVCNQVSYGEMVACDNNECKIEWFHFGCVNLKEQPKGKWYCPDCATVKKSRKGR

>CgING2 Cagra.2694s0002.1 *Capsella grandiflora*

MAIARTGVYVDDYLEYASTFPAELQRLLNTVRELDERSQSLINQTRQQTKYCLGLASQSSKKGNGNHYSNGIDEEETIEKMRKEIESSQENALSLCTEKVLLARQAYDLIDSHVKRLDEDLNNFSEDLKQEGKIPQDEPSVLPPLPIVPKQEKRKSIYGTPQSKKIDYRDREWDRDRDFELMPPPGSNRKDLTPIDEQAIDPNEPTYCVCHQVSFGDMIACDNANCQGGEWFHYTCVGLSPETRFKGKWYCPTCRLLPQSH

>OrING1 orange1.1g025899m *Citrus sinensis*

MSFLDEFQANLESLPNILQKKYALLRDLDKSLQEIQRQNEQRCEQEIEDLMREIRAGNITPNTSLIRFSDDALDEQKHSIRIADEKVALAVQAYDLVDTHIQQLDQYLKNFDEELRRERDSVATTASPAPSLDGSTKSGRSSEGGRGGRKKTRLATAAAAAAAAVTEAAATPVANPTGMELDLPVDPNEPTYCVCNQVSYGEMVACDNPNCKIEWFHFGCVGLKEQPKGKWYCPDCAALKNRRKGR

>OrING2 orange1.1g024980m *Citrus sinensis*

MAIARTGVYVDDYLEYASTLPAELQRLLNTIRELDERSHSMINQTRQQTKYCLGLASQGNKKGNGNEEDEAIEKMRKEIEVNQDNALSLCTEKVLLARQAYDIIDSHIKRLDEDLNNFAEDLKQEGKIPADEPAILPPLPIIPKIEKRKPFYGTPQPKRLDFRDRDWDRERDRDFELMPPPGSQKREFTTPMDVDQPIDPNEPTYCVCHQVSFGDMIACDNENCQGGEWFHYACVGLTPETRFKGKWYCPTCRLLPQCQ

>CsING1 Cucsa.256060.1 *Cucumis sativus*

MSFLEEFQANLESLPNILQKKYALLHDLDQSLQGLVKQNEQRCEQEIEDIKRGVKCGNITPDTSLIRFSDEALDEQKHSIRIADEKVALAVQAYDLVDTHIQQLDQYLKNFDEKLRHERGTAAATGLPASSVDVNTKSGRGTEGGRGGRKKRNGQAAAATTEAPTTANPTGMELELPVDPNEPTYCLCNQVSYGEMVACDNPNCKIEWFHFGCVGLKEQPKGKWYCSDCAGSKGRRKGR

>CsING2 Cucsa.024030.1 *Cucumis sativus*

MAIARTGVYVDDYLEYASTLPAELQRLLNTIRELDDRSQSMIDQTRQQTKYCLGLSTQSSKKGYGNSNTDDEESAFEKLRKDIEANQDNALSLCTEKVLLARQAGDLIDSHIKRLDEDLNNFAEDLKQEGKISPDEPAILPPLPLVSKNERRRPVFITPQSKRPDYRDRDWDRERDRDFELMPPPGSHKKDFAPSLDVDQPIDPNEPTYCICHQVSFGDMIACDNENCQGGEWFHYSCVGLTPETRFKGKWYCPTCRDVSPSQ

>EsING1 Thhalv10002653m *Eutrema salsugineum*

MSFADEFEANLVSLAHVLQKKYALLRDLDKSLQENQRQNEQRCEKEIDDIRRGRTGNVTPSTSLTQFSEEALDEQKHSVRIADEKVALAMQAYDLVDMHVQQLDQYMKKSDEEMRREKEKENAAASNLESSEAGKAGEGRRGGRKKTRLATAASTAAAATTGTSSTNMDLDLPVDPNEPTYCTCNQVSYGEMVACDNNECKIEWFHFGCVGLKEQPKGKWYCPDCATVKKSRKGR

>EsING2 Thhalv10003462m *Eutrema salsugineum*

MAIARTGVYVDDYLEYASTFPAELQRLLNTVRELDERSQSLINQTRQQTKYCLGLASQSSKKGNGNNHYNNGLDEDETIEKMRKEIESSQENALSLCTEKVLLARQAYDLIDSHVKRLDEDLNNFSEDLKQEGKIPPDEPSVLPPLPIVPKPEKRKSFYGTPQSKKIDYSNREWDRDRDFELMPPPGSNRKDFTPIDEQPIDPNEPTYCVCHQVSFGDMIACDNEKNCQGGEWFHYTCVGLTPETRFKGKWYCPTCRLLPQSH

>FvING1 mrna02730.1-v1.0-hybrid  *Fragaria vesca*

MTSAKYDKILVLAEESSVIYGDRYWSTDLESLPNILQRHYAVLRNLDKSLQDIQRQNEQRCEQEIEDIKREIKSGNITPDSSLIRFSDEALDEQKHSIGIADEKVMLAVRAYDLVDTHIQQLDQYLKKIGAELRPASDAVAASAMPAPSLDGTAKSGRSGEGGRGGRKKKQATVTAPEAAPVPATATVPAPANPTGMDLDIPVDPNEPTYCLCNQVSYGDMVACDNPNCKIEWFHFGCVGLKDQPKGKWYCPDCAVVRNRRKR

>FvING2 mrna14409.1-v1.0-hybrid *Fragaria vesca*

MVIARTGVYVDDYLEYASTLPAELQRLLNTIRELEERSQTMIHQTRQQTKQCLGLSKKWNMEEDEAAIDKLLKEIESNQENAVSLCTEKVLLAKQAYDLIDSHVKRLDEDLHHFAEDLKQEGKISPDEPAILPPLPIVPKTEKRKPIYVTPQSKRFDYRDREWDRERDRDFELMPPPGSYKKEYAIPMDADQPIDPNEPTYCVCHQVSFGDMIACDNENCQGGEWFHYACVGLTPETRFKGKWYCPTCRQQPQGYSYLNSSKIRNFLNPWPLDIWFSP

>GrING1a Gorai.005G039000.1 *Gossypium raimondii*

MSFFIDEFQADLEALPNILQKKYALMRDLDKSLQEIVRQNEQRCEQEIEDMKRGLRAGNITPDTSLLRFSEEALDEQKHSVRIADEKVALAIQAYDLVDSHIQQLDQYLKKSGEELRRERENTATASPTQTPDGTTKSGRSGESGRGGRKKTRLATAAAATEVAASAANPTGVELDLPVDPNEPTYCLCNQVSYGEMVACDNPNCKIEWFHFGCVGLKEQPKGKWYCPDCATVKNRRKGR

>GrING1b Gorai.006G054900.1 *Gossypium raimondii*

MSFIDQFQADLEALPNILQKKYALMRDLDKSLQEIVRQNEQRCEQEIEDIKRGLRAENIRFSDEALDEQKHGIRIADEKVALAIQTYDLVDSHIQQLDQYLKMSDEELRRERENAATASPVPSPNSTTKSGRSNESGRGGRKKTRLATAAAAAAAATEVAAAAAENPTGMELDLPVDPNEPTYCLCNQVSYGEMVACDNPNCKIEWFHFGCVGLKEQPRGKWYCPDCAALKNRRKGRSR

>GrING2 Gorai.007G288500.1 *Gossypium raimondii*

MAIARTGVYVDDYLEYSSTLPAELQRLLNTIRELDERSQSMINQTRQQTKYCLGIAANRGNGNSYMNNGHEDEETVEKMRKDIEASQENALSLCTEKVLLARQAYDLIDSHVKRLDEDLTYFAEDLKQEGKIPPDEPAILPPLPIVPPKVEKRKFFYGTPQSKRLDYRERDWDRERDRDFELMPPPGSHKRDFATHIDVDQPIDPNEPTYCVCHQVSFGDMIACDNENCQGGEWFHYACVGLTPETRFKGKWYCPTCRTLPQCQ

>PtING1 Potri.003G174000.1 *Populus trichocarpa*

MSSFVDEFQANLEALPNILQKKYSLLRDLDKSLQEIQRQNEQRCEQEIEDIKRGVKAGNITPNTSLIRFSDEALDEQKHSIRIADEKMALAVQAYDLVDAHIQQLDQFLKLCDEDNRRERDTAAAAPALLASSLDGGTKSGRGSESGRGGRKKTRLVAAEEATETEVAVATTFANTTGMQLDLPVDPNEPTYCFCNQVSYGDMIACDNPDCKIEWFHFGCVGLKEKVKGKWYCSDCAPLKNRRRGR

>PtING2a Potri.019G033800.2 *Populus trichocarpa*

MAIARTGVYVDDYLEYANTLPAELQRLLNTIRELDDRSQSMINQTRQQTNYCLGLASQSSKKGNGSIYNCYNTNNREEDDAVEKMRKDIEANQDNALILCTEKVLLARQAYELIDSHIKRLDEDLNNFAEDLKHEGKLSPDEPAILPPLPLIVPKIEKRRNFYGTPQSKRIDFRDRYWDRERDRDFELMPPPGSHKKDFTVPVEAEQPIDPNEPTYCVCHQVSFGDMIACDNENCQGGEWFHYSCVGLTPETRFKGKWYCPTCRNLPQFQW

>PtING2b Potri.013G058800.1 *Populus trichocarpa*

MAIARTGVYVDDYLEYANTLPAELQRLLNTVRELDDRSQSMINQTRQQTNYCLGLASQSLRKGNGSIYNCYNTNNNEEDEAVEKMRKDIEANQDSALSLCTEKVLLARQAYDLIDSHVKRLDEDLNNFAEDLKQEGKLSPDEPAILPPLPIIVPKIEKRRNFYGTPQSQSKRIDFRDRDWDRERDRDFELMPPPGSHRKDFPVPVEVEQPIDPNEPTYCVCHQVSFGDMIACDNENCQGGEWFHYSCVGLTPETRFKGKWYCPTCRNLPQFQW

>BdING1 Bradi1g75507.1 *Brachypodium distachyon*

MGFLEDFQASVEALPAMLHKNYSLMRELDKSLQGVQLENEQRCQQEIEDIKHGLESGSITYEPAKLKFSDEAMEEQKHCVRIADEKVALATQTYDLVDAHIQQLDQFMRKLEELRQEKEAAAATAAAGSVVAATVAAPASAGTSRSSAADAAPKSGRSGERGRGGRKKAKIPMEQPAIDLELPVDPNEPTYCLCNQVSYGEMVACDNSDCKIEWFHFGCVGLKEQPKGKWYCPNCSMFQKKRKGK

>BdING2a Bradi1g08690.1 *Brachypodium distachyon*

MAIARTGVYVDDYLEYSSTLAGDLQRILSTMHELDERADGIMGQTKGQIKHLLGVPSHGVDRPNMVGDDEAALERMKRDIESSQDNALSLCTEKVLLARQAYDLIESHIKRLDEDLGQFAEDLKQEGKIPPDEPHILPPMPVGGRDDKRRHILGTPQATKKFREREWERGMDFDLMPPPGSSKKTATSMDADQMIDPNEPTYCVCHQVSYGDMIACDNENCEGGEWFHYSCVGLTPETRFKGKWFCPTCRNLQ

>BdING2b Bradi5g17460.1 *Brachypodium distachyon*

MAIARTGVYVDDYLEYSSTLAGDLQRILSTMHELDERADGIMGQTKEQIKHLLGVPSHGFDRPNMLGDDEAASERMKRDIESSQDNALSLSTEKVLLARQAYDLIESHIKRLDEDLGQFAEDLKQEGKIPPDEPHILPPMPVGGRDDKRRHGLSTPQATKKFREREWERVMDFDLMPPPGSNKKTVTSDADQMIDPNEPTYCVCHQVSYGDMIACDNENCEGGEWFHYSCVGLTPETRFKGKWFCPTCRNLQ

>OsING1 Os03g04980.1 *Oryza sativa*

MGFLEDFQASVEALPAMLQRNYSLMRELDKSLQGVQTGNEQRCQQEIEDIKHGLESGSITYDPAKLKFSDEAIEEQKHCVRIADEKVALASQTYDLVDAHIQQLDQFMRKLEELRQEKEAATTAAAAAAAAAASVATGTPVAATVTASAGTSTADNTPKGGRSSERGRGGRKKTAKVPTEQPAPAIDLELPVDPNEPTYCLCNQVSYGEMVACDNNDCKIEWYHFGCVGVKEHPKGKWYCPSCIGFQKKRKGK

>OsING2 LOC_Os03g53700.1 *Oryza sativa*

MAIARTGVYVDDYLEYSSTLAGDLQRILSTMRELDERAHGIMGQTKEQIKYLLGVPSHGFDRSNMDDDESASERMKKDIEASQDNALSLCTEKVLLARQAYDLIESHIKRLDEDLGQFAEDLKQEGKIPPDEPSILPAISAFSRDDKRRPGFSTPQATKKFREREWDRERGMDFDLMPPPGSNKKTTAPMDVDQTIDPNEPTYCICHQISYGDMIACDNDNCEGGEWFHYTCVGLTPETRFKGKWFCPTCRNL

>ZmING1 GRMZM2G178072_T02 *Zea mays*

MGFLEDFQASVEALPSMLHRNYSLMRELDKSLHGVQLENEQRCQQEIEDIKHGLESGSITYDPAKLKFSEEAMEEQKHCVRIADEKVALATQTYDLVDAHIQQLDQFMRKLEEIRQGKEAAAAVTAGTAVATTATPTVNAGATTADANPKSGRSGERGRGGRKKAKVPTEQSVPVPVPLPPIDLELPVDPNEPTYCLCNQVSYGEMVACDNPNCKIEWYHYGCVGVKEQPKGKWYCPNCIGFQKKRKGK

>ZmING2 GRMZM5G813111_T02 *Zea mays*

MAIARTGVYVDDYLEYSSTLAGDLQRILSTMRELDDRAHGIMGQTKEQIKYILGVSSHGYDRSNMDDDESERMKKDIEASQDNALNLCTEKVLLARQAYDLIESHIKRLDEDLGQFAEDLKHEGKIPPDEPTVLPPVPVVSRDEKRRFGFSTPQASKKFREREWDRERGMDFDLMPPPGSSKKAGTSMDVDQTIDPNEPTYCICHQISYGDMIACDNENCGGGEWFHYTCVGLTPETRFKGKWFCPTCRNLQ

>SmING1 Sm270360 *Selaginella moellendorffii*

MTGISFLEDFIGSTASLPAELQQNYSLIRQLDEQQNELQRQMHGCCAHGLEDVKQVLKAGGAAAEAVANKYASSIASVHKTCVDVANEKVALALSTYDMVDNYIQRLDKYLKKYKEDCEQAKDLQGGDEQQADVGATGEDDRQNPSTDLDIPVDPNEPTYCLCNQVSYGEMIACDNPDCKIEWFHFECAGVRERPKGKWYCPDCSQMKRRRAR

>SmING2 Sm92651 *Selaginella moellendorffii*

MAIARTGVFVDDYLEYSSSLHAEVQRHLSTMTELDVRAHNMMLQTREDTKTCLFLPTQQIKKPSPEQEEFERLKKEIEANHDNIRSLCTEKVLLAQQAYDLIDSQMKRLDEDLNQFAEDLKLEGKISPDEPAILPMLPLRDEKRKSSFFVPPGKRLELKDWERDRDTELMPPPGNYRKRSLPAPDLDQPVDPNEETYCICGQVSFGDMIACDNENCTGGEWFHYQCVGLSSETRFKAKWYCPTCTKLQRRGLLDPLA

>PpING1 Pp1s277_88V6.1 *Physcomitrella patens*

MTYLEDYISSVQTLPTELQKNYQLLRELDQKFQALQKHMQVQCAKGLEEVRKAKESGSASPDTVALQHSNEVLNVHKACLTVAEEKVFLAVQTYDLVDGHIQRLDKDLKKFEEELRRDREAAGGGLLSAEPRLDGVGGTLGRDGGDGTRFGRRKGAGGGGGGQTIPTNVNVELDLPVDPNEPTYCYCGQVSYGEMIACDNSECKIEWFHFDCVGIKERPKGKWYCSDCAVVMKRRRPKLSIGGKAGHPSTKKVHSDFQYS

>PpING2 Pp1s173_3V6.1 *Physcomitrella patens*

MTRTGVYVDDYLEFCSSLPAELQRLLSTMRELDDRSQNMIMQTREQVRACLAKKRDGLDADSSLDIQRQQIESNQTNTLKLCTEKVLLAQQAYDLIETHMKRLDEDLSSFAEDLKQEGKVLNDETYISPLTFKDEKRKVQFLTPIGKRLDSRLDRDRGLESARERERDRDRDSELMPPPGSHKKSLPAPTDVDQPIDPNEPTYCICHQVSFGDMIACDNEKCEGGEWFHYQCVGLSSETRFKGKWYCPTCRSLQKRGLLDAPS

>CrING1 Cre16.g668200.t1.1 *Chlamydomonas reinhardtii*

MTDYLKDFIDRAADVPLQLRRRLALIRDLDEKAQALHREIDEHCKRTLAEKSQQHAAKKQKQAAGEDAGGSAAAPYDVESALKRLIGLGDEKVNIANQIYDFMDNHINQLDTDLQQLDGEIEADRKELGLEGDETACEKLGIEAPQGSRPHTVGKGAADQKKKRGRKKDESTAAAAGGLPPIENEPAYCICNKPSAGQMVGCDNPECTIEWFHFECVGLTEEPKGKWYCPVCRGDLQVKSGKKSGRR

>CrING2 Cre07.g357650.t1.3 *Chlamydomonas reinhardtii*

MALHNSANVMYLESFVESTGTLPTELQRILNTIKALDEKCNDLSAEVQANTSALVSLPPAYQQPGPEYDEVLNRVAASQKLLLQFADEKVQLAQQAHDLLEMHALELERVTDDLEKELRKNNPDGGLDYLQDFTLDTGRGKTPRLDEFNMSLQQELSLPPPPAAAPAAAPKKAAAAANVASKGKRPRDEDGGGGGGAPTPAGQQVKKKAAAASMPLNSQPSTFEEYQIEALPAYEEPGAAAEANNPVPFMRPPPAGYKASSSRPQASTGGNVRYLMLEDIGDGLVGRHAELFWPDDNLWYLIEIQEVYAAQREARVLYSTGDFETLSLEETARDMHMVLIPDQLY

>VcING1 Vocar20001446m *Volvox carteri*

MTDYLKQFIDRATDVPLQLRRRLALIRDLDEKAVALHREVDEHCKRLLAEKGQQSNKKQRVANAEDPAPPYDVELALKRLLGLADEKVNIANQIYDFMDKHINQLDSDLQQLDMEIESDRRELGLEDEETACGKLGLEVPKAGDTKKKRGRRKEETAAAAAALAPIEIEPAYCICNKPSAGQMVGCDNPDCTIEWFHFECVGLKADPVGKWFCPICRGERKPGKKQGKKAA

>VcING2 Vocar20011983m  *Volvox carteri*

MALHNSANVMYLESFIESTGSLPTELQRILNTIKALDEKCNDLSSELQANVSVLLGMPHAHQQPAVSPEYEELVSRVAGAQKMLVQFAEEKVQLAQQAHDLLEVHALELERVTDDLDKELRRNNPDAGLEYLQDFALDTTRGKTPRLEDFNMSLQQELSLPPAPAPPPKKAAATQNVASKGKRPRDEDGVAGTGAVGGGAGGGGGQQAPVKKKAAVAAMQLSSQPSAFEDYPLDSLPAYEEPVAAEAPVPFMRPPPAGYKSSSSRPQAATGGLVRYLCPEDIREDLVGRHAELFWPDDNLWYLIEIQEVNVTTREARVLYSTGDFETLNLEETARDMHMVLIPDQLY

>OlING1 27695 *Ostreococcus lucimarinus*

MATTPSTRALCEATCVVDRAIASWRARDGAIDDAQRRASRACETLVAPFVEARATGEDASRDASEAQRAVKALEKHHARLRALADARVKLAQATYDAVDDHITRLDKDLATFERERGSAHRAGERTKFDLALAGEHGFEALTNAPSASPGSALASPGNPNEPRYCVCRSVSDGKMIGCDNDDCAIEWFHFACVGLNPNAEVKGKWICPPCRRKKR

**Associated factors: EBS/SHL**

>AtEBS AT4G22140 *Arabidopsis thaliana*

MAKTRPGVASKIKTGRKELDSYTIKGTNKVVRAGDCVLMRPSDAGKPPYVARVEKIEADARNNVKVHCRWYYRPEESLGGRRQFHGAKELFLSDHFDVQS101AHTIEGKCIVHTFKNYTRLENVGAEDYYCRFEYKAATGAFTPDRVAVYCKCEMPYNPDDLMVQCEGCKDWYHPACVGMTIEEAKKLDHFVCAECSSDDDVKKSQNGFTSS PADDVKVRLS LFSHLLYRCS ITYL

>AtSHL AT4G39100 *Arabidopsis thaliana*

MPKQKAPRKQLKSYKLKHINKSIQEGDAVLMRSSEPGKPSYVARVEAIETDARGSHAKVRVRWYYRPEES IGGRRQFHGAKEVFLSDHFDFQSADTIEGKCKVHSFSSYT KLDSVGNDDF FCRFEYNSTT GAFDPDRVTV FCKCEMPYNPDDLMVQCEECSEWFHPSCIGTTIEEAKKPDNFYCEECSPQQQNLHNSNSTSNNRDAKVNG KRSLEVTKSK NKHTKRPG

>BsEBSa Bostr.10089s0034.1 *Boechera stricta*

MAKTRPGVASKIKPGRKELDSYTIKGTNKVVRVGDCVLMRPSDAGKPPYVARVEKIEADARNNVKVHCRWYYRPEESLGGRRQFHGAKELFLSDHFDVQSAHTIEGKCIVHTFKNYTRLENVGAEDYYCRFEYKAATGAFTPDRVAVYCKCEMPYNPDDLMVQCEGCKDWYHPACVGMTIEEAKKLDHFVCAECSSDDDVKKSQNGFTSSPADDVKVETKRRKR

>BsEBSb Bostr.25463s0019.1 *Boechera stricta*

MAKTRPGVASSKSKLGKEDIDSYTIRGTTKVVRVGDCILMRPSDAGKPPYVARVEKIEADARNNVKVHCRWYYRPEESLGGRRQFHGAKELFLSDHYDVQSAHTIEGKCIVHTFKNYTRLENVEAEDYYCRFEYKAATGAFTPDRVAVYCKCEMPYNPDDLMVQCEGCKDWYHPACVGMTIEEAKKLEHFVCAECSSDEDGVKRSQNGFASSPTNYLKVEAKRRKR

>BsSHL Bostr.25542s0097.1 *Boechera stricta*

MPKQKAPRKQLKSHKLKHINKTIQEGDAVLMRSSEPGKPSYVARVEAIETDQRGSHAKVRVRWYYRPEESIGGRRQFHGAKEVFLSDHFDFQSADTIEGKCKVHSFSSYTKLDSVGNDDFFCRFEYNSATGAFNPDRVAVFCKCEMPYNPDDLMVQCEECSEWFHPSCIGTTIEEAKKLDHFYCEECSPQQQDLQNSNSTSKHRNAKVNGKRNLEVTKPKNKQIKRPG

>BrEBSa Bra019395 *Brassica rapa*

MAKTRPGVPSKIKTGRKELDSYTIKGTNKLVRAGDCVLMRPSDAGKPPYVARVEKIEADARNNVKVHCRWYYRPEESLGGRRQFHGAKELFLSDHFDVQSAHTIEGKCIVHTFKNYTRLENVGAEDYSCRFEYKAATGAFTPDRVAVYCKCEMPYNPDDLMVQCEGCKDWYHPACVGMTIEEAKKLDHFVCAECSSDDDVKKSQNGFAASPADDVKVETKRRKR

>BrEBSb Bra013592 *Brassica rapa*

MAKTRPGVPSKIKTGRKELDSYTIKGTNKVVRAGECVLMRPSDAGKPPYVARVEKIEADARNNVKVHCRWYYRPEESLGGRRQFHGAKELFLSDHFDVQSGHTIEGKCIVHTFKNYTRLENVGAEDYYCRFEYKAATGAFTPDRVAVYCKCEMPYNPDDLMVQCEGCKDWYHPGCVGMTIEEAKKLDHFVCAECSSDDDVKKSQNGFTASPADDVKITLASTALCAPLPCH

>BrEBSc Bra020856 *Brassica rapa*

MAKTRPGVPSKIKTGRKELDTYTIKGTNKVVRAGDYVLMRPSDAGKPPYVARVERIEADARNNVKVHCRWYYRPEESLGGRRQFHGAKELFLSDHFDVQSAHTIEGKCIVHTFKNYTRLENVGAEDYYCRFEYKAATGAFTPDRVAVYCKCEMPYNPDDLMVQCDGCKDWYHPACVGMTTEEAKLLDHFACAECSSDDDVKKSQNGFTASPADDVKVRCISLVIACVNALLVYNASFCRRSVCNLVLSVQFDGALLSVKDTILGSVWICYLLNVAPVLTAKHSIC

>BrSHL Bra010705 *Brassica rapa*

MENISGLMAFDMGELRNNLPKKRGLSRFYSGKARSYVCIFDVKCLEDLKKPTQLLDIDDDDVAYTKRKKKNKQSSSSSFSAAVNSNVNYQNYPCRRVSSSTHCSSPCMPKQKAQKKQLKSYKLKHINRTIQEGDAVLMRSSEPGKPSYVARVEAIEATDARGSNARVRVRWYYRPEESMGGRRQFHGAKEVFLSDHFDLQSADTIEGKCKVHSFSSYTKLSSVGNDDFFCRFEYNSATGAFIPDRVAVFCKCEMPYNPDDLMVQCEECSEWFHPSCIGTTIEAAKKLDHFYCEECSPEQQ DLDNSNSTSKTTDAKVNTKRSLEVSKTRNKHAKRSG

>CgEBSa Cagra.8209s0004.1 *Capsella grandiflora*

MAKTRPGVASKIKPGRKELDSYTIKGTNKVVRVGDCVLMRPSDAGKPPYVARVEKIEADARNNVKVHCRWYYRPEESLGGRRQFHGAKELFLSDHFDVQSAHTIEGKCIVHTFKNYTRLENVGAEDYYCRFEYKAATGAFTPDRVAVYCKCEMPYNPDDLMVQCEGCKDWYHPACVGMTIEEAKKLDHFVCAECSSDDDVKKSQNGFNSSPADDVKVEPKRRKR

>CgEBSb Cagra.4137s0030.1 *Capsella grandiflora*

MAKTRPGVASSAKGKLGKKDIDSYTIRGTTKVVKVGDCVLMRPSDAGKPPYVARVEKIEADARNNVKVHCRWYYRPEESLGGRRQFHGAKELFLSDHYDVQSAHTIEGKCIVHTFKNYTRLENVGAEDYYCRFEYKAATGAFTPDRVAVYCKCEMPYNPDDLMVQCEGCKDWYHPACVGMTIEEAKKLEHFVCVECSSDEDGVKRSQNGFASSPTNDLKVEAKRRKR

>CgSHL Cagra.2374s0001.1 *Capsella grandiflora*

MPKQKAPRKQLKSYKLKHINKTIQEGDAVLMRSSEPGKPSYVARVEAIETDARGSHAKVRVRWYYRPEESIGGRRQFHGAKEVFLSDHFDFQSADTIEGKCKVHSFSSYTKLDSVGNDDFFCRFEYNSATGAFIPDRVAVFCKCEMPYNPDDLMVQCEECSEWFHPSCIETTIEEAKKLDHFYCEECSPQEQDLQNSNSTSKHRDVKVNGKRNLEVTKPKNRQTKRPG

>OrEBS orange1.1g027093m *Citrus sinensis*

MAKTKPGKKDLDSYNIKGTNKVVRPGDCVLMRPADSDKPPYVARVEKIEADHRNNVKVRVRWYYRPEESIGGRRQFHGAKELFLSDHYDVQSAHTIEGKCTVHTFKNYTKLENVGAEDYFCRFEYKAATGGFTPDRVAVYCKCEMPYNPDDLMVQCEGCKDWFHPSCMGMTIEEAKKLDHFLCSDCSSDVDAKRSLNTFSVSPSVEAKVRAHMFHFVYVVYILQDKNQ

>OrSHL orange1.1g027973m *Citrus sinensis*

MAKPKAPRRTLESYTVKSISKTIKPGDCVLMRPSEPSKPSYVAKIERIESDARGANVKVHVRWYYRPEESIGGRRQFHGSKEVFLSDHHDIQSADTIEGKCTVHSFKSYTKLDAVGNDDFFCRFEYNSSSGAFNPDRVAVYCKCEMPYNPDDLMVQCEGCSDWFHPNCINMTAEEAKRLDHFFCESCSTEGQKKLQNSQANGRHSDAKVETKRRRR

>CsEBSa Cucsa.332660.1 *Cucumis sativus*

MAKTKPPKKDLDSYTIRATNKIVRAGDCVLMRPSETSKLPYVALVEKIEADNRNNIKVRVRWYYRPEESIGGRRQFHGAKELFLSDHYDVQSAHTIEGKCIVHSFKNYTKLDNVGAEDYYSRFEYKAATGAFTPDRVAVYCKCEMPYNPDDLMVQCEGCKDWYHPACVSMTIEEAKKLDHFVCSECGSDGDIKKNENAFSASPVADGKLESKRRKR

>CsEBSb Cucsa.148810.1 *Cucumis sativus*

MAKTKPGKKDLDSYTIKGTNKIVRHGDCVLMRPSDSDKPPYVARVEKIEADHRNNVKVRVRWYYRPEESIGGRRQFHGAKELFLSDHFDVQSAHTIEGKCTVHTFKNYTKLENVGAEDYFCRFEYKAATGGFTPDRVAVYCKCEMPYNPDDLMVQCEGCKDWFHPSCMGMTIEEAKKLDHFLCSDCSSENEAKRSLNAFPVSPSAEAKVEPKRRKR

>CsSHL Cucsa.377780.1 *Cucumis sativus*

MAKPKAPRQTLDSYTVKRINKTIKAGDCVLMRPSEPSKPSYVAKIEKIEADSRGANVKVHVRWYYRPEESIGGRRQFHGSKELFLSDHFDVQSADTIEGKCTVHTFKNYTKLDAVGNDDYFCRFDYNSTTGAFNPDRVAVYCKCEMPYNPDDLMVQCENCSDWFHPACIEMTTEEAKKLDHFYCESCSSEGQKKLQNSQSTSKVAETKVDTKRRRR

>EsEBSa Thhalv10026181m *Eutrema salsugineum*

MAKTRPGVASKIKPGRKELDSYTIKGTNKVVRVGDCVLMRPSDAGKPPYVARVEKIEADARNNVKVHCRWYYRPEESLGGRRQFHGAKELFLSDHFDVQSAHTIEGKCIVHTFKNYTRLENVGAEDYYCRFEYKAATGAFTPDRVAVYCKCEMPYNPDDLMVQCEGCKDWYHPACVGMTIEEAKKLDHFVCAECSSDDDVKKSQNGFTASPADDVKVETKRRKR

>EsEBSb Thhalv10028947m *Eutrema salsugineum*

MAKTRPGVASSKSTPGKKEIESYTIRGTNKVVQAGDCVLMRPSDAGKPPYVARVEKIEADARNNVKVHCRWYYRPEESLGGRRQFHGVKELFMSDHYDVQSAHTIEGKCIVHTFKNYTRLENVGAEDYYCRFEYKAATGAFTPDRVAVYCKCEMPYNPDDLMVQCEGCKDWYHPACVGMTIEEATKLEHFACTECSSDEDVKRSQNGFASSPTNDLKVEAKRRKR

>EsSHL Thhalv10026157m *Eutrema salsugineum*

MPKQKAPKKQLKSYKLNHINRTIQEGDAVLMRSSEPGKPSYVARIEAIETGARGSHARVRVRWYYRPEESIGGRRQFHGAKEVFLSDHYDLQSADTIQGKCKVHSFSSYTKLDSVRNDDFFCRFEYNSATGAFNPDRVAVFCKCEMPYNPDDLMVQCEDCSEWFHPSCIGTTIEAAKKLDHFYCQECSPEQQDLDNSNSTSKKSDDKVKTKRSLEVTKTRNKHTKRSG

>GrEBSa Gorai.009G181300.1 *Gossypium raimondii*

MAKTRPGISGTKPKQGKKDLDSYTIRGTNKVVRVGDCVLMRPSDTGKPPYVARVEKIEADSRNNVKVRVRWYYRPEESLGGRRQFHGAKELFLSDHYDVQSAHTIEGKCIVHSFKNYTKLEDVGAEDYYCRFEYKAATGAFTPDRVAVYCKCEMPYNPDDLMVQCEGCKDWYGVLCIFEFLVPMGGPIIFKISIGSYYCSYRYHPACVDMTIEEAKMLDHFVCSECSEDDLKRSQNGFHPSPVSDVKVDAKRRKR

>GrEBSb Gorai.010G183600.1 *Gossypium raimondii*

MAKTRPGVSAPKLKAGKKDLHSYTIRGTNKIVRVGDCVLMRPSDIGNPPYVARVEKIESDNRNNVKVRVRWYYRPEESLGGRRQFHGAKELFLSDHHDVQSAQTVEGKCIVHSFKNYTKLENVGAEDYYWRFEYKAATGAFTPDRVAVYCKCEMPYNPDDLMVQCEGCKDWYHPACVGMTIEEAKMLEHFVCFECSEDDFKQFQNGFHASPVSDAKVESKRHKR

>GrEBSc Gorai.006G082500.1 *Gossypium raimondii*

MAKTRPGSLAPKSKPGKKDLDSFTIRGTNKVVRVGDCVLMRPPDNGKPPYVARVEKIESDSRNNVKVRVRWYYRPEESLGGRRQFHGAKELFLSDHYDVQSAQTIEGKCIVHSFKNYSKLENVGAEDYFCRFEYKAVTGAFTPDRVAVYCKCEMPYNPDDLMVQCEVCKDWYHPACVDMTIEEAKMLDHFVCFECTEDDAKRSQNGFHSSPVSDSDEKVETKRRKR

>GrEBSd Gorai.001G216500.1 *Gossypium raimondii*

MAKTKPGKKDLDSYAIKGTNKVVRPGDCVLMRPSDSDKPPYVARVEKIEADHRNNVKVRVRWYYRPEESIGGRRQFHGAKELFLSDHYDVQSAHTIEGKCIVHTFKNYTKLENVGAEDYFCRFEYKAATGGFTPDRVAVYCKCEMPYNPDDLMVQCEGCKDWFHPSCMEMTIEEAKGLDHFLCSDCTSEDDAKRSMNTFPVSASLEPKVEPKRRKR

>GrSHLa Gorai.002G005000.1 *Gossypium raimondii*

MAKGKAPRRTLDSYTLRNINKTIKAGDCVLMRPAERSKPQYVARIERIEADARGGNVKVHVRWYYRPEESIGGRRQFHGSKEVFLSDHYDVQSADTIEAKCTVHSFKSYTKLDAVGNDDFFCRFEYNSATGAFNPDRVAVYCKCEMPYNPDDLMVQCEGCSDWFHPACIEMTAEEVKLLDHYFCETCLSDDPKKLQNSLASSVDLDTKTCSDYMSKFKFRILYRGKDLTSEEREEKYKVIFFK

>GrSHLb Gorai.013G034100.1 *Gossypium raimondii*

MAKAKAPRRPLESCTIKHINKTINAGDCVLMRPADQSNPQYVARIERIDADARGGNVKVLARWYYRPEESIGGRRQFHGSKELFLSDHYDVQSADTIEGKCTVHSFKSYTKLDSVGNDDFFCRFEYNSSTGAFNPDRVAVYCKCEMPYNPDDLMVQCEDCSDWFHPACIEMTAEEAKILDHFFCETCSSSGQKKLQNSHAASRPSDTKVDAKRRRR

>GrSHLc Gorai.005G246700.1 *Eutrema salsugineum*

MVKAKAPRRTLDSYAVKHINKIIKAGDCVLMRPADQSKPQYVSRIERIEADARGGNVKVHVRWYYRPEESIGGRRQFHGSKELFLSDHYDVQSADTIEGKCTVHSFKSYTKLDAVGNDDFFCRFEYNSSTGSFNPDRVAVYCKCEMPYNPDDLMVQCEGCSDWFHPACIEMTAEEAKRLDHFFCESCSSEGQKKLQNSHAASRHSDTKVDTKRRRR

>FvEBS mrna19505.1-v1.0-hybrid *Fragaria vesca*

MAKTRPGKKDIDAYTIRGTNKVVRAGDCVLMRPSDVGKPQYVARIEKIESDSKSNAKVKVRWYYRPEESIGGRRQFHGSKELFLSDHYDMQSANTIEGKCVVHSFKNYTKLENVGAEDYYCRFEYKAATGAFTPDRVAVYCKCEMPYNPDDLMVQCEECKDWFHPACVNLSNEVAKKLEHYICSECSADEDVKKPENSYSASFADDVKDFHVYLWLNYVHVQVDILIA

>FvSHL mrna21875.1-v1.0-hybrid *Fragaria vesca*

MAKPKAPRQTLNSYTVKPINKTVRAGDCVLMRPSEPGKPSYVAKIERIEADSRGANVKVHVRWYYRPEESIGGRRQFHGSKEVFLSDHHDVQSADTIEGKCTVHTFRGYSKLDAVGNDDFFCRFEYNSTTGSFNPDRVAVYCKCEMPYNPDDLMVQCEGCNDWFHPACIDMSAEEAERLEHFFCESCSPEGQKKLENSHTVSRQLDTKVETKRRRR

>PtEBSa Potri.004G004900.1 *Populus trichocarpa*

MAKTRPGGLISKPKTGKRDLDSYTIRGTTKVVRVGDCVMMRPSDTGRPSYVARIEGMEADSRNNVKVRVRWYYRPEESLGGRRQFHGAKELFLSDHYDVQSAHTIEGKCIVHSFKNYTKLENVGAEDYYCRFEYKAATGGFTPDRVAVYCKCEMPYNPDDLMVQCEGCKDWYHPACVDMTIEEAKKLDHFVCSECASDDDVKRSQNGFSVSSVTDVKVENKRRKR

>PtEBSb Potri.011G010800.1 *Populus trichocarpa*

MAKTRPGGIISKPKTGKRDLESYTIRGTTKVVRAGDCVLMRPSDTGRPSYVAKIEAIEADSRNNVKVRVRWYYRPEESLGGRRQFHGAKELFLSDHYDVQSAHTIEGKCIVHSFKNYTKLENVGAEDYYCRFEYKAATGGFTPDRVAVYCKCEMPYNPDDLMVQCEGCKDWYHPACVDMTIEEAKKLDHFMCSECASDDDVKRSQNGFSASSLAEVKVENKRRKR

>PtEBSc Potri.002G226000.1 *Brachypodium distachyon*

MAKTKPGKKDLDSYTIKGTNKVVRPGDCVLMRPSDTDKLPYVALVEKIEADHRNNVKVRVRWYYRPEESIGGRRQFHGAKELFLSDHHDMQSAHTIEGKCTVHSFKNYSKLENVGAEDYFCRFEYKASTGGFTPDRVAVYCKCEMPYNPDDLMVQCEGCKDWFHPSCMGMTIEEAKKLDHFLCSDCSSEDDAKRSMNVFPVSPSLEAKVETKRRKR

>PtEBSd Potri.014G157100.1 *Populus trichocarpa*

MAKTKPGKKDLDSYTIKGTNKVVRPGDCVLMRPSDTDKLPYVARIEKIEADHRNNVKVRVRWYYRPEESIGGRRQFHGAKELFLSDHYDVQSAHTIEGKCTVHSFKNYTKLENVGAEDYFCRFEYKASTGGFTPDRVAVYCKCEMPYNPDDLMVQCEGCKDWFHPSCMGMTIEEAKKSDHFLCSDCSSDDDAKRSLNVFPVSPSLEVKVETKRRKR

>PtSHLa Potri.004G159900.1 *Populus trichocarpa*

MAKAKAPRRTLDSYTVKPINKTVKPGDCVLMRPSDPSKPSYVAKIERIESDGRGPNVRVRVRWYYRPEESIGGRRQFHGSKEVFLSDHYDTQSADTIEGKCMVHSFKNYTKLDAVGNDDFFCRFEYNSSTGAFNPDRVAVYCKCEMPYNPDDLMVQCEGCSDWFHPACIEMSAEEAKRLDHFFCENCSSEGQKKLQNSHNTRQSDAKVSSSTAVLSVF

>PtSHLb Potri.009G121000.1 *Populus trichocarpa*

MAKAKAPRRTLDSYTVKPINKIVKPGDCVLLRPSDPSTPSYVAKIERIESDGRGANARVHVRWYYRPEESIGGRRQFHGSKEVFFSDHYDIQSADTIEGKCTVHSFKSYTKLDAVGNDDFFCRFEYNSSTGAFIPDRVAVYCKCEMPYNPDDLMVQCEICSDWFHPACIEMSAEEAKRLDHFFCENCSSESQKKLQNPHNTRQSDAKVFPFTVVLSVWQVGKHVALRQTSQKSF

>BdEBSa Bradi3g36110.3 *Brachypodium distachyon*

MAKTKQGKKDVDSYTIRGTTKVVRVGDTVLMRASESDTMPYVARIEKMETDGRGSVRVRVRWYYRPEEAKGGRRQFHGAKELFLSDHLDTQSAHTIEETCVVHSFKEYTKLNNVGPEDFFCRFDYNAASGAFHPDRVAVYCKCEMPYNPDDLMVQCEACKDWFHPSCLAMTIEQAKKLAHFMCSDCDEENDGNRPSNGHAPHCGPEADSKRQRR

>BdEBSb Bradi4g29560.1 *Brachypodium distachyon*

MAKTKQGKRDVDSYTIKGTTKVVRVGDCVLMRSSDKDNPPYVARVESLESDGRGSLRVRVRWYYRPEESKGGRRQFHGAKELFLSDHFDTQSAHTIEGQCIVHPFKTYTKLDNVGPEDFFCRFEYKAATGAFTPDRVAVYCKCEMPYNPDDLMVQCEGCKDWFHPSCMGMTIEQTKKLDYFMCSDCAKENGTKRPSYSDPASPSSDSKVQPKRRKR

>BdSHLa Bradi1g05247.1 *Brachypodium distachyon*

MSLSAPAKPLGQRKRTLKSYTLKGSDVVIKPRGTVLLKAPDSSKSPYVARVEAIEAAGSRGTNVRVKAFHGAKEVFLSGHQDVQSVDAIEGKCNVYSFPKYTKLDVVNDEDYFCRFEYNEVTGKLVPDTISVYCKCWMPCNPDDLMIQCEECTDWFHPACIGKTIKEAKKLEHFSCESCAAEKRRRLKESSEQKIEIVP

>BdSHLb Bradi1g55090.1 *Brachypodium distachyon*

MAKTRPPKKILESYTIKGSDKVIKPGDCVLMRSVDTSKPPYVARIESIEAAGSRGTNVRVRVRWYYRPEESMGGRRPFHGSKEVFLSDHYDVQSADTIEGKCNVHSFRSYTKLDSVNAEDYFCRFEYKSASGSFVPDRIAVFCKCEMPYNPDDLMIQCEECSDWFHPSCIGMTIKEAKKREHFFCQSCTTEGHGKTAENSHEATAQSEEKPVESKRRRR

>OsEBSa LOC_Os08g32620.1 *Oryza sativa*

MAKTKQGKRDVDSYTISGTNKVVRVGDCVLMRPVDSDNQPYVARVEKMELDGRGSVRVRVRWYYRPEESKGGRRQFHGAKELFLSDHFDMQSANTIEGKCVVHSFKNYTKLDNVGPEDFFCRFEYKAATGAFTPDRVAVYCKCEMPYNPDDLMVQCDDCKDWFHPSCMSMTIEQAKKLDHFVCS

DCVKENGAKRPSHAYAGSTKYEPKAESKRQRR

>OsEBSb LOC_Os09g21770.1 *Oryza sativa*

MAKTKQGKKDVESYTIKGTTKIVRVGDCVLMRASDTEKAPYVGRVERLETDGRGSVRVRVRWYYRPEESKGGRRQFHGAKELFLSDHFDTQSAHTIEGKCVVHSFKNYTKLDNVGPEDFFCRFEYKAATGAFTPDRVAVYCKCEMPYNPDDLMVQCEGCKDWFHPSCMGMTIEQAKKLDHFLCADCVKENGTKRPSNSYPASSNSDSKVEPKKRKR

>OsSHLa LOC_Os03g58530.1 *Oryza sativa*

MAKTRQPQKRVLESFTIKGPDGVIKPGDTVLMMAPDSSKKPYVARVEEIEATGPQASQVKIKVRWYYRPEESIGGRRPFHGSKEVFLSDHYDSQSADTIEGKCYVHTFRDYTKLRSVSAEDFFCRFEYKSATGSFVPDRIAVFCKCEMPYNPDNLMIQCEDCSDWFHPSCVEITIKEAKKLEHFYCKSCIAENGKDLQKSNGATVQSEEKVQSKRRRR

>OsSHLb LOC_Os07g08880.1 *Oryza sativa*

MAKSRPPKRILESYTIKGSDKVIKPGDCVLMRASDTSKPPYVARVEAIEAAGSRGTNVRVRVRWYYRPEESMGGRRPFHGAKEVFLSDHYDVQSADTIEGKCNVHSFRSYTKLDSVNAEDFFCRFEYKSATGSFVPDRIAVFCKCEMPYNPDDLMIQCEECSDWFHPSCIGMTIKDAKKLEHFFCQSCTAENGKMAENSHEATAQSEEKQVESKRRRR

>ZmEBSa GRMZM2G119357_T01 *Zea mays*

MVRMKQVKISVKKDVDSYTIRGTNKVVHVGDCVLMRASDSDKQPYVARVEKMEADGRGSVRVQVRWYYRPEESKGGRRQFHGAKELFLSDHFDLQSAHTIEGKCVVHSFKNYTKLDNVGPEDFFSRFEYKAATGSFTPDRVAVYCKCEMPYNPDDLMVQCEACKDWFHPSCMAMTIEQAKKLDHFVCSDCLKENGSKRLSNVYATSSNS

>ZmEBSb GRMZM5G871463_T02 *Zea mays*

MAKTKQGNKDVDSYTIGGTNKVVYVGDCVLMRASDSDNQPYVARVEKMEGDGRGSVRVQVRWYYRPEESKGGRRQFHGAKELFLSDHFDLQSAHTIEGKCVVHSFKNYTRLDNVGPEDFFCRFEYKAATGSFTPDRVAVYCKCEMPYNPDDLMVQCDACKHWFHPSCVAMTIEQAKKLDHFVCSDCFKENGSKRLSNAYATSPNFEPKAEPKRQRR

>ZmEBSc GRMZM2G091265_T05 *Zea mays*

MAKTKQGKRDVDAYTIKGTNKVVRVGDCVLMRPADTDNPPYVARVERMESDGRGSVRVRVRWYYRPEEAKGGRRPFHGAKELFLSDHFDTQSAHTIEGKCIVHSFKSYTKLDNVGPEDFYCRFDYKAATGAFTPDRVAVYCKCEMPYNPDDLMVQCEGCKDWFHPSCMGMTIEQAKKIDHYMCSDCAKENGAKRPSNSYSVSPNSDSKIESKRRKR

>ZmSHL GRMZM2G097726_T02 *Zea mays*

MAGKSRPPKRILESYTIKGSDRVIKPGDCVLMRASDASKPPYVARVEAIEAAGSRGTNVRVRVRWYYRPEESIGGRRPFHGSKEVFLSDHYDVQSADTIEGKCNVHSFRSYTKLDSVNAEDFFCRFEYKSATGSFVPDRIAVFCKCEMPYNPDDLMIQCEECSDWFHPACIGMTIKEAKKLEHFFCQTCTAENGKMVENSHEATAQSEEKPVESKRRRR

>SmEBSa 412601 *Selaginella moellendorffii*

MVNKGVKKVLDSYTIKGTNKTIKVGDAVLMRAQDPEKPPYVARVEQIEADTKNNAKVRVRWYYRPEESMGGRRQFHGFKELFLSDHYDVQSADTIEGKCIVHTFKNYTKLESVASEDYFCRFEYKAATGGFTPDRVAVYCKCEMPYNPDDLMVQCEECKDWFHPSCIGLPVDQVKKMESYYCPDCSPQAQDKSKPTGHKVSSSKPKLPQKVSPPQELKVDPKRRKK

>SmEBSb 420725 *Selaginella moellendorffii*

MAKSKVARKVVESYTIRGTHKVVKAGDTVFMRAPDPEKPSYVAKIERIEADARNNIKVNVRWYYRPEESMGGRRQFHGAKELFLSDHFDIQSADTIEGKCTVHSFKSYTKLESVGSDDFFCRFEYKAATGGFTPDRVPVYCKCEMPYNPDDLMVQCESCKDWFHPTCMSLSPDQVKKLESFHCPECSSSPPDEKKTKKSSPPHEAKSEPKRRKK

>PpEBSa Pp3c6_15430V3.1 *Physcomitrella patens*

MAKSKGAKKALDSYTVKGTQKVVKVGDCVLMRGQDPDKPPYVAKIEKIEADNRNNTKVRVRWYYRPEESMGGRRQFHGSKELFLSDHYDIQSADTIEGKCIVHTFKNYTKLDSVGTEDYFCRFEYKASTGGFTPDRVAVYCKCEMPYNPDDLMVQCETCKDWFHPSCMSFTPDQVKRMEKFVCPDCSLPDGDRKLRQSSPGSSPTPEHVHKPEAKRRKR

>PpEBSb Pp3c12_7090V3.1 *Physcomitrella patens*

MAKSKAAKKSLDSYTVKGTNKIVKVGDTVLMRGQDPEKPSYVARIEKIEADGRSNSNVKVRCRWYYRPEESMGGRRQFHGTKELFLSDHYDIQSADTIEGKCTVHTFKNYTKLESVGAEDYFCRFEYKASTGGFTPDRVAVYCKCEMPYNPDDLMVQCEICKDWFHPSCMSMTPDQVKKMEKFFCPDCLSQPGEKKLRLSSPRSSPAPDHGKPDAKRRKR

>PpEBSc Pp3c4_21200V3.1 *Physcomitrella patens*

MAKSKAAKKSLDSYTVKGTNKVVKVGDTVLMRGQDPEKPPYVAKIEKIEADGRNNSNVKVRCRWYYRPEESMGGRRQFHGTKELFLSDHYDIQSADTIEGKCTVHTFKNYTKLESVGAEDYFCRFEYKASTGGFTPDRVAVYCKCEMPYNPDDLMVQCEICKDWFHPSCMSMTPDQVKKMEKFFCPDCISQSGEKKVRQSSPRSSPATDHVKPDAKRRKR

>PpEBSd Pp3c1_31190V3.1 *Physcomitrella patens*

MAKPKNGKKTLDSCVIKGTRKIVKVGDTVLMRSEDPDKPPYIAKVENIEGDSRGNVKVQVRWYYRPEESMSGRKQFHGQKEVFLSDHYDVQSADTIEGKCIVHSFKNYTKLEAVSAEDYFCRFEYKATTGGFTPDRVAVYCKCEMPYNPDHVMLECNSCKDWFHRHCVGLSEEQVKHVDRYICPGCAPETVKKSNGPSHMTPDAKPEPKRQRR

>PpEBSe Pp3c2_7400V3.1 *Physcomitrella patens*

MAKPKNGKKTLDSCVIKGTRKIVKVGDTVLMRSEDADKPPYIAKVEKIEGDSRGNVKVRVRWYYRPEESMSGRKQFHGQKEVFLSDHYDVQSADTIEGKCIVHSFKNYTKLETVSAEDYFCRFEYKATTGGFTPDRVAVYCKCEMPYNPDHVMLECNSCKDWFHRHCVGLSEEQVKHVDRYICPTCAPETVKKSNGSSHKTPDAKPDLKRQRR

>PpEBSf Pp3c2_7410V3.1 *Physcomitrella patens*

MAKPKNGKKTLDSCVIKGTRKIVKVGDTVLMRSEDADKPPYIAKVEKIEGDSRGNVKVRVRWYYRPEESMSGRKQFHGQKEVFLSDHYDVQSADTIEGKCIVHSFKNYTKLETVSAEDYFCRFEYKATTGGFTPDRVAVYCKCEMPYNPDHVMLECNSCKDWFHRHCVGLSEEQVKHVDRYICPTCAPETVKKSNGSSHKTPDAKPDLKRQRR

>CrEBS Cre16.g675246.t1.1 *Chalamydomonas reinhardtii*

MTKHKRRELPSAVHDGEEYKPGDCVLINPDASAPAYIARIRKLIQIGAEPEQVELEVTWFYRPEEAIGGRKAFHGEAEVFDSDHQDKAPLAAILGRCNVHNVSRYESLERRDENDFFCRFTYKPRTKQFEPDRVPVYCVCELPYNPDRPMINCDNCDEWYHPQCLGLGQHVLQQDHFVCPTCTTPQQPAKKSRPGA

>VcEBS Vocar.0022s0153.1 *Volvox carteri*

MKGKRKDRRKELDSAVYNGQEYRPGDCVLINPHDDAPAYIGRIRKISQALSDPADVELEVAWFYRPEEAVGGRKIFHGESEVFESSHQDKAPLAAILDRCFVHSMETYESLKDRKETDFFCRLVYKPQTKQFEPDEVPVYCECELPYNPDRPMVMCGTCEEWYHPQCLGLGPEVFQQENFVCPKCSGSGAPAKKQRAVMAGSVDVGGGPASTA

>OlSHL 34069 *Ostreococcus lucimarinus*

MTGEVARVVIGGDVFQVNDAVLVKAPGANERYVGRIVSVAVENGAVKARLCWYYRPQETRGGRKRFHGVKELFSSDHYDWVSVNTIDAKCEVWSLREYQELEAVTEFDFYARFLYRSSRGEFRPEKVPVFCKCAEPYNPDRFMVECDQCNDWFHPECVNETKSSASQLDVWRCPDCRLSKITGEV
